# LoTToR: An Algorithm for Missing-Wedge Correction of the Low-Tilt Tomographic 3D Reconstruction of a Single-Molecule Structure

**DOI:** 10.1038/s41598-020-66793-1

**Published:** 2020-06-26

**Authors:** Xiaobo Zhai, Dongsheng Lei, Meng Zhang, Jianfang Liu, Hao Wu, Yadong Yu, Lei Zhang, Gang Ren

**Affiliations:** 1 The Molecular Foundry, Lawrence Berkeley National Laboratory, Berkeley, CA 94720 USA; 2 College of Science, Xi’an University of Science and Technology, Xi’an, 710054 People’s Republic of China; 3 School of Physical Science and Technology, Lanzhou University, Lanzhou, 730000 Gansu China; 4 University of California Berkeley, Berkley, CA 94720 USA; 5 College of Artificial Intelligence, Beijing Normal University, Beijing, China

**Keywords:** Imaging, Software, Structure determination, Cryoelectron tomography, Molecular conformation, Thermodynamics

## Abstract

A single-molecule three-dimensional (3D) structure is essential for understanding the thermal vibrations and dynamics as well as the conformational changes during the chemical reaction of macromolecules. Individual-particle electron tomography (IPET) is an approach for obtaining a snap-shot 3D structure of an individual macromolecule particle by aligning the tilt series of electron tomographic (ET) images of a targeted particle through a focused iterative 3D reconstruction method. The method can reduce the influence on the 3D reconstruction from large-scale image distortion and deformation. Due to the mechanical tilt limitation, 3D reconstruction often contains missing-wedge artifacts, presented as elongation and an anisotropic resolution. Here, we report a post-processing method to correct the missing-wedge artifact. This low-tilt tomographic reconstruction (LoTToR) method contains a model-free iteration process under a set of constraints in real and reciprocal spaces. A proof of concept is conducted by using the LoTToR on a phantom, *i.e*., a simulated 3D reconstruction from a low-tilt series of images, including that within a tilt range of ±15°. The method is validated by using both negative-staining (NS) and cryo-electron tomography (cryo-ET) experimental data. A significantly reduced missing-wedge artifact verifies the capability of LoTToR, suggesting a new tool to support the future study of macromolecular dynamics, fluctuation and chemical activity from the viewpoint of single-molecule 3D structure determination.

## Introduction

Macromolecules, including proteins in solution, naturally follow the third law of thermodynamics, *e.g*., all atoms of macromolecules are in vibration at a temperature above absolute zero. Due to the thermal vibration and fluctuation, no macromolecule has an identical 3D structure to that of another one or even to itself at different times. A similar statement was made by the ancient Greek philosopher Heraclitus: “*No man ever steps in the same river twice, for it’s not the same river and he’s not the same man*.” Based on this reason, the ultimate method for studying the protein structure and dynamics should be based on a snap-shot three-dimensional (3D) structure determination for each individual macromolecule at a time point, *i.e*., a single-molecule 3D structure. The advantage of the single-molecule approach includes the following: i) Regardless of heterogeneity nature of the sample: Each targeted particle can be used for 3D structure determination. There is no need to purify or select a homogeneous species for crystallization or classification and averaging by the current techniques, including X-ray crystallography, nuclear magnetic resonance (NMR) and cryo-electron microscopic (cryo-EM) single-particle 3D averaging. ii) A complete statistic for structural variety analysis: Since any protein particles can be targeted for structure determination instead of only a small percentage of particles, all 3Ds of particles were used  to perform structural variety analysis determination. As a result, the distribution of the structural variability can be used to reflect the energy distribution of this type of molecule. iii) The ability to determine the intermediate 3D structure of macromolecules in the process of a chemical reaction: The intermediate structures can be used as footprints to understand the detailed structural changes during unfolding/folding, aggregation and chemical reaction. iv) Identifying a protein on the surface of a cell, which may uncover important biological processes, such as a receptor for viral invaded cells.

Due to the above benefits, the single-molecule method has been considered and pursued for decades by structural biologists^1^. Unfortunately, due to the weak signal from a single molecule, the development of the technique has been hindered. As a compromise, by using an averaging approach, due to the nature of protein heterogeneity and dynamics, a small percentage of protein particles have been selected from a large pool for 3D structure determination via crystallization or classification. The averaging approach assumes that thousands of atoms within particles share identical positions. Precisely speaking, this assumption violates the third law of thermodynamics and often causes artifacts, including the elimination of flexible domains and the loss of loops in the determined structures. However, the substantial success of high-resolution structure determination by the averaging approach silenced criticism regarding its fundamental assumption; as a result, people have long believed that the averaging method is the only solution for protein structure determination. The high-resolution averaging results for a small portion of the population have been published, while the low-resolution averaging results have been treated as null results and then trashed or forgotten. This is a type of confirmation bias, in which the tendency is to search for, interpret, favor, and recall information in a way that confirms or strengthens one’s prior personal beliefs or hypotheses^2,3^. The encouragement derived from recent replication studies and null results may help scientists to stand against conformational bias, as proposed by the editor of *Nature*^4^. We expect that one could open up the way to judge the development of a single-molecule structure determination method as an additional approach.

Based on the above motivation, we developed an individual-particle electron tomography (IPET) method for the determination of a snap-shot 3D structure of an individual protein particle (without averaging or the pre-assumption of structural homogeneity)^5^. In this method, transmission electron tomography (ET) is used to image each targeted molecular particle from a series of tilting views^6,7^. Through alignment of the tilt series, the 3D density map of a targeted particle can be reconstructed using a focused electron tomography reconstruction (FETR) algorithm^5,8^. Historically, the first single-molecule 3D reconstruction, *i.e*., the yeast fatty-acid synthetase molecule, was reported by Hoppe *et.al*. in 1974 from negative-staining (NS) ET images^1,9^. This approach has rarely been followed owing to several difficulties^1,10–17^, including the radiation damaging limitation, instrument alignment stability, low success rate for data acquisition, long time for data acquisition, tracking the target particles during tilting, low-contrast images, alignment of the noisy images, computing power limitation, software availability, mechanical tilt limitation and missing-wedge artifacts. In our approach, we developed several tools and optimized some methods to extend the ET capability for single-molecule structure studies. The developments included the FETR method^5^ to avoid large-scale image distortion, the optimized sample preparation method^18–23^, instrument controlling software^24^ and an image denoising algorithm^25^. These improvements enable us to obtain a snap-shot 3D density map of many protein particles at low to intermediate resolution (up to 1–2 nm). Through structure modeling and molecular dynamics (MD) simulations, the dynamics of macromolecules have been characterized^8,26,27^. The studied proteins from the NS ET data at an ~1–3 nm 3D map resolution^8,16^ include antibodies^5,27,28^, antibody-peptide conjugates^16^, IgG1 homodimers^29^, DNA-nanogold conjugates^6^, phospholipid transfer protein (PLTP)^30^, contactin-associated protein-like 2^31^, calsyntenin-3^32^, and neurexin 1α^33^. The studied protein/macromolecules embedded in vitreous ice include DNA origami^26^, antibodies^34^, cholesteryl ester transfer proteins (CETPs)^25^, high-density lipoproteins (HDLs)^5^, low-density lipoproteins (LDLs), very-low density lipoproteins (VLDLs)^35^, intermediate low-density lipoproteins (IDLs)^34^, complexes of VLDLs and IDLs binding to an antibody^34,35^, the complex of an LDL binding to a CETP^25^ and a liposome vesicle binding to a CETP^36^. The resolutions were found to be in the range of ~3–9 nm by cryo-ET^35^. These studies showed the possibility of revealing the molecular structure variety and dynamics via the structure determination of each individual macromolecule particle.

In ET data acquisition, a single-axis tilt is often used. Due to the mechanical limitations (usually within an angle range of ±60°), 3D reconstruction often suffers from a significant missing-wedge artifact. The artifact includes anisotropic resolution and elongation along the z-direction (the direction of the electron beam)^37–39^. These artifacts could mislead the structural interpretation or decrease the accuracy in the sub-tomo classification, alignment and averaging^37^. To overcome this weakness, several approaches have been reported to reduce the missing-wedge effect, including the following: i) A redesigned sample holder, in which the maximal tilt angle could be increased to ±80° or even to ±90° by using a thin-walled carbon tube^40^ or cylindrical specimens^41^. Unfortunately, the difficulty in sample loading and the strong substrate background influence the image quality. ii) The dual-axis tilt approach, in which tilt images are acquired from two perpendicular tilt axes^42–44^. This method can partially fill the data within a wedge shape volume/region/zone (named missing-wedge data) and still leave a pyramid-shaped region unfilled^42^. Moreover, double exposure on the same imaging area results in more images sharing the maximal electron, which is limited by the radiation damage, resulting in more noise in the tilt images^42,45–47^, which would increase the difficulty in aligning the tilt series. iii) The sub-tomo averaging approach, in which hundreds to thousands of sub-volumes are clipped from a large-scale 3D reconstruction and classified, aligned and averaged into averaged 3D density maps^48^. Although near-atomic resolution can be achieved from certain ridge-body proteins^49,50^, high-resolution 3D structures have rarely been reported on flexible proteins. Moreover, the intrinsic errors in the sub-tomos, such as the nature of the structure variety of molecules, the error from the alignment, the missing-wedge artifact and the noise-induced error, will together influence the accuracy of classification, alignment, and 3D averaging^51^. iv) Computational approaches, in which the missing-wedge data can be partially restored by computer algorithms. The algorithms include the power spectrum smooth constraint^52^, the discrete algebraic reconstruction technique (DART)^53^, the posteriori expectation maximization (sMAP-EM)^38^, the compressed-sensing optimized nonuniform fast Fourier transform reconstruction (ICON) method^54^, the model-based iterative reconstruction method^55^, and the sequential statistical reconstruction method^38^. Although these algorithms partially compensate for the missing-wedge artifact, all approaches demonstrate their capabilities on either large objects (such as human organ sections or cellular sections), high signal-to-noise (SNR) ratio data (*e.g*., simulated noise-free data, material science data, 2D crystal data) or prior knowledge of the objects (*e.g*., providing a structural model). No algorithm has demonstrated capability on a small biological macromolecule 3D map reconstructed from a low-tilt angle range with a low SNR. Considering that the signal from a small molecule (<100 kDa) is extremely weak, a 3D map is even challenging to be reconstructed. As a result, single-molecule 3D structures, especially those of small molecules, are generally believed to be impossible to achieve.

Here, we report a **lo**w-**t**ilt **to**mography **r**econstruction (LoTToR) method for single-molecule 3D reconstruction. The method contains a model-free iterative procedure for restoring missing-wedge data through a set of constraints in both real and reciprocal spaces. To characterize this method, two phantoms are used to study the parameters related to the restorability of the missing-wedge data. To validate the method, real NS ET data and real cryo-ET data are used. The results show that this post-processing method for IPET can correct a missing-wedge artifact for the 3D structure determination of a single molecule.

## Theory

Is a tilt angle range of ±90° a necessary condition for a complete 3D reconstruction, in which all coordinates of the atoms of an object can be defined? The simple answer is no. Theoretically, two projections of a 3D object should be sufficient to identify a 3D object, which is how we observe 3D objects from the images received by each of our eyes. In an extremely simple case, where an object contains a single atom, three coordinates of this atom can be preciously determined from its two projections. By increasing the number of atoms of the object, some coordinates may overlap with each other. In this case, additional projections will be required to solve the overlapping problem.

**Figure 1:**
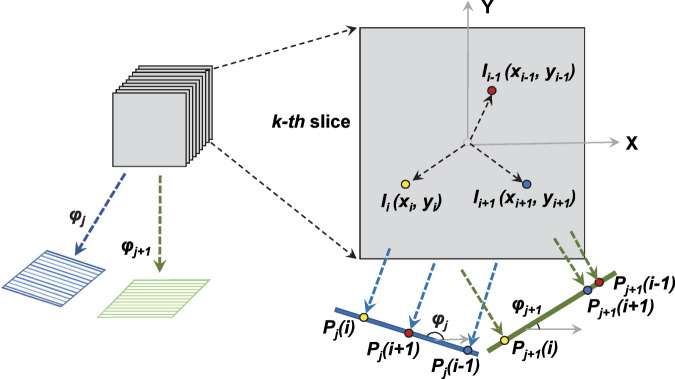
Schematic of the theory of 3D/2D reconstructions from two projections. A 3D object can be treated as a stack of 2D objects, while 3D map projections can be treated as a stack of 1D projection lines from 2D objects along the same projection angle. Given that one 2D object is composited with a total of *N*_*k*_ atoms, in the coordinates of (*x*_*i*_, *y*_*i*_) or (*r*_*i*_, θ_*i*_), two projection lines could be sufficient to determine the coordinates of each atom. There is no requirement regarding how large the angle between two projection angles needs to be.

A general mathematical description is the following. Given that the *k-th* slice of a 3D object contains a total of *N*_*k*_ atoms, in which the coordinates of the *i-th* atom are (*x*_*i*_, *y*_*i*_) or (*r*_*i*_, θ_*i*_), where $i=1,\ldots ,{N}_{k}$, the coordinate of the *i-th* atom on the 1D projection line at a tilt angle of *φ*_*j*_ (*j* = 1, 2) is *P*_*j*_(*i*), as follows (Fig. 1):

1 $${P}_{j}(i)={r}_{i}\ast \cos ({\theta }_{i}-{\varphi }_{j});(i=1,\ldots ,{N}_{k};j=1,2)$$

One projection line contains a total of *N*_*k*_ equations identical to the one above. Thus, two projections will contain a total of 2 *N*_*k*_ equations. Based on linear algebra, a total of 2 *N*_*k*_ variables (*x*_*i*_, *y*_i_) or (*r*_*i*_, θ_*i*_) could be solved from these 2 *N*_*k*_ independent equations; hence, all atoms and coordinates in this slide can be determined. Considering that the 3D reconstruction can be treated as a stack of 2D reconstructions, the determination of each slide results in the determination of all atoms’ coordinates in the 3D object. Notably, as long as the angle between two projections is not an angle that can cause any atom projections to overlap, there is no constraint on what the angle should be, such as within ±45° or ±90°. With the coordinates of all atoms in the 3D object, any projection from any projecting angle can be computed. Therefore, the tilt angle range of ±90° is not a necessary condition for a complete 3D reconstruction.

For an object containing hundreds and thousands of atoms, such as a protein, the atom projections have a high chance of overlapping with each other, especially when projected on digital images. The overlapping coordinates will reduce the total number of independent equations in Eq. (1). Under this situation, the projections from the third angle is required to increase the independent equations to completely solve all coordinates of the atoms. When the projections are digital images, the physical dimensions of the image detector, such as the pixel size, limit the accuracy of the coordinate determination of atoms, and as a result, many additional coordinates overlap with each other. In this case, more projecting angles are needed. Although more projections are needed, there is no requirement regarding the ranges of those projecting angles. In other words, the tilt angle range of ±90° is still not a necessary condition for the determination of all coordinates of the atoms of an object.

In digital images, two perpendicular tilt angles, such as ±45°, are needed for the highest-resolution reconstruction, although the tilt angle range of ±90° is not a necessary condition for the determination of all coordinates of atoms. This is because one projection provides the highest-resolution information along two directions, such as the X- and Y-direction, but no information along the third direction, such as the Z-direction. Its perpendicular projection can provide the highest-resolution information along the third direction and one of the previous two directions, such as the Z- and Y-direction or the Z- and X-direction. Unfortunately, due to the complexity of the mathematics, we cannot derive a mathematical equation to express how a projection at a projecting angle beyond ±45° can be computed from the projections within the angle range of ±45°. Alternatively, we report a method to compute the projections via restoration/correction of the 3D reconstruction from the tilt angle range beyond ±45° in the following sections.

The motivation behind the development is as follows. (i) Filling the data within the missing-wedge zone from the observed data. (ii) Reducing the missing-wedge artifact of the 3D reconstruction. (iii) Providing a low-tilt 3D reconstruction method. (iv) Increasing the SNR. The lower the total number of images used for the 3D reconstruction, the fewer tilt series of the images acquired, the higher the dose used, the higher the SNR of the images, and the greater the accuracy of the tilt series alignment, the higher the resolution of the 3D reconstruction that can be achieved. (v) Avoiding high-tilt imaging. At a high tilt angle, the ice thickness will be increased significantly, which will cause high noise in the tilt images. (vi) Simplifying image acquisition. Imaging at a high tilt angle is often hindered by mechanically unstable features, such as draft, charging and vibration from the liquid nitrogen container. (vii) Simplifying the contrast transfer function (CTF) correction. The gradient of defocus for high tilt images is often difficult to be corrected precisely due to the flatness of the EM grid. (viii) Enabling a low-polepiece TEM instrument for a tomographic study. The polepiece distance of the object lens in many high-resolution TEMs is too narrow to allow the hold to tilt above ~30°. Our LoTToR method may expend the capability of these instruments for a tomographic study.

## Algorithm

**Figure 2:**
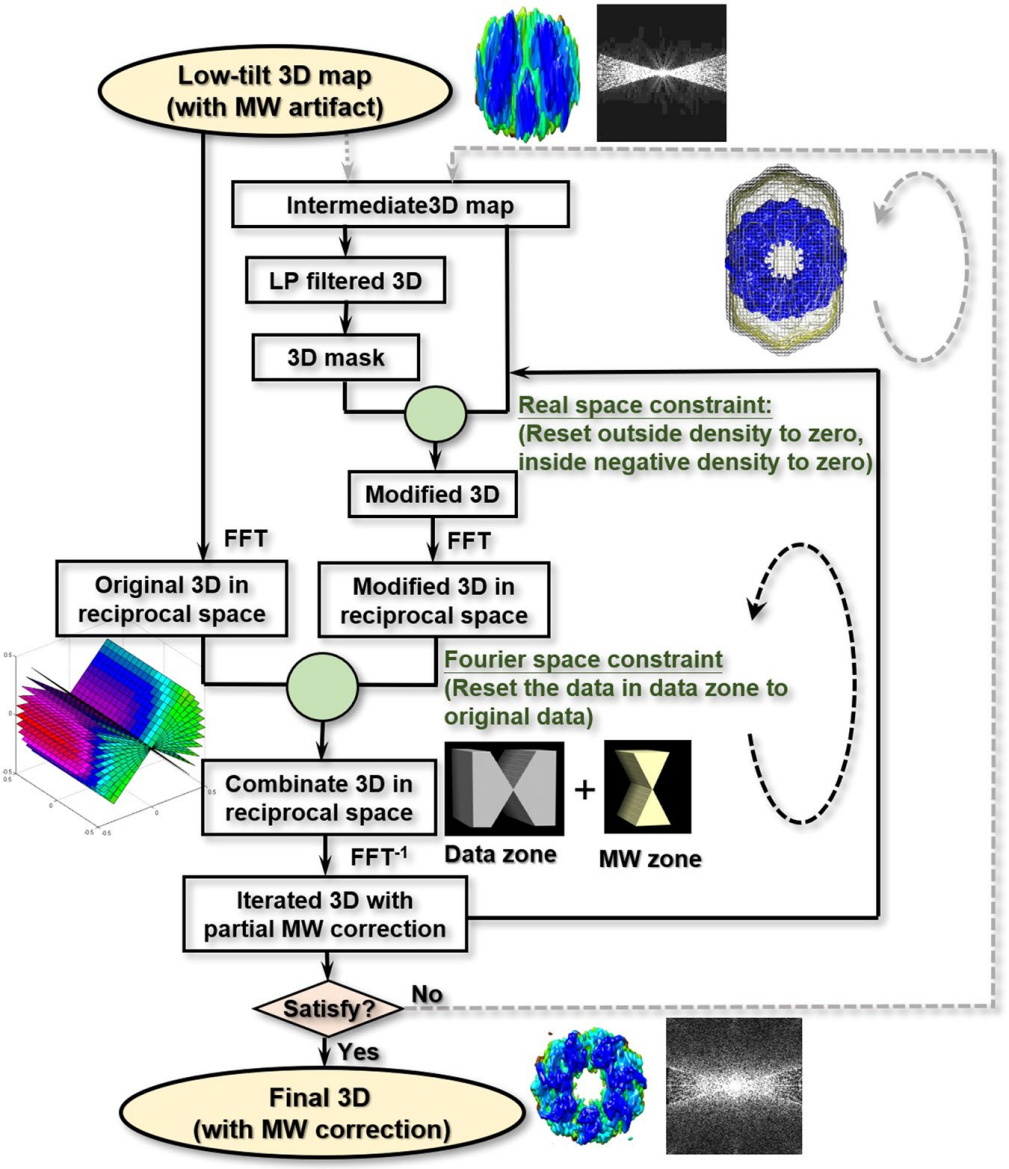
Flowchart of the missing-wedge correction algorithm. The iterative procedure includes the following steps: (i) use a reconstructed 3D density map to generate a mask after low-pass filtering; (ii) apply the mask to the 3D map to remove all densities outside the mask; (iii) reset the negative densities inside the mask to zero (remove imaginary parts if any exist); (iv) Fourier transform the masked/modified 3D map; (v) replace the data within the data zone with the original data (the tile series or initial data); (vi) inverse Fourier transform to the real space to obtain the first corrected 3D map; (vii) repeat the above steps from (ii) to (vi) for 1,000 iteration cycles (referred to as round 1 or Rd_1) to achieve a convergent 3D map. If the result is unsatisfactory, additional rounds are required, in which a new mask is generated from the output map of the last round.

Our algorithm for the restoration of missing-wedge data from the low-tilt tomography 3D reconstruction was conducted through an iterative method under a set of constraints (Fig. 2). The input data include the low-tilt 3D reconstruction (named the initial 3D map), tilt angle range, macromolecular weight, and Å/pixel of the image. The iterative processes include the following steps. (i) Low-pass filtering of the initial 3D map, *I*_0_(*x,y*), to ~40 Å−80 Å to generate a featureless and soft-boundary mask. The mask volume is defined as that corresponding to ~2–4 times the molecular weight of the protein. (ii) Applying the mask to the initial 3D map (or the last iterated 3D map, *I*_*i*−1_(*x,y*)) to mask out the densities outside the mask. (iii) Deleting negative values (caused by normalization) in the first iteration cycle. In the second and later cycles, all imaginary parts and all negative real parts of the densities are all reset to zero as follows:

2 $${I}_{i}(x,y)=\{\begin{array}{cc}0, & (x,y)\notin \{masking\,region\}\\ 0, & (x,y)\,\in \,\{masking\,region\}\,when\,Real\,[{I}_{i-1}(x,\,y)] < 0\\ Real\,[{I}_{i-1}(x,\,y)], & (x,y)\,\in \,\{{\rm{m}}{\rm{a}}{\rm{s}}{\rm{k}}{\rm{i}}{\rm{n}}{\rm{g}}\,{\rm{r}}{\rm{e}}{\rm{g}}{\rm{i}}{\rm{o}}{\rm{n}}\}\,when\,Real\,[{I}_{i-1}(x,y)]\ge 0\end{array}$$

The imaginary parts can be generated by the inverse Fourier transform of the modified data in reciprocal space in the next step. (iv) Fourier transforming of the masked 3D map, ${\mathcal F} [{I}_{i}(x,y)]$. (v) Replacing the data within the data zone (within the tilt range) by the Fourier transfer data of the initial 3D map, ${\mathcal F} [{I}_{0}(x,y)]$, as follows:

3 $${\mathcal{F}}[{I}_{i}(x,y)]=\{\begin{array}{cc}{\mathcal{F}}[{I}_{0}(x,y)] & \theta \,\in \,\{tilt\,angle\,range\}\\ {\mathcal{F}}[{I}_{i}(x,\,y)] & \theta \notin \{tilt\,angle\,range\}\end{array}$$

where θ is the tilt angle. In this case, the experimental data (within the data zone, the tilt range) are locked to those of the initial 3D map without any changes, while the data within the missing-wedge zone are treated as variables for refinement during the iteration. (vi) Inverse Fourier transforming of the combined data into a real space 3D map, *I*_*i*_(*x,y*). Since the above step of replacement can cause incoherence of the data between that within the data zone and that within the missing-wedge zone, the anti-Fourier transform of the combined data could have imaginary parts and negative values, even though they were removed in the last cycle. (vii) Deleting the imaginary portion of the data, resetting the negative real portion to zero within the mask, and resetting all intensities outside the mask to zero as shown in Eq. (2). (vii) Fourier transferring of the modified 3D map into Fourier space again to lock the experimental data within the data zone as in Eq. (3). ix) Repeating the above process 1,000 times as round 1 (Rd_1). The data within the missing-wedge zone are gradually restored. However, if the 3D map exhibits elongation phenomena, a new mask can be generated from the low-pass-filtered Rd_1 3D map since the Rd_1 3D map usually has a slightly higher resolution and lesser elongation artifact than that of the initial 3D map. The improved mask can further improve the accuracy of the restored missing-wedge data. Usually, a total of 5 rounds should be sufficent to achieve satisfactory restored missing-wedge data and corrected 3D maps based on our experience.

### Proof of concept by a phantom

**Figure 3:**
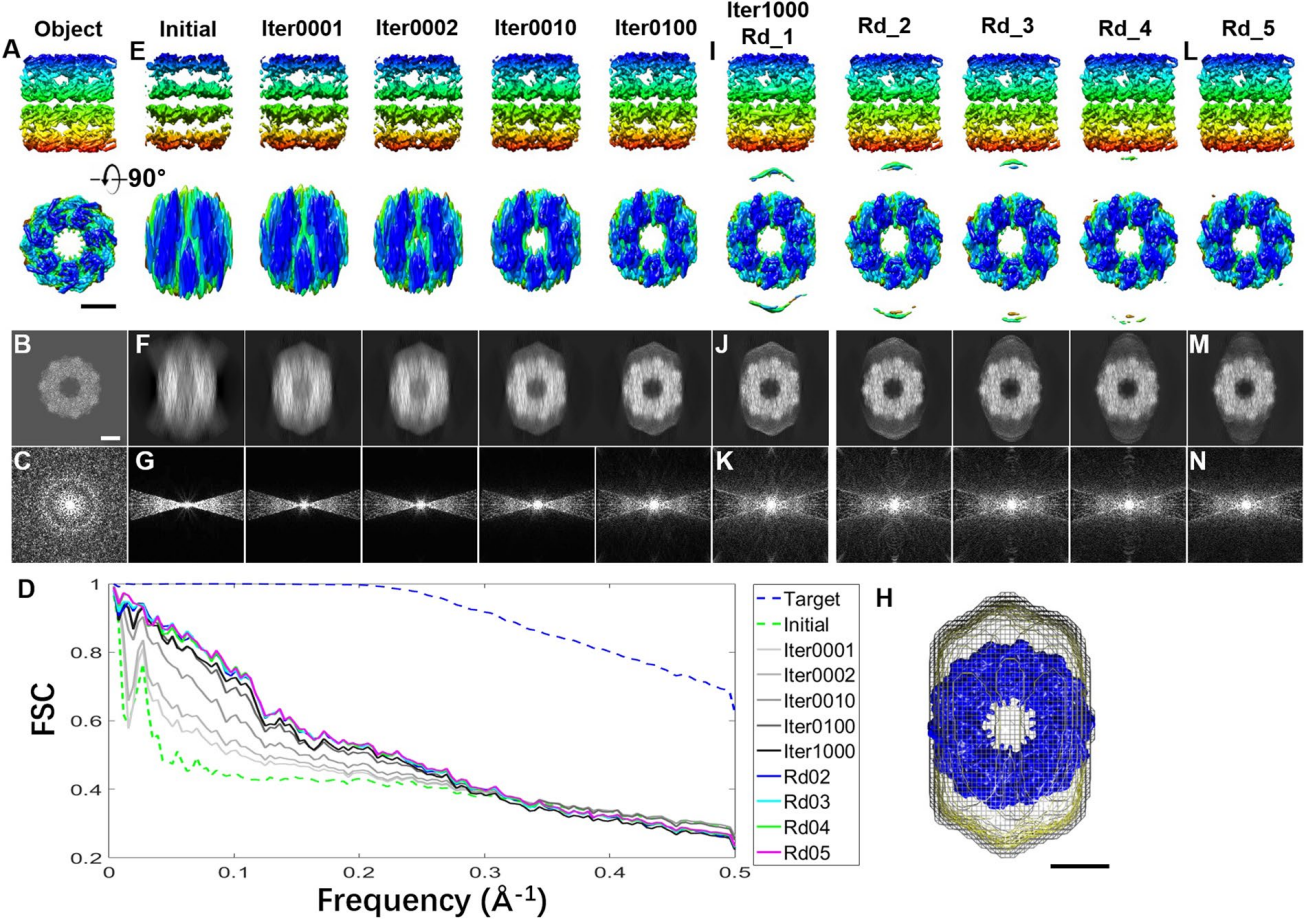
The missing-wedge correction under a large mask on a simulated 3D map reconstructed from the noise-free ± 15° tilt series of GroEL (**A**) A 3D object shown from two perpendicular views. The object was generated from the crystal structure of GroEL, and the ideal 3D map was constructed from the tilt series of the noise-free 2D projections of the object from tilt angles in a range of ±90° in steps of 1.5°, while the initial 3D map was reconstructed from angles of ±15°. (**B**) The projection of the object on the X-Z plane and (**C**) its Fourier transform. (**D**) The ideal FSC curve was computed between the object and the ideal 3D map, as shown by the blue dashed line. The other curves were computed between the object and iterative maps. (**E**) The initial 3D map and iterative 3D maps, shown from perpendicular views. (**F**,**G**) Their corresponding projections on the X-Z plane, and the corresponding Fourier transforms. (**H**) A large mask corresponding to 3.8 times the molecular weight of GroEL was generated from the low-pass-filtered initial 3D map (~40 Å, shown in the mesh; the object is shown in blue). (**I**) The final 3D maps after each round (containing 1,000 cycles of iteration) of missing-wedge correction. The maps of round 1 (Rd_1) and rounds 2–4 are shown from perpendicular views, as well as (**J**) their projections on the X-Z plane and (**K**) the Fourier transforms. (**L**–**N**) The final 3D map after round 5 is shown from perpendicular views, compared to its projection on the X-Z plane and the Fourier transform. All 3D maps underwent low-pass filtering to 8 Å. Scale bars: 50 nm.

To test whether a 3D map with a significant missing-wedge artifact can be corrected from its low-tilt series, such as within a tilt angle range of ±15° (in steps of 1.5°), we conducted the following test and compared the result to that within the range of ±90° (named the ideal map) (Fig. 3). The 3D density map of an object was created from the crystal structure of GroEL (D_7_ symmetry, molecular mass of ~800 kDa, PDB code of 1KP8^56^) (Fig. 3A–C). The tilt series were generated by projecting the 3D map from a series of tilting angles within a tilt angle range of −90° to +90° in steps of 1.5° (along the Y-axis). The 3D map reconstructed from the tilt series within the range of ±90° was used as an ideal 3D map, while the 3D map reconstructed from the range of ±15° was used as the input 3D map. To quantitively validate the corrected 3D map, the Fourier shell correlation (FSC) (Fig. 3D) and real-space cross-correlation coefficient (CCC) were employed to evaluate the similarity between the object and corrected 3D map (the detailed procedure is shown in the Methods section).

Since the initial 3D map reconstructed from the tilt series within the angle range of ±15° (Fig. 3) has more than 83% of data missing, the 3D map showed nearly no structural features with significant elongation artifacts along the Y-axis. Moreover, the intrinsic D_7_ symmetry was unrecognizable from the top and side views of the map, and additional dust appeared near the edge of the mask. The 2D projection along the Z-axis showed an obvious elongation artifact (Fig. 3F), which was confirmed by the Fourier transfer of the projection showing a horizontal bow-tie-shaped density area (named the data zone) and a vertical bow-tie-shaped black area (named the missing-wedge zone) (Fig. 3G). The data within the missing data zone are what we intend to restore from the data zone using our algorithm. The FSC curve computed between the ideal 3D map (reconstructed from that of ±90°) and the object was used as an ideal restoration curve (blue dashed line in Fig. 3D), in which the ideal 3D map resolution was beyond the Nyquist frequency, *e.g*., ~2 Å. The FSC curve computed between the initial 3D map and the object was computed as the initial curve (green dashed line in Fig. 3D), in which the resolution was ~11.6 Å and the CCC was ~0.32.

In the first iteration cycle, the initial 3D map was low-pass filtered to 40 Å to generate a mask (mash in Fig. 3H), of which the mask volume corresponded to ~3.8 times the molecular weight of GroEL. The output 3D map (labeled “Iter0001”) still presented elongation artifacts but was slightly reduced than the initial 3D map in that regard (Fig. 3E,F). The intrinsic D_7_ symmetry was still undistinguishable, and the missing-wedge zone showed no obvious filled data (Fig. 3G). However, the FSC between the Iter0001 map and the object showed an obvious improvement, especially for frequencies from ~5–20 Å (Fig. 3D). The Iter0001 map resolution was improved to ~9.5 Å from ~11.6 Å, while the CCC showed only an ~5% improvement, *i.e*., 0.36.

After 1,000 iterations (named Iter1000, round 1, or Rd_1), the features of the restored 3D map (Fig. 3I–K) showed the following. i) The top view has a round shape, except for two extra densities induced by the edge of the mask. ii) The elongation artifact was obviously corrected. iii) The intrinsic D_7_ symmetry became visible. iv) The Fourier transfer of the projection along the Z-axis showed that the original empty missing-wedge region was gradually filled with data, which generated a complete 3D map in reciprocal space. v) The 3D map with the resolution of 4 Å represents a significant improvement over the initial 3D map shown by the FSC curve but was still inferior to the ideal map (Fig. 3D), vi) The resolution was improved to ~4.7 Å, and the CCC was improved to ~0.37.

To further improve the restored 3D map, a better mask was generated from the Rd_1 map since the Rd_1 map has fewer elongation artifacts than does the initial map. The less elongation artifacts there are in the mask, the more power there is in the restorability. After the second 1,000 iterations (named Rd_2), the Rd_2 map showed further improvement, including the following: i) the FSC curve was closer to the ideal curve; ii) the resolution was ~4.29 Å, and the CCC was ~0.379; and iii) several features were visible, such as the helices in the top-right corner of the map. By further improving the restored 3D map by repeating the above round three more times, the final 3D map (named Rd_5) showed that the FSC, resolution and CCC were all further improved (Fig. 3L,N), with the resolution improved to ~4.28 Å and the CCC improved to ~0.384. Notably, the intrinsic D_7_ symmetry was much clearer than that of the initial and previous rounds of the 3D maps (Fig. 3L). Although ~83% percent of the data were missing in the initial 3D map, the final map at an ~4.3 Å resolution showed significant restoration of the overall shape and structural features (Fig. 3L,N). This indirectly proved our theory that the high-tilt projections can be derived from low-tilt data. The FSC curve and resolution of the restored map were below those of the ideal 3D map (Fig. 3D), which was due to the object containing a large number of atoms (~810 kDa) within a small box (256 × 256 × 256 voxels). By increasing the box size (pixel ratio) and decreasing the molecular weight, the restorability can be further improved.

Notably, in the above test, two parameters, *i.e*., the shape and volume of the mask, may influence the restorability. To evaluate the influence, we conducted the following two tests. In the first test, a tight mask was used (Supplementary Fig. 1), in which the volume was approximately half that of the above mask, *i.e*., ~1.75 times the molecular weight that was generated from a 60 Å filtered initial 3D map (Supplementary Fig. 1H). After round 1 (1,000 cycles of iteration), the map showed an obvious reduction in the elongation artifact. The intensities within the missing-wedge zone and the intrinsic D_7_ symmetry were both observed (Supplementary Fig. 1I–K). The resolution and CCC were also improved to ~5.7 Å and ~0.374, respectively, which were both slightly worse than those in the above test (because some portions of the object were cut off by the mask) (Supplementary Fig. 1H). The overtight mask turned the circular shape of the top view of the map into a rectangular shape (Supplementary Fig. 1I,J). Thus, we conducted four more rounds. The new tight masks (with same volume) were generated from each of the 3D maps of the previous round after low-pass filtering to 40 Å. The final 3D map (Rd_5 map) was similar to that from the above test (Supplementary Fig. 1L–N). The same resolution (~4.3 Å) and similar CCC (~0.39) suggest that the volume of the mask was not a critical parameter.

In the second test, a precise mask shape was used (Supplementary Fig. 2). The precise mask was generated from an object low-pass filtered to 40 Å, of which the volume was similar to that in the first test, *i.e*., ~3 times the protein molecular weight (Supplementary Fig. 2M). An FSC analysis of the Rd_1 curve showed an improvement in the map resolution (~4.1 Å) and CCC (~0.37). The structural features of the Rd_1 map was richer than those of above Rd_5 maps, suggesting that a precise mask can improve the efficacy of restoration.

We further asked whether increasing the percentage of data and decreasing the total number of atoms of the object can benefit the restorability. To answer this question, we conducted the following two tests. In the first test, three 3D maps with more percentage of data, such as ~33.3%, ~50% and ~66.7% of the observed data (within the data zone) were generated from the tilt series within the tilt angle ranges of ±30°, ±45° and ±60°, respectively (Supplementary Figs. 3–5). By using the precise mask discussed above, we submitted each initial 3D map for 1,000 cycles of iteration (round 1). The FSC analyses showed that the restored curves quickly approached the ideal curve, especially those within ±60° (Supplementary Fig. 5J). The resolution and CCC were also increased by increasing the percentage of data, where the resolutions were all near and beyond the Nyquist threshold, *i.e*., ~2.1 Å, ~1.8 Å and ~1.5 Å, and the CCCs were 0.53, 0.67 and 0.74, respectively (Supplementary Table 1). This test suggests that the restorability can be improved by increasing the percentage of data.

The second test was conducted by reducing the molecular weight, in which a small protein, a fragment of the molybdate transporter ModB_2_C_2_ (C_2_ symmetry, molecular mass of ~108 kDa, PDB code of 2ONK^57^), was used. The motivation behind choosing this protein as an object was that it contains several distinguishable structural features that could be used to evaluate the resolution, such as an ~100 Å Donna-like overall shape, an ~30 Å hole, 12 transmembrane α-helices, short α-helices and β-strands (Fig. 2A). Moreover, a small and low-symmetric protein is usually challenging to consider in an EM study. The object, which was within a box of 160 × 160 × 160 voxels (each voxel corresponding to 1 × 1 × 1 Å^3^ in real space, as described above) was used to generate the tilt series by being projected from −90° to +90° in steps of 1.5° (along the Y-axis). 3D maps reconstructed from the tilt series with angle ranges of ±15°, ±30°, ±45° and ±60° were used as the initial 3D maps and submitted for round 1 testing (Supplementary Figs. 6–9), in which a precise mask generated from a low-pass filtered object was used. The Rd_1 3D map showed a consistent result with that from GroEL, with the major features being restored, even for ±15° (Supplementary Fig. 6). The accuracy of the restoration was increased by increasing the percentage of data. The resolutions of the restored 3D maps of ±15°, ±30°, ±45° and ±60° were all near or even better than the Nyquist resolution, *i.e*., ~3.6 Å, ~2.0 Å, ~1.6 Å and ~1.3 Å, and the CCCs were 0.46, 0.60, 0.72 and 0.78, respectively (Supplementary Table 1), which further confirmed the above conclusion, *i.e*., increasing the percentage of data can benefit the restorability. The FSC and CCC analyses showed that the resolutions were all better than those from GroEL, evidencing that reducing the total number of atoms can improve the restorability.

All of the above tests showed that the missing-wedge data were not independent and can be derived from the low-tilt data via our restoration method. This result indirectly showed that the tilt angle range of ±90° is not necessary for a complete 3D reconstruction, although the restorability was dependent on several parameters, such as the accuracy of the mask and volume, the percentage of data within the data zone and the protein molecular weight.

### Noise influence on the capability

In real experiments, the projected image contains noise, which influences the accuracy in the determination of the atom coordinates and therefore reduces the restorability. To study how noise influences the capability of the method, three levels of noise were chosen and applied to the above tilt series at signal-to-noise ratios (SNRs) of 0.3, 0.5 and 1.0 (the SNR is defined in the Methods section). Although the SNR in cryo-EM images has a wide distribution, typically from 0.1 to 0.3^5,25,27,34,58^, considering that our purpose here was only to study the restorability of the method and its dependence on noise, Gaussian-type noise was used to roughly represent the noise in the cryo-ET images, as often used in cryo-EM methodology papers^20,25,59,60^.

**Figure 4:**
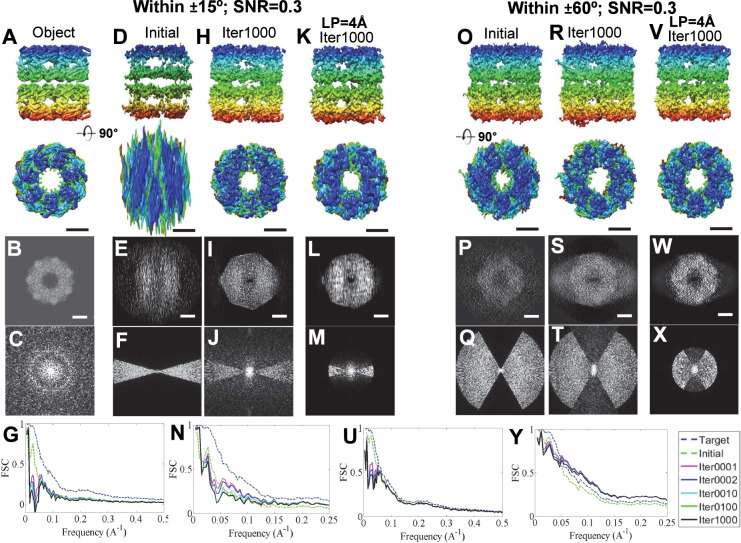
The missing-wedge correction on two simulated 3D maps of GroEL reconstructed from the noisy tilt series (SNR = 0.3) within the tilt angle range of ± 15° and ± 60°. (**A**) Two perpendicular views of the object of GroEL, (**B** its projection on the X-Z plane and (**C**) the corresponding Fourier transform. (**D**) The initial 3D map, shown from two perpendicular views. The initial 3D map was constructed from a noisy tilt series (SNR = 0.3) in a range of ±15° in steps of 1.5°, while the ideal 3D map was constructed from that of ±90°. (**E**) The projection of the initial 3D map on the X-Z plane and (**F**) its Fourier transform. (**G**) The FSC curves of the initial, ideal and iterative 3D maps against the object. The iterative 3D maps were generated after 1, 2, 10, and 100 cycles of interaction of missing-wedge correction. (**H**) The final restored 3D map (after 1,000 iterations of missing-wedge correction), shown from two perpendicular views, (**I**) the corresponding 2D projection (along the X-Z plane) and (**J**) the Fourier transform. (**K**) To reduce the influence of noise, the noisy tilt series (SNR = 0.3) were filtered to 4 Å before generating an initial 3D map for restoration. The final restored 3D map after 1,000 iterations is shown from two perpendicular views, as well as the (**L**) 2D projection (along the X-Z plane) and (**M**) Fourier transform. (**N**) The FSC curves of the initial, ideal and iterative 3D maps against the object. (**O-Y**) The procedures were repeated by using the above tilt series for the angle range of ±60°. All 3D maps were low-pass filtered to 8 Å. Scale bars: 50 nm.

The 3D reconstruction of GroEL from the tilt range of ±15° under an SNR = 0.3 was used as the initial 3D map for the round 1 test (Fig. 4A–J and Supplementary Fig. 10). As a comparison, the FSC between the ideal 3D map at an SNR of 0.3 and the object was also computed (blue line in Fig. 4J). The FSC showed that the ideal curve was decreased significantly by the noise, in which the resolution of the ideal 3D map (ideal resolution) was ~15.3 Å, which was close to the resolution of the initial 3D map (initial resolution), *i.e*., ~28.9 Å. After round 1, the FSC showed that the restored curve failed to improve, especially for the low-resolution portion (below ~20 Å), which was even worse than that of the initial portion (Fig. 4J and Supplementary Fig. 10J). The resolution was reduced to ~73.8 Å (Supplementary Table 1), which suggested that the noise hindered the restorability. The poor improvement in restoration may be due to the overweighting of the noise portion compared to the signal from the protein in the determination of the variables within the missing-wedge zone. We would like to check whether reducing the volume of the mask and increasing the percentage of the data can reduce the weight of the noise and whether increasing the protein signals can benefit the restoration. We found, by reducing the mask volume to half, *i.e*., ~1.5 times the molecular weight, that the resolution improved to ~21.1 Å, suggesting that the volume of the mask has a certain power to reduce the noise influence, especially for a low frequency. To test how increasing the percentage of data influences the result, 3D maps reconstructed from tilt angle ranges of ±30°, ±45° and ±60° under an SNR = 0.3 were used as the initial models for the round 1 test (Fig. 4O–U and Supplementary Figs. 11–13). The FSC showed that the highest resolutions were improved up to ~16.9 Å, which is better before and close to the initial resolution (~18.6 Å) but still much worse than the ideal resolution (~15.3 Å) (Supplementary Table 2). Although all restored curves were similar to the ideal curve at resolutions beyond ~20 Å, the unexpectedly poor curve at frequencies below ~20 Å implies that the weight of noise was still strong and interrupted the determination of the missing-wedge data. The above tests suggest that noise plays a key role in reducing restorability by reducing the weight of the data contribution. Both tighter masks and more tilt data have limited power to benefit the restoration.

To confirm the above result that the noise plays a key role in reducing the restorability, we repeated the above tests by reducing the noise level, *i.e*., using the initial 3D map reconstructed from the tilt series with a higher SNR (=0.5). The FSC curves of the restored 3D maps from the tilt series of ±15°, ±30°, ±45° and ±60° still had some sharp declines in their low-resolution portion (Supplementary Figs. 14–17). Although the restored resolutions are a certain improvement over that for an SNR = 0.3, the resolutions were still generally worse than the initial resolutions (~36.0 Å vs. ~25.3 Å, ~15.9 Å vs. ~18.6 Å, ~13.4 Å vs. ~15.4 Å and ~11.0 Å vs. ~11.6 Å for restoration vs. initial of ±15°, ±30°, ±45° and ±60°, respectively) and the ideal resolution (~9.8 Å) (Supplementary Table 1). The highest resolutions were improved up to ~11.0 Å, which is slightly better than the initial resolution (~11.6 Å) and very close to the ideal resolution (~9.8 Å), suggesting that the lower the noise is, the greater the power to achieve restorability.

To further confirm the above result, initial 3D maps reconstructed from a high SNR (=1.0) for all tilt series were used. The FSC analysis showed that the restored curves of ±15°, ±30°, ±45° and ±60° presented significant improvement compared to the initial ones (Supplementary Figs. 18–21). The low-resolution portions were also improved, while the restored resolutions were all better than those of the initial ones, *i.e*., ~13.4 Å vs. ~24.2 Å, ~8.7 Å vs. ~11.5 Å, ~7.8 Å vs. ~8.8 Å and ~6.9 Å vs. ~8.1 Å (restoration vs. initial) for the ±15°, ±30°, ±45° and ±60° tilt ranges, respectively (Supplementary Table 2). Although the best resolution (~6.9) was still lower than the ideal resolution (~4.5 Å), the continuous improvement in the FSC curve and resolution confirmed that noise played a key role in hindering the restorability and that lower noise could benefit the restorability.

From the aspect of the molecular weight influence, we repeated the above tests by reducing the molecular weight, *i.e*., using a small protein, the ModB_2_C_2_ 3D maps were reconstructed from tilt series of ±15°, ±30°, ±45° and ±60° under SNRs of 0.3, 0.5 and 1.0, respectively (Supplementary Figs. 22–33). A total of 12 initial 3D maps were reconstructed. The FSC analyses of the restored 3D maps under SNR = 0.3 showed that the curves at low resolution also declined sharply, in which the restored resolutions were significantly worse than the initial resolutions, *i.e*., ~51.3 Å vs. ~28.8 Å; ~47.1 Å vs. ~23.2 Å; ~14.2 Å vs. ~14.7 Å; and ~11.3 Å vs. ~13.7 Å (restoration vs. initial) for ranges of ±15°, ±30°, ±45° and ±60°, respectively (Supplementary Figs. 22–25, Supplementary Table 3), and were all significantly lower than the ideal resolution, *i.e*., 9.4 Å. The phenomenon was consistent with that when using a large protein, *i.e*., GroEL. By increasing the SNR to 0.5, the FSC analyses showed that the restored resolutions were significantly improved compared with those for an SNR = 0.3. The resolutions were similar to or even higher than the initial resolutions, *i.e*., ~43.9 Å vs. ~27.5 Å, ~10.7 Å vs. ~14.9 Å, ~8.2 Å vs. ~11.0 Å and ~7.6 Å vs. ~10.1 Å (restoration vs. initial) for ±15°, ±30°, ±45° and ±60°, respectively (Supplementary Figs. 26–29 and Supplementary Table 3). The restored curves showed better resolutions for ±45° and ±60°, which were closer to the ideal resolution, *i.e*., ~7.4 Å. By further increasing the SNR to 1.0, the resolutions were improved to ~9.4 Å, ~4.2 Å, ~3.7 Å and ~3.5 Å, respectively (Supplementary Figs. 30–33, Supplementary Table 3), in which the restored resolutions for ±30°, ±45° and ±60° were all similar to the ideal resolution, *i.e*., 3.5 Å. The above tests confirmed again that noise played a key role in limiting the restorability, but a smaller protein has a better resistance to the noise.

The above characterization of our method can be summarized as follows. (i) The tilt series beyond ±45° is not completely independent from that within ±45° and can be nearly restored. (ii) The restorability can be increased by decreasing the molecular weight. (iii) The restorability can be increased by increasing the percentage of data (the range of the data zone). (iv) The restorability can be increased by increasing the accuracy of the mask but is not sensitive to the volume of the mask. (v) The noise plays a key role in reducing the reconstruction resolution and limits the power of the method. (vi) A smaller protein has a better resistance to the interruption from noise.

### Low-pass filter to reduce the influence from noise

To reduce the influence of noise on the restorability, we proposed using a low-pass filter to reduce the noise in the tilt series first before 3D reconstruction. Usually, the tilt series were low-pass filtered to 4 Å, which is beyond our desired resolution (such as ~8 Å, a resolution that can show α helices). The tilt series of GroEL under SNRs of 0.3, 0.5 and 1.0 were all filtered before 3D reconstruction for restoration (Supplementary Figs. 34–46). Notably, the ideal 3D maps were not filtered. The restored curves of ±15° showed a certain improvement compared to those without filtering (Fig. 4K–N and Supplementary Figs. 34–36), especially for resolutions between ~5 Å and ~20 Å. The restored 3D map resolutions were all better than those of the nonfiltered one and were comparable to the initial values, *i.e*., ~34.8 Å vs. ~28.9 Å, ~30.9 Å vs. ~25.3 Å, and ~19.5 Å vs. ~24.2 Å (restored vs initial) at SNRs of 0.3, 0.5 and 1.0, respectively (Supplemental Table 2). Similarly, by increasing the percentage of data (angle ranges), the restored curves from ±30° showed even better results, in which the restored structures have a higher similarity to the object than to the initial structures within the resolution between ~5 and ~20 Å (Supplementary Figs. 37–39). The restored resolutions were ~33.2, ~14.5 and ~8.9 Å, respectively (Supplementary Table 2). When the percentage of data was increased to that of ±45° and ±60°, the restored curves showed a near overlap relative to the ideal curves, especially within the resolution between ~5 Å and ~20 Å (Fig. 4V–Y and Supplementary Figs. 40–45). The restored resolutions were nearly all similar or even better than the ideal resolutions at the corresponding SNRs, *i.e*., ~16.4 Å vs. ~21.6 Å, ~9.8 Å vs. ~15.4 Å, and ~4.3 Å vs. ~8.8 Å (restored vs. ideal for ±45°) and ~13.1 Å vs. ~18.6 Å, ~8.4 Å vs. ~11.6 Å and ~4.0 Å vs. ~8.1 Å (restored vs. ideal for ±60°) (Supplementary Table 2), which are even better than the ideal resolution (since the ideal map contains noise). The above tests suggest that by reducing the noise, the missing-wedge restorability can be improved.

We want to test whether the power of the restoration can be improved by reducing the molecular weight, as shown in the noise-free tilt series. To test this hypothesis, a smaller protein, *i.e*., ModB_2_C_2_, was used to repeat the above test (Supplementary Figs. 46–57). The tilt series under SNRs of 0.3, 0.5 and 1.0 were low-pass filtered to 4 Å before being submitted to the test. The ideal 3D maps were not filtered. The restored FSC curves from ±15° showed an obvious improvement over those without filtering (Supplementary Figs. 46–48). Other than the poor low-resolution portion (beyond ~20 Å), all curves were better than the initial ones (Supplementary Figs. 46–48). The resolutions were all better than those of the initial curves, *i.e*., ~28.5 Å vs. ~28.8 Å, ~15.6 Å vs. ~27.5 Å, and ~11.6 Å vs. ~26.8 Å (restored vs initial) at SNRs of 0.3, 0.5 and 1.0, respectively (Supplementary Table 3). By increasing the percentage of the data to that from ±30°, the capability was further increased (Supplementary Figs. 49–51). By further increasing the percentage of data to that from ±45° and ±60°, the capability was increased to values nearly better than the ideal resolutions (Supplementary Figs. 52–57, and Supplementary Table 3). The above tests further confirmed that by reducing the noise, the capability of the method can be restored, and a smaller protein can be more resistant to noise.

Although the noise levels may not precisely represent the real noise in the experiments, which also depend on the exposure time, the method used to compute the SNR, and the chemical components, such as the water and solvent, the purpose of using above noise levels was to characterize the algorithm and build up our experience before performing the real experiment. In the following sections, we validate the method on real experimental data with real noise.

### Tests on real negative-staining (NS) ET data

To test the capability on real experimental data, three types of NS samples were used, *i.e*., 53 kDa cholesteryl ester transfer protein (CETP), 84-base-pair double-stranded DNA (dsDNA; the molecular mass is ~52 kDa) conjugated to 5 nm nanogold, and a nucleosome (assembled by the histone protein, the molecular mass of which is ~11 kDa, and dsDNA, the molecular mass of which is ~400 kDa). The CETP sample was prepared by the optimized NS (OpNS) protocol^19,21,22^, and the tilt series were acquired within a tilt angle range from −45° to +45° in steps of 1.5° under a magnification of 67 k× (corresponding to ~1.73 Å/pixel). The survey views at three representative tilt angles showed rod-shaped CETP particles (Supplementary Fig. 58). After CTF correction of the tilt series by the TomoCTF software package^61^, one CETP particle was tracked and boxed out from the tilt series. The tilt series of the boxed CETP particle were low-pass filtered at 10 Å and then submitted to an additional process, *i.e*., contrast enhancement processing^62^, to further increase the SNR and reduce the noise before iteratively aligning the tilt series to a global center by the IPET program^5^ (Supplementary Fig. S57B–E). Our previously reported enhancement algorithm can increase the SNR of the images and 3D reconstruction on both synthetic data and real experimental data but does not obviously decrease the resolution^62^.

**Figure 5:**
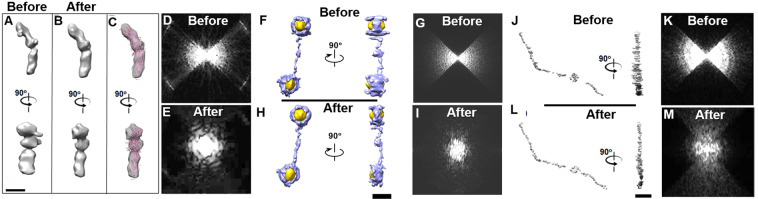
Missing-wedge correction of the NS single-molecule 3D reconstructions. (**A**) Two perpendicular views of a single-molecule 3D density map (low-pass filtered at 8 Å) of an individual CETP particle reconstructed by IPET on the NS tilt series (a portion of tilt series from −45° to +45° in steps of 1.5° was used for 3D reconstruction) after CTF correction and contrast enhancement^25^. (**B**) The corresponding views of the 3D map after the 1,000 iterations of missing-wedge correction. (**C**) The corrected 3D density map rigid-body-docked with the crystal structure of CETP. (**D**,**E**) Fourier transforms of X-Z projections of the initial and final 3D maps. (**F**–**I**) The missing-wedge correction on a 3D map of an individual complex of DNA-nanogold conjugate imaged by NS ET and reconstructed by IPET from a portion of the tilt series (from −45° to +45° in steps of 1.5°). Two perpendicular views and Fourier transforms of the X-Z projections of the 3D maps before and after correction are shown. (**J**–**M**) The missing-wedge correction on a 3D map of an individual nucleosome particle (DNA-histone complex) imaged by NS ET and reconstructed by IPET from a portion of the tilt series (from  −45° to +45° in steps of 1.5°). Two perpendicular views and Fourier transforms of X-Z projections of the 3D maps before and after correction are shown. Scale bars: 5 nm in A and 20 nm in H and L.

The final IPET 3D reconstruction at a resolution of ~15 Å (Supplementary Fig. 58F) showed a curved rod shape with a length of ~130.0 Å (low-pass filtered to 8 Å, displayed from two perpendicular viewing directions in Fig. 5A and Supplementary Fig. 58C). Considering that the ~15 Å resolution could be underestimated by using two half-data reconstructions for evaluation based on the FSC curved at a frequency of 0.5 as described^5^, the underestimation could be partially compensated for by using a slightly higher-resolution filter, such as 8 Å. The 3D map showed significant elongation in the Z-direction as a missing-wedge artifact (Fig. 5A), in which the length along the Z-direction was nearly doubled compared to that along the perpendicular direction due to nearly 50% of the data being missing (Fig. 5D and the first column in Supplementary Fig. 58C, and G). After the restoration, the data within the missing-wedge zone gradually appeared (Fig. 5B and Supplementary Fig. 58G). The restored 3D map showed fewer elongation artefacts than before. The uniform rod shape with cross-sectional dimensions of ~32 Å × ~35 Å (Fig. 5C and Supplementary Fig. 58E) was similar to that of the CETP crystal structure^63^, suggesting that the missing-wedge-caused elongation artifact along the Z-direction was corrected. This is because the Fourier transform of the X-Z plane projection showed that the original empty data in the missing wedge were gradually filled with data, which benefits the complete 3D reconstruction (Fig. 5D, and E and Supplementary Fig. 58G).

To confirm the above result, another CETP IPET 3D reconstruction at an ~14 Å resolution (Supplementary Fig. 58L) was submitted for restoration. The initial 3D map showed an oblate rod shape with a length of ~120 Å and a cross section of ~16 Å × ~44 Å (Supplementary Fig. 58I). The cross section presented obvious elongation as a missing-wedge artifact along the Z-direction. After the correction, the elongation phenomenon was significantly reduced (Supplementary Fig. S57J), and the dimensions of the particle cross section were corrected to ~21 Å × ~30 Å, which were similar to those in the crystal structure (Supplementary Fig. S57K). The Fourier transform of the X-Z plane projection confirmed that the missing information was filled, which could benefit a complete 3D reconstruction, with the missing-wedge-caused elongation artifact being limited (Fig. 4D,E and Supplementary Fig. S57M).

The second NS sample used for the validation was 84-base pair dsDNA conjugated to two 5 nm nanogolds^8^ that were also prepared by the OpNS protocol^19,22^. The tilt series were imaged under a magnification of 125 k× (corresponding to 0.94 Å/pixel)^8^. Three representative survey views showed conjugates formed by the fiber-shaped dsDNA, of which the distal ends bound the two nanogolds (Supplementary Fig. 59A). After CTF correction, the 3D map of an individual conjugate was reconstructed from the tilt series within -45° to +45° (in steps of 1.5°) by IPET (Supplementary Fig. 59B,C). The 3D map at a resolution of ~15 Å (Supplementary Fig. 59E) showed fiber-shaped DNA (with a length of ~210 Å after low-pass filtering to 15 Å) connected with two irregular circular-shaped densities (Fig. 5F and Supplementary Fig. 59C). The irregular densities were represented by the coating with thiolated short chain polyethylene glycol (PEG) molecules on the nanogolds to stabilize the particle against aggregation at high ionic strength. To display the architecture between the nanogold particles and DNA strands, the contrast-reversed 3D map (highlighted in gold) was overlaid on top of the original 3D map (Fig. 5F and Supplementary Fig. 59C). Along the Z-direction, the nanogold showed a significant elongation, and the length along the Z-direction was ~89–92 Å, obviously longer than that in the X-Y plane (~60 Å and ~63 Å). Meanwhile, the DNA was flattened and adhered to the carbon film with dimensions of ~15 Å × ~24 Å × ~106 Å (Supplementary Fig. 59C,D). After the correction, the elongation on the two nanogold particles was reduced, and the nanogold particles became roughly spherical, with diameters along the Z-direction reduced to ~74 Å and ~70 Å. In the meantime, the length of the DNA measured along the X-Y plane (adhered C-film) remained the same, and the width on the X-Y plane was also the same. However, the width along the Z-direction was reduced to ~16 Å from the original 24 Å (Fig. 5H and Supplementary Fig. 59D), which was toward the DNA dimensions^64^. The Fourier transform of the projection on the X-Z plane showed that the missing-wedge information was gradually filled in for a complete 3D reconstruction as the missing wedge artifact was reduced (Fig. 5G,I, and Supplementary Fig. 59F).

The IPET 3D map of another dsDNA-nanogold conjugate at a resolution of ~13 Å was also submitted for correction (Supplementary Fig. 59G–K). Before the correction, the elongation artifact was visible, including ellipsoid-shaped nanogold with dimensions of ~64 Å × ~64 Å × ~91 Å and ~54 Å × ~59 Å × ~83 Å and flattened DNA along the Z-direction with dimensions of ~13 Å × ~24 Å × ~106 Å (Supplementary Fig. 59H,I). After the correction, the artifacts were nearly invisible since the dimensions of the nanogold particles became ~82 Å and ~68 Å, while the dimensions of the DNA became ~16 Å × ~17 Å, similar to the DNA dimensions^64^. The Fourier transforms of the projection on the X-Z plane showed the progress of the missing-wedge correction (Supplementary Fig. 59K).

The third NS sample for the validation was nucleosomes (a histone octamer bound with ~600 base pairs of DNA) that was imaged by ET under a magnification of 80 k× (corresponding to 1.48 Å/pixel)^24^. Three representative survey views of the first nucleosome particle showed long fiber-shaped dsDNA combined with a disc-shaped histone octamer (Supplementary Fig. 60A). The CTF-corrected tilt series within the tilt angle range of ±45° (in steps of 1.5°) were submitted for image contrast enhancement^25^ before IPET 3D reconstruction (Supplementary Fig. 60B,C). The 3D reconstruction at an ~25 Å resolution (Supplementary Fig. 60E) showed a fiber with a total length of ~1,613 Å (corresponding to ~474 base pairs composited with two sections in lengths of ~1,095 Å and ~518 Å) wrapped around a disc. Although the DNA portion contained the Z-direction elongation/stretching artifacts, the elongation/stretching artifacts present along the Z-direction will not influence the length measured in the X- and Y-direction. Considering that all the DNA adhered to the supporting carbon film, *e.g*., the X- and Y-direction, the measured length on the X-Y plane was still reliable for computing the number of DNA base pairs. The disc dimensions were ~105 Å × ~109 Å × ~101 Å, similar to those of the histone crystal structure. Based on the crystal structure^65^, the DNA wrapped around the histone octamer with 1.65 turns and 147 base pairs, and the total length of the DNA was 621 base pairs (based on 3.4 Å/base pair). The ~74 base-pair difference with respect to the designed length, *i.e*., 547 base pairs, may be due to the outer turn of the wrapped DNA being unwrapped, as reported by the magnetic twister experiment^66^. Similar to the above artifact in the DNA portion, the width of the dsDNA along the Z-direction was three times that along the perpendicular directions (the cross section was ~20 Å × ~67 Å) (Fig. 5J, and Supplementary Fig. 60C). After correction, the elongated dimension of the DNA portion was decreased to ~19.1 Å (Fig. 5L, and Supplementary Fig. 60D), which can be evidenced by the Fourier transforms of the X-Z plane projections (Fig. 5K,M, and Supplementary Fig. 60F).

Through a similar procedure, the IPET 3D density map of second nucleosome particle was corrected (Supplementary Fig. 60G–K). This IPET 3D map, with an ~27 Å resolution, showed a flexible fiber (in a cross section of ~18 Å × ~48 Å) twist around a disc, with dimensions of ~97 Å × ~118 Å × ~114 Å. Two sections  of the fibers had lengths of ~1114.0 Å and ~258.0 Å, which corresponded to a total of 404 base pairs (based on 3.4 Å/base pair). According to the crystal structure^65^, 147 base pairs of DNA were wrapped around the histone octamer, and the total length of the DNA was 551 base pairs, approximately 4 base pairs longer than the designed length, *i.e*., 547 base pairs, which may be due to the slight dynamics of the wrapped DNA compared to the crystal structure. After correction, the cross section of the fiber changed to ~20 Å × ~23 Å (Supplementary Fig. 60I), while the missing-wedge information was restored gradually, as shown by the Fourier transform of the X-Z plane projection (Supplementary Fig. 60K), suggesting that the elongation artifacts along the Z-direction were reduced.

Based on all the above tests from the three NS samples, the missing-wedge artifacts were all successfully reduced in their restored 3D maps, suggesting that our method is a practical approach for processing real NS experimental data.

### Test on real cryo-ET data

One of the most important motivations of our method was to correct the missing-wedge artifact in the cryo-ET 3D reconstruction. Due to the radiation damage and tilt limitation, 3D reconstructions often contain missing-wedge artifacts, especially those from a single-tilt axis. To test the capability of cryo-EM 3D reconstruction, three types of samples imaged by cryo-ET and reconstructed by IPET were used, *i.e*., the lipid vesicles (phosphatidylcholine, POPC, only liposome), the LDL, molecular mass: ~2500 kDa^67^ and 53 kDa CETP^35^.

**Figure 6:**
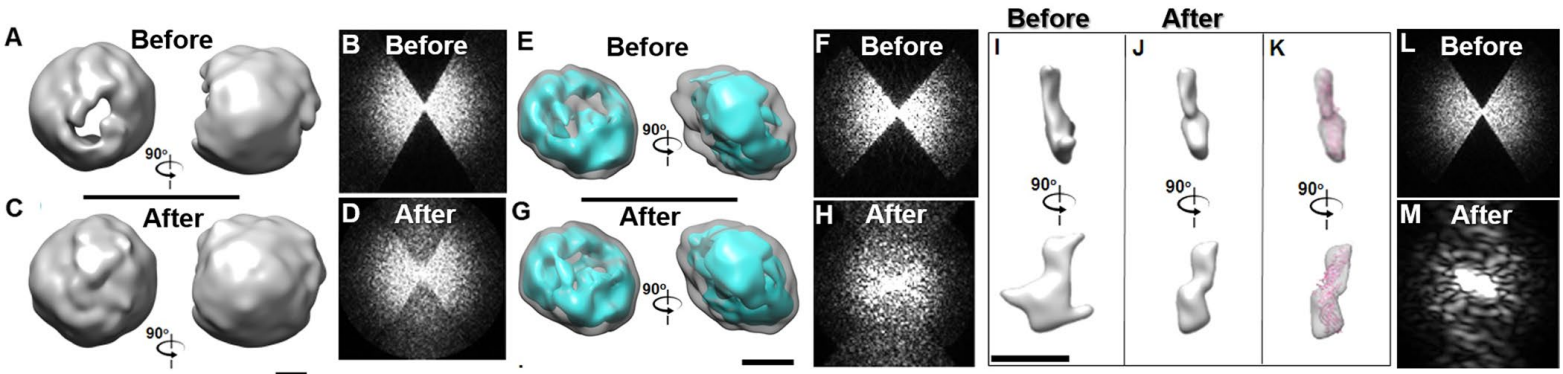
Missing-wedge correction of the cryo-EM single-molecule 3D reconstructions. (**A**) Two perpendicular views of a single-molecule 3D density map of an individual liposome particle reconstructed by IPET on the cryo-EM tilt series (from −57° to +60° in steps of 1.5°) after CTF correction and contrast enhancement^25^. The 3D map was low-pass filtered at 70 Å. (**B**) Fourier transform of the X-Z projection of the 3D map. (**C**) The corresponding views of the 3D map after the 1,000 iterations of missing-wedge correction. (**D**) Fourier transform of the X-Z projection of the corrected 3D map. (**E**–**H**) The missing-wedge correction on a 3D map of an individual LDL particle imaged by cryo-ET and reconstructed by IPET from the tilt series (from −45° to +45° in steps of 1.5°). Two Fourier transforms of X-Z projections of the 3D maps before and after correction are shown. (**I**–**M**) The missing-wedge correction on a 3D map of an individual CETP particle (53 kDa) imaged by cryo-ET and reconstructed by IPET from the tilt series (from −49° to +65° in steps of 1.5°). Two perpendicular views and Fourier transforms of X-Z projections of the 3D maps before and after correction are shown. Scale bars: 50 nm in C and G and 10 nm in I.

The samples of POPC liposome vesicles were imaged by cryo-ET under low-dose conditions (a total dose of ~120 e^-^/Å^2^) with a magnification of 50 k× (corresponding to 2.4 Å/pixel) and tilt series of angles in a range of -57° to +60° in steps of 1.5°. Three survey tilted images displayed liposome particles with a roughly hollow circular shape embedded in a high noise background of vitreous ice (Supplementary Fig. 61A). A 3D map of one representative liposome vesicle was reconstructed by IPET after CTF correction, low-pass filtering at 10 Å (which was sufficient to reduce the white noise in the ET images) and contrast enhancement (Supplementary Fig. 61B). The 3D map at a low contour level displayed an ellipsoid with dimensions of ~424 Å × ~413 Å × ~440 Å, rather than a spherical shell structure (the 3D map was low-pass filtered to 70 Å based on the resolution of 71 Å, Supplementary Fig. 61E). The resolution may be underestimated as described^5^ because the FSC curve is computed from two half-data reconstructions. At a high contour level, the 3D reconstruction displayed two holes on each side of the shell along the Z-direction (Fig. 6A and Supplementary Fig. 61C). The ellipsoid shape and holes were well recognized as artifacts of the missing wedge. After the correction (Fig. 6 and Supplementary Fig. 61D), the diameter of the 3D map at the low contour level decreased to ~420 Å along its Z-direction, which resulted in an overall spherical shape. At the high contour level, the holes disappeared on the surface. The unified shell density of the lipid bilayer of the liposome after correction suggests that the missing-wedge-caused artifact was reduced (Fig. 6C). The Fourier transform of the projection on the X-Z plane showed that the original empty data within the missing-wedge zone was gradually filled with data, which benefited artifact correction (Fig. 6D and Supplementary Fig. 61F).

The second cryo-EM sample was human low-density lipoprotein (LDL). The tilt series of the images were acquired within the tilting angle range from −57° to +52.5° at 1.5° increments under low-dose conditions (a total dose of ~360 e^-^/Å^2^) and a magnification of 80 k× (corresponding to 1.48 Å/pixel). The survey cryo-ET micrographs showed ellipsoid-shaped LDL particles in vitreous ice (Supplementary Fig. 62A). After CTF correction, low-pass filtering at 10 Å and contrast enhancement, the tilt images of a targeted LDL particle were iteratively aligned to a global center by IPET (Supplementary Fig. 62B). The 3D reconstruction (low-pass filtering to 50 Å) at a resolution of ~68 Å (Supplementary Fig. 62E) showed an ellipsoid-shaped particle with dimensions of ~217.0 Å × ~233.0 Å × ~135.0 Å (Supplementary Fig. 62C). Although the resolution was low, the 3D map obtained from an individual LDL particle can provide a base for future statistical analysis of the LDL 3D map size variety in solution, which could not be obtained from any averaging methods. At a high contour level, two holes were observed on the top and bottom sides of the particle along the Z-direction (Fig. 6E and Supplementary Fig. 62C). After missing-wedge correction, although the particle overall dimensions underwent no obvious change, the holes were partially filled, and the high-density portion (cyan) became more connected than it was before correction (Fig. 6G, and Supplementary Fig. 62D). The second LDL particle, imaged at a higher magnification, 125 k× (corresponding to 0.94 Å/pixel), under a lower total dose of ~250 e^-^/Å^2^ than that of the first LDL particle, was reconstructed by IPET (Supplementary Fig. 62G). The 3D map at a resolution of ~55 Å (Supplementary Fig. 62J) also showed an ellipsoidal-shaped particle with dimensions of ~203.0 Å × ~219.0 Å × ~124.0. Two holes at a high contour level (cyan in Fig. 6H and Supplementary Fig. 62H) were visible. After the correction, the holes became partially filled, as for the first one (Fig. 6I and Supplementary Fig. 62I). The Fourier transforms of the X-Z plane projections of both particles showed that the missing-wedge information was gradually restored (Fig. 6F,H, and Supplementary Fig. 62F,K). As a result, a complete 3D reconstruction could be obtained from it as part of the missing-wedge correction of the artifact.

To challenge the limitation, a small protein, *i.e*., 53 kDa CETP, was also used. The tilt series of the images of CETP particles in the mixture sample of LDL and CETP were acquired from a tilt angle range from -49° to +65° at 1.5° increments under a low-dose condition (a total dose of ~100 e^-^/Å^2^) and a magnification of 50 k× (corresponding to 4.8 Å/pixel), as reported^24,25^. The survey cryo-ET micrographs showed discoidal-shaped LDL and rod-shaped CETP particles in vitreous ice (Supplementary Fig. 63A). Considering that the IPET 3D reconstructions of LDL alone (Fig. 6E,G) and LDL bound to CETP have been published^25^, we instead focused on the IPET 3D reconstruction of CETP alone. The tilt images for a targeted CETP particle were iteratively aligned to a global center by IPET after the images were CTF-corrected, low-pass filtered to 12 Å and contrast-enhanced (Supplementary Fig. 63B). The IPET 3D reconstruction at ~61 Å (Fig. 6I, and Supplementary Fig. 63C,F) showed a rod-shaped CETP particle with dimensions of ~27 Å × ~55 Å × ~126 Å. Notably, the protrusions at one end were likely missing-wedge artifacts. After the correction, the elongation and protrusion phenomena were both reduced, leading to a more uniform rod-shaped CETP with dimensions of ~27 Å × ~35 Å × ~126 Å (Fig. 6J, and Supplementary Fig. 63D map), similar to the dimensions and shape of the crystal structure (PDB entry: 2OBD^68^, Fig. 6K and Supplementary Fig. 63E). The Fourier transforms of the X-Z plane projection showed the progress of the missing-wedge correction (Fig. 6L,M and Supplementary Fig. 63G). All of the above tests suggest that the algorithm is a useful method for reducing the missing-wedge artifact of the cryo-ET 3D reconstruction.

## Discussion

Our LoTToR method for missing-wedge correction benefited from the following constraints: (i) the densities outside the mask were zero, a strategy that is similar to the solvent flattening method in X-ray crystallography;^69^ (ii) the experimental/observed data were unchanged during the refinement of the missing-wedge data, an approach that is similar to the phase extension method in X-ray crystallography;^70^ (iii) non-negative density existed within the mask, and non-imaginary portion of the densities. The restorability depends on the following parameters, *i.e*., the accuracy of the mask, volume of the mask, molecular weight of the object, percentage of the experimental/observation data against missing data, and noise level. The higher the noise was, the lower the restorability.

From the viewpoint of real space, our strategy of refining the missing data from the experimental/observed data under the constraints of the data region and observations has certain similarity to the solvent flattening method in X-ray crystallography^69^. Solvent flattening is a method to improve the crystallographic phases of macromolecular structures to obtain a high-resolution structure via an iterative method for maximization of a likelihood function consisting of both an experimental phase and a density map within a region. In this method, the crystal phases and diffraction/amplitude at certain lattice spots gradually extend from the low-resolution experimental phase. However, in our method, both the phases and amplitudes at each voxel are computed or refined. Much more data have been refined without supported from the high SNR signals of the crystal. From the viewpoint of reciprocal space, our method has certain similarity to the procedure for phase extension in crystallography^70^. In phase extension, the high-resolution phases gradually extend from the low-resolution experimental phase under the constraint of observed high-resolution diffraction. In our method, both high- and low-resolution and both amplitudes and phases within the missing data zone are refined. Refining both phases and amplitudes are  more challenging than extending the phases only.

The question regarding the resolution limitation of single-molecule 3D reconstruction is not what this paper focused on. However, in our early publication^5^, we have discussed a theoretical limitation of resolution at that beyond ~20 Å is possible. It is because a high-reputed theoretical paper predicts 20 Å as the resolution limitation^71^. In this paper, two key parameters used were underestimated, *i.e*. the total dose of 5–20 e^-^/Å ^2^ is significantly lower than that used in the real experiments (>~100 e^-^/Å ^2^), and the solvent contrast factor of 0.28 of x-ray scattering is lower than that of electron scattering^71^. With a higher dose and a higher contrast factor, that resolution higher than 20 Å is reasonable to be expected^5^. What the limitation of single-molecule 3D resolution is remaining undefined.

Our method is different from  the widely used “projection onto the convex set (POCS)” method^72^. In POCS, the high-resolution feasible solutions are refined under a set of constraints, such as smoothness, energy boundedness, and consistency of data observations. Although the POCS algorithm has been applied to cases with any smooth movement, linear variable airspace vagueness, nonuniform additive noise and reduced distortions caused by missing data in 3D reconstruction, the POCS algorithm does not perform well in the reconstruction of high-frequency information and depressing noise^73^. Moreover, the POCS, as well as its modified methods, such as the conventional hybrid input-output (HIO) algorithm for the phase retrieval of oversampled diffraction intensities^74^, requires high-contrast images from hard materials or a priori knowledge, such as the possible density range or the overall size of the reconstructed object. Additionally, the degradation factors of POCS include blur and movement^75^. In our method, we do not need prior knowledge of the density range and the exact overall size of the reconstructed object but can handle low-SNR and low-tilt images without generating significant blur and movement.

In our approach, we proposed a theory that the tilt angle range of ±90° is not necessary for a complete 3D reconstruction and that a low-tilt angle range, such as ±45°, would be possible for a complete 3D reconstruction. This statement seems contrary to Orlov’s sufficiency condition for a complete 3D reconstruction^76,77^, *i.e*., “*a complete data set can be obtained if every great circle intersects the trajectory of the unit vector θ, which is the direction of the parallel rays*.”^78^ The trajectory of the unit vector in our LoTToR method did not intersect with all great circles. Thus, the direct reconstruction from the low-tilt series was not sufficient to give a complete 3D map, as shown by the significant missing-wedge artifact, which is consistent with Orlov’s sufficiency condition. However, in our method, we used the iteration method to fill in or restore the data within the missing-wedge zone. The combination of the observed data and restored missing data together satisfy Orlov’s sufficiency condition for a complete 3D reconstruction.

## Conclusions

In this paper, we proposed a theory that a tilt angle range beyond ±45° was not a necessary condition for a complete 3D reconstruction. For a simple 3D object, two tilt images are sufficient to determine all coordinates of atoms in the object. The greater the number of atoms an object has, the more tilt images are needed. A nearly complete 3D reconstruction can be achieved from a low-tilt series by our reported LoTToR method, in which the missing-wedge data are restored from the observed low-tilt data via a model-free refinement under a set of constraints. The LoTToR method was first characterized by a phantom and then validated by both NS and cryo-EM experimental tilt series. The ability to reduce the missing-wedge artifact suggests that the method can be used as a post-processing tool for IPET 3D reconstruction by enhancing the power of the single-molecule 3D structure studying in the macromolecular 3D dynamics.

## Methods

### Preparation and analyses of the simulated reconstructions

Two crystal structures used to generate the simulation ET data were GroEL (D_7_ symmetry, molecular mass of ~800 kDa, PDB code of 1KP8^56^) and a fragment of ModB_2_C_2_ (C_2_ symmetry, molecular mass of ~108 kDa, PDB code of 2ONK^57^). The 3D object of GroEL was generated by the “*pdb2mrc*” command (EMAN software package^59^) within a box of 256 × 256 × 256 voxels (each voxel corresponded to 1 × 1 × 1 Å^3^ in real space to simulate an EM image under a magnification of ~100 K× and acquired by a 4 K × 4 K CCD camera). The 3D object of ModB_2_C_2_ was generated from the crystal structures by the “*pdb2mrc*” command (EMAN software package) within a box of 160 × 160 × 160 voxels for ModB_2_C_2_, with a voxel size of 1 × 1 × 1 Å^3^. The 2D projections of the map within a tilt angle range from -90° to +90° in steps of 1.5° (along the Y axis) were generated by the “*PJ 3Q*” command (SPIDER software package^79^) to simulate a complete tilt series under noise-free conditions. The 3D reconstruction was conducted by using the “*BP 3 F*” command in SPIDER based on the demanded tilt angle ranges, such as ±90°, ±60°, ±45°, ±30°, and ±15°, of which the ±90° reconstruction was used as the ideal reconstruction.

The 3D reconstructions of GroEL and ModB_2_C_2_ under noise conditions were generated as followings. The tilt series under SNRs of 1.0, 0.5, and 0.3 were generated by adding Gaussian noise to the noise-free projections using the “*AD*” command in SPIDER. The SNR of 2D images was measured by using the equation *SNR* $=({I}_{s}-{I}_{b})/{N}_{b}$, where *I*_*s*_ is the average intensity inside a particle, I_b_ is the average intensity outside a particle, and N_b_ is the standard deviation (*s.d*.) of the noise calculated from the background^5,25^. A Gaussian noise with a *s.d*. 3.3 times higher than that of the particle was added to the tilt series to simulate the noise level at SNR = 0.3 using the “*MO*” and “*AD*” commands in the SPIDER software package^79,80^. The noisy tilt series were then used for 3D reconstructions.

To quantitatively evaluate the quality of the missing-wedge-restored 3D map, Fourier shell correlation (FSC) and real-space cross-correlation coefficient (CCC) analyses were used. The FSC curves were computed between the 3D reconstructions and the reference map by the “*RF 3*” command, and the CCC value was calculated by the “*CC C*” command in SPIDER^81–83^. The frequency at which the FSC curve falls to a value of 0.5 was used to represent the resolution of the 3D reconstruction^59,79,84^.

### Preparation of the NS-EM specimens

The NS specimens of CETP, DNA-nanogold conjugates, and the nucleosome were prepared by the OpNS protocol^8,19,20,22,24^. Briefly, an aliquot (∼4 μl) of the sample was placed on a thin carbon-coated 200-mesh copper grid (Cu-200CN, Pacific Grid-Tech, San Francisco, CA, USA) that had been glow-discharged. After ∼1 min of incubation, the excess solution on the grid was blotted with filter paper. Then, the grid was washed with water and stained with 1% (w/v) uranyl formate on Parafilm before air-drying with nitrogen.

### Preparation of the cryo-EM specimens

The liposome vesicle samples were produced by Encapsula NanoSciences (Brentwood, TN)^36^. The sample contained 1 mg/mL 1-palmitoyl-2-oleoylphosphatidylcholine (POPC, from Avanti Polar lipids) with a peak vesicle size of ~50 nm in a buffer containing 20 mM Tris-Cl and 154 mM NaCl, pH 7.4. Human LDLs were isolated from the plasma of a healthy individual by density gradient ultracentrifugation (produced by Dr. Krauss from the Children’s Hospital Oakland Research Institute) as reported^21,34,58^. To prepare the LDL-CETP mixture sample, LDL was incubated with recombinant human CETP (expressed and purified from the Chinese hamster ovary cell line DG441 by MERCK) at 37 °C for 15 minutes at a molar ratio of ~4:1^20^. We used a high concentration of CETP to ensure the binding of LDL and CETP. To prepare the cryo-EM specimens, an aliquot (∼3 μl) of liposome vesicle, LDL or LDL-CETP mixture sample was placed on a glow-discharged holy-carbon grid (Cu-200HN, Pacific Grid-Tech, San Francisco, CA, USA). Then, the samples were flash-frozen in liquid ethane at ~90% humidity and 4 °C with a Leica EM GP rapid-plunging device (Leica, Buffalo Grove, IL, USA) after being blotted with filter paper.

### ET data acquisition and image preprocessing

The NS sample of CETP was imaged by an FEI Tecnai T12 TEM (FEI, Oregon, America), while other samples were imaged by a Zeiss Libra 120 TEM (Carl Zeiss SMT GmbH, Oberkochen, Germany). Both TEMs were operated at 120 kV. The tilt series of the CETP NS sample was collected from −60° to +60° in steps of 1.5° at a nominal magnification of 67 k× (1.73 Å/pixel). The tilt series of the DNA-nanogold was collected from −60° to +60° in steps of 1.5° at a nominal magnification of 125 k× (0.94 Å/pixel)^8^. The tilt series of the nucleosome was collected from −61.5° to +61.5° in steps of 1.5° at a nominal magnification of 80 k× (1.48 Å/pixel)^24^. The tilt series of the liposome was collected from −57° to +60° in steps of 1.5° at a nominal magnification of 50 k× (2.4 Å/pixel)^36^. The tilt series of LDL were collected from −57° to +52.5° in steps of 1.5° at a nominal magnification of 80 k× (1.48 Å/pixel) and from −63° to +60° in steps of 1.5° at a nominal magnification of 125 k× (0.94 Å/pixel). The tilt series of the LDL-CETP mixture was collected from −57° to +57° in steps of 1.5° at a nominal magnification of 50 k× (4.8 Å/pixel). On Zeiss Libra 120, the low-dose data of the cryo-EM samples were acquired by using the in-house developed fully mechanically controlled automated ET software^24^ and Gatan TEM tomography software (Advanced Tomography mode Gatan Inc., Pleasanton, CA, USA).

### IPET 3D reconstruction

The defocus values of each tilt image were measured by using the program *tomoctffind.exe* and then corrected by the program *ctfcorrect.exe* in the TomoCTF software^61^. To reconstruct the 3D structures of individual particles, the images of each targeted particle were tracked and windowed from tilt series of micrographs (within the tilt angle range from -45° to +45°) and then submitted to low-pass filtering and a contrast enhancement algorithm^62^ before 3D reconstruction by IPET^5^. The resolution was estimated based on the FSC at the frequency at which it first falls to a value of 0.5. The FSC curve was calculated by two 3D reconstructions that were generated from odd- or even-numbered index aligned images. The obtained reconstructions were submitted for 1,000 iterations of missing-wedge correction to restore the missing data.

## Supplementary information

Supplementary information.

## References

[1] Hoppe W, Gassmann J, Hunsmann N, Schramm HJ, Sturm M (1974). Three-dimensional reconstruction of individual negatively stained yeast fatty-acid synthetase molecules from tilt series in the electron microscope. Hoppe-Seyler’s Z. fur physiologische Chem..

[2] Pronin E, Lin DY, Ross L (2016). The Bias Blind Spot: Perceptions of Bias in Self Versus Others. Personality Soc. Psychol. Bull..

[3] Lee CJ, Sugimoto CR, Zhang G, Cronin B (2013). Bias in peer review. J. Am. Soc. Inf. Sci. Technol..

[4] Editorials. In praise of replication studies and null results. *Nature* **578**, 489–490 (2020). 10.1038/d41586-020-00530-6 32099132

[5] Zhang L, Ren G (2012). IPET and FETR: experimental approach for studying molecular structure dynamics by cryo-electron tomography of a single-molecule structure. PLoS One.

[6] Milne JL, Subramaniam S (2009). Cryo-electron tomography of bacteria: progress, challenges and future prospects. Nat. reviews. Microbiology.

[7] Koning RI, Koster AJ (2009). Cryo-electron tomography in biology and medicine. Ann. Anat..

[8] Zhang L (2016). Three-dimensional structural dynamics and fluctuations of DNA-nanogold conjugates by individual-particle electron tomography. Nat. Commun..

[9] Frank, J. *Electron Tomography, Methods for Three-Dimensional Visualization of Structures in the Cell*. (Springer, 2006).

[10] Skoglund U, Andersson K, Strandberg B, Daneholt B (1986). Three-dimensional structure of a specific pre-messenger RNP particle established by electron microscope tomography. Nature.

[11] Mehlin H, Daneholt B, Skoglund U (1992). Translocation of a specific premessenger ribonucleoprotein particle through the nuclear pore studied with electron microscope tomography. Cell.

[12] Sandin S, Ofverstedt LG, Wikstrom AC, Wrange O, Skoglund U (2004). Structure and flexibility of individual immunoglobulin G molecules in solution. Structure.

[13] Skoglund U, Ofverstedt LG, Burnett RM, Bricogne G (1996). Maximum-entropy three-dimensional reconstruction with deconvolution of the contrast transfer function: a test application with adenovirus. J. Struct. Biol..

[14] Zhao Q, Ofverstedt LG, Skoglund U, Isaksson LA (2004). Morphological variation of individual Escherichia coli 50S ribosomal subunits *in situ*, as revealed by cryo-electron tomography. Exp. Cell Res..

[15] Fera A (2012). Direct visualization of CaMKII at postsynaptic densities by electron microscopy tomography. J. Comp. Neurol..

[16] Tong H (2013). Peptide-conjugation induced conformational changes in human IgG1 observed by optimized negative-staining and individual-particle electron tomography. Sci. Rep-Uk.

[17] Iwasaki K (2005). Electron tomography reveals diverse conformations of integrin alphaIIbbeta3 in the active state. J. Struct. Biol..

[18] Ercius P, Alaidi O, Rames MJ, Ren G (2015). Electron Tomography: A Three-Dimensional Analytic Tool for Hard and Soft Materials Research. Adv. Mater..

[19] Rames, M., Yu, Y. & Ren, G. Optimized negative staining: a high-throughput protocol for examining small and asymmetric protein structure by electron microscopy. *Journal of visualized experiments: JoVE*, **e51087** (2014). 10.3791/51087 PMC4710468 25145703

[20] Zhang L (2012). Structural basis of transfer between lipoproteins by cholesteryl ester transfer protein. Nat. Chem. Biol..

[21] Zhang L (2011). Morphology and structure of lipoproteins revealed by an optimized negative-staining protocol of electron microscopy. J. Lipid Res..

[22] Zhang L (2010). An optimized negative-staining protocol of electron microscopy for apoE4 POPC lipoprotein. J. Lipid Res..

[23] Jones MK (2010). Assessment of the validity of the double superhelix model for reconstituted high density lipoproteins: a combined computational-experimental approach. J. Biol. Chem..

[24] Liu J (2016). Fully Mechanically Controlled Automated Electron Microscopic Tomography. Sci. Rep-Uk.

[25] Wu H (2018). An Algorithm for Enhancing the Image Contrast of Electron Tomography. Sci. Rep-Uk.

[26] Lei D (2018). Three-dimensional structural dynamics of DNA origami Bennett linkages using individual-particle electron tomography. Nat. Commun..

[27] Zhang X (2015). 3D Structural Fluctuation of IgG1 Antibody Revealed by Individual Particle Electron Tomography. Sci. Rep-Uk.

[28] Jay JW (2018). IgG Antibody 3D Structures and Dynamics. Antibodies.

[29] Lei D (2019). Single-Molecule 3D Images of “Hole-Hole” IgG1 Homodimers by Individual-Particle Electron Tomography. Sci. Rep-Uk.

[30] Zhang M (2018). Structural basis of the lipid transfer mechanism of phospholipid transfer protein (PLTP). Biochim. Biophys. Acta Mol. Cell Biol. Lipids.

[31] Lu Z (2016). Molecular Architecture of Contactin-associated Protein-like 2 (CNTNAP2) and Its Interaction with Contactin 2 (CNTN2). J. Biol. Chem..

[32] Lu Z (2014). Calsyntenin-3 molecular architecture and interaction with neurexin 1alpha. J. Biol. Chem..

[33] Liu, J. *et al*. Structural Plasticity of Neurexin 1alpha: Implications for its Role as Synaptic Organizer. *Journal of molecular biology* (2018). 10.1016/j.jmb.2018.08.026 PMC6223652 30193986

[34] Lei D (2019). Single-molecule 3D imaging of human plasma intermediate-density lipoproteins reveals a polyhedral structure. Biochim. Biophys. Acta Mol. Cell Biol. Lipids.

[35] Yu YD (2016). Polyhedral 3D structure of human plasma very low density lipoproteins by individual particle cryo-electron tomography. J. Lipid Res..

[36] Zhang M (2015). HDL surface lipids mediate CETP binding as revealed by electron microscopy and molecular dynamics simulation. Sci. Rep-Uk.

[37] Koster AJ (1997). Perspectives of molecular and cellular electron tomography. J. Struct. Biol..

[38] Paavolainen L (2014). Compensation of missing wedge effects with sequential statistical reconstruction in electron tomography. PLoS One.

[39] Goris B, V den Broek W, Batenburg KJ, Heidari Mezerji H, Bals S (2012). Electron tomography based on a total variation minimization reconstruction technique. Ultramicroscopy.

[40] Palmer CM, Lowe J (2014). A cylindrical specimen holder for electron cryo-tomography. Ultramicroscopy.

[41] Kawase N, Kato M, Nishioka H, Jinnai H (2007). Transmission electron microtomography without the “missing wedge” for quantitative structural analysis. Ultramicroscopy.

[42] Zheng SQ, Matsuda A, Braunfeld MB, Sedat JW, Agard DA (2009). Dual-axis target mapping and automated sequential acquisition of dual-axis EM tomographic data. J. Struct. Biol..

[43] Penczek P, Marko M, Buttle K, Frank J (1995). Double-tilt electron tomography. Ultramicroscopy.

[44] Arslan I, Tong JR, Midgley PA (2006). Reducing the missing wedge: High-resolution dual axis tomography of inorganic materials. Ultramicroscopy.

[45] Guesdon A, Blestel S, Kervrann C, Chretien D (2013). Single versus dual-axis cryo-electron tomography of microtubules assembled *in vitro*: limits and perspectives. J. Struct. Biol..

[46] Ercius P, Alaidi O, Rames MJ, Ren G (2015). Electron Tomography: A Three-Dimensional Analytic Tool for Hard and Soft Materials Research. Adv. Mater..

[47] Subramaniam S, Zhang PJ, Lefman J, Juliani J, Kessel M (2003). Electron tomography: a powerful tool for 3D cellular microscopy. Asm N..

[48] Bartesaghi A (2008). Classification and 3D averaging with missing wedge correction in biological electron tomography. J. Struct. Biol..

[49] Schur FK (2016). An atomic model of HIV-1 capsid-SP1 reveals structures regulating assembly and maturation. Science.

[50] Schur FK, Hagen WJ, de Marco A, Briggs JA (2013). Determination of protein structure at 8.5A resolution using cryo-electron tomography and sub-tomogram averaging. J. Struct. Biol..

[51] Yu Z, Frangakis AS (2011). Classification of electron sub-tomograms with neural networks and its application to template-matching. J. Struct. Biol..

[52] Agard DA, Stroud RM (1982). Linking regions between helices in bacteriorhodopsin revealed. Biophysical J..

[53] Batenburg KJ, Sijbers J (2011). DART: a practical reconstruction algorithm for discrete tomography. IEEE Trans. Image Process..

[54] Deng Y (2016). ICON: 3D reconstruction with ‘missing-information’ restoration in biological electron tomography. J. Struct. Biol..

[55] Yan R, Venkatakrishnan SV, Liu J, Bouman CA, Jiang W (2019). MBIR: A cryo-ET 3D reconstruction method that effectively minimizes missing wedge artifacts and restores missing information. J. Struct. Biol..

[56] Wang J, Boisvert DC (2003). Structural basis for GroEL-assisted protein folding from the crystal structure of (GroEL-KMgATP)14 at 2.0A resolution. J. Mol. Biol..

[57] Hollenstein K, Frei DC, Locher KP (2007). Structure of an ABC transporter in complex with its binding protein. Nature.

[58] Yu Y (2016). Polyhedral 3D structure of human plasma very low density lipoproteins by individual particle cryo-electron tomography1. J. Lipid Res..

[59] Ludtke SJ, Baldwin PR, Chiu W (1999). EMAN: semiautomated software for high-resolution single-particle reconstructions. J. Struct. Biol..

[60] Leschziner AE, Nogales E (2006). The orthogonal tilt reconstruction method: an approach to generating single-class volumes with no missing cone for ab initio reconstruction of asymmetric particles. J. Struct. Biol..

[61] Fernandez JJ, Li S, Crowther RA (2006). CTF determination and correction in electron cryotomography. Ultramicroscopy.

[62] Wu H (2018). An Algorithm for Enhancing the Image Contrast of Electron Tomography. Sci. Rep..

[63] Qiu XY (2007). Crystal structure of cholesteryl ester transfer protein reveals a long tunnel and four bound lipid molecules. Nat. Struct. Mol. Biol..

[64] Drew HR (1981). Structure of a B-DNA dodecamer: conformation and dynamics. Proc. Natl Acad. Sci. USA.

[65] Richmond TJ (1999). Hot papers - Crystal structure - Crystal structure of the nucleosome core particle at 2.8 angstrom resolution by K. Luger, A.W. Mader, R.K. Richmond, D.F. Sargent, T.J. Richmond - Comments. Scientist.

[66] Chien FT, van der Heijden T (2014). Characterization of nucleosome unwrapping within chromatin fibers using magnetic tweezers. Biophysical J..

[67] Kahlon TS, Shore VG, Lindgren FT (1992). Heterogeneity of molecular weight and apolipoproteins in low density lipoproteins of healthy human males. Lipids.

[68] Qiu X (2007). Crystal structure of cholesteryl ester transfer protein reveals a long tunnel and four bound lipid molecules. Nat. Struct. Mol. Biol..

[69] Wang BC (1985). Resolution of phase ambiguity in macromolecular crystallography. Methods Enzymol..

[70] Gaykema WPJ (1984). 3.2 Å structure of the copper-containing, oxygen-carrying protein Panulirus interruptus haemocyanin. Nature.

[71] Rosenthal PB, Henderson R (2003). Optimal determination of particle orientation, absolute hand, and contrast loss in single-particle electron cryomicroscopy. J. Mol. Biol..

[72] Stark H, Oskoui P (1989). High-resolution image recovery from image-plane arrays, using convex projections. J. Opt. Soc. Am. A.

[73] Guo, L. & He, Z. In 2*008 9th International Conference on Signal Processing* 1039–1041 (IEEE, Beijing 2008).

[74] Chen, C.-C., Miao, J., Wang, C. W. & Lee, T. K. Application of optimization technique to noncrystalline x-ray diffraction microscopy: Guided hybrid input-output method. *Physical Review B* **76** (2007).

[75] Fan, C., Wu, C., Li, G. & Ma, J. Projections onto Convex Sets Super-Resolution Reconstruction Based on Point Spread Function Estimation of Low-Resolution Remote Sensing Images. *Sensors (Basel)* **17** (2017). 10.3390/s17020362 PMC5335938 28208837

[76] Penczek PA (2010). Fundamentals of three-dimensional reconstruction from projections. Methods Enzymol..

[77] Orlov SS (1976). Theory of three-dimensional reconstruction 1. Conditions for a complete set of projections. Sov. Phys. Crystallography.

[78] Zeng, G. In *Medical Image Reconstruction: A Conceptual Tutorial* (ed Gengsheng Zeng) Ch. **5**, 89 (Springer 2010).

[79] Frank J (1996). SPIDER and WEB: processing and visualization of images in 3D electron microscopy and related fields. J. Struct. Biol..

[80] Shaikh TR (2008). SPIDER image processing for single-particle reconstruction of biological macromolecules from electron micrographs. Nat. Protoc..

[81] Saxton WO, Baumeister W (1982). The correlation averaging of a regularly arranged bacterial cell envelope protein. J. microscopy.

[82] van Heel M, Stoffler-Meilicke M (1985). Characteristic views of E. coli and B. stearothermophilus 30S ribosomal subunits in the electron microscope. EMBO J..

[83] Unser M, Trus BL, Steven AC (1987). A new resolution criterion based on spectral signal-to-noise ratios. Ultramicroscopy.

[84] Bottcher B, Wynne SA, Crowther RA (1997). Determination of the fold of the core protein of hepatitis B virus by electron cryomicroscopy. Nature.

