# Supplementary material for: LoTToR: An Algorithm for Missing-Wedge Correction of the Low-Tilt Tomographic 3D Reconstruction of a Single-Molecule Structure
Source: Sci Rep. 2020 Jun 26;10:10489. doi: 10.1038/s41598-020-66793-1 (PMC7320192; doi:10.1038/s41598-020-66793-1)
Supplement: Supplementary file 1 — Supplementary information. [file 41598_2020_66793_MOESM1_ESM.pdf]

Supplementary information
for

LoTToR: An Algorithm for Missing-Wedge Correction of the Low-Tilt Tomographic 3D Reconstruction of a Single-Molecule Structure

Xiaobo Zhai, Dongsheng Lei, Meng Zhang, Jianfang Liu, Hao Wu, Yadong Yu, Lei Zhang, Gang Ren

Supplementary Table 1. The resolution and CCC of the phantoms before and after the missing-wedge correction. The phantoms are the 3D maps of GroEL and $ModB_2C_2$ reconstructed from noise-free simulated tilt series.

Tilt angle range	GroEL without noise				$ModB_2C_2$ without noise			
	Resolution (Å) @ FSC=0.5		CC C		Resolution (Å) @ FSC=0.5		CC C	
	Before corr.	After corr.	Before corr.	After MW	Before corr.	After corr.	Before corr.	After corr.
**±15°**	11.6	4.1	0.32	0.37	25.9	3.6	0.36	0.46
**±30°**	2.3	2.1	0.44	0.53	2.1	2.0	0.48	0.60
**±45°**	2.0	1.8	0.54	0.67	1.8	1.6	0.58	0.72
**±60°**	1.8	1.5	0.62	0.74	1.6	1.3	0.66	0.78

Supplementary Table 2. The resolution and CCC of the phantom before and after the missing-wedge correction. The phantom is a simulated 3D density map of GroEL reconstructed from noisy tilt series.

Tilt angle range	GroEL with noise																				
	SNR=0.3 (ideal resolution=15.3 Å)							SNR=0.5 (ideal resolution=9.8 Å)							SNR=1.0 (ideal resolution=4.5 Å)						
	No filtering				Low-pass filter (4 Å)			No filtering				Low-pass filter (4 Å)			No filtering				Low-pass filter (4 Å)		
	Res. (Å)		CC C		Res. (Å)	CC C		Res. (Å)		CC C		Res. (Å)	CC C		Res. (Å)		CC C		Res. (Å)	CC C	
	Bef.	Aft.	Bef.	Aft.	Aft.	Bef.	Aft.	Bef.	Aft.	Bef.	Aft.	Aft.	Bef.	Aft.	Bef.	Aft.	Bef.	Aft.	Aft.	Bef.	Aft.
**±15°**	28.9	73.8	0.05	0.05	34.8	0.07	0.11	25.3	36.0	0.08	0.10	30.9	0.11	0.16	24.2	13.4	0.15	0.14	19.5	0.18	0.22
**±30°**	24.4	73.1	0.06	0.07	33.2	0.08	0.13	18.6	15.9	0.10	0.10	14.5	0.13	0.18	11.5	8.7	0.18	0.19	8.9	0.21	0.26
**±45°**	21.6	71.6	0.07	0.08	16.4	0.09	0.14	15.4	13.4	0.11	0.12	9.8	0.14	0.20	8.8	7.8	0.21	0.25	4.3	0.23	0.30
**±60°**	18.6	16.9	0.08	0.09	13.1	0.10	0.15	11.6	11.0	0.13	0.23	8.4	0.15	0.22	8.1	6.9	0.24	0.28	4.0	0.25	0.33

Supplementary Table 3. The resolution and CCC of the phantom before and after the missing-wedge correction. The phantom is a simulated 3D density map of $ModB_2C_2$ reconstructed from noisy tilt series.

Tilt angle range	$ModB_2C_2$ with noise																				
	SNR=0.3 (ideal resolution=9.4 Å)							SNR=0.5 (ideal resolution=7.4 Å)							SNR=1.0 (ideal resolution=3.5 Å)						
	No filtering				Low-pass filtering (4 Å)			No filtering				Low-pass filtering (4 Å)			No filtering				Low-pass filtering (4 Å)		
	Res. (Å)		CC C		Res. (Å)	CC C		Res. (Å)		CC C		Res. (Å)	CC C		Res.(Å)		CC C		Res.(Å)	CC C	
	Bef.	Aft.	Bef.	Aft.	Aft.	Bef.	Aft.	Bef.	Aft.	Bef.	Aft.	Aft.	Bef.	Aft.	Bef.	Aft.	Bef.	Aft.	Aft.	Bef.	Aft.
**±15°**	28.8	51.3	0.06	0.08	28.5	0.10	0.19	27.5	43.9	0.10	0.12	15.6	0.15	0.24	26.8	9.4	0.18	0.21	11.6	0.20	0.28
**±30°**	23.2	47.1	0.08	0.11	11.3	0.12	0.23	14.9	10.7	0.13	0.17	4.7	0.18	0.29	10.7	4.2	0.23	0.30	4.00	0.25	0.34
**±45°**	14.7	14.2	0.10	0.12	7.9	0.13	0.25	11.0	8.2	0.15	0.19	4.0	0.20	0.32	4.6	3.7	0.28	0.36	3.92	0.29	0.38
**±60°**	13.7	11.3	0.11	0.13	7.3	0.15	0.27	10.1	7.6	0.17	0.21	4.0	0.32	0.33	4.0	3.5	0.31	0.39	3.91	0.38	0.41

Supplementary Figures

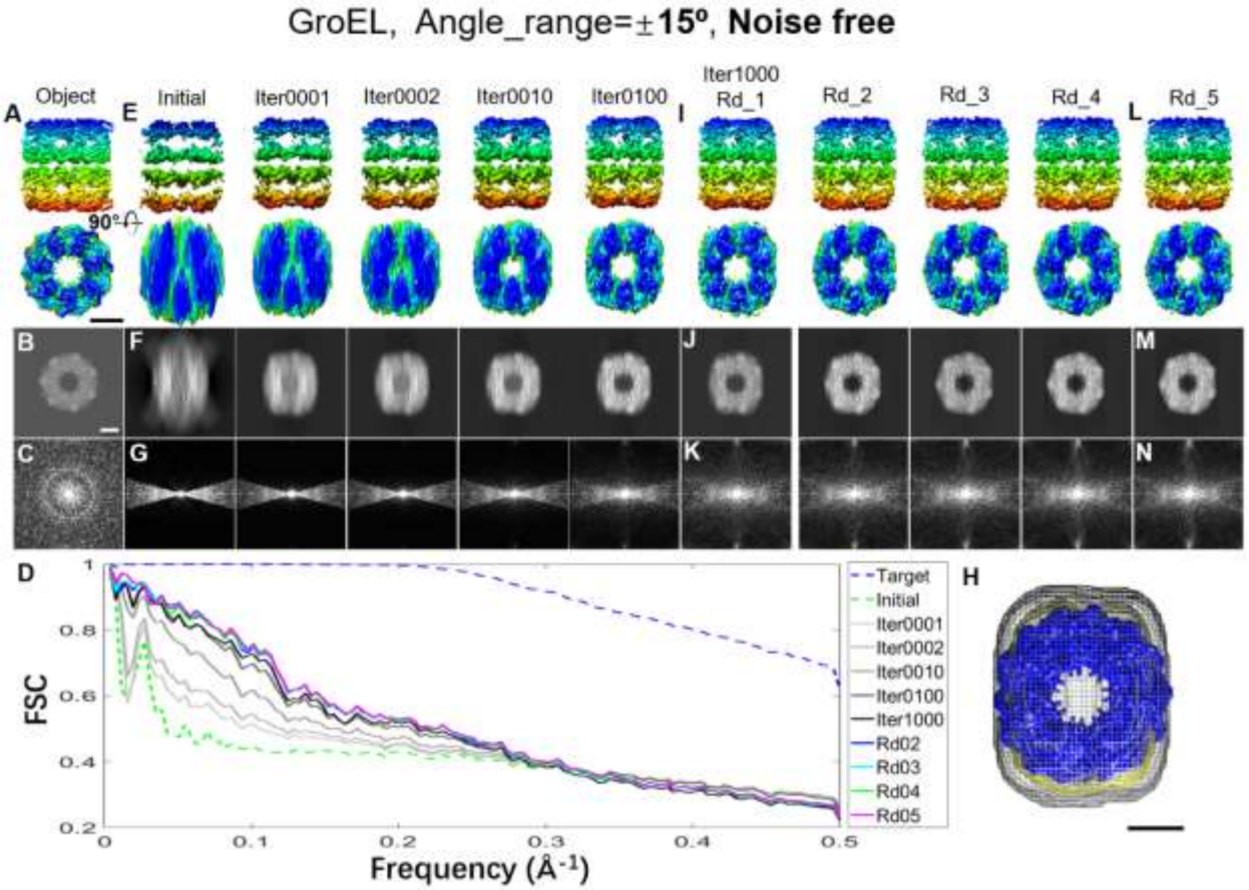

Supplementary Fig. 1 | Under a small volume mask, the missing-wedge correction on a simulated 3D map of GroEL reconstructed from the noise-free ±15° tilt series (A) A 3D object shown from two perpendicular views. The object was a generated from the crystal structure of GroEL and the idea 3D was constructed from the tilt series of the noise-free 2D projections, which were generated by projecting the object from a series of tilt angles in a range of ±90° in steps of 1.5°. (**B**) The projection of the object on the X-Z plane, and (**C**) the corresponding Fourier transform. (**D**) The ideal FSC curve was computed between the object and the ideal 3D map, as shown by the blue dashed line. The other curves were computed between the object and iterative maps. (**E**) The initial 3D and iterative 3D maps, shown from perpendicular views. (**F and G**) Their corresponding projections on the X-Z plane and the corresponding Fourier transforms. (**H**) A small mask corresponding to ~1.75 times the molecular weight of GroEL was generated from the low-passed filtered initial 3D (~60 Å, shown in the mesh; the object is shown in blue). (**I**) The final 3Ds after each round (containing 1,000 cycles of iteration) of missing-wedge correction. The maps of round 1 (Rd_1) and the rounds 2-4 are shown from perpendicular views, and (**J**) their projections on the X-Z plane and (**K**) the corresponding Fourier transforms. (**L-N**) The final 3D after round 5 is shown from perpendicular views, compared to its projection on the X-Z plane and the corresponding Fourier transform. All 3D maps underwent low-pass filtering to 8 Å. Scale bars: 50 nm.

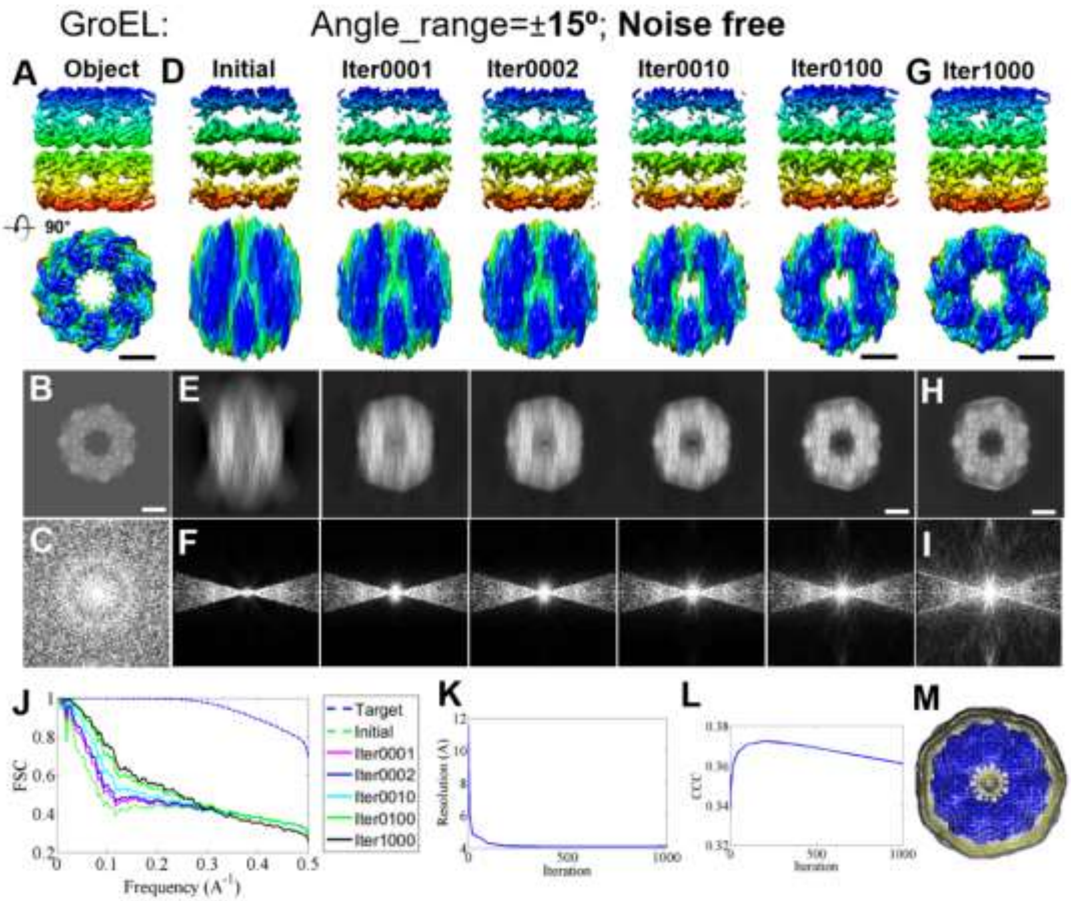

Supplementary Fig. 2 | Under a precious shape and large volume mask, the missing-wedge correction on a simulated 3D map of GroEL reconstructed from the noise-free ±15° tilt series (A) A 3D object shown from two perpendicular views. The object was a generated from the crystal structure of GroEL, and the idea 3D was constructed from the tilt series of the noise-free 2D projections, which are the projections of the object from a tilt angle range of ±90° in steps of 1.5°, while the initial 3D map was reconstructed from that within the angle range of ±15°. (**B**) The projection of the object on the X-Z plane, and (**C**) the corresponding Fourier transform. (**D**) The initial 3D and iterative 3D maps, shown from perpendicular views. (**E**) Their corresponding projections on the X-Z plane and the (**F**) Fourier transforms. (**G**) The final corrected 3D after 1,000 cycles of iteration (round 1, Rd_1), shown from two perpendicular views, and (**H**) the projection along the X-Z plane and (**I**) the corresponding Fourier transform. (**J**) FSC curves of the iterative 3Ds against the object. The blue dashed line is the ideal FSC curve, calculated between the ideal 3D and the object. The dashed green line is the initial FSC, calculated between the initial 3D and the object. The rest solid lines are the iterative FSC calculated between the object and 3Ds after 1 (in purple), 2 (in blue), 10 (in cyan), 100 (in green), and 1,000 (in black) cycles of iteration. (**K**) The plot of the iterative 3D resolutions against the cycles of iteration (the resolution is defined by the frequency at the FSC curve falls to 0.5). (**L**) The plot of the CCC (between the iterative 3D and the object) against the cycles of the iteration. (**M**) The mask corresponding to ~3 times the molecular weight of GroEL was generated from the low-passed filtered object (~40 Å), showed in gray mash, while the object showed in blue. All 3Ds were low-pass filtered to 8 Å. Bars: 50 nm.

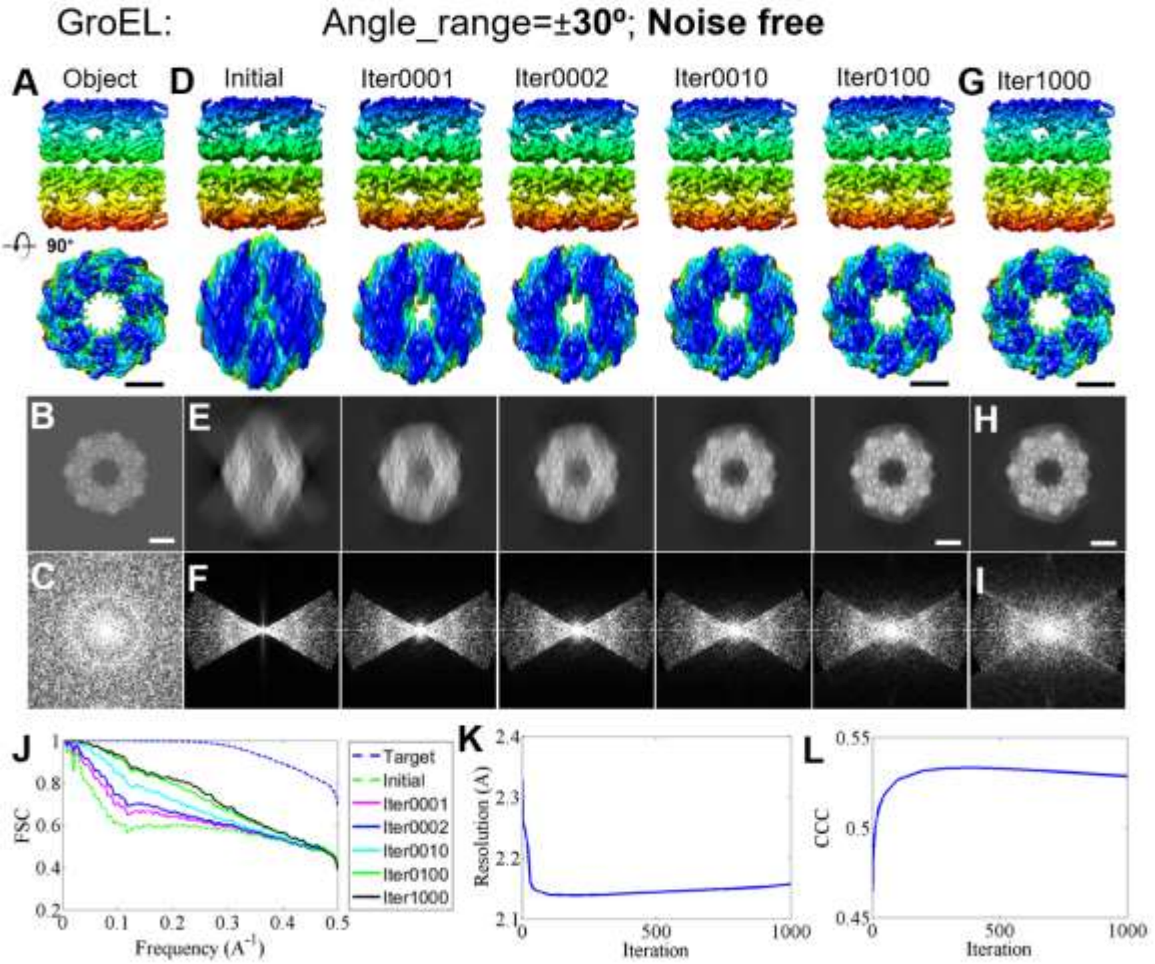

Supplementary Fig. 3 | The missing-wedge correction on a simulated 3D map of GroEL reconstructed from the noise-free ±30° tilt series (A) A 3D object shown from two perpendicular views. The object was a generated from the crystal structure of GroEL, and the idea 3D was constructed from the tilt series of the noise-free 2D projections of the object in a tilt angle range of ±90° in steps of 1.5°, while the initial 3D was reconstructed from angles of ±30°. (**B**) The projection of the object on the X-Z plane, and (**C**) the corresponding Fourier transform. (**D**) The initial 3D and iterative 3D maps, shown from perpendicular views. (**E**) Their corresponding projections on the X-Z plane and the (**F**) Fourier transforms. The mask corresponding to ~3 times the molecular weight of GroEL was generated from the low-passed filtered object (~40 Å). (**G**) The final corrected 3D after 1,000 cycles of iteration (round 1, Rd_1), shown from two perpendicular views, and (**H**) the projection along the X-Z plane and the corresponding (**I**) Fourier transform. (**J**) FSC curves of the iterative 3Ds against the object. The blue dashed line is the ideal FSC curve, calculated between the ideal 3D and the object. The dashed green line is the initial FSC, calculated between the initial 3D and the object. The rest solid lines are the iterative FSC calculated between the object and 3Ds after 1 (in purple), 2 (in blue), 10 (in cyan), 100 (in green), and 1,000 (in black) cycles of iteration. (**K**) The plot of the iterative 3D resolutions against the cycles of iteration (the resolution is defined by the frequency at the FSC curve falls to 0.5). (**L**) The plot of the CCC (between the iterative 3D and the object) against the cycles of the iteration. All 3Ds were low-pass filtered to 8 Å. Bars: 50 nm.

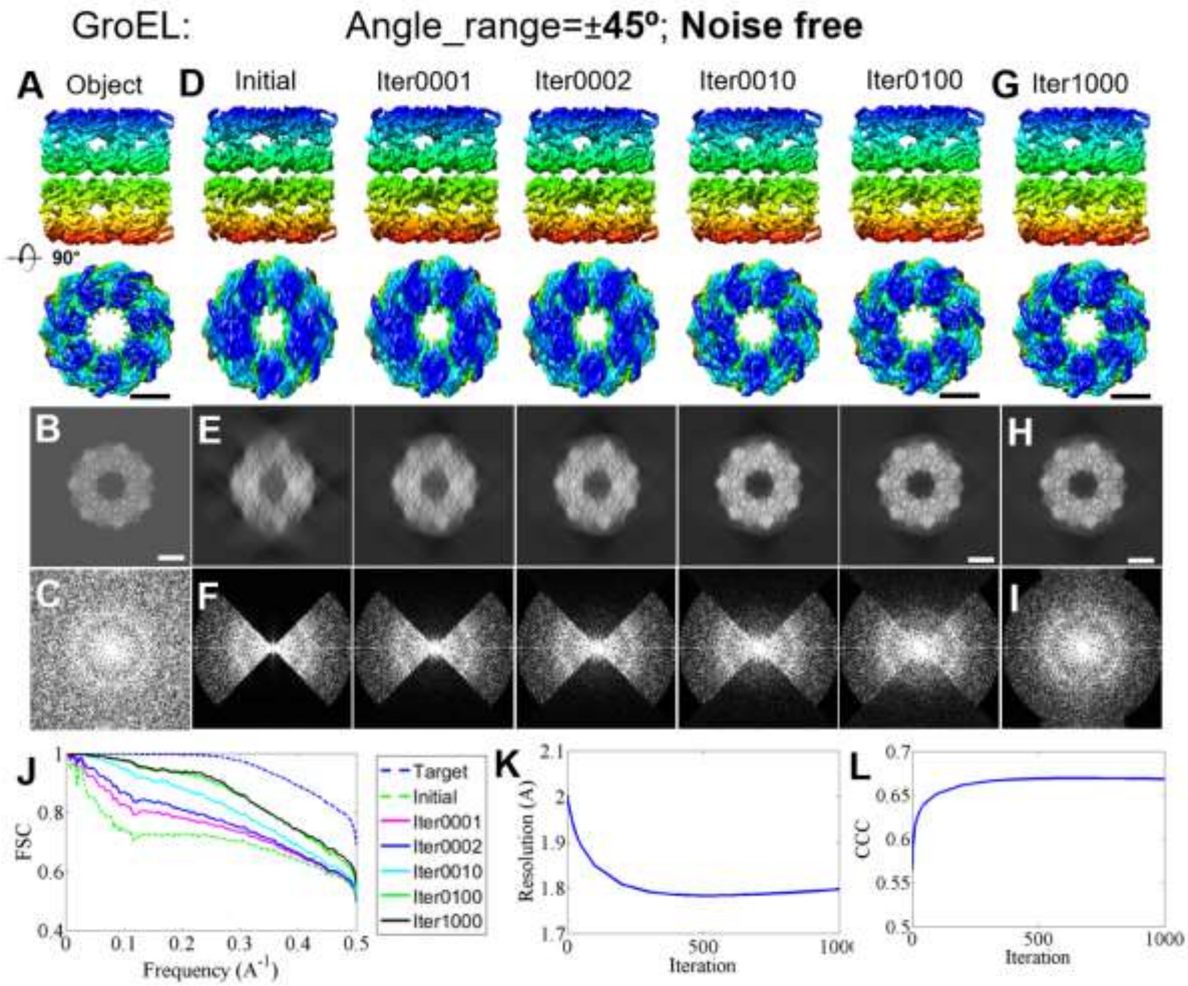

Supplementary Fig. 4 | The missing-wedge correction on a simulated 3D map of GroEL reconstructed from the noise-free ±45° tilt series (A) A 3D object shown from two perpendicular views. The object was a generated from the crystal structure of GroEL, and the idea 3D was constructed from the tilt series of the noise-free 2D projections of the object in a tilt angle range of ±90° in steps of 1.5°, while the initial 3D was reconstructed from angles of ±45°. (**B**) The projection of the object on the X-Z plane, and (**C**) the corresponding Fourier transform. (**D**) The initial 3D and iterative 3D maps, shown from perpendicular views. (**E**) Their corresponding projections on the X-Z plane and the (**F**) Fourier transforms. The mask corresponding to ~3 times the molecular weight of GroEL was generated from the low-passed filtered object (~40 Å). (**G**) The final corrected 3D after 1,000 cycles of iteration (round 1, Rd_1), shown from two perpendicular views, and (**H**) the projection along the X-Z plane and the corresponding (**I**) Fourier transform. (**J**) FSC curves of the iterative 3Ds against the object. The blue dashed line is the ideal FSC curve, calculated between the ideal 3D and the object. The dashed green line is the initial FSC, calculated between the initial 3D and the object. The rest solid lines are the iterative FSC calculated between the object and 3Ds after 1 (in purple), 2 (in blue), 10 (in cyan), 100 (in green), and 1,000 (in black) cycles of iteration. (**K**) The plot of the iterative 3D resolutions against the cycles of iteration (the resolution is defined by the frequency at the FSC curve falls to 0.5). (**L**) The plot of the CCC (between the iterative 3D and the object) against the cycles of the iteration. All 3Ds were low-pass filtered to 8 Å. Bars: 50 nm.

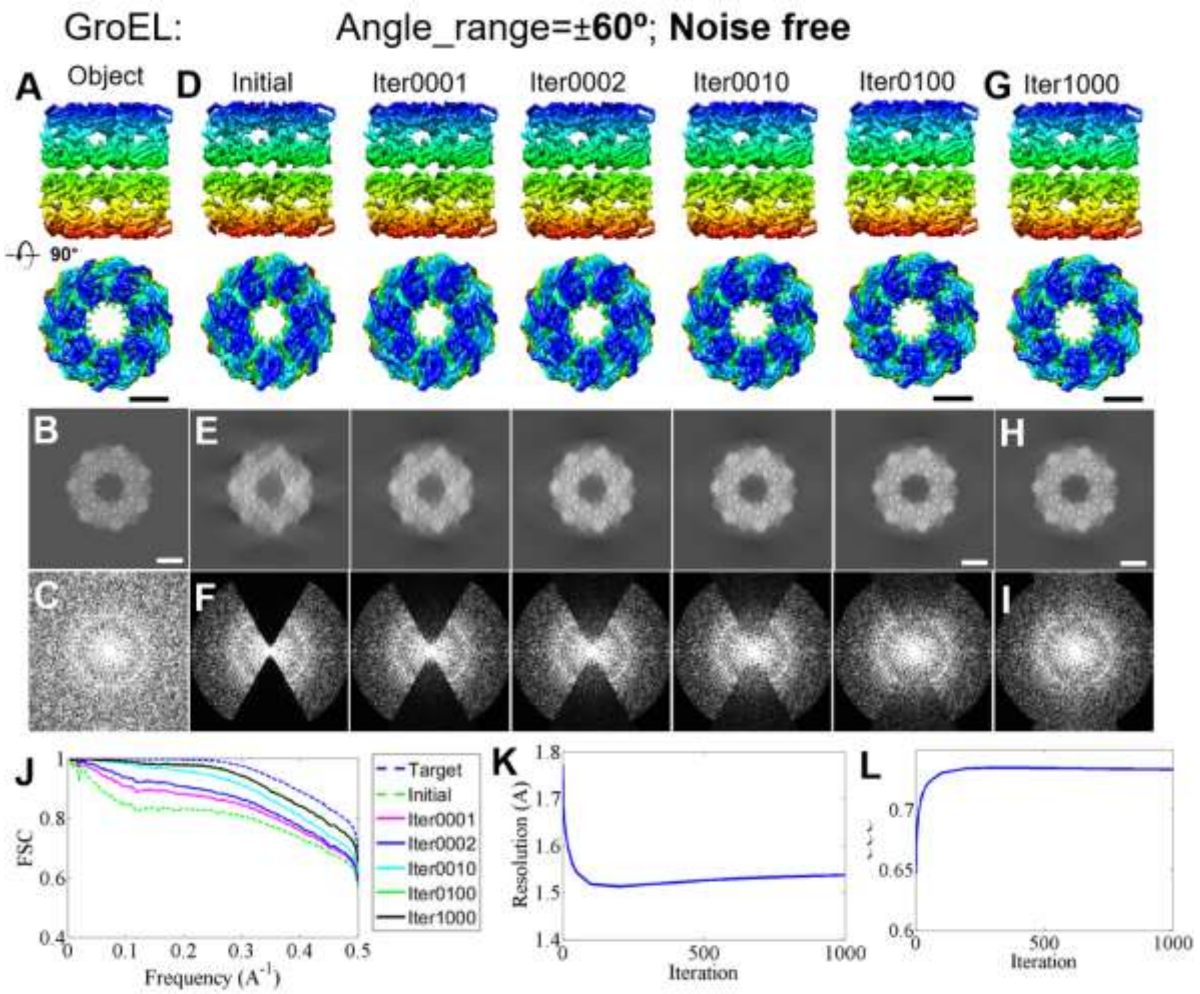

Supplementary Fig. 5 | The missing-wedge correction on a simulated 3D map of GroEL reconstructed from the noise-free ±60° tilt series (A) A 3D object shown from two perpendicular views. The object was a generated from the crystal structure of GroEL, and the idea 3D was constructed from the tilt series of the noise-free 2D projections of the object in a tilt angle range of ±90° in steps of 1.5°, while the initial 3D was reconstructed from angles of ±60°. (**B**) The projection of the object on the X-Z plane, and (**C**) the corresponding Fourier transform. (**D**) The initial 3D and iterative 3D maps, shown from perpendicular views. (**E**) Their corresponding projections on the X-Z plane and the (**F**) Fourier transforms. The mask corresponding to ~3 times the molecular weight of GroEL was generated from the low-passed filtered object (~40 Å). (**G**) The final corrected 3D after 1,000 cycles of iteration (round 1, Rd_1), shown from two perpendicular views, and (**H**) the projection along the X-Z plane and the corresponding (**I**) Fourier transform. (**J**) FSC curves of the iterative 3Ds against the object. The blue dashed line is the ideal FSC curve, calculated between the ideal 3D and the object. The dashed green line is the initial FSC, calculated between the initial 3D and the object. The rest solid lines are the iterative FSC calculated between the object and 3Ds after 1 (in purple), 2 (in blue), 10 (in cyan), 100 (in green), and 1,000 (in black) cycles of iteration. (**K**) The plot of the iterative 3D resolutions against the cycles of iteration (the resolution is defined by the frequency at the FSC curve falls to 0.5). (**L**) The plot of the CCC (between the iterative 3D and the object) against the cycles of the iteration. All 3Ds were low-pass filtered to 8 Å. Bars: 50 nm.

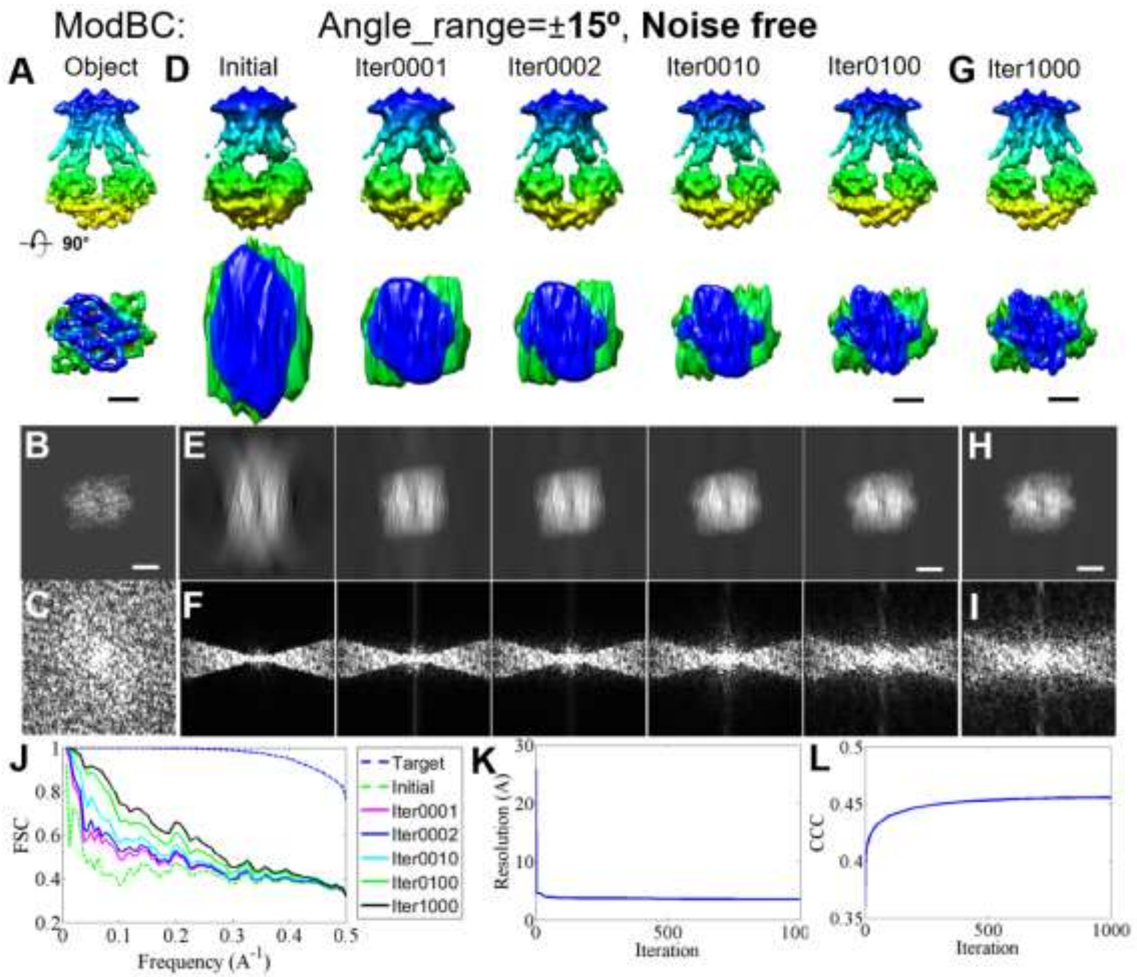

Supplementary Fig. 6 | The missing-wedge correction on a simulated 3D map of $ModB_2C_2$ reconstructed from the noise-free ±15° tilt series (A) A 3D object shown from two perpendicular views. The object was a generated from the crystal structure of $ModB_2C_2$, and the idea 3D was constructed from the tilt series of the noise-free 2D projections, which were generated by projecting the object from a series of tilt angles in a range of ±90° in steps of 1.5°, while the initial 3D was reconstructed from that within the angle range of ±15°. (**B**) The projection of the object on the X-Z plane, and (**C**) the corresponding Fourier transform. (**D**) The initial 3D and iterative 3D maps, shown from perpendicular views. (**E**) Their corresponding projections on the X-Z plane and the (**F**) Fourier transforms. The mask corresponding to ~3 times the molecular weight of $ModB_2C_2$ was generated from the low-passed filtered object (~40 Å). (**G**) The final corrected 3D after 1,000 cycles of iteration (round 1, Rd_1), shown from two perpendicular views, and (**H**) the projection along the X-Z plane and the corresponding (**I**) Fourier transform. (**J**) FSC curves of the iterative 3Ds against the object. The blue dashed line is the ideal FSC curve, calculated between the ideal 3D and the object. The dashed green line is the initial FSC, calculated between the initial 3D and the object. The rest solid lines are the iterative FSC calculated between the object and 3Ds after 1 (in purple), 2 (in blue), 10 (in cyan), 100 (in green), and 1,000 (in black) cycles of iteration. (**K**) The plot of the iterative 3D resolutions against the cycles of iteration (the resolution is defined by the frequency at the FSC curve

falls to 0.5). (**L**) The plot of the CCC (between the iterative 3D and the object) against the cycles of the iteration. All 3Ds were low-pass filtered to 8 Å. Bars: 20 nm.

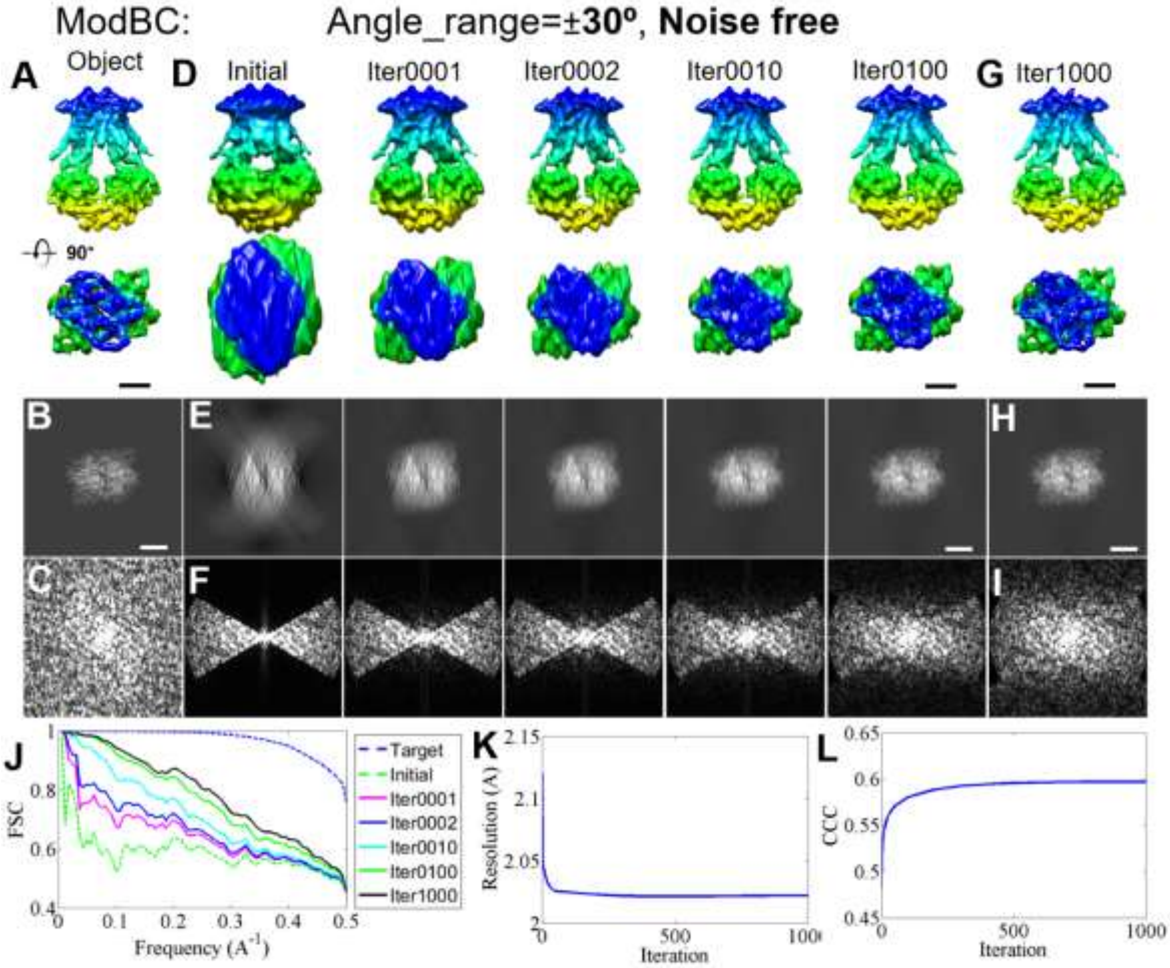

Supplementary Fig. 7 | The missing-wedge correction on a simulated 3D map of ModB$_2$C$_2$ reconstructed from the noise-free ±30° tilt series (A) A 3D object shown from two perpendicular views. The object was a generated from the crystal structure of ModB$_2$C$_2$, and the idea 3D was constructed from the tilt series of the noise-free 2D projections, which were generated by projecting the object from a series of tilt angles in a range of ±90° in steps of 1.5°, while the initial 3D was reconstructed from angles of ±30°. (**B**) The projection of the object on the X-Z plane, and (**C**) the corresponding Fourier transform. (**D**) The initial 3D and iterative 3D maps, shown from perpendicular views. (**E**) Their corresponding projections on the X-Z plane and the (**F**) Fourier transforms. The mask corresponding to ~3 times the molecular weight of ModB$_2$C$_2$ was generated from the low-passed filtered object (~40 Å). (**G**) The final corrected 3D after 1,000 cycles of iteration (round 1, Rd_1), shown from two perpendicular views, and (**H**) the projection along the X-Z plane and the corresponding (**I**) Fourier transform. (**J**) FSC curves of the iterative 3Ds against the object. The blue dashed line is the ideal FSC curve, calculated between the ideal 3D and the object. The dashed green line is the initial FSC, calculated between the initial 3D and the object. The rest solid lines are the iterative FSC calculated between the object and 3Ds after 1 (in purple), 2 (in blue), 10 (in cyan), 100 (in green), and 1,000 (in black) cycles of iteration. (**K**) The plot of the iterative 3D resolutions against

the cycles of iteration (the resolution is defined by the frequency at the FSC curve falls to 0.5). (**L**) The plot of the CCC (between the iterative 3D and the object) against the cycles of the iteration. All 3Ds were low-pass filtered to 8 Å. Bars: 20 nm.

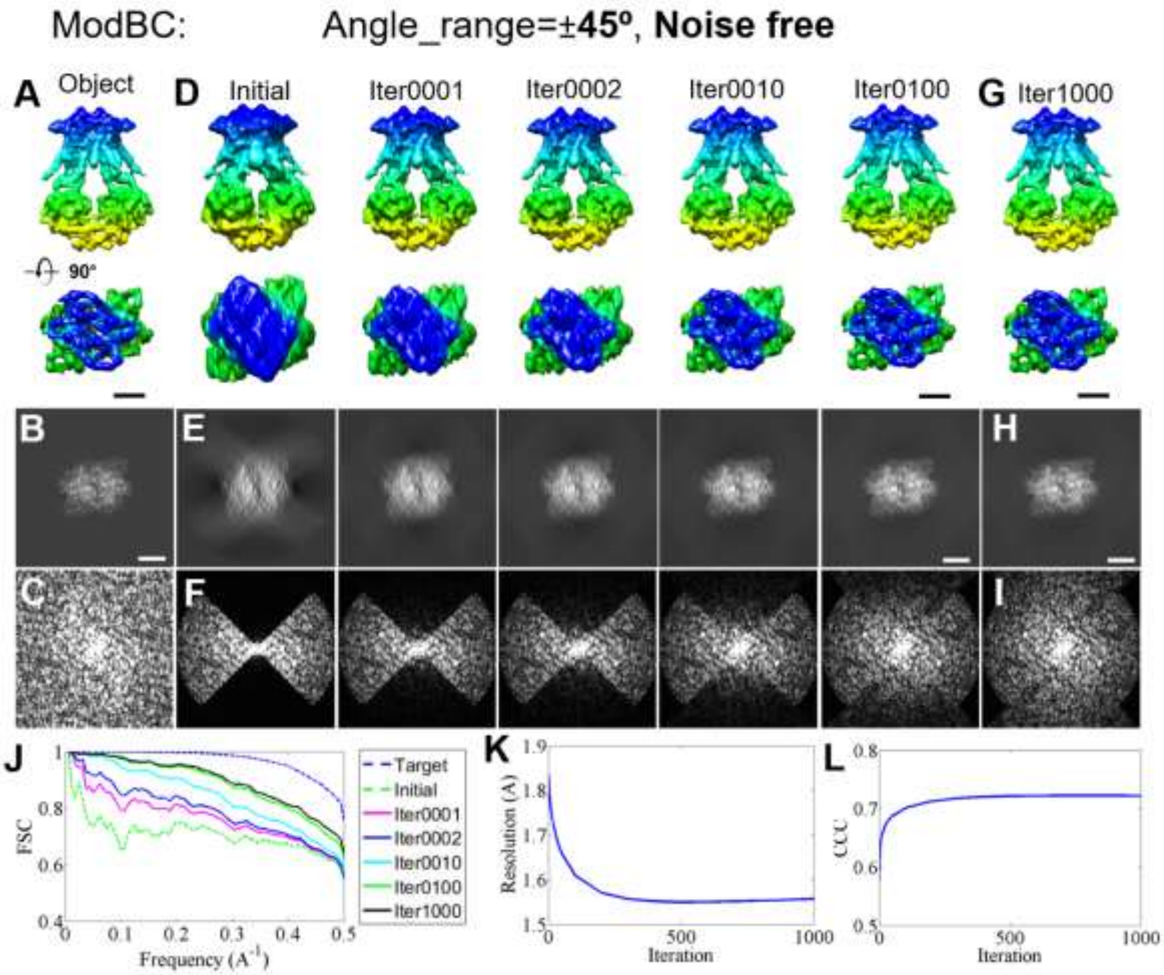

Supplementary Fig. 8 | The missing-wedge correction on a simulated 3D map of $ModB_2C_2$ reconstructed from the noise-free ±45° tilt series (A) A 3D object shown from two perpendicular views. The object was a generated from the crystal structure of $ModB_2C_2$, and the idea 3D was constructed from the tilt series of the noise-free 2D projections, which were generated by projecting the object from a series of tilt angles in a range of ±90° in steps of 1.5°, while the initial 3D was reconstructed from angles of ±45°. (**B**) The projection of the object on the X-Z plane, and (**C**) the corresponding Fourier transform. (**D**) The initial 3D and iterative 3D maps, shown from perpendicular views. (**E**) Their corresponding projections on the X-Z plane and the (**F**) Fourier transforms. The mask corresponding to ~3 times the molecular weight of $ModB_2C_2$ was generated from the low-passed filtered object (~40 Å). (**G**) The final corrected 3D after 1,000 cycles of iteration (round 1, Rd_1), shown from two perpendicular views, and (**H**) the projection along the X-Z plane and the corresponding (**I**) Fourier transform. (**J**) FSC curves of the iterative 3Ds against the object. The blue dashed line is the ideal FSC curve, calculated between the ideal 3D and the object. The dashed green line is the initial FSC, calculated between the initial 3D and the object. The rest solid lines are the iterative FSC calculated between the object and 3Ds after 1 (in purple), 2 (in blue), 10 (in cyan),

100 (in green), and 1,000 (in black) cycles of iteration. (**K**) The plot of the iterative 3D resolutions against the cycles of iteration (the resolution is defined by the frequency at the FSC curve falls to 0.5). (**L**) The plot of the CCC (between the iterative 3D and the object) against the cycles of the iteration. All 3Ds were low-pass filtered to 8 Å. Bars: 20 nm.

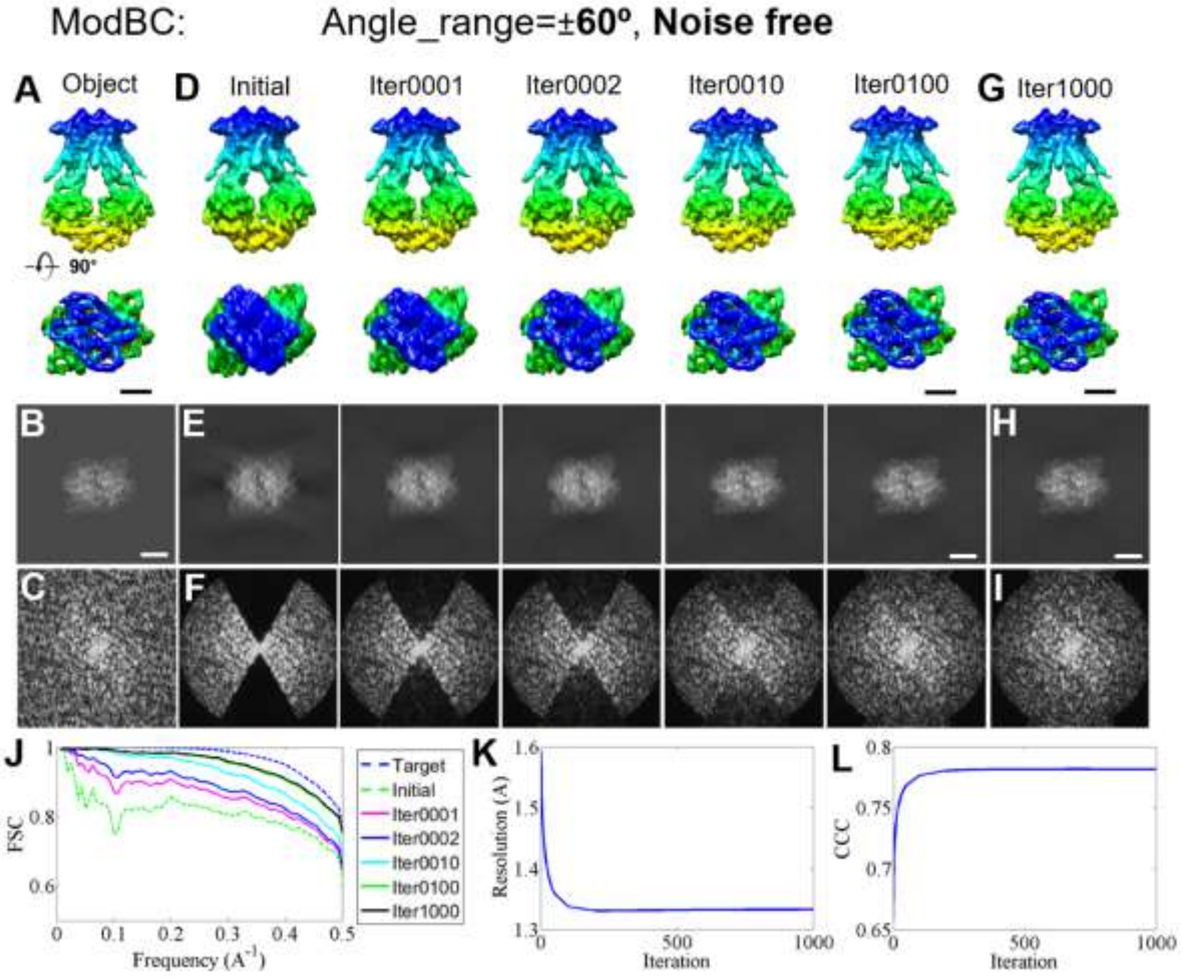

Supplementary Fig. 9 | The missing-wedge correction on a simulated 3D map of ModB$_2$C$_2$ reconstructed from the noise-free ±60° tilt series (A) A 3D object shown from two perpendicular views. The object was a generated from the crystal structure of ModB$_2$C$_2$, and the idea 3D was constructed from the tilt series of the noise-free 2D projections, which were generated by projecting the object from a series of tilt angles in a range of ±90° in steps of 1.5°, while the initial 3D was reconstructed from angles of ±60°. (**B**) The projection of the object on the X-Z plane, and (**C**) the corresponding Fourier transform. (**D**) The initial 3D and iterative 3D maps, shown from perpendicular views. (**E**) Their corresponding projections on the X-Z plane and the (**F**) Fourier transforms. The mask corresponding to ~3 times the molecular weight of ModB$_2$C$_2$ was generated from the low-passed filtered object (~40 Å). (**G**) The final corrected 3D after 1,000 cycles of iteration (round 1, Rd_1), shown from two perpendicular views, and (**H**) the projection along the X-Z plane and the corresponding (**I**) Fourier transform. (**J**) FSC curves of the iterative 3Ds against the object. The blue dashed line is the ideal FSC curve, calculated between the ideal 3D and the object. The dashed green line is the initial FSC, calculated between the initial 3D and the object. The rest solid lines

are the iterative FSC calculated between the object and 3Ds after 1 (in purple), 2 (in blue), 10 (in cyan), 100 (in green), and 1,000 (in black) cycles of iteration. (**K**) The plot of the iterative 3D resolutions against the cycles of iteration (the resolution is defined by the frequency at the FSC curve falls to 0.5). (**L**) The plot of the CCC (between the iterative 3D and the object) against the cycles of the iteration. All 3Ds were low-pass filtered to 8 Å. Bars: 20 nm.

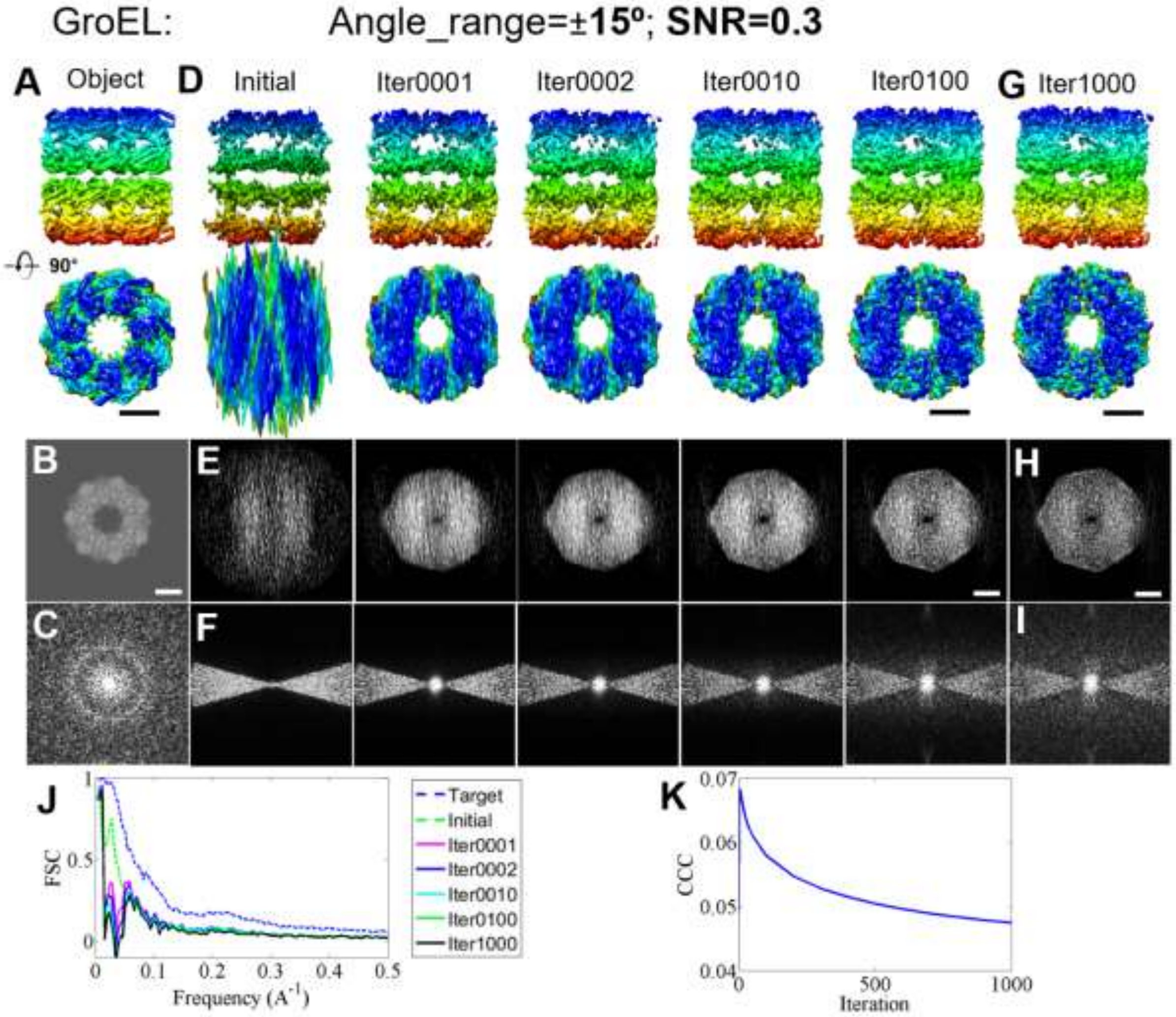

Supplementary Fig. 10 | The missing-wedge correction on a simulated 3D map of GroEL reconstructed from the noisy ±15° tilt series (SNR=0.3) (A) A 3D object shown from two perpendicular views. The object was a generated from the crystal structure of GroEL, and the idea 3D was constructed from the tilt series of the noisy 2D projections (SNR=0.3) of the object from tilt angles in a range of ±90° in steps of 1.5°, while the initial 3D was reconstructed from that within the angle range of ±15°. (**B**) The projection of the object on the X-Z plane, and (**C**) the corresponding Fourier transform. (**D**) The initial 3D and iterative 3D maps, shown from perpendicular views. (**E**) Their corresponding projections on the X-Z plane and the (**F**) Fourier transforms. The mask corresponding to ~3 times the molecular weight of GroEL was generated from the low-passed filtered object (~40 Å). (**G**) The final corrected 3D after 1,000 cycles of iteration (round 1, Rd_1), shown from two perpendicular views, and (**H**) the projection along the X-Z plane and the corresponding (**I**) Fourier transform. (**J**) FSC curves of the iterative 3Ds against the object.

The blue dashed line is the ideal FSC curve, calculated between the ideal 3D and the object. The dashed green line is the initial FSC, calculated between the initial 3D and the object. The rest solid lines are the iterative FSC calculated between the object and 3Ds after 1 (in purple), 2 (in blue), 10 (in cyan), 100 (in green), and 1,000 (in black) cycles of iteration. (**K**) The plot of the CCC (between the iterative 3D and the object) against the cycles of the iteration. All 3Ds were low-pass filtered to 8 Å. Bars: 50 nm.

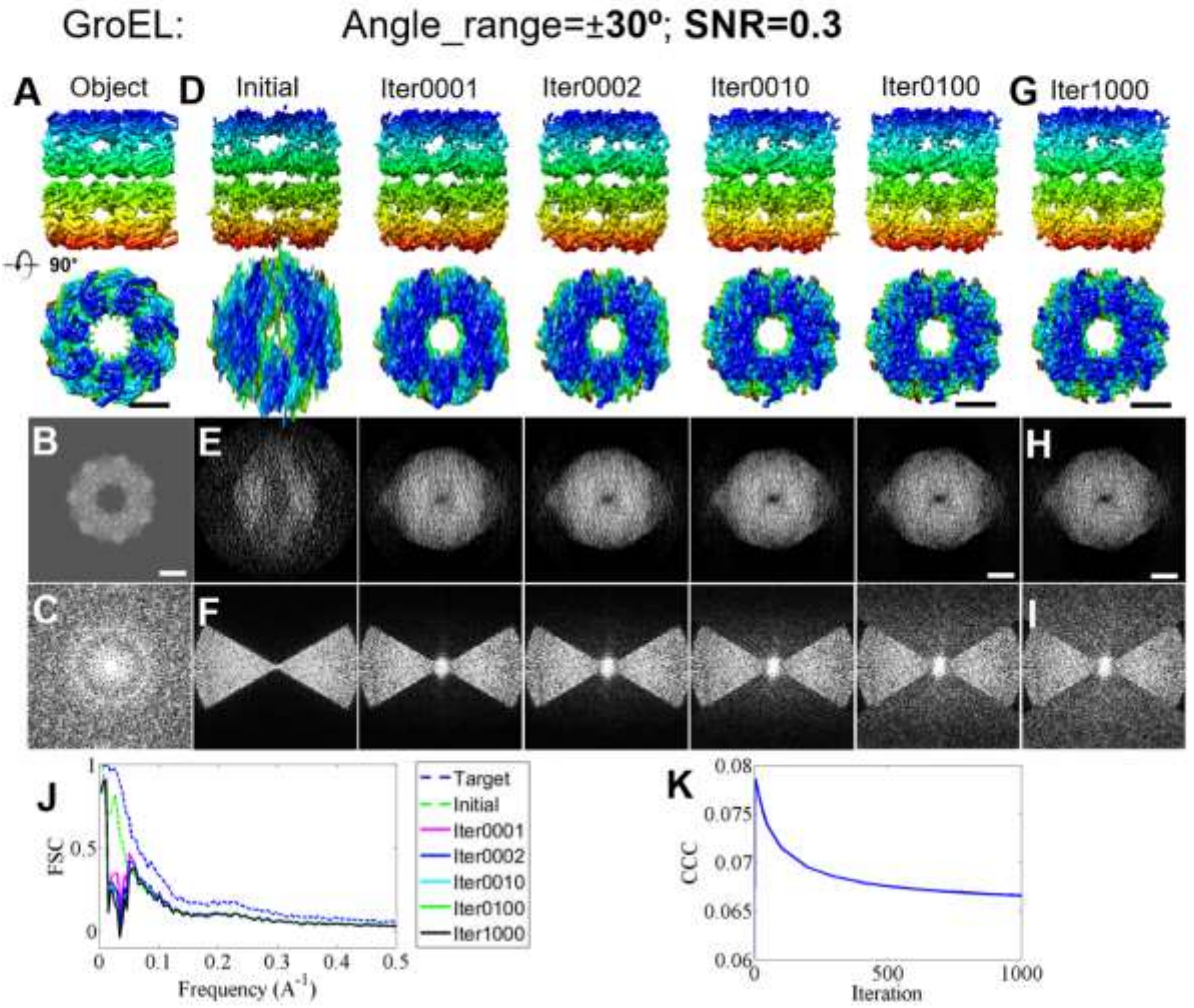

Supplementary Fig. 11 | The missing-wedge correction on a simulated 3D map of GroEL reconstructed from the noisy ±30° tilt series (SNR=0.3) (A) A 3D object shown from two perpendicular views. The object was a generated from the crystal structure of GroEL, and the idea 3D was constructed from the tilt series of the noisy 2D projections (SNR=0.3) of the object from tilt angles in a range of ±90° in steps of 1.5°, while the initial 3D was reconstructed from angles of ±30°. (**B**) The projection of the object on the X-Z plane, and (**C**) the corresponding Fourier transform. (**D**) The initial 3D and iterative 3D maps, shown from perpendicular views. (**E**) Their corresponding projections on the X-Z plane and the (**F**) Fourier transforms. The mask corresponding to ~3 times the molecular weight of GroEL was generated from the low-passed filtered object (~40 Å). (**G**) The final corrected 3D after 1,000 cycles of iteration (round 1, Rd_1), shown from two perpendicular views, and (**H**) the projection along the X-Z plane and the corresponding (**I**) Fourier transform. (**J**) FSC curves of the iterative 3Ds against the object. The blue dashed

line is the ideal FSC curve, calculated between the ideal 3D and the object. The dashed green line is the initial FSC, calculated between the initial 3D and the object. The rest solid lines are the iterative FSC calculated between the object and 3Ds after 1 (in purple), 2 (in blue), 10 (in cyan), 100 (in green), and 1,000 (in black) cycles of iteration. (**K**) The plot of the CCC (between the iterative 3D and the object) against the cycles of the iteration. All 3Ds were low-pass filtered to 8 Å. Bars: 50 nm.

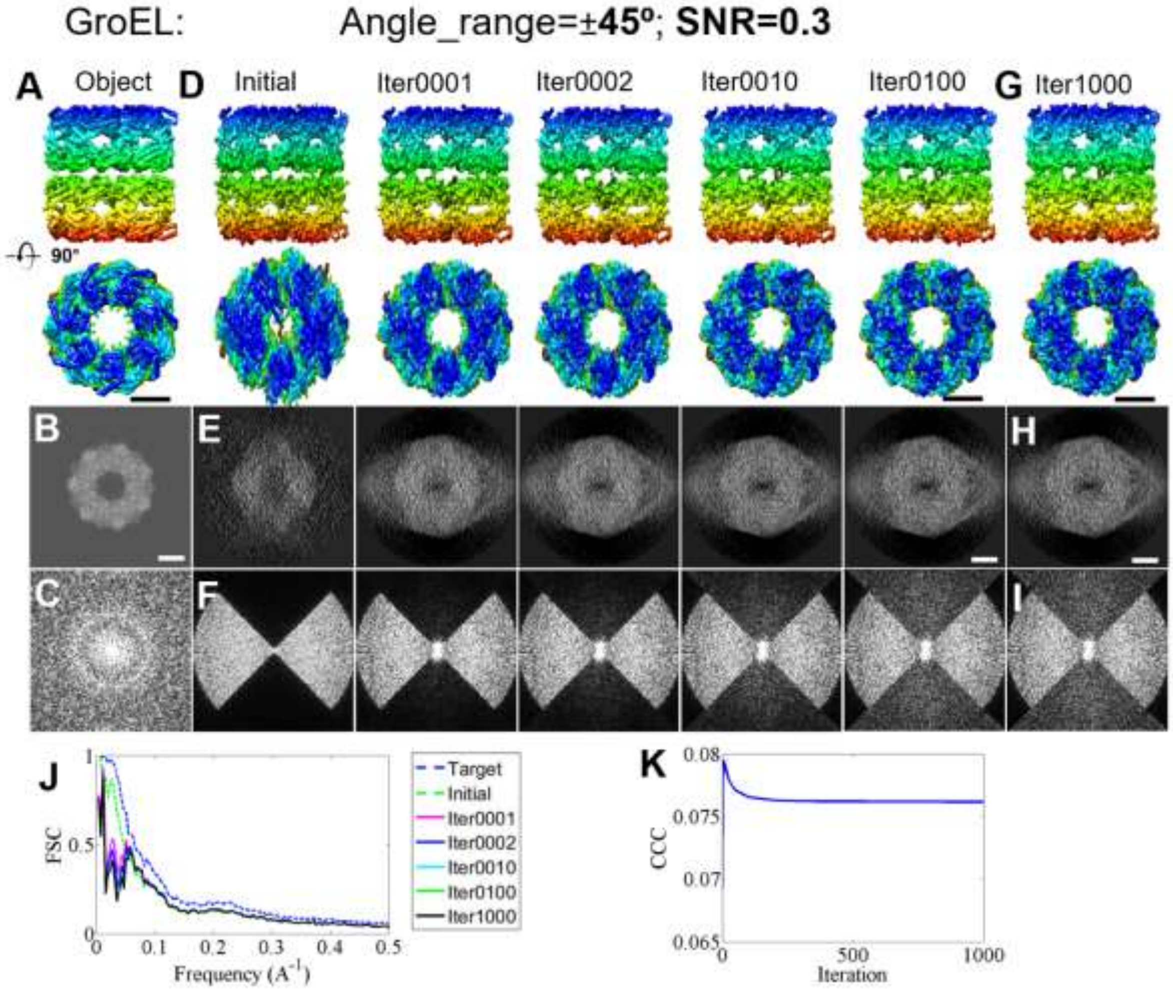

Supplementary Fig. 12 | The missing-wedge correction on a simulated 3D map of GroEL reconstructed from the noisy ±45° tilt series (SNR=0.3) (A) A 3D object shown from two perpendicular views. The object was a generated from the crystal structure of GroEL, and the idea 3D was constructed from the tilt series of the noisy 2D projections (SNR=0.3) of the object from tilt angles in a range of ±90° in steps of 1.5°, while the initial 3D was reconstructed from angles of ±45°. (**B**) The projection of the object on the X-Z plane, and (**C**) the corresponding Fourier transform. (**D**) The initial 3D and iterative 3D maps, shown from perpendicular views. (**E**) Their corresponding projections on the X-Z plane and the (**F**) Fourier transforms. The mask corresponding to ~3 times the molecular weight of GroEL was generated from the low-passed filtered object (~40 Å). (**G**) The final corrected 3D after 1,000 cycles of iteration (round 1, Rd_1), shown from two perpendicular views, and (**H**) the projection along the X-Z plane and the corresponding (**I**) Fourier transform. (**J**) FSC curves of the iterative 3Ds against the object. The blue dashed

line is the ideal FSC curve, calculated between the ideal 3D and the object. The dashed green line is the initial FSC, calculated between the initial 3D and the object. The rest solid lines are the iterative FSC calculated between the object and 3Ds after 1 (in purple), 2 (in blue), 10 (in cyan), 100 (in green), and 1,000 (in black) cycles of iteration. (**K**) The plot of the CCC (between the iterative 3D and the object) against the cycles of the iteration. All 3Ds were low-pass filtered to 8 Å. Bars: 50 nm.

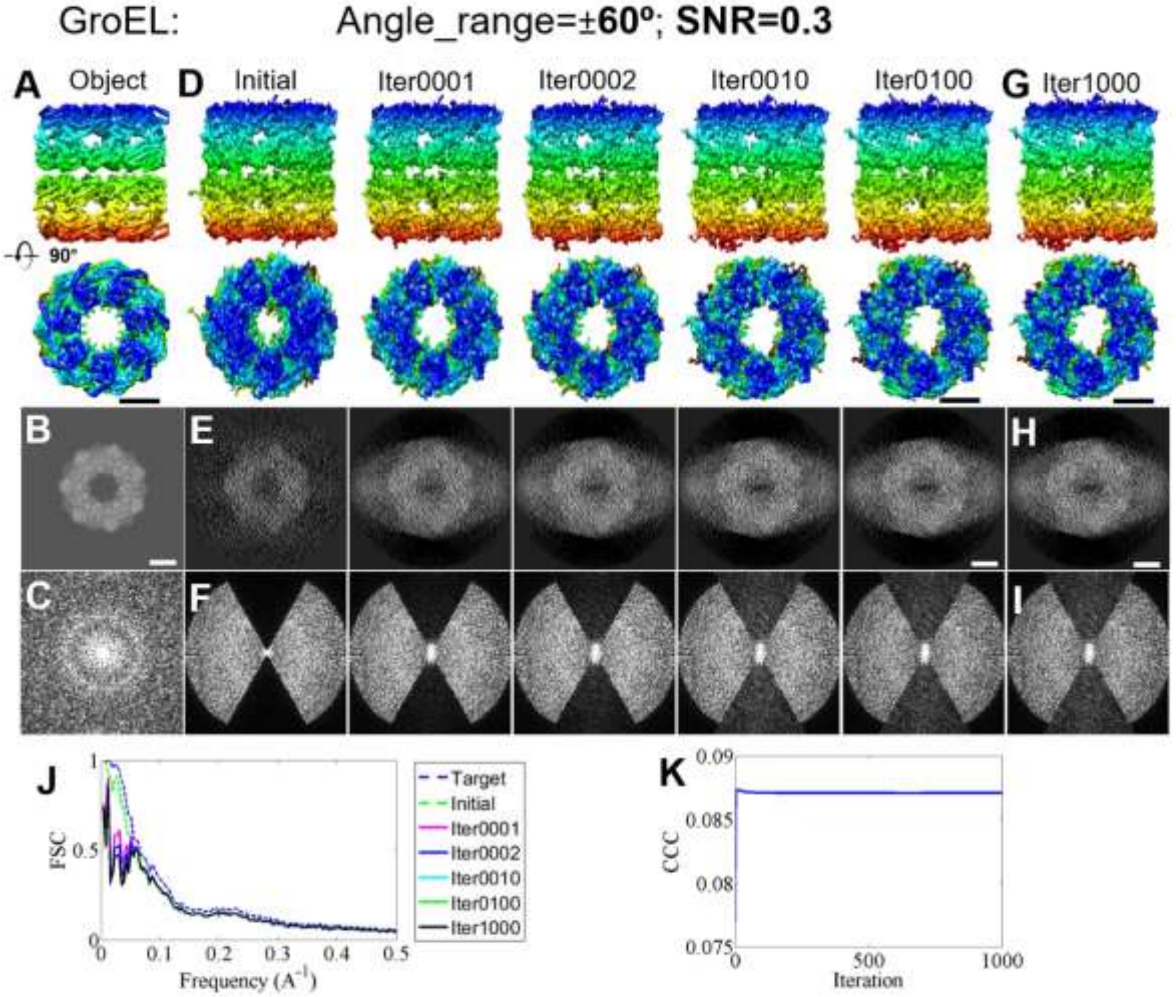

Supplementary Fig. 13 | The missing-wedge correction on a simulated 3D map of GroEL reconstructed from the noisy ±60° tilt series (SNR=0.3) (A) A 3D object shown from two perpendicular views. The object was a generated from the crystal structure of GroEL, and the idea 3D was constructed from the tilt series of the noisy 2D projections (SNR=0.3) of the object from tilt angles in a range of ±90° in steps of 1.5°, while the initial 3D was reconstructed from angles of ±60°. (**B**) The projection of the object on the X-Z plane, and (**C**) the corresponding Fourier transform. (**D**) The initial 3D and iterative 3D maps, shown from perpendicular views. (**E**) Their corresponding projections on the X-Z plane and the (**F**) Fourier transforms. The mask corresponding to ~3 times the molecular weight of GroEL was generated from the low-passed filtered object (~40 Å). (**G**) The final corrected 3D after 1,000 cycles of iteration (round 1, Rd_1), shown from two perpendicular views, and (**H**) the projection along the X-Z plane and the corresponding (**I**) Fourier transform. (**J**) FSC curves of the iterative 3Ds against the object. The blue dashed

line is the ideal FSC curve, calculated between the ideal 3D and the object. The dashed green line is the initial FSC, calculated between the initial 3D and the object. The rest solid lines are the iterative FSC calculated between the object and 3Ds after 1 (in purple), 2 (in blue), 10 (in cyan), 100 (in green), and 1,000 (in black) cycles of iteration. (**K**) The plot of the CCC (between the iterative 3D and the object) against the cycles of the iteration. All 3Ds were low-pass filtered to 8 Å. Bars: 50 nm.

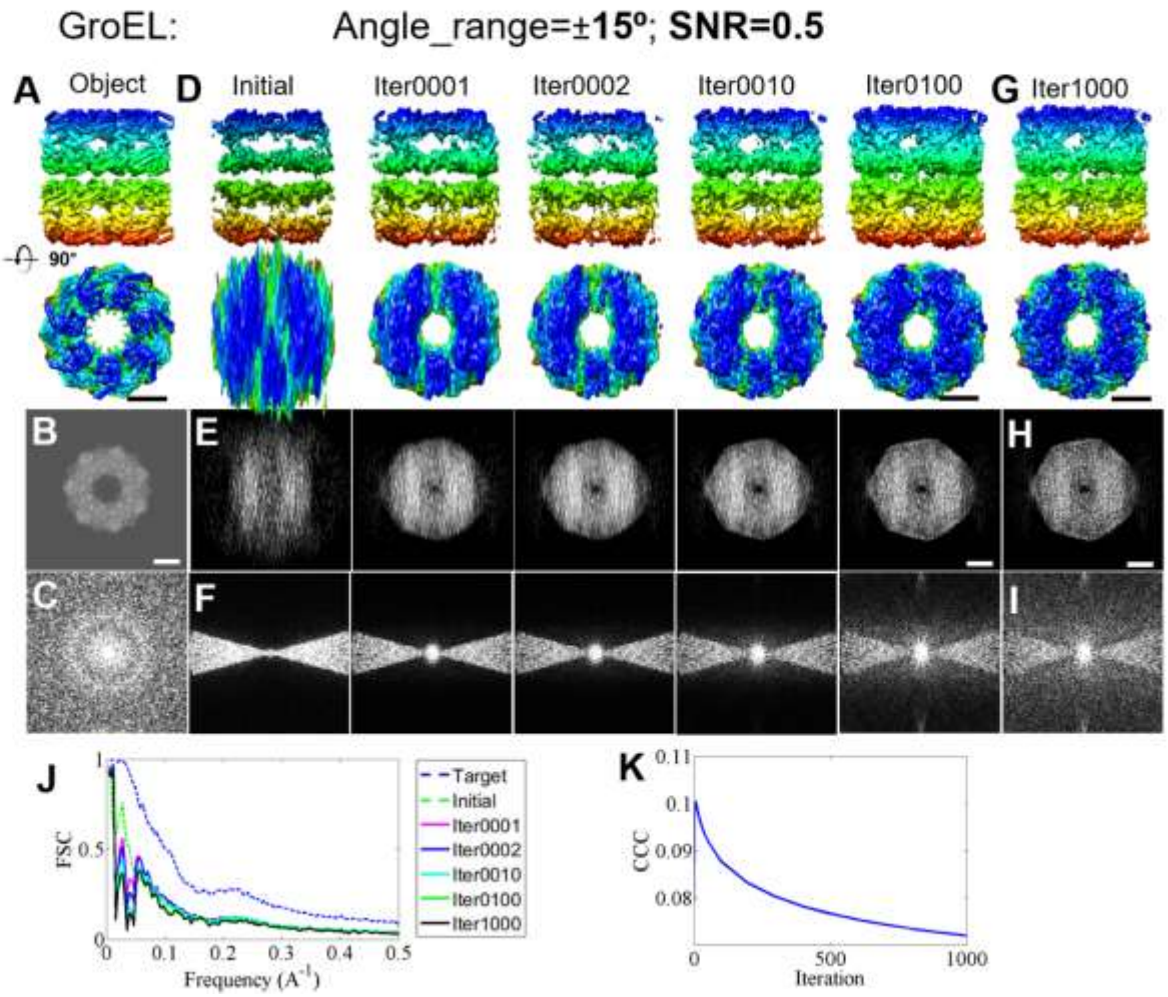

Supplementary Fig. 14 | The missing-wedge correction on a simulated 3D map of GroEL reconstructed from the noisy ±15° tilt series (SNR=0.5) (A) A 3D object shown from two perpendicular views. The object was a generated from the crystal structure of GroEL, and the idea 3D was constructed from the tilt series of the noisy 2D projections (SNR=0.5) of the object from tilt angles in a range of ±90° in steps of 1.5°, while the initial 3D was reconstructed from that within the angle range of ±15°. (**B**) The projection of the object on the X-Z plane, and (**C**) the corresponding Fourier transform. (**D**) The initial 3D and iterative 3D maps, shown from perpendicular views. (**E**) Their corresponding projections on the X-Z plane and the (**F**) Fourier transforms. The mask corresponding to ~3 times the molecular weight of GroEL was generated from the low-passed filtered object (~40 Å). (**G**) The final corrected 3D after 1,000 cycles of iteration (round 1, Rd_1), shown from two perpendicular views, and (**H**) the projection along the X-Z plane and the corresponding (**I**) Fourier transform. (**J**) FSC curves of the iterative 3Ds against the object.

The blue dashed line is the ideal FSC curve, calculated between the ideal 3D and the object. The dashed green line is the initial FSC, calculated between the initial 3D and the object. The rest solid lines are the iterative FSC calculated between the object and 3Ds after 1 (in purple), 2 (in blue), 10 (in cyan), 100 (in green), and 1,000 (in black) cycles of iteration. (**K**) The plot of the CCC (between the iterative 3D and the object) against the cycles of the iteration. All 3Ds were low-pass filtered to 8 Å. Bars: 50 nm.

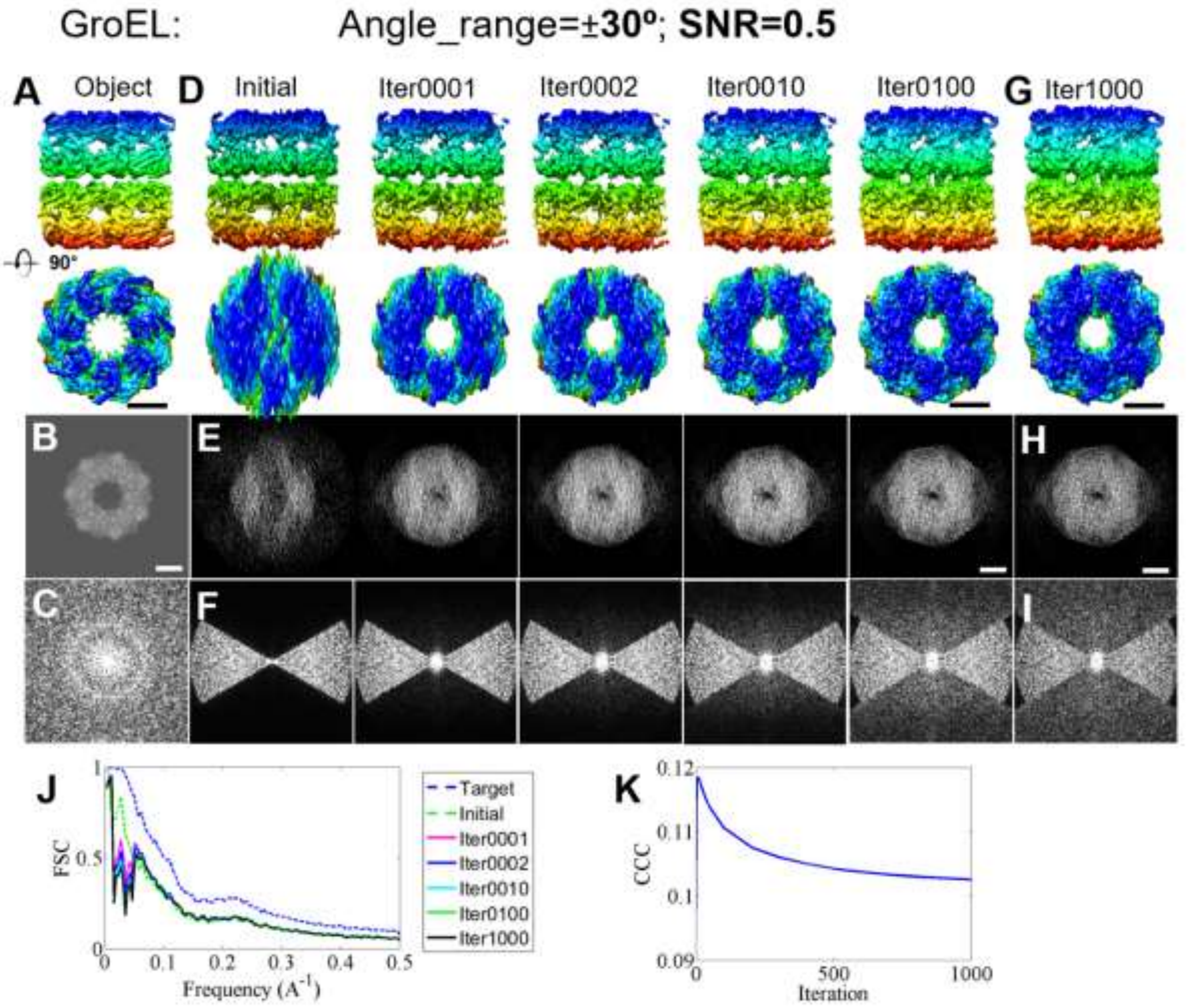

Supplementary Fig. 15 | The missing-wedge correction on a simulated 3D map of GroEL reconstructed from the noisy ±30° tilt series (SNR=0.5) (A) A 3D object shown from two perpendicular views. The object was a generated from the crystal structure of GroEL, and the idea 3D was constructed from the tilt series of the noisy 2D projections (SNR=0.5) of the object from tilt angles in a range of ±90° in steps of 1.5°, while the initial 3D was reconstructed from angles of ±30°. (**B**) The projection of the object on the X-Z plane, and (**C**) the corresponding Fourier transform. (**D**) The initial 3D and iterative 3D maps, shown from perpendicular views. (**E**) Their corresponding projections on the X-Z plane and the (**F**) Fourier transforms. The mask corresponding to ~3 times the molecular weight of GroEL was generated from the low-passed filtered object (~40 Å). (**G**) The final corrected 3D after 1,000 cycles of iteration (round 1, Rd_1), shown from two perpendicular views, and (**H**) the projection along the X-Z plane and the corresponding (**I**) Fourier transform. (**J**) FSC curves of the iterative 3Ds against the object. The blue dashed

line is the ideal FSC curve, calculated between the ideal 3D and the object. The dashed green line is the initial FSC, calculated between the initial 3D and the object. The rest solid lines are the iterative FSC calculated between the object and 3Ds after 1 (in purple), 2 (in blue), 10 (in cyan), 100 (in green), and 1,000 (in black) cycles of iteration. (**K**) The plot of the CCC (between the iterative 3D and the object) against the cycles of the iteration. All 3Ds were low-pass filtered to 8 Å. Bars: 50 nm.

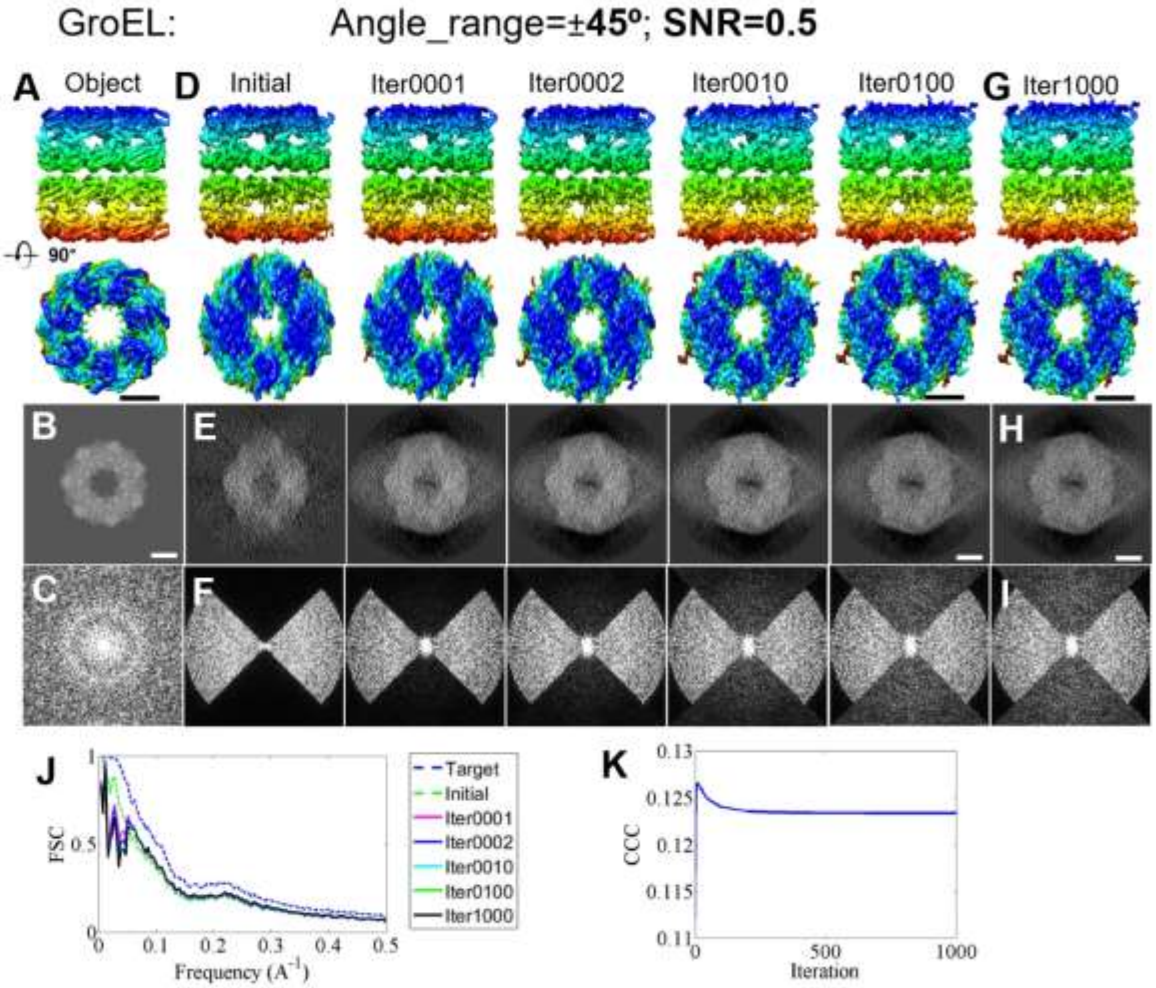

Supplementary Fig. 16 | The missing-wedge correction on a simulated 3D map of GroEL reconstructed from the noisy ±45° tilt series (SNR=0.3) (A) A 3D object shown from two perpendicular views. The object was a generated from the crystal structure of GroEL, and the idea 3D was constructed from the tilt series of the noisy 2D projections (SNR=0.5) of the object from tilt angles in a range of ±90° in steps of 1.5°, while the initial 3D was reconstructed from angles of ±45°. (**B**) The projection of the object on the X-Z plane, and (**C**) the corresponding Fourier transform. (**D**) The initial 3D and iterative 3D maps, shown from perpendicular views. (**E**) Their corresponding projections on the X-Z plane and the (**F**) Fourier transforms. The mask corresponding to ~3 times the molecular weight of GroEL was generated from the low-passed filtered object (~40 Å). (**G**) The final corrected 3D after 1,000 cycles of iteration (round 1, Rd_1), shown from two perpendicular views, and (**H**) the projection along the X-Z plane and the corresponding (**I**) Fourier transform. (**J**) FSC curves of the iterative 3Ds against the object. The blue dashed line is the ideal FSC curve, calculated between the ideal 3D and the object. The dashed green line is the

initial FSC, calculated between the initial 3D and the object. The rest solid lines are the iterative FSC calculated between the object and 3Ds after 1 (in purple), 2 (in blue), 10 (in cyan), 100 (in green), and 1,000 (in black) cycles of iteration. (**K**) The plot of the CCC (between the iterative 3D and the object) against the cycles of the iteration. All 3Ds were low-pass filtered to 8 Å. Bars: 50 nm.

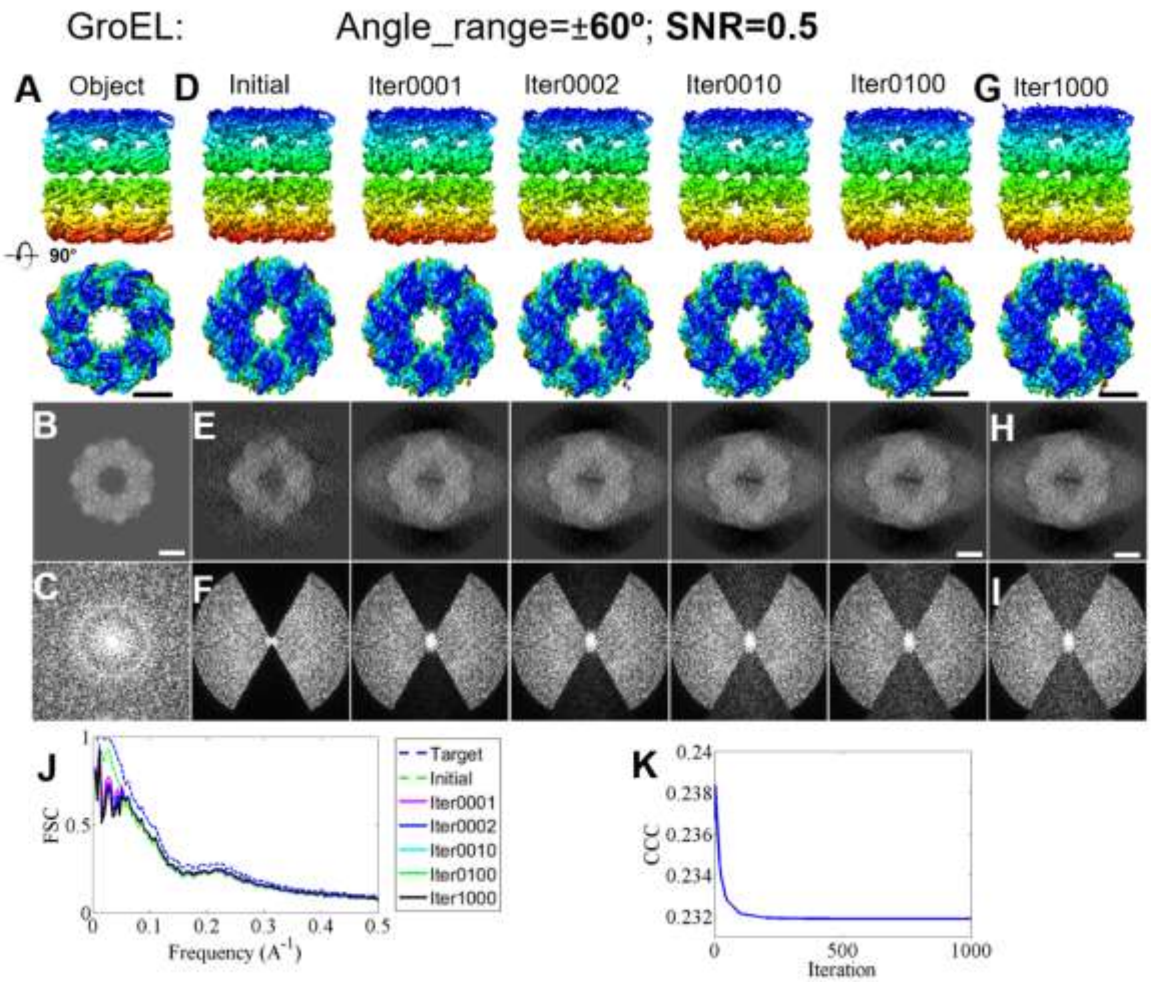

Supplementary Fig. 17 | The missing-wedge correction on a simulated 3D map of GroEL reconstructed from the noisy ±60° tilt series (SNR=0.5) (A) A 3D object shown from two perpendicular views. The object was a generated from the crystal structure of GroEL, and the idea 3D was constructed from the tilt series of the noisy 2D projections (SNR=0.5) of the object from tilt angles in a range of ±90° in steps of 1.5°, while the initial 3D was reconstructed from angles of ±60°. (**B**) The projection of the object on the X-Z plane, and (**C**) the corresponding Fourier transform. (**D**) The initial 3D and iterative 3D maps, shown from perpendicular views. (**E**) Their corresponding projections on the X-Z plane and the (**F**) Fourier transforms. The mask corresponding to ~3 times the molecular weight of GroEL was generated from the low-passed filtered object (~40 Å). (**G**) The final corrected 3D after 1,000 cycles of iteration (round 1, Rd_1), shown from two perpendicular views, and (**H**) the projection along the X-Z plane and the corresponding (**I**) Fourier transform. (**J**) FSC curves of the iterative 3Ds against the object. The blue dashed line is the ideal FSC curve, calculated between the ideal 3D and the object. The dashed green line is the

initial FSC, calculated between the initial 3D and the object. The rest solid lines are the iterative FSC calculated between the object and 3Ds after 1 (in purple), 2 (in blue), 10 (in cyan), 100 (in green), and 1,000 (in black) cycles of iteration. (**K**) The plot of the CCC (between the iterative 3D and the object) against the cycles of the iteration. All 3Ds were low-pass filtered to 8 Å. Bars: 50 nm.

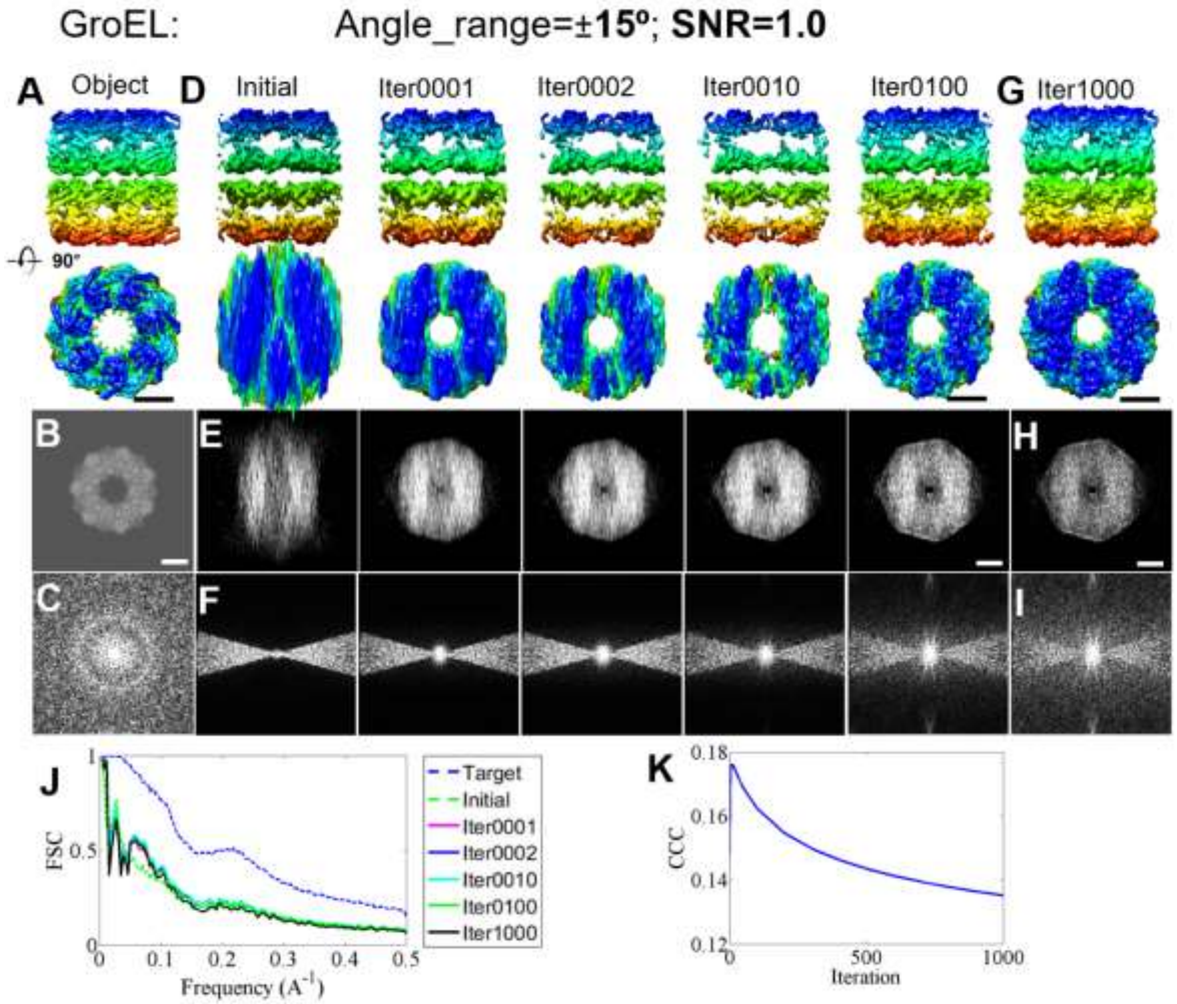

Supplementary Fig. 18 | The missing-wedge correction on a simulated 3D map of GroEL reconstructed from the noisy ±15° tilt series (SNR=1.0) (A) A 3D object shown from two perpendicular views. The object was a generated from the crystal structure of GroEL, and the idea 3D was constructed from the tilt series of the noisy 2D projections (SNR=1.0) of the object from tilt angles in a range of ±90° in steps of 1.5°, while the initial 3D was reconstructed from that within the angle range of ±15°. (**B**) The projection of the object on the X-Z plane, and (**C**) the corresponding Fourier transform. (**D**) The initial 3D and iterative 3D maps, shown from perpendicular views. (**E**) Their corresponding projections on the X-Z plane and the (**F**) Fourier transforms. The mask corresponding to ~3 times the molecular weight of GroEL was generated from the low-passed filtered object (~40 Å). (**G**) The final corrected 3D after 1,000 cycles of iteration (round 1, Rd_1), shown from two perpendicular views, and (**H**) the projection along the X-Z plane and the corresponding (**I**) Fourier transform. (**J**) FSC curves of the iterative 3Ds against the object.

The blue dashed line is the ideal FSC curve, calculated between the ideal 3D and the object. The dashed green line is the initial FSC, calculated between the initial 3D and the object. The rest solid lines are the iterative FSC calculated between the object and 3Ds after 1 (in purple), 2 (in blue), 10 (in cyan), 100 (in green), and 1,000 (in black) cycles of iteration. (**K**) The plot of the CCC (between the iterative 3D and the object) against the cycles of the iteration. All 3Ds were low-pass filtered to 8 Å. Bars: 50 nm.

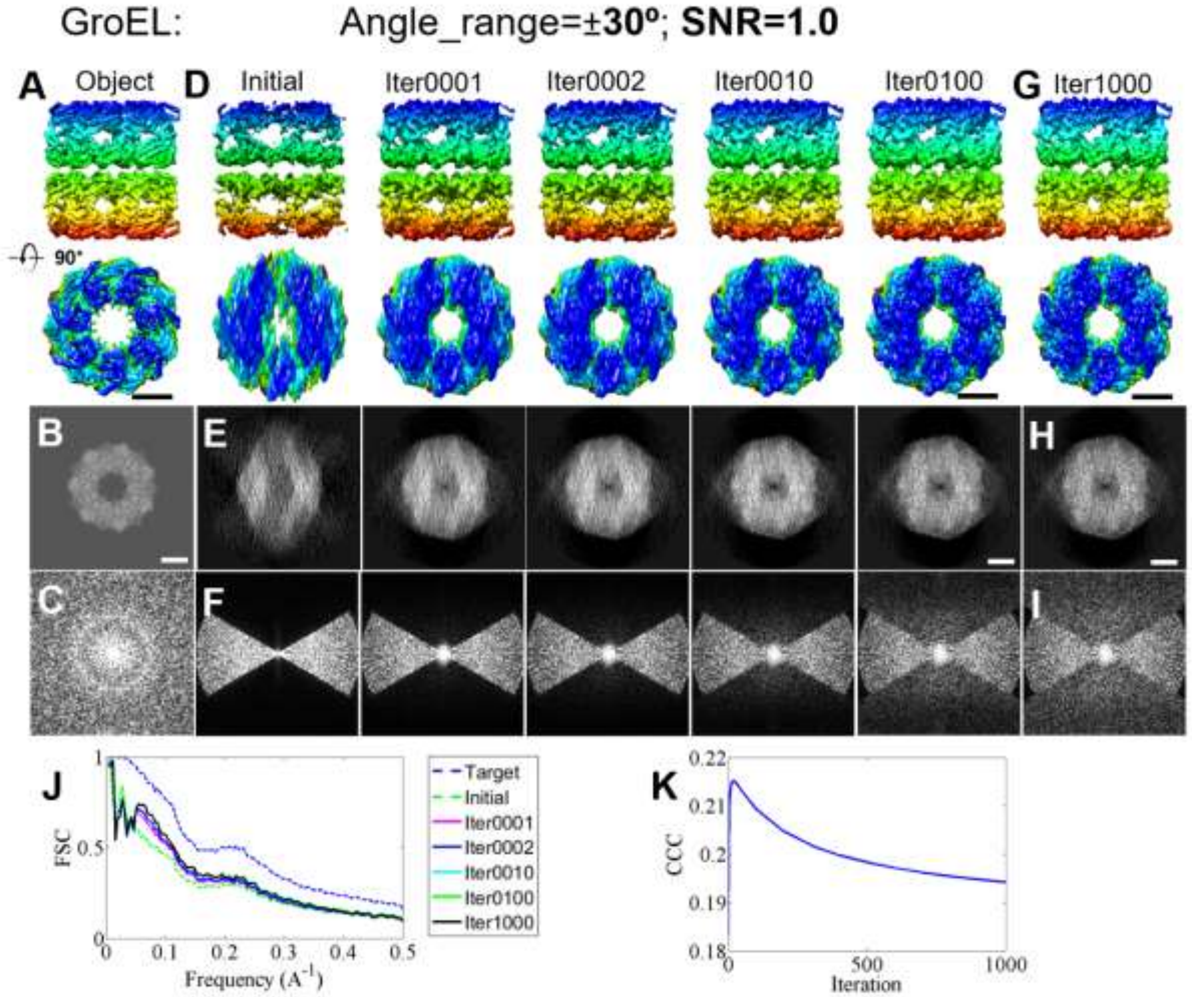

Supplementary Fig. 19 | The missing-wedge correction on a simulated 3D map of GroEL reconstructed from the noisy ±30° tilt series (SNR=1.0) (A) A 3D object shown from two perpendicular views. The object was a generated from the crystal structure of GroEL, and the idea 3D was constructed from the tilt series of the noisy 2D projections (SNR=1.0) of the object from tilt angles in a range of ±90° in steps of 1.5°, while the initial 3D was reconstructed from angles of ±30°. (**B**) The projection of the object on the X-Z plane, and (**C**) the corresponding Fourier transform. (**D**) The initial 3D and iterative 3D maps, shown from perpendicular views. (**E**) Their corresponding projections on the X-Z plane and the (**F**) Fourier transforms. The mask corresponding to ~3 times the molecular weight of GroEL was generated from the low-passed filtered object (~40 Å). (**G**) The final corrected 3D after 1,000 cycles of iteration (round 1, Rd_1), shown from two perpendicular views, and (**H**) the projection along the X-Z plane and the corresponding (**I**) Fourier transform. (**J**) FSC curves of the iterative 3Ds against the object. The blue dashed

line is the ideal FSC curve, calculated between the ideal 3D and the object. The dashed green line is the initial FSC, calculated between the initial 3D and the object. The rest solid lines are the iterative FSC calculated between the object and 3Ds after 1 (in purple), 2 (in blue), 10 (in cyan), 100 (in green), and 1,000 (in black) cycles of iteration. (**K**) The plot of the CCC (between the iterative 3D and the object) against the cycles of the iteration. All 3Ds were low-pass filtered to 8 Å. Bars: 50 nm.

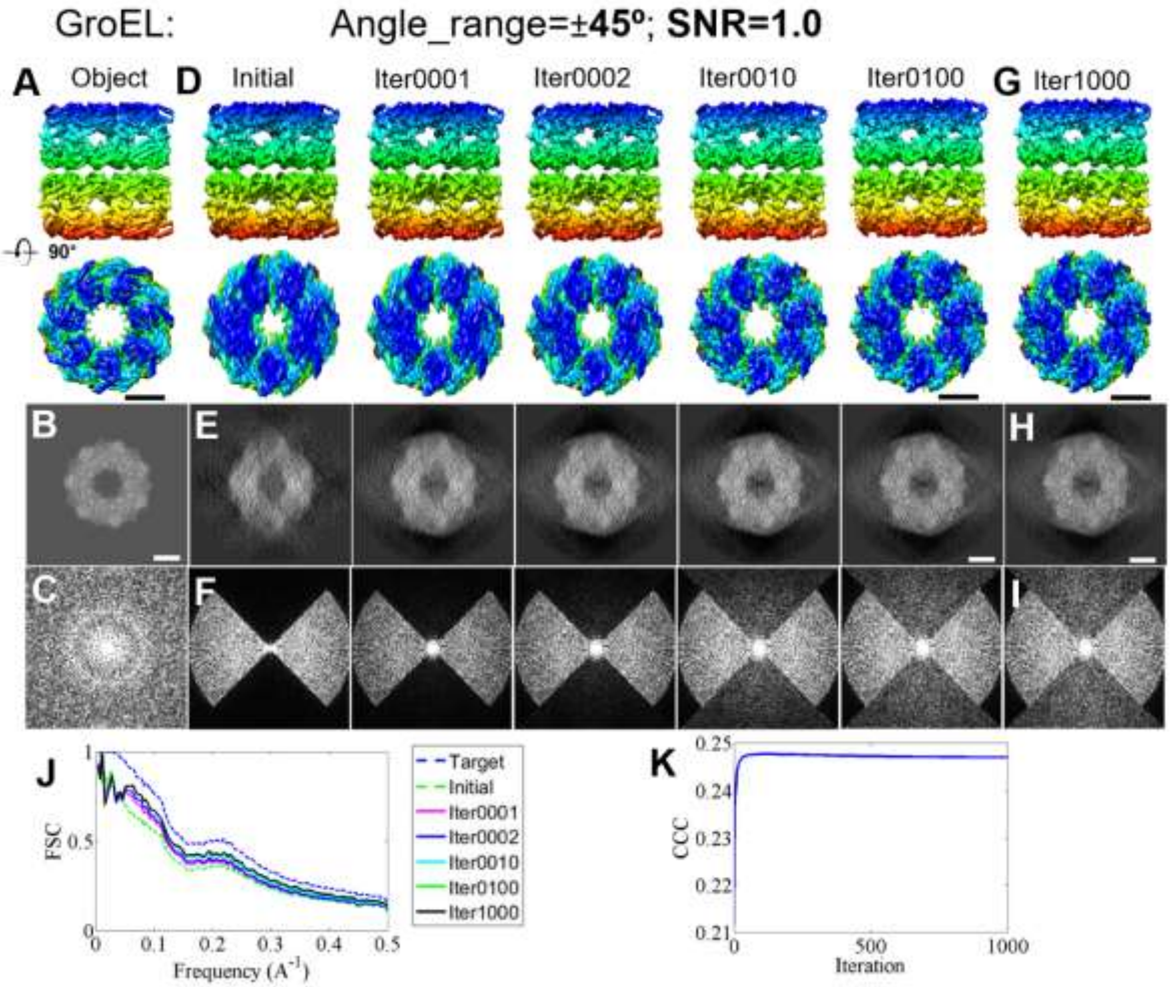

Supplementary Fig. 20 | The missing-wedge correction on a simulated 3D map of GroEL reconstructed from the noisy ±45° tilt series (SNR=1.0) (A) A 3D object shown from two perpendicular views. The object was a generated from the crystal structure of GroEL, and the idea 3D was constructed from the tilt series of the noisy 2D projections (SNR=1.0) of the object from tilt angles in a range of ±90° in steps of 1.5°, while the initial 3D was reconstructed from angles of ±45°. (**B**) The projection of the object on the X-Z plane, and (**C**) the corresponding Fourier transform. (**D**) The initial 3D and iterative 3D maps, shown from perpendicular views. (**E**) Their corresponding projections on the X-Z plane and the (**F**) Fourier transforms. The mask corresponding to ~3 times the molecular weight of GroEL was generated from the low-passed filtered object (~40 Å). (**G**) The final corrected 3D after 1,000 cycles of iteration (round 1, Rd_1), shown from two perpendicular views, and (**H**) the projection along the X-Z plane and the corresponding (**I**) Fourier transform. (**J**) FSC curves of the iterative 3Ds against the object. The blue dashed

line is the ideal FSC curve, calculated between the ideal 3D and the object. The dashed green line is the initial FSC, calculated between the initial 3D and the object. The rest solid lines are the iterative FSC calculated between the object and 3Ds after 1 (in purple), 2 (in blue), 10 (in cyan), 100 (in green), and 1,000 (in black) cycles of iteration. (**K**) The plot of the CCC (between the iterative 3D and the object) against the cycles of the iteration. All 3Ds were low-pass filtered to 8 Å. Bars: 50 nm.

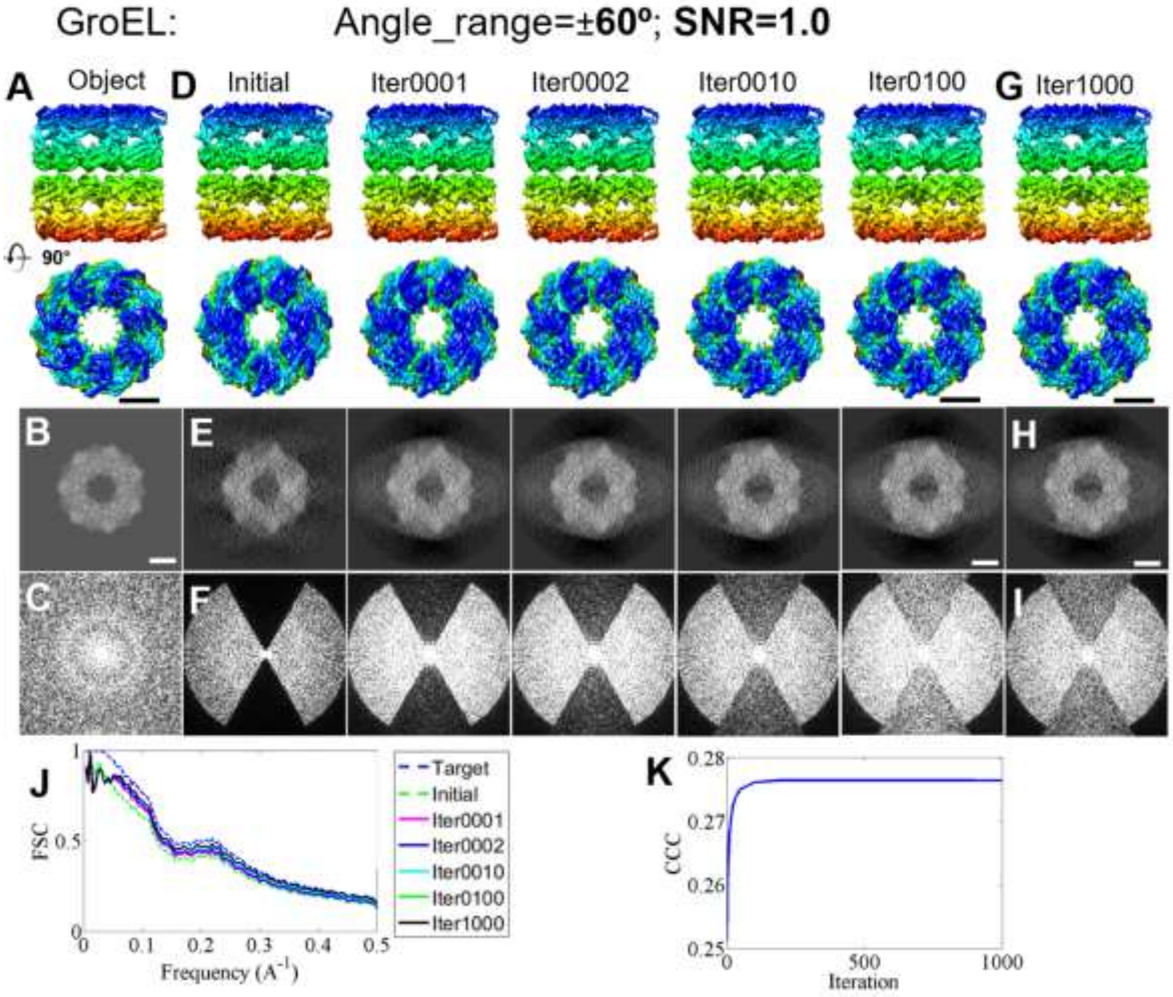

Supplementary Fig. 21 | The missing-wedge correction on a simulated 3D map of GroEL reconstructed from the noisy ±60° tilt series (SNR=1.0) (A) A 3D object shown from two perpendicular views. The object was a generated from the crystal structure of GroEL, and the idea 3D was constructed from the tilt series of the noisy 2D projections (SNR=1.0) of the object from tilt angles in a range of ±90° in steps of 1.5°, while the initial 3D was reconstructed from angles of ±60°. (**B**) The projection of the object on the X-Z plane, and (**C**) the corresponding Fourier transform. (**D**) The initial 3D and iterative 3D maps, shown from perpendicular views. (**E**) Their corresponding projections on the X-Z plane and the (**F**) Fourier transforms. The mask corresponding to ~3 times the molecular weight of GroEL was generated from the low-passed filtered object (~40 Å). (**G**) The final corrected 3D after 1,000 cycles of iteration (round 1, Rd_1), shown from two perpendicular views, and (**H**) the projection along the X-Z plane and the corresponding (**I**) Fourier transform. (**J**) FSC curves of the iterative 3Ds against the object. The blue dashed

line is the ideal FSC curve, calculated between the ideal 3D and the object. The dashed green line is the initial FSC, calculated between the initial 3D and the object. The rest solid lines are the iterative FSC calculated between the object and 3Ds after 1 (in purple), 2 (in blue), 10 (in cyan), 100 (in green), and 1,000 (in black) cycles of iteration. (**K**) The plot of the CCC (between the iterative 3D and the object) against the cycles of the iteration. All 3Ds were low-pass filtered to 8 Å. Bars: 50 nm.

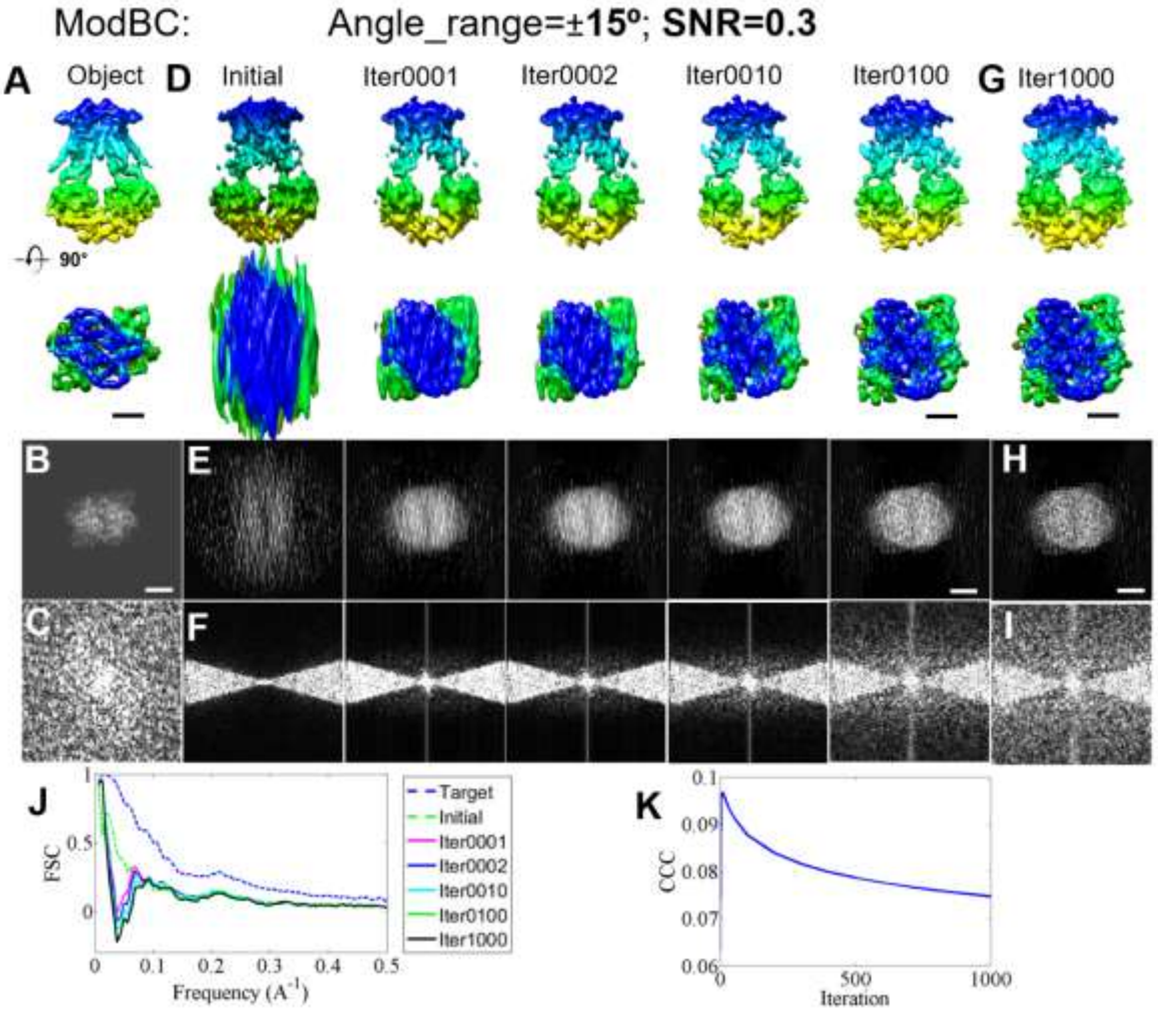

Supplementary Fig. 22 | The missing-wedge correction on a simulated 3D map of $ModB_2C_2$ reconstructed from the noisy ±15° tilt series (SNR=0.3) (A) A 3D object shown from two perpendicular views. The object was a generated from the crystal structure of $ModB_2C_2$, and the idea 3D was constructed from the tilt series of the noisy 2D projections (SNR=0.3) of the object from tilt angles in a range of ±90° in steps of 1.5°, while the initial 3D was reconstructed from that within the angle range of ±15°. (**B**) The projection of the object on the X-Z plane, and (**C**) the corresponding Fourier transform. (**D**) The initial 3D and iterative 3D maps, shown from perpendicular views. (**E**) Their corresponding projections on the X-Z plane and the (**F**) Fourier transforms. The mask corresponding to ~3 times the molecular weight of $ModB_2C_2$ was generated from the low-passed filtered object (~40 Å). (**G**) The final corrected 3D after 1,000 cycles of iteration (round 1, Rd_1), shown from two perpendicular views, and (**H**) the projection along the X-Z plane and the corresponding (**I**) Fourier transform. (**J**) FSC curves of the iterative 3Ds against the

object. The blue dashed line is the ideal FSC curve, calculated between the ideal 3D and the object. The dashed green line is the initial FSC, calculated between the initial 3D and the object. The rest solid lines are the iterative FSC calculated between the object and 3Ds after 1 (in purple), 2 (in blue), 10 (in cyan), 100 (in green), and 1,000 (in black) cycles of iteration. (**K**) The plot of the CCC (between the iterative 3D and the object) against the cycles of the iteration. All 3Ds were low-pass filtered to 8 Å. Bars: 20 nm.

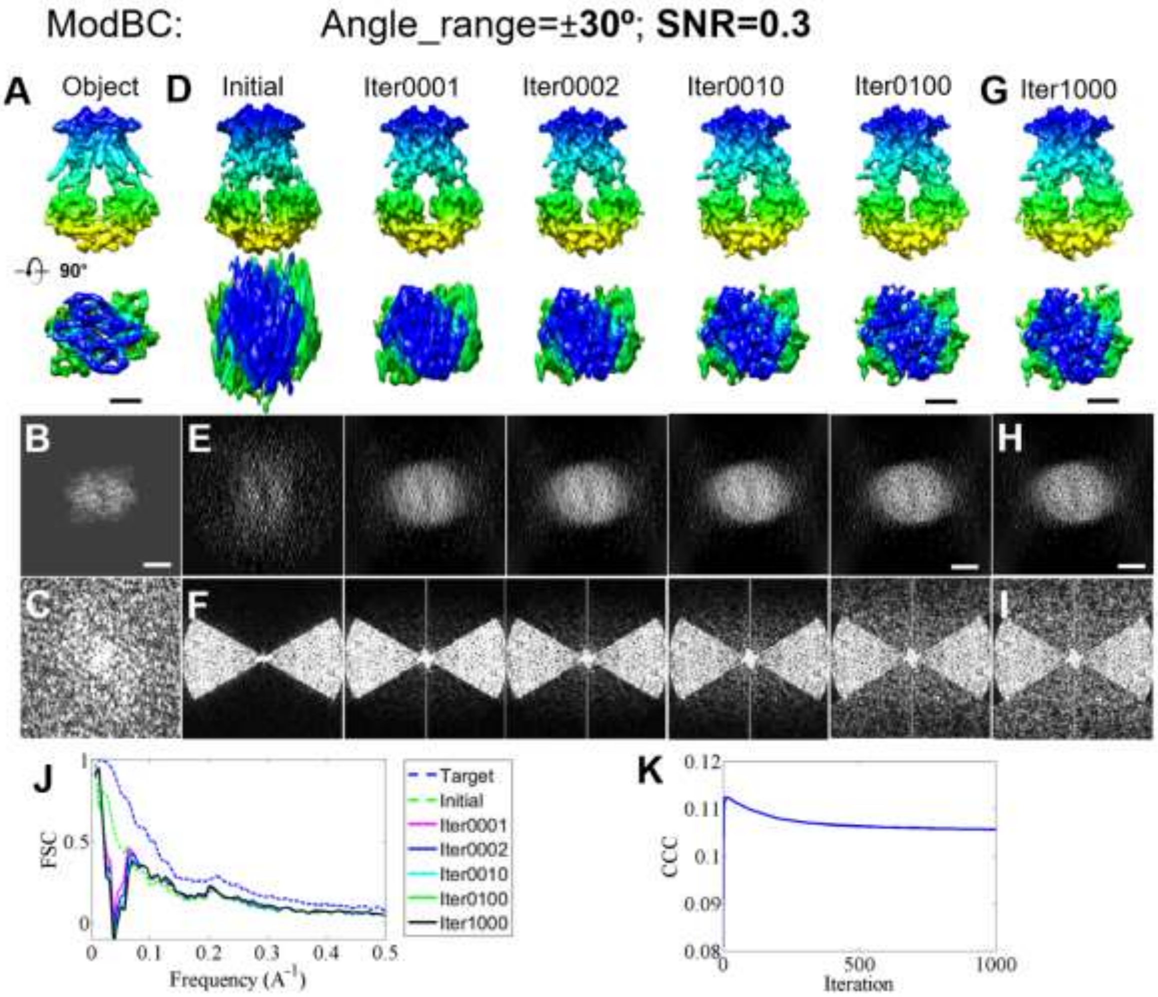

Supplementary Fig. 23 | The missing-wedge correction on a simulated 3D map of ModB$_2$C$_2$ reconstructed from the noisy ±30° tilt series (SNR=0.3) (A) A 3D object shown from two perpendicular views. The object was a generated from the crystal structure of ModB$_2$C$_2$, and the idea 3D was constructed from the tilt series of the noisy 2D projections (SNR=0.3) of the object from tilt angles in a range of ±90° in steps of 1.5°, while the initial 3D was reconstructed from angles of ±30°. (**B**) The projection of the object on the X-Z plane, and (**C**) the corresponding Fourier transform. (**D**) The initial 3D and iterative 3D maps, shown from perpendicular views. (**E**) Their corresponding projections on the X-Z plane and the (**F**) Fourier transforms. The mask corresponding to ~3 times the molecular weight of ModB$_2$C$_2$ was generated from the low-passed filtered object (~40 Å). (**G**) The final corrected 3D after 1,000 cycles of iteration (round 1, Rd_1), shown from two perpendicular views, and (**H**) the projection along the X-Z plane and the corresponding (**I**) Fourier transform. (**J**) FSC curves of the iterative 3Ds against the object. The blue dashed

line is the ideal FSC curve, calculated between the ideal 3D and the object. The dashed green line is the initial FSC, calculated between the initial 3D and the object. The rest solid lines are the iterative FSC calculated between the object and 3Ds after 1 (in purple), 2 (in blue), 10 (in cyan), 100 (in green), and 1,000 (in black) cycles of iteration. (**K**) The plot of the CCC (between the iterative 3D and the object) against the cycles of the iteration. All 3Ds were low-pass filtered to 8 Å. Bars: 20 nm.

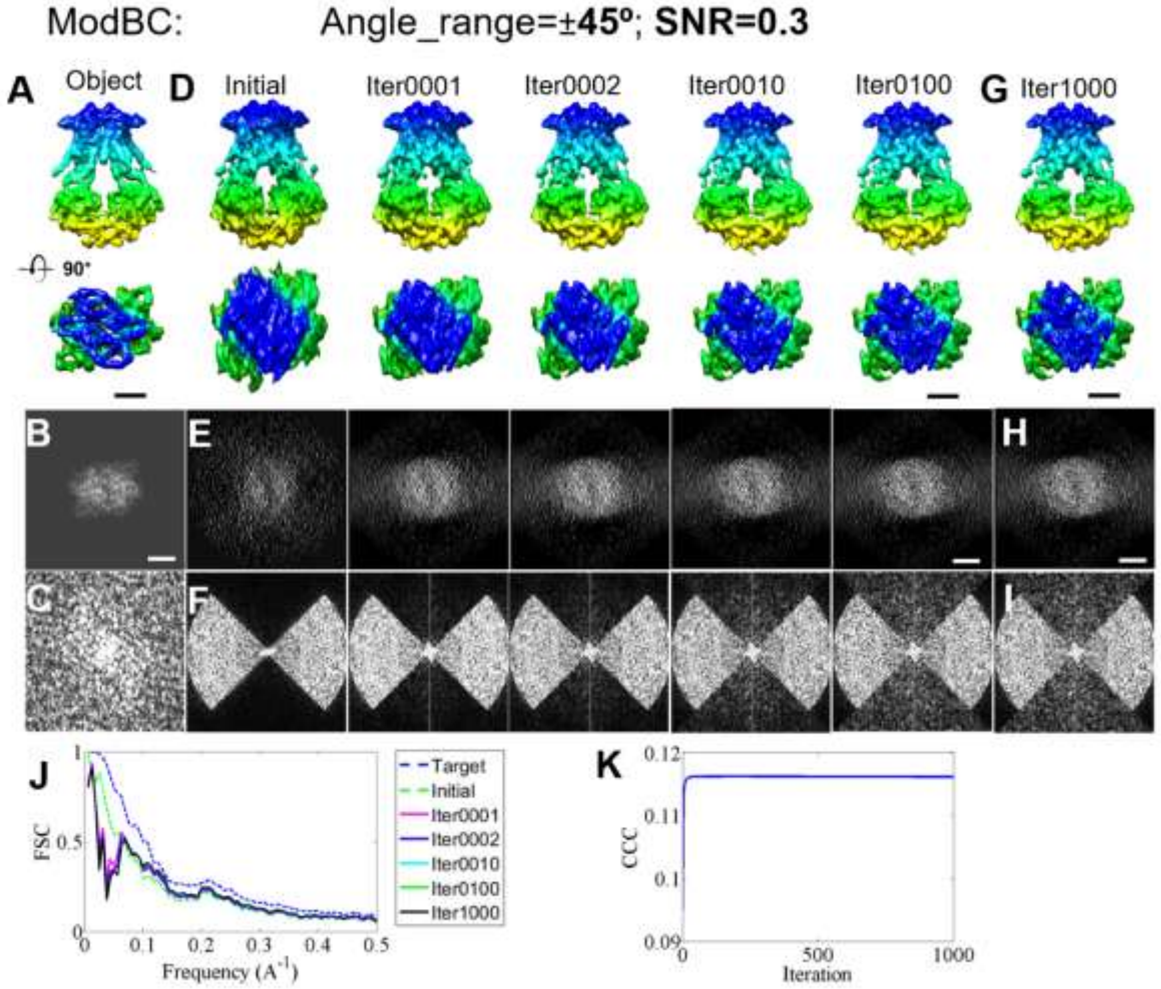

Supplementary Fig. 24 | The missing-wedge correction on a simulated 3D map of $ModB_2C_2$ reconstructed from the noisy ±45° tilt series (SNR=0.3) (A) A 3D object shown from two perpendicular views. The object was a generated from the crystal structure of $ModB_2C_2$, and the idea 3D was constructed from the tilt series of the noisy 2D projections (SNR=0.3) of the object from tilt angles in a range of ±90° in steps of 1.5°, while the initial 3D was reconstructed from angles of ±45°. (**B**) The projection of the object on the X-Z plane, and (**C**) the corresponding Fourier transform. (**D**) The initial 3D and iterative 3D maps, shown from perpendicular views. (**E**) Their corresponding projections on the X-Z plane and the (**F**) Fourier transforms. The mask corresponding to ~3 times the molecular weight of $ModB_2C_2$ was generated from the low-passed filtered object (~40 Å). (**G**) The final corrected 3D after 1,000 cycles of iteration (round 1, Rd_1), shown from two perpendicular views, and (**H**) the projection along the X-Z plane and the corresponding (**I**) Fourier transform. (**J**) FSC curves of the iterative 3Ds against the object. The blue dashed

line is the ideal FSC curve, calculated between the ideal 3D and the object. The dashed green line is the initial FSC, calculated between the initial 3D and the object. The rest solid lines are the iterative FSC calculated between the object and 3Ds after 1 (in purple), 2 (in blue), 10 (in cyan), 100 (in green), and 1,000 (in black) cycles of iteration. (**K**) The plot of the CCC (between the iterative 3D and the object) against the cycles of the iteration. All 3Ds were low-pass filtered to 8 Å. Bars: 20 nm.

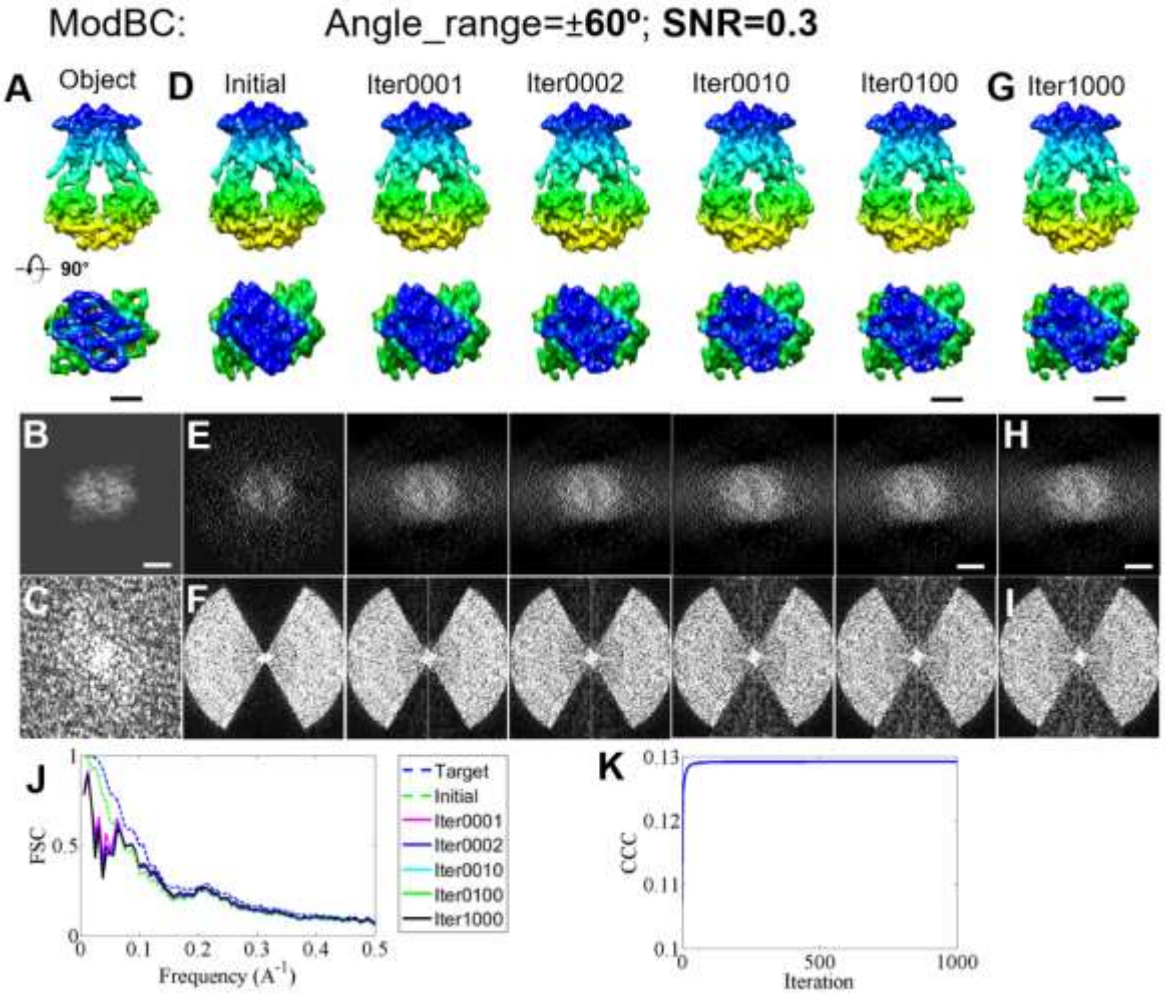

Supplementary Fig. 25 | The missing-wedge correction on a simulated 3D map of $ModB_2C_2$ reconstructed from the noisy ±60° tilt series (SNR=0.3) (A) A 3D object shown from two perpendicular views. The object was a generated from the crystal structure of $ModB_2C_2$, and the idea 3D was constructed from the tilt series of the noisy 2D projections (SNR=0.3) of the object from tilt angles in a range of ±90° in steps of 1.5°, while the initial 3D was reconstructed from angles of ±60°. (**B**) The projection of the object on the X-Z plane, and (**C**) the corresponding Fourier transform. (**D**) The initial 3D and iterative 3D maps, shown from perpendicular views. (**E**) Their corresponding projections on the X-Z plane and the (**F**) Fourier transforms. The mask corresponding to ~3 times the molecular weight of $ModB_2C_2$ was generated from the low-passed filtered object (~40 Å). (**G**) The final corrected 3D after 1,000 cycles of iteration (round 1, Rd_1), shown from two perpendicular views, and (**H**) the projection along the X-Z plane and the corresponding (**I**) Fourier transform. (**J**) FSC curves of the iterative 3Ds against the object. The blue dashed

line is the ideal FSC curve, calculated between the ideal 3D and the object. The dashed green line is the initial FSC, calculated between the initial 3D and the object. The rest solid lines are the iterative FSC calculated between the object and 3Ds after 1 (in purple), 2 (in blue), 10 (in cyan), 100 (in green), and 1,000 (in black) cycles of iteration. (**K**) The plot of the CCC (between the iterative 3D and the object) against the cycles of the iteration. All 3Ds were low-pass filtered to 8 Å. Bars: 20 nm.

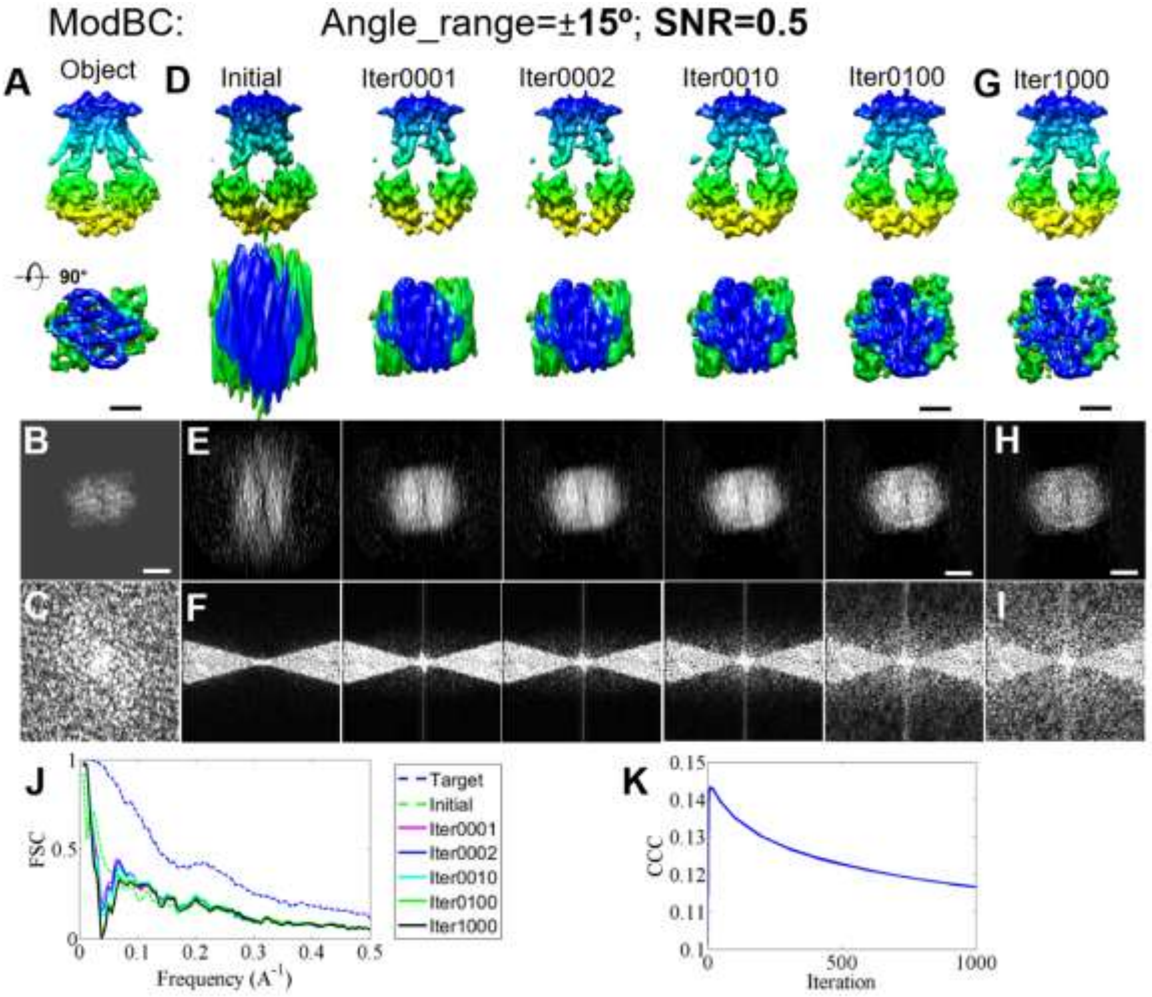

Supplementary Fig. 26 | The missing-wedge correction on a simulated 3D map of $ModB_2C_2$ reconstructed from the noisy ±15° tilt series (SNR=0.5) (A) A 3D object shown from two perpendicular views. The object was a generated from the crystal structure of $ModB_2C_2$, and the idea 3D was constructed from the tilt series of the noisy 2D projections (SNR=0.5) of the object from tilt angles in a range of ±90° in steps of 1.5°, while the initial 3D was reconstructed from that within the angle range of ±15°. (**B**) The projection of the object on the X-Z plane, and (**C**) the corresponding Fourier transform. (**D**) The initial 3D and iterative 3D maps, shown from perpendicular views. (**E**) Their corresponding projections on the X-Z plane and the (**F**) Fourier transforms. The mask corresponding to ~3 times the molecular weight of $ModB_2C_2$ was generated from the low-passed filtered object (~40 Å). (**G**) The final corrected 3D after 1,000 cycles of iteration (round 1, Rd_1), shown from two perpendicular views, and (**H**) the projection along the X-Z plane and the corresponding (**I**) Fourier transform. (**J**) FSC curves of the iterative 3Ds against the object. The blue dashed line is the ideal FSC curve, calculated between the ideal 3D and the object. The

dashed green line is the initial FSC, calculated between the initial 3D and the object. The rest solid lines are the iterative FSC calculated between the object and 3Ds after 1 (in purple), 2 (in blue), 10 (in cyan), 100 (in green), and 1,000 (in black) cycles of iteration. (**K**) The plot of the CCC (between the iterative 3D and the object) against the cycles of the iteration. All 3Ds were low-pass filtered to 8 Å. Bars: 20 nm.

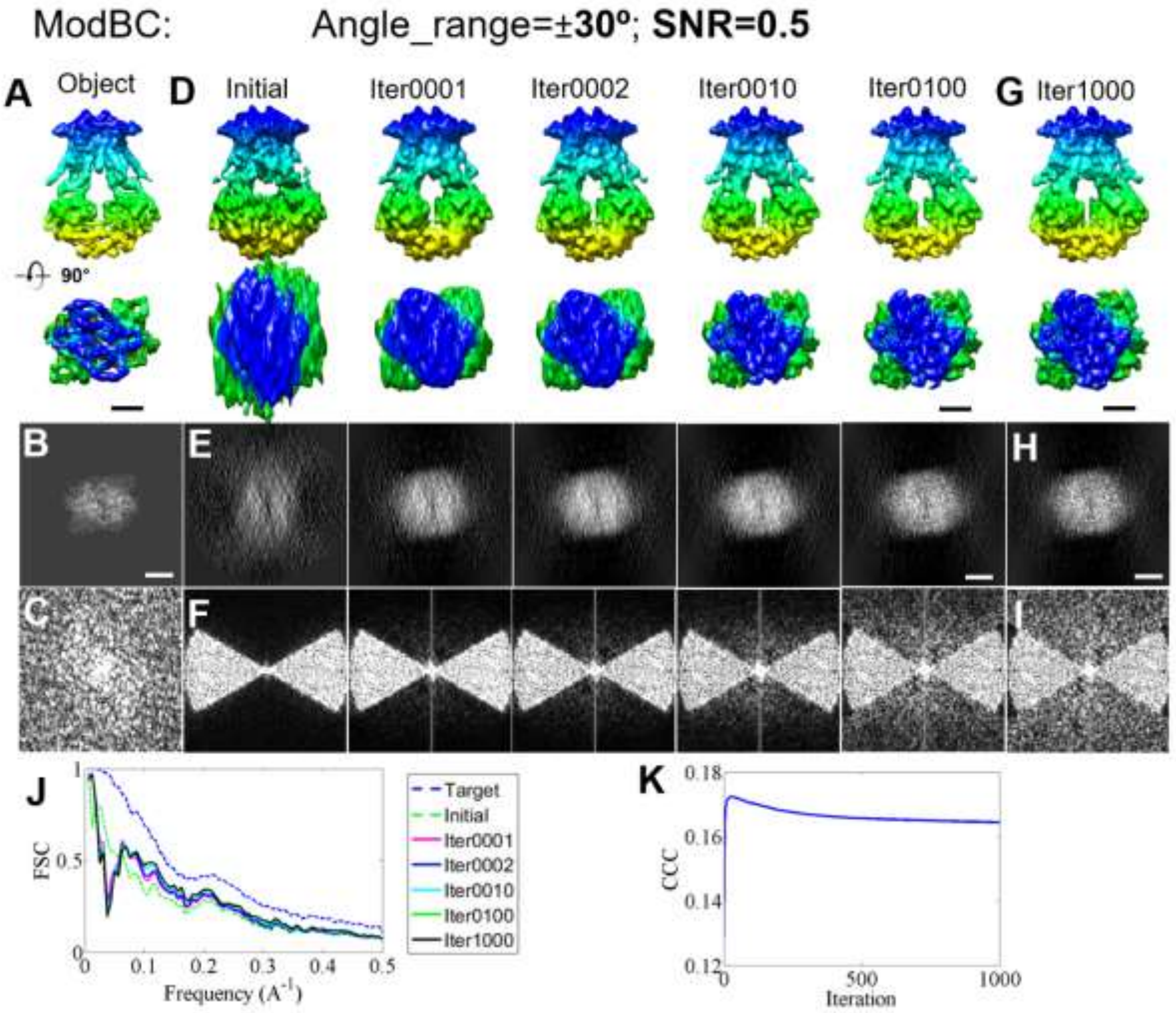

Supplementary Fig. 27 | The missing-wedge correction on a simulated 3D map of $ModB_2C_2$ reconstructed from the noisy ±30° tilt series (SNR=0.5) (A) A 3D object shown from two perpendicular views. The object was a generated from the crystal structure of $ModB_2C_2$, and the idea 3D was constructed from the tilt series of the noisy 2D projections (SNR=0.5) of the object from tilt angles in a range of ±90° in steps of 1.5°, while the initial 3D was reconstructed from angles of ±30°. (**B**) The projection of the object on the X-Z plane, and (**C**) the corresponding Fourier transform. (**D**) The initial 3D and iterative 3D maps, shown from perpendicular views. (**E**) Their corresponding projections on the X-Z plane and the (**F**) Fourier transforms. The mask corresponding to ~3 times the molecular weight of $ModB_2C_2$ was generated from the low-passed filtered object (~40 Å). (**G**) The final corrected 3D after 1,000 cycles of iteration (round 1, Rd_1), shown from two perpendicular views, and (**H**) the projection along the X-Z plane and the corresponding (**I**) Fourier transform. (**J**) FSC curves of the iterative 3Ds against the object. The blue dashed

line is the ideal FSC curve, calculated between the ideal 3D and the object. The dashed green line is the initial FSC, calculated between the initial 3D and the object. The rest solid lines are the iterative FSC calculated between the object and 3Ds after 1 (in purple), 2 (in blue), 10 (in cyan), 100 (in green), and 1,000 (in black) cycles of iteration. (**K**) The plot of the CCC (between the iterative 3D and the object) against the cycles of the iteration. All 3Ds were low-pass filtered to 8 Å. Bars: 20 nm.

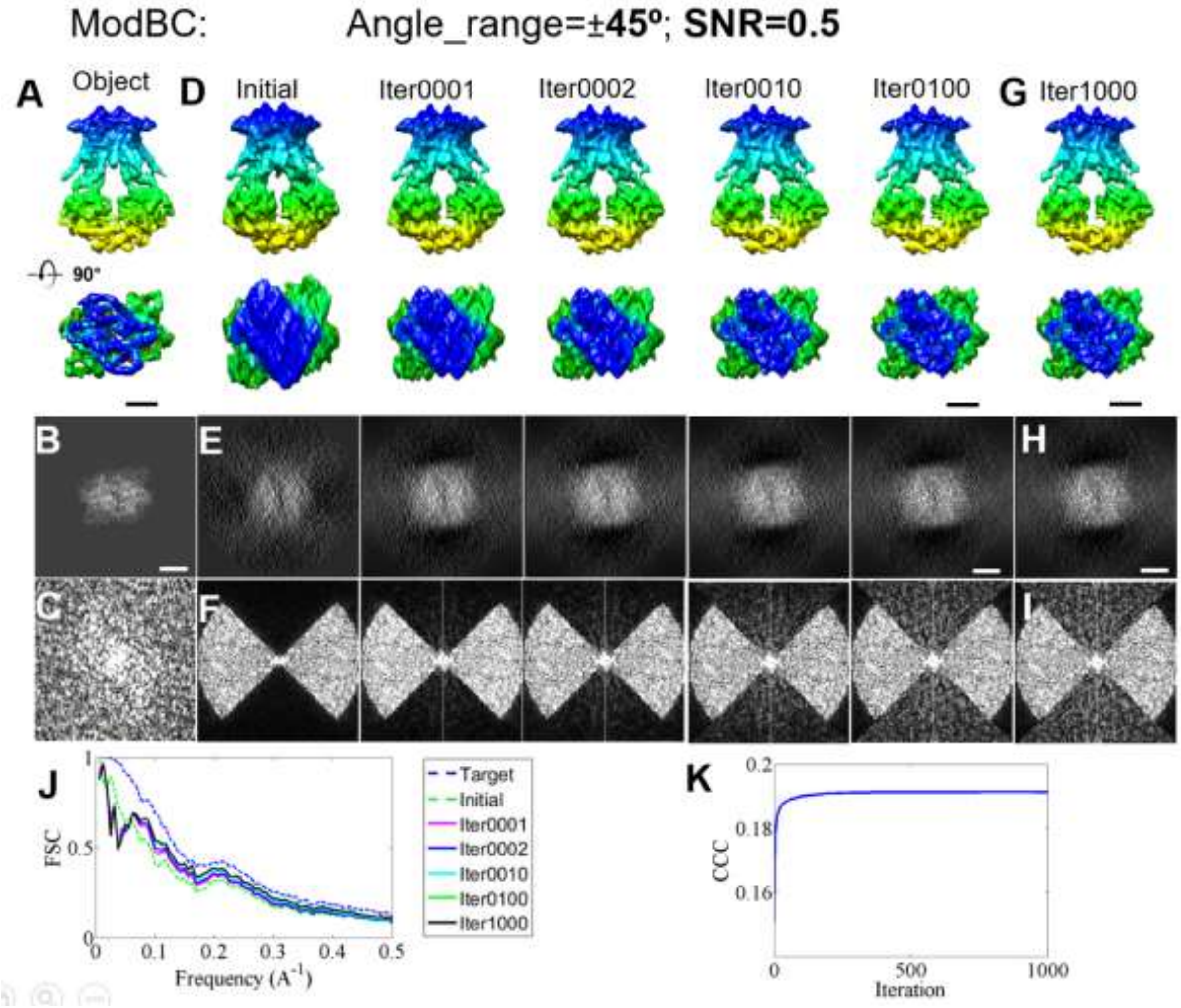

Supplementary Fig. 28 | The missing-wedge correction on a simulated 3D map of $ModB_2C_2$ reconstructed from the noisy ±45° tilt series (SNR=0.5) (A) A 3D object shown from two perpendicular views. The object was a generated from the crystal structure of $ModB_2C_2$, and the idea 3D was constructed from the tilt series of the noisy 2D projections (SNR=0.5) of the object from tilt angles in a range of ±90° in steps of 1.5°, while the initial 3D was reconstructed from angles of ±45°. (**B**) The projection of the object on the X-Z plane, and (**C**) the corresponding Fourier transform. (**D**) The initial 3D and iterative 3D maps, shown from perpendicular views. (**E**) Their corresponding projections on the X-Z plane and the (**F**) Fourier transforms. The mask corresponding to ~3 times the molecular weight of $ModB_2C_2$ was generated from the low-passed filtered object (~40 Å). (**G**) The final corrected 3D after 1,000 cycles of iteration (round 1, Rd_1), shown from two perpendicular views, and (**H**) the projection along the X-Z plane and the corresponding (**I**) Fourier transform. (**J**) FSC curves of the iterative 3Ds against the object. The blue dashed

line is the ideal FSC curve, calculated between the ideal 3D and the object. The dashed green line is the initial FSC, calculated between the initial 3D and the object. The rest solid lines are the iterative FSC calculated between the object and 3Ds after 1 (in purple), 2 (in blue), 10 (in cyan), 100 (in green), and 1,000 (in black) cycles of iteration. (**K**) The plot of the CCC (between the iterative 3D and the object) against the cycles of the iteration. All 3Ds were low-pass filtered to 8 Å. Bars: 20 nm.

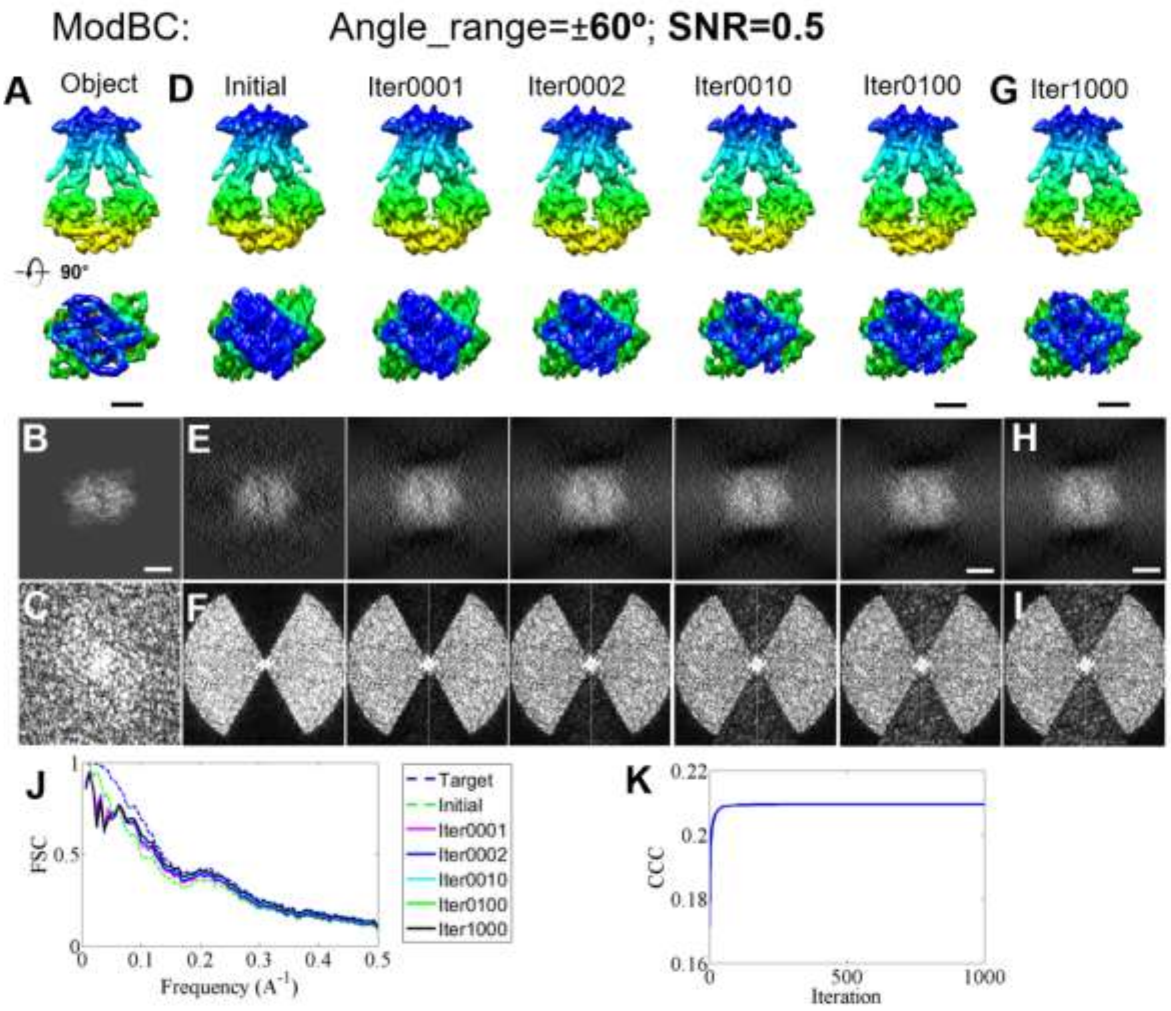

Supplementary Fig. 29 | The missing-wedge correction on a simulated 3D map of $ModB_2C_2$ reconstructed from the noisy ±60° tilt series (SNR=0.5) (A) A 3D object shown from two perpendicular views. The object was a generated from the crystal structure of $ModB_2C_2$, and the idea 3D was constructed from the tilt series of the noisy 2D projections (SNR=0.5) of the object from tilt angles in a range of ±90° in steps of 1.5°, while the initial 3D was reconstructed from angles of ±60°. (**B**) The projection of the object on the X-Z plane, and (**C**) the corresponding Fourier transform. (**D**) The initial 3D and iterative 3D maps, shown from perpendicular views. (**E**) Their corresponding projections on the X-Z plane and the (**F**) Fourier transforms. The mask corresponding to ~3 times the molecular weight of $ModB_2C_2$ was generated from the low-passed filtered object (~40 Å). (**G**) The final corrected 3D after 1,000 cycles of iteration (round 1, Rd_1), shown from two perpendicular views, and (**H**) the projection along the X-Z plane and the corresponding (**I**) Fourier transform. (**J**) FSC curves of the iterative 3Ds against the object. The blue dashed

line is the ideal FSC curve, calculated between the ideal 3D and the object. The dashed green line is the initial FSC, calculated between the initial 3D and the object. The rest solid lines are the iterative FSC calculated between the object and 3Ds after 1 (in purple), 2 (in blue), 10 (in cyan), 100 (in green), and 1,000 (in black) cycles of iteration. (**K**) The plot of the CCC (between the iterative 3D and the object) against the cycles of the iteration. All 3Ds were low-pass filtered to 8 Å. Bars: 20 nm.

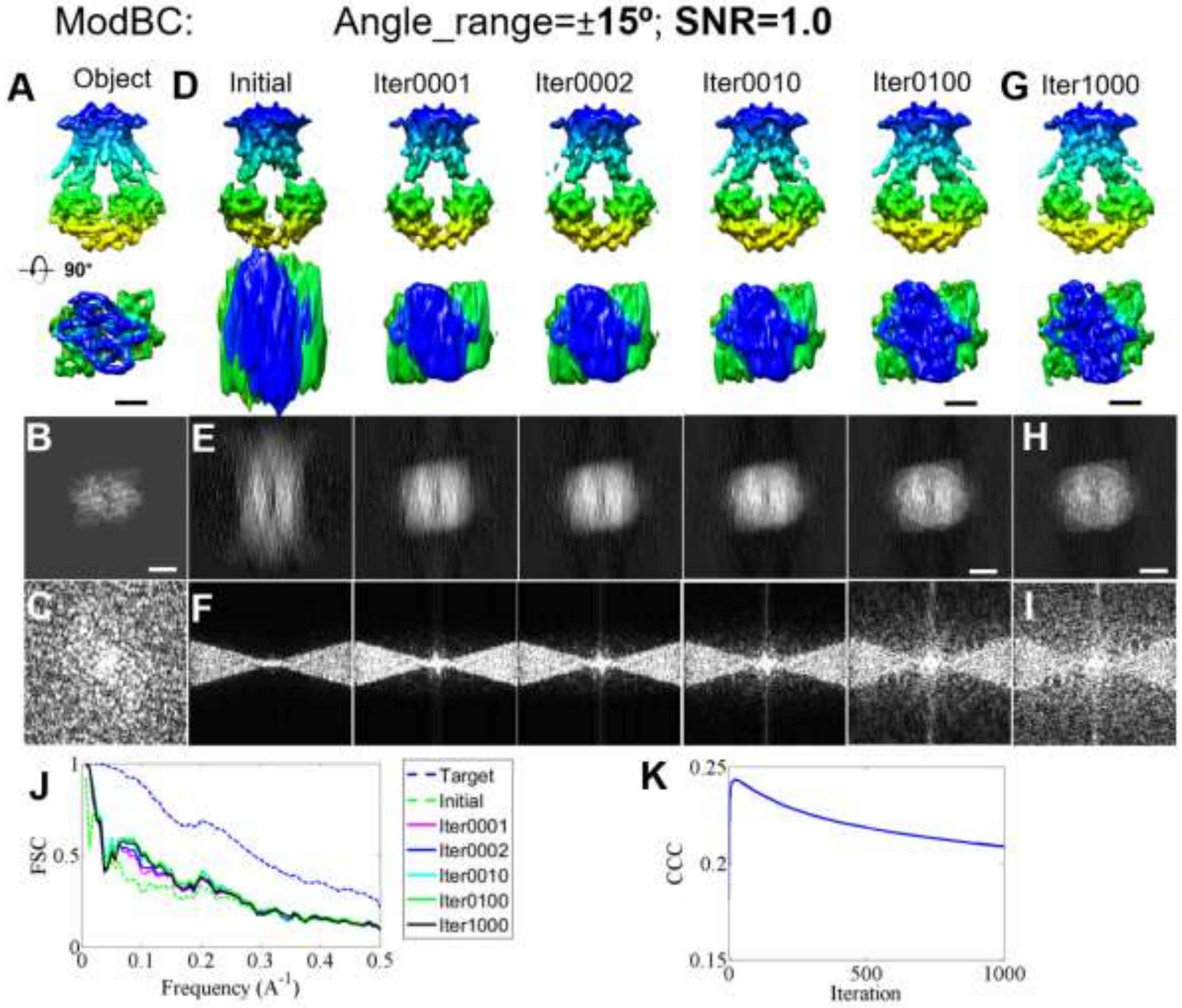

Supplementary Fig. 30 | The missing-wedge correction on a simulated 3D map of ModB$_2$C$_2$ reconstructed from the noisy ±15° tilt series (SNR=1.0) (A) A 3D object shown from two perpendicular views. The object was a generated from the crystal structure of ModB$_2$C$_2$, and the idea 3D was constructed from the tilt series of the noisy 2D projections (SNR=1.0) of the object from tilt angles in a range of ±90° in steps of 1.5°, while the initial 3D was reconstructed from that within the angle range of ±15°. (**B**) The projection of the object on the X-Z plane, and (**C**) the corresponding Fourier transform. (**D**) The initial 3D and iterative 3D maps, shown from perpendicular views. (**E**) Their corresponding projections on the X-Z plane and the (**F**) Fourier transforms. The mask corresponding to ~3 times the molecular weight of ModB$_2$C$_2$ was generated from the low-passed filtered object (~40 Å). (**G**) The final corrected 3D after 1,000 cycles of iteration (round 1, Rd_1), shown from two perpendicular views, and (**H**) the projection along the X-Z plane and the corresponding (**I**) Fourier transform. (**J**) FSC curves of the iterative 3Ds against the

object. The blue dashed line is the ideal FSC curve, calculated between the ideal 3D and the object. The dashed green line is the initial FSC, calculated between the initial 3D and the object. The rest solid lines are the iterative FSC calculated between the object and 3Ds after 1 (in purple), 2 (in blue), 10 (in cyan), 100 (in green), and 1,000 (in black) cycles of iteration. (**K**) The plot of the CCC (between the iterative 3D and the object) against the cycles of the iteration. All 3Ds were low-pass filtered to 8 Å. Bars: 20 nm.

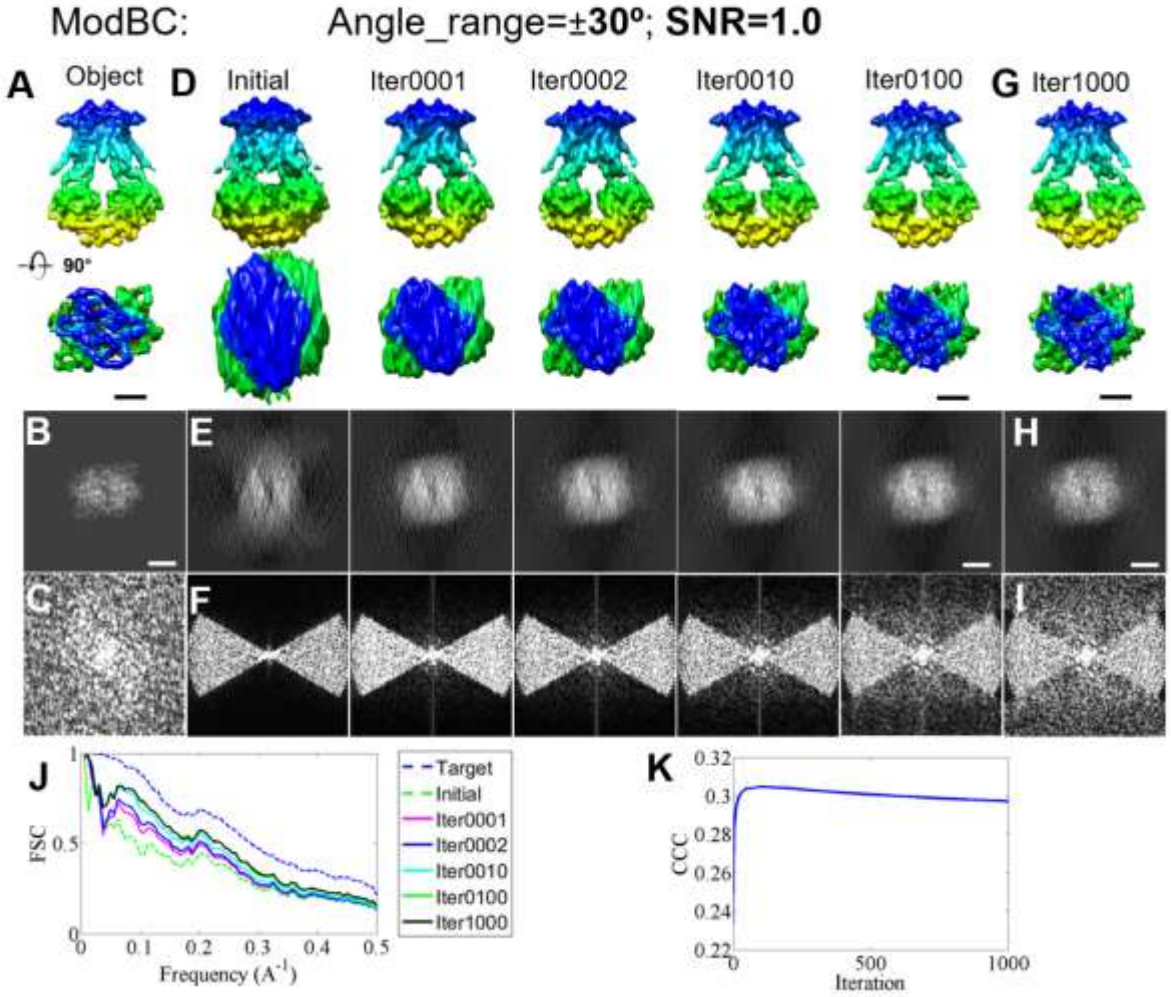

Supplementary Fig. 31 | The missing-wedge correction on a simulated 3D map of ModB$_2$C$_2$ reconstructed from the noisy ±30° tilt series (SNR=1.0) (A) A 3D object shown from two perpendicular views. The object was a generated from the crystal structure of ModB$_2$C$_2$, and the idea 3D was constructed from the tilt series of the noisy 2D projections (SNR=1.0) of the object from tilt angles in a range of ±90° in steps of 1.5°, while the initial 3D was reconstructed from angles of ±30°. (**B**) The projection of the object on the X-Z plane, and (**C**) the corresponding Fourier transform. (**D**) The initial 3D and iterative 3D maps, shown from perpendicular views. (**E**) Their corresponding projections on the X-Z plane and the (**F**) Fourier transforms. The mask corresponding to ~3 times the molecular weight of ModB$_2$C$_2$ was generated from the low-passed filtered object (~40 Å). (**G**) The final corrected 3D after 1,000 cycles of iteration (round 1, Rd_1), shown from two perpendicular views, and (**H**) the projection along the X-Z plane and the corresponding (**I**) Fourier transform. (**J**) FSC curves of the iterative 3Ds against the object. The blue dashed

line is the ideal FSC curve, calculated between the ideal 3D and the object. The dashed green line is the initial FSC, calculated between the initial 3D and the object. The rest solid lines are the iterative FSC calculated between the object and 3Ds after 1 (in purple), 2 (in blue), 10 (in cyan), 100 (in green), and 1,000 (in black) cycles of iteration. (**K**) The plot of the CCC (between the iterative 3D and the object) against the cycles of the iteration. All 3Ds were low-pass filtered to 8 Å. Bars: 20 nm.

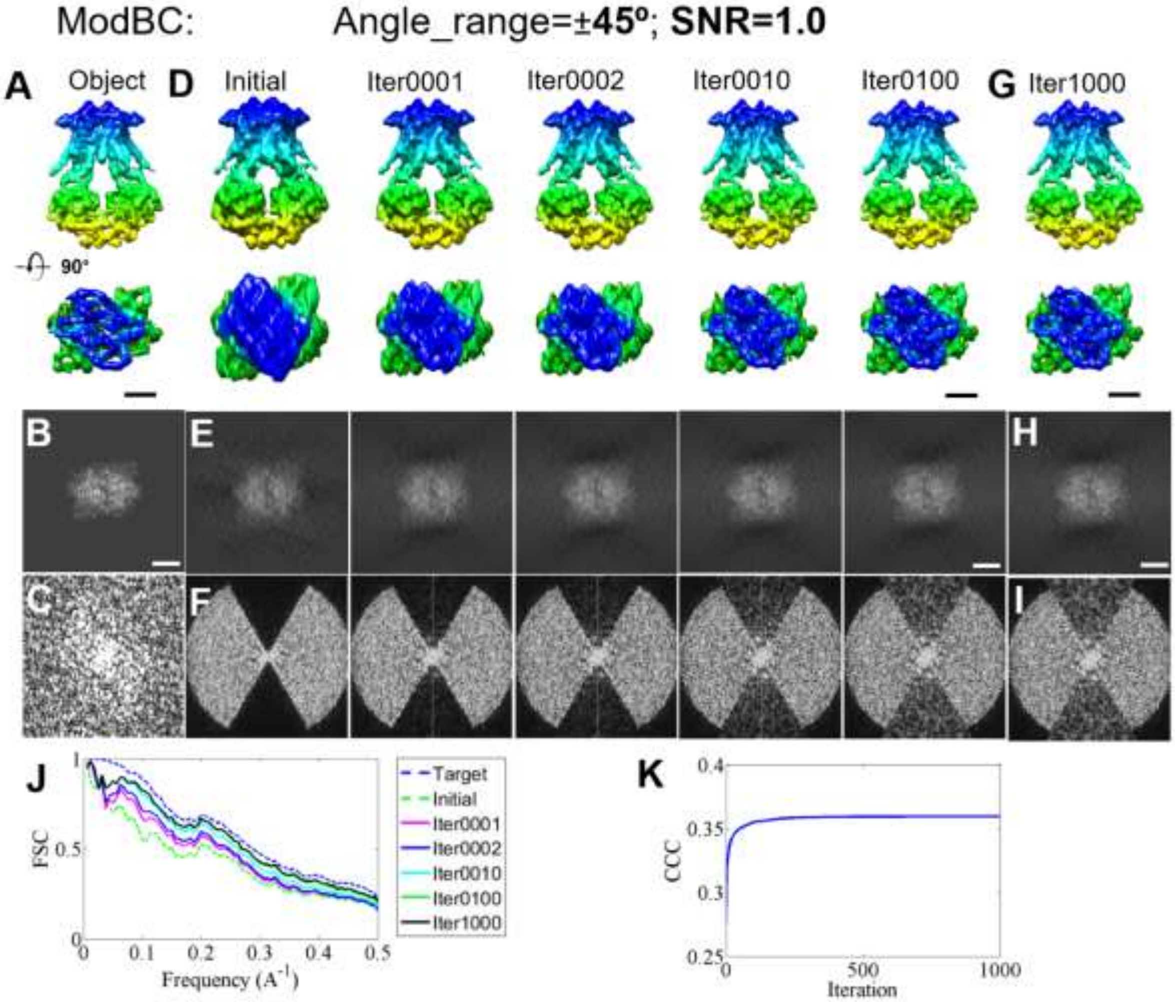

Supplementary Fig. 32 | The missing-wedge correction on a simulated 3D map of $ModB_2C_2$ reconstructed from the noisy ±45° tilt series (SNR=1.0) (A) A 3D object shown from two perpendicular views. The object was a generated from the crystal structure of $ModB_2C_2$, and the idea 3D was constructed from the tilt series of the noisy 2D projections (SNR=1.0) of the object from tilt angles in a range of ±90° in steps of 1.5°, while the initial 3D was reconstructed from angles of ±45°. (**B**) The projection of the object on the X-Z plane, and (**C**) the corresponding Fourier transform. (**D**) The initial 3D and iterative 3D maps, shown from perpendicular views. (**E**) Their corresponding projections on the X-Z plane and the (**F**) Fourier transforms. The mask corresponding to ~3 times the molecular weight of $ModB_2C_2$ was generated from the low-passed filtered object (~40 Å). (**G**) The final corrected 3D after 1,000 cycles of iteration (round 1, Rd_1), shown from two perpendicular views, and (**H**) the projection along the X-Z plane and the corresponding (**I**) Fourier transform. (**J**) FSC curves of the iterative 3Ds against the object. The blue dashed

line is the ideal FSC curve, calculated between the ideal 3D and the object. The dashed green line is the initial FSC, calculated between the initial 3D and the object. The rest solid lines are the iterative FSC calculated between the object and 3Ds after 1 (in purple), 2 (in blue), 10 (in cyan), 100 (in green), and 1,000 (in black) cycles of iteration. (**K**) The plot of the CCC (between the iterative 3D and the object) against the cycles of the iteration. All 3Ds were low-pass filtered to 8 Å. Bars: 20 nm.

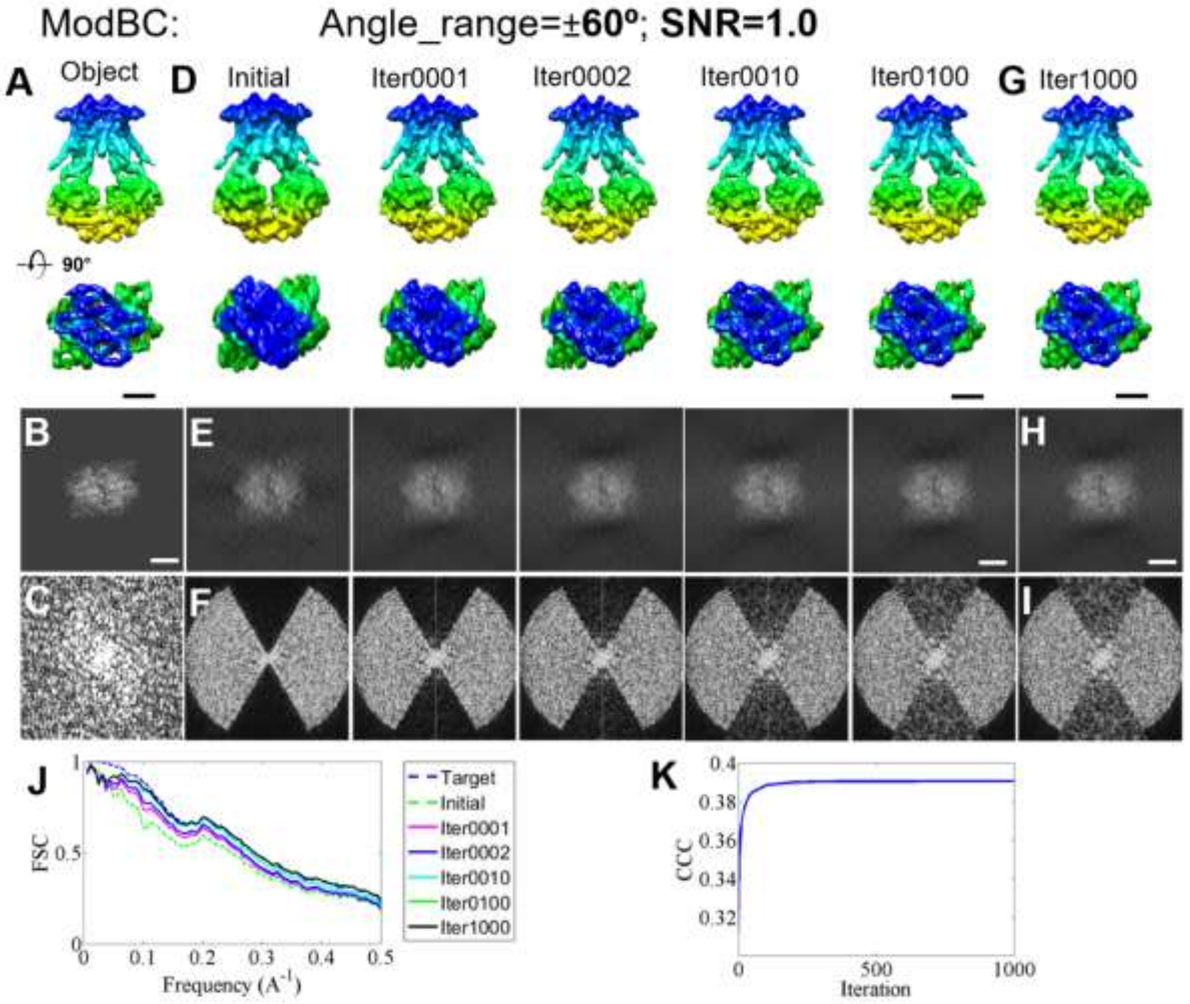

Supplementary Fig. 33 | The missing-wedge correction on a simulated 3D map of $ModB_2C_2$ reconstructed from the noisy ±60° tilt series (SNR=1.0) (A) A 3D object shown from two perpendicular views. The object was a generated from the crystal structure of $ModB_2C_2$, and the idea 3D was constructed from the tilt series of the noisy 2D projections (SNR=1.0) of the object from tilt angles in a range of ±90° in steps of 1.5°, while the initial 3D was reconstructed from angles of ±60°. (**B**) The projection of the object on the X-Z plane, and (**C**) the corresponding Fourier transform. (**D**) The initial 3D and iterative 3D maps, shown from perpendicular views. (**E**) Their corresponding projections on the X-Z plane and the (**F**) Fourier transforms. The mask corresponding to ~3 times the molecular weight of $ModB_2C_2$ was generated from the low-passed filtered object (~40 Å). (**G**) The final corrected 3D after 1,000 cycles of iteration (round 1, Rd_1), shown from two perpendicular views, and (**H**) the projection along the X-Z plane and the corresponding (**I**) Fourier transform. (**J**) FSC curves of the iterative 3Ds against the object. The blue dashed

line is the ideal FSC curve, calculated between the ideal 3D and the object. The dashed green line is the initial FSC, calculated between the initial 3D and the object. The rest solid lines are the iterative FSC calculated between the object and 3Ds after 1 (in purple), 2 (in blue), 10 (in cyan), 100 (in green), and 1,000 (in black) cycles of iteration. (**K**) The plot of the CCC (between the iterative 3D and the object) against the cycles of the iteration. All 3Ds were low-pass filtered to 8 Å. Bars: 20 nm.

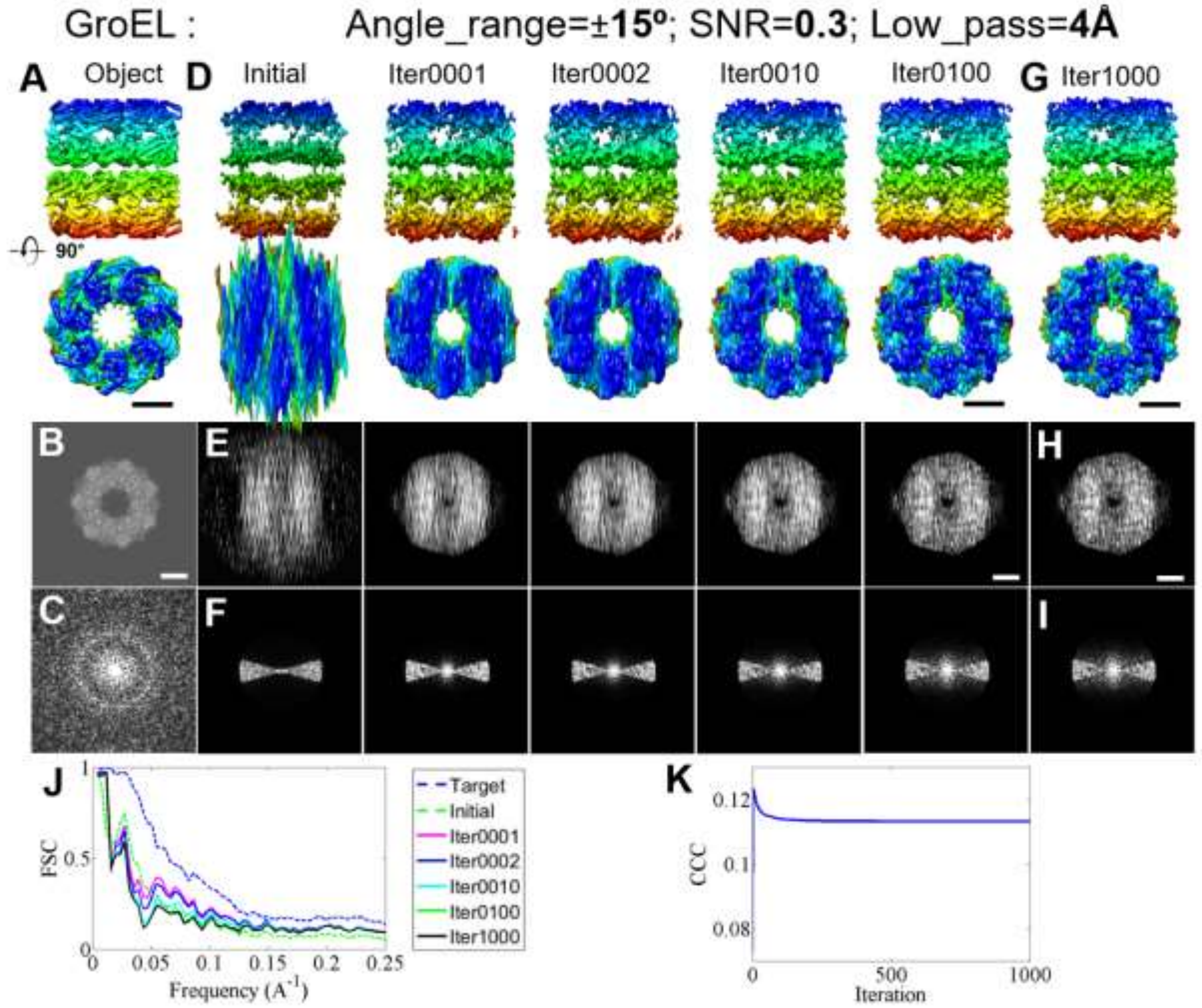

Supplementary Fig. 34 | The missing-wedge correction on a simulated 3D map of GroEL reconstructed from the low-pass filtered ±15° tilt series that with SNR=0.3 (A) A 3D object shown from two perpendicular views. The object was a generated from the crystal structure of GroEL, and the idea 3D was constructed from the tilt series of the noisy 2D projections (SNR=0.3) of the object from tilt angles in a range of ±90° in steps of 1.5°, while the initial 3D was reconstructed from that within the angle range of ±15° tilt series after low-pass filtered to 4 Å. (**B**) The projection of the object on the X-Z plane, and (**C**) the corresponding Fourier transform. (**D**) The initial 3D and iterative 3D maps, shown from perpendicular views. (**E**) Their corresponding projections on the X-Z plane and the (**F**) Fourier transforms. The mask corresponding to ~3 times the molecular weight of GroEL was generated from the low-passed filtered object (~40 Å). (**G**) The final corrected 3D after 1,000 cycles of iteration (round 1, Rd_1), shown from two perpendicular views, and (**H**) the projection along the X-Z plane and the corresponding (**I**) Fourier

transform. (**J**) FSC curves of the iterative 3Ds against the object. The blue dashed line is the ideal FSC curve, calculated between the ideal 3D and the object. The dashed green line is the initial FSC, calculated between the initial 3D and the object. The rest solid lines are the iterative FSC calculated between the object and 3Ds after 1 (in purple), 2 (in blue), 10 (in cyan), 100 (in green), and 1,000 (in black) cycles of iteration. (**K**) The plot of the CCC (between the iterative 3D and the object) against the cycles of the iteration. All 3Ds were low-pass filtered to 8 Å. Bars: 50 nm.

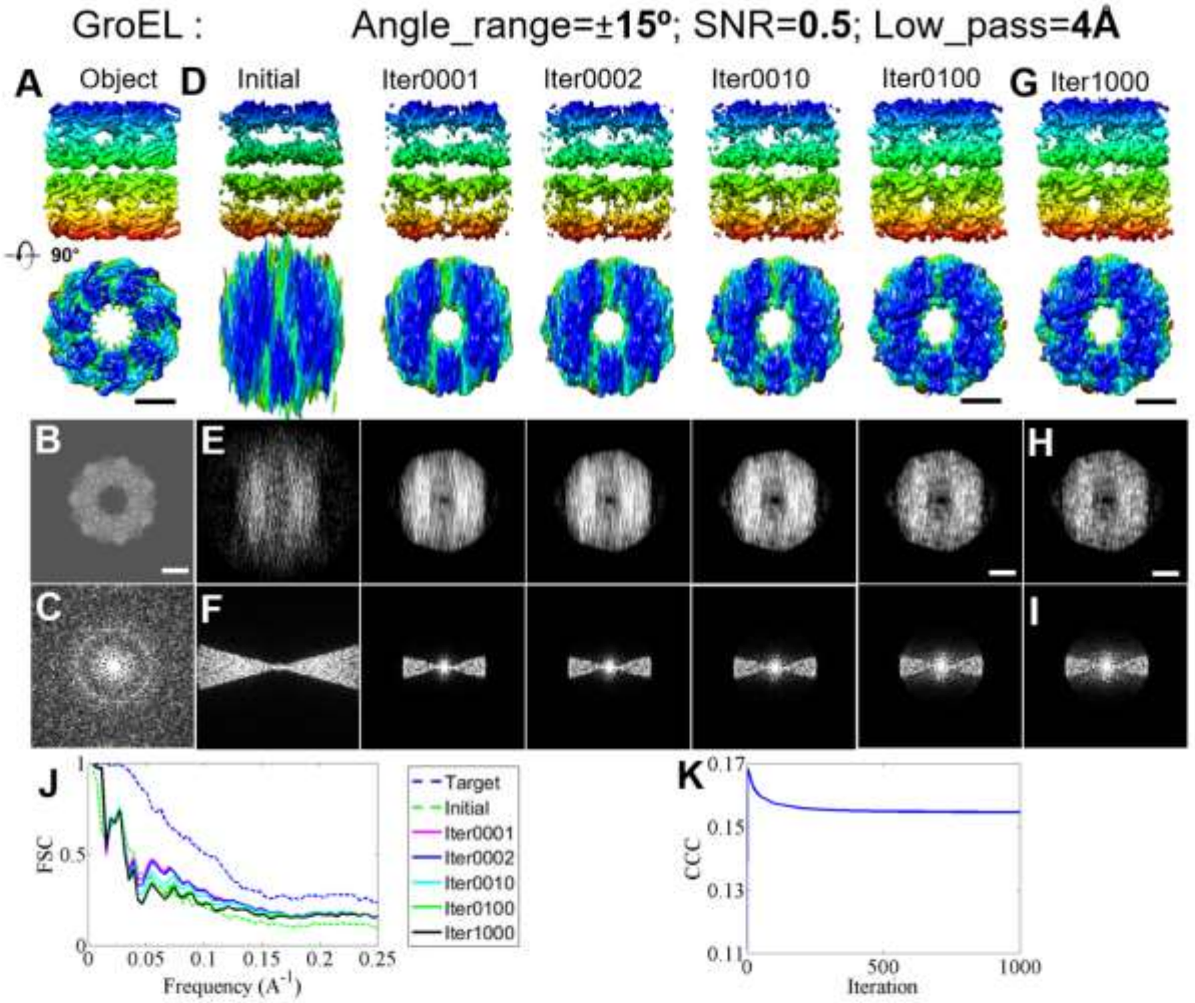

Supplementary Fig. 35 | The missing-wedge correction on a simulated 3D map of GroEL reconstructed from the low-pass filtered ±15° tilt series that with SNR=0.5 (A) A 3D object shown from two perpendicular views. The object was a generated from the crystal structure of GroEL, and the idea 3D was constructed from the tilt series of the noisy 2D projections (SNR=0.5) of the object from tilt angles in a range of ±90° in steps of 1.5°, while the initial 3D was reconstructed from that within the angle range of ±15° tilt series after low-pass filtered to 4 Å. (**B**) The projection of the object on the X-Z plane, and (**C**) the corresponding Fourier transform. (**D**) The initial 3D and iterative 3D maps, shown from perpendicular views. (**E**) Their corresponding projections on the X-Z plane and the (**F**) Fourier transforms. The mask corresponding to ~3 times the molecular weight of GroEL was generated from the low-passed filtered object (~40 Å). (**G**) The final corrected 3D after 1,000 cycles of iteration (round 1, Rd_1), shown from two

perpendicular views, and (**H**) the projection along the X-Z plane and the corresponding (**I**) Fourier transform. (**J**) FSC curves of the iterative 3Ds against the object. The blue dashed line is the ideal FSC curve, calculated between the ideal 3D and the object. The dashed green line is the initial FSC, calculated between the initial 3D and the object. The rest solid lines are the iterative FSC calculated between the object and 3Ds after 1 (in purple), 2 (in blue), 10 (in cyan), 100 (in green), and 1,000 (in black) cycles of iteration. (**K**) The plot of the CCC (between the iterative 3D and the object) against the cycles of the iteration. All 3Ds were low-pass filtered to 8 Å. Bars: 50 nm.

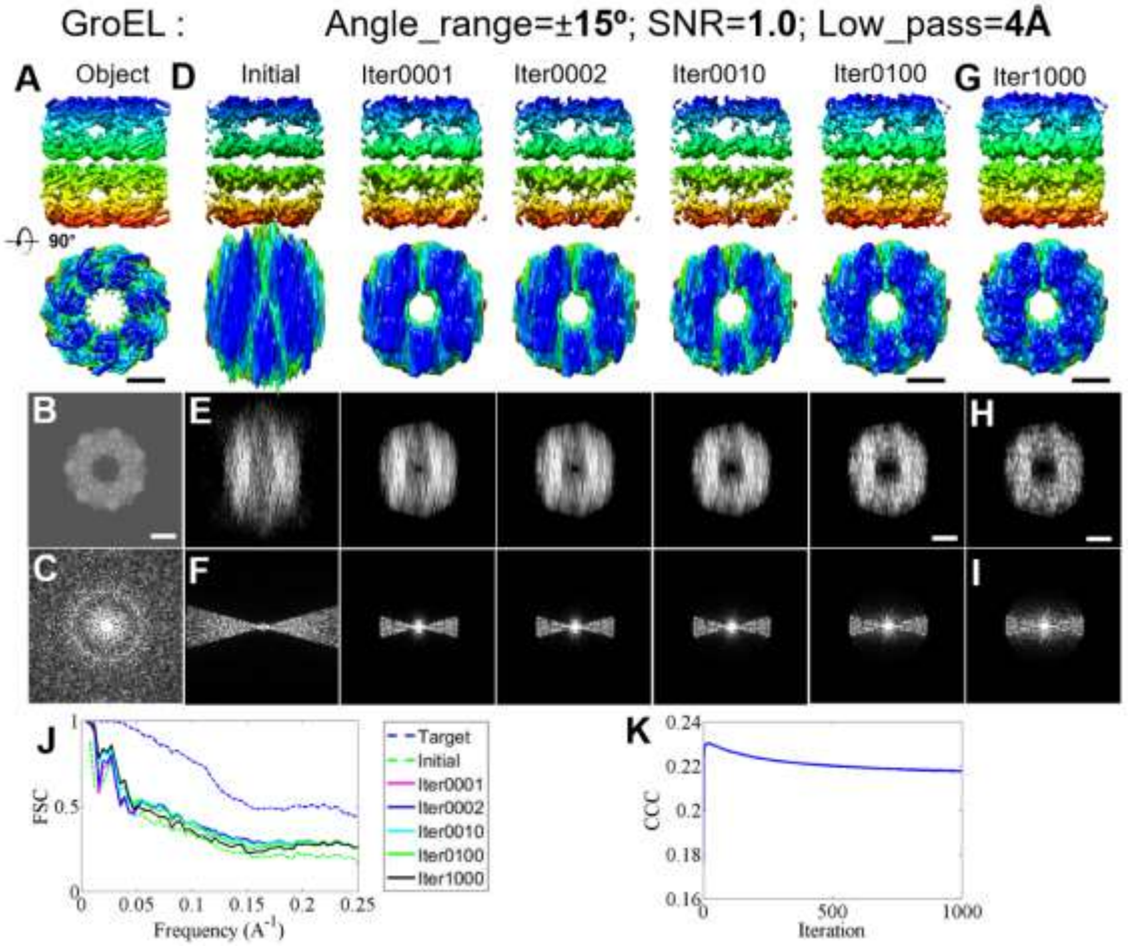

Supplementary Fig. 36 | The missing-wedge correction on a simulated 3D map of GroEL reconstructed from the low-pass filtered ±15° tilt series that with SNR=1.0 (A) A 3D object shown from two perpendicular views. The object was a generated from the crystal structure of GroEL, and the idea 3D was constructed from the tilt series of the noisy 2D projections (SNR=1.0) of the object from tilt angles in a range of ±90° in steps of 1.5°, while the initial 3D was reconstructed from that within the angle range of ±15° tilt series after low-pass filtered to 4 Å. (**B**) The projection of the object on the X-Z plane, and (**C**) the corresponding Fourier transform. (**D**) The initial 3D and iterative 3D maps, shown from perpendicular views. (**E**) Their corresponding projections on the X-Z plane and the (**F**) Fourier transforms. The mask corresponding to ~3 times the molecular weight of GroEL was generated from the low-passed filtered object (~40 Å). (**G**) The final corrected 3D after 1,000 cycles of iteration (round 1, Rd_1), shown from two perpendicular views, and (**H**) the projection along the X-Z plane and the corresponding (**I**) Fourier

transform. (**J**) FSC curves of the iterative 3Ds against the object. The blue dashed line is the ideal FSC curve, calculated between the ideal 3D and the object. The dashed green line is the initial FSC, calculated between the initial 3D and the object. The rest solid lines are the iterative FSC calculated between the object and 3Ds after 1 (in purple), 2 (in blue), 10 (in cyan), 100 (in green), and 1,000 (in black) cycles of iteration. (**K**) The plot of the CCC (between the iterative 3D and the object) against the cycles of the iteration. All 3Ds were low-pass filtered to 8 Å. Bars: 50 nm.

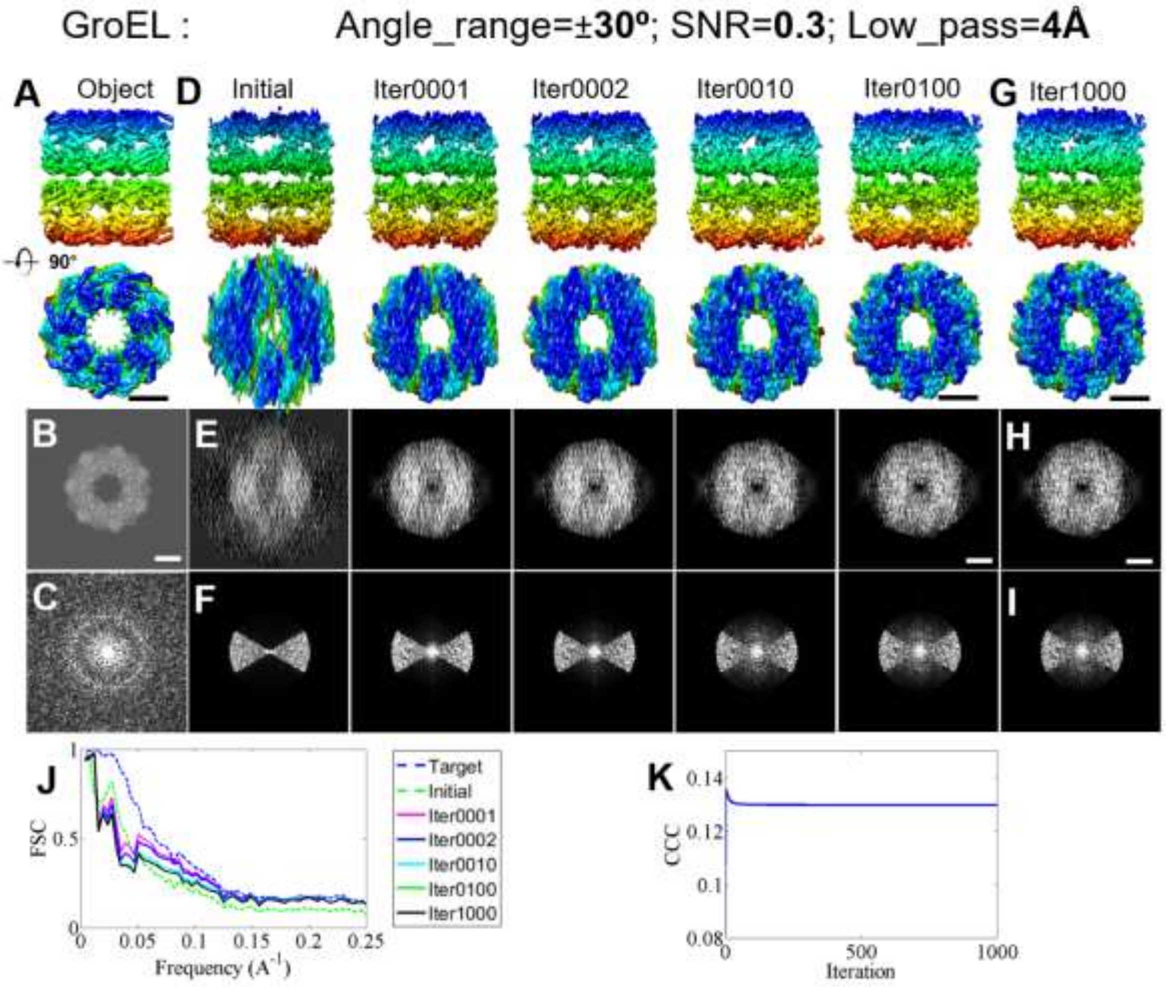

Supplementary Fig. 37 | The missing-wedge correction on a simulated 3D map of GroEL reconstructed from the low-pass filtered ±30° tilt series that with SNR=0.3 (A) A 3D object shown from two perpendicular views. The object was a generated from the crystal structure of GroEL, and the idea 3D was constructed from the tilt series of the noisy 2D projections (SNR=0.3) of the object from tilt angles in a range of ±90° in steps of 1.5°, while the initial 3D was reconstructed from angles of ±30° tilt series after low-pass filtered to 4 Å. (**B**) The projection of the object on the X-Z plane, and (**C**) the corresponding Fourier transform. (**D**) The initial 3D and iterative 3D maps, shown from perpendicular views. (**E**) Their corresponding projections on the X-Z plane and the (**F**) Fourier transforms. The mask corresponding to ~3 times the molecular weight of GroEL was generated from the low-passed filtered object (~40 Å). (**G**) The final corrected 3D after 1,000 cycles of iteration (round 1, Rd_1), shown from two perpendicular views,

and (**H**) the projection along the X-Z plane and the corresponding (**I**) Fourier transform. (**J**) FSC curves of the iterative 3Ds against the object. The blue dashed line is the ideal FSC curve, calculated between the ideal 3D and the object. The dashed green line is the initial FSC, calculated between the initial 3D and the object. The rest solid lines are the iterative FSC calculated between the object and 3Ds after 1 (in purple), 2 (in blue), 10 (in cyan), 100 (in green), and 1,000 (in black) cycles of iteration. (**K**) The plot of the CCC (between the iterative 3D and the object) against the cycles of the iteration. All 3Ds were low-pass filtered to 8 Å. Bars: 50 nm.

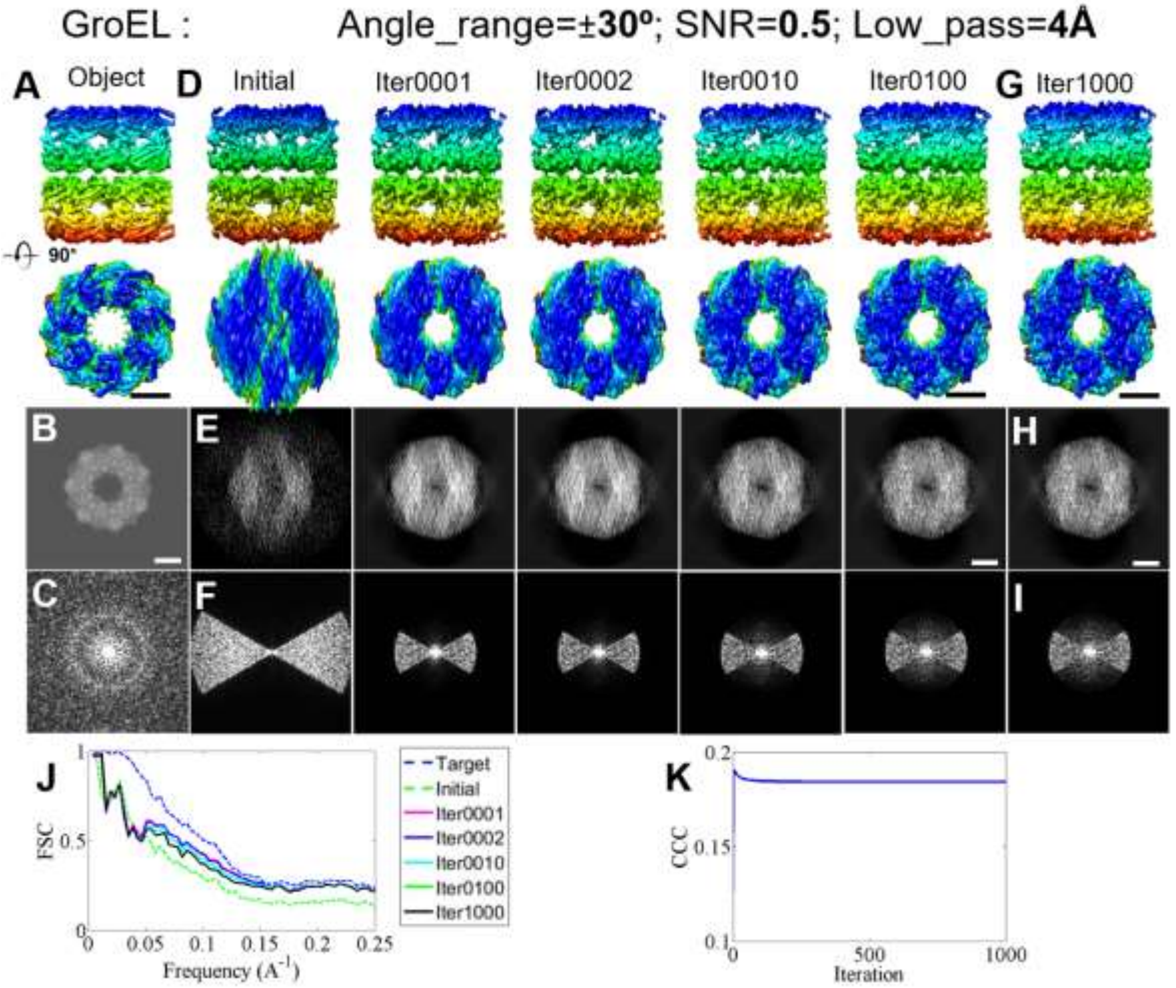

Supplementary Fig. 38 | The missing-wedge correction on a simulated 3D map of GroEL reconstructed from the low-pass filtered ±30° tilt series that with SNR=0.5 (A) A 3D object shown from two perpendicular views. The object was a generated from the crystal structure of GroEL, and the idea 3D was constructed from the tilt series of the noisy 2D projections (SNR=0.5) of the object from tilt angles in a range of ±90° in steps of 1.5°, while the initial 3D was reconstructed from angles of ±30° tilt series after low-pass filtered to 4 Å. (**B**) The projection of the object on the X-Z plane, and (**C**) the corresponding Fourier transform. (**D**) The initial 3D and iterative 3D maps, shown from perpendicular views. (**E**) Their corresponding projections on the X-Z plane and the (**F**) Fourier transforms. The mask corresponding to ~3

times the molecular weight of GroEL was generated from the low-passed filtered object (~40 Å). (**G**) The final corrected 3D after 1,000 cycles of iteration (round 1, Rd_1), shown from two perpendicular views, and (**H**) the projection along the X-Z plane and the corresponding (**I**) Fourier transform. (**J**) FSC curves of the iterative 3Ds against the object. The blue dashed line is the ideal FSC curve, calculated between the ideal 3D and the object. The dashed green line is the initial FSC, calculated between the initial 3D and the object. The rest solid lines are the iterative FSC calculated between the object and 3Ds after 1 (in purple), 2 (in blue), 10 (in cyan), 100 (in green), and 1,000 (in black) cycles of iteration. (**K**) The plot of the CCC (between the iterative 3D and the object) against the cycles of the iteration. All 3Ds were low-pass filtered to 8 Å. Bars: 50 nm.

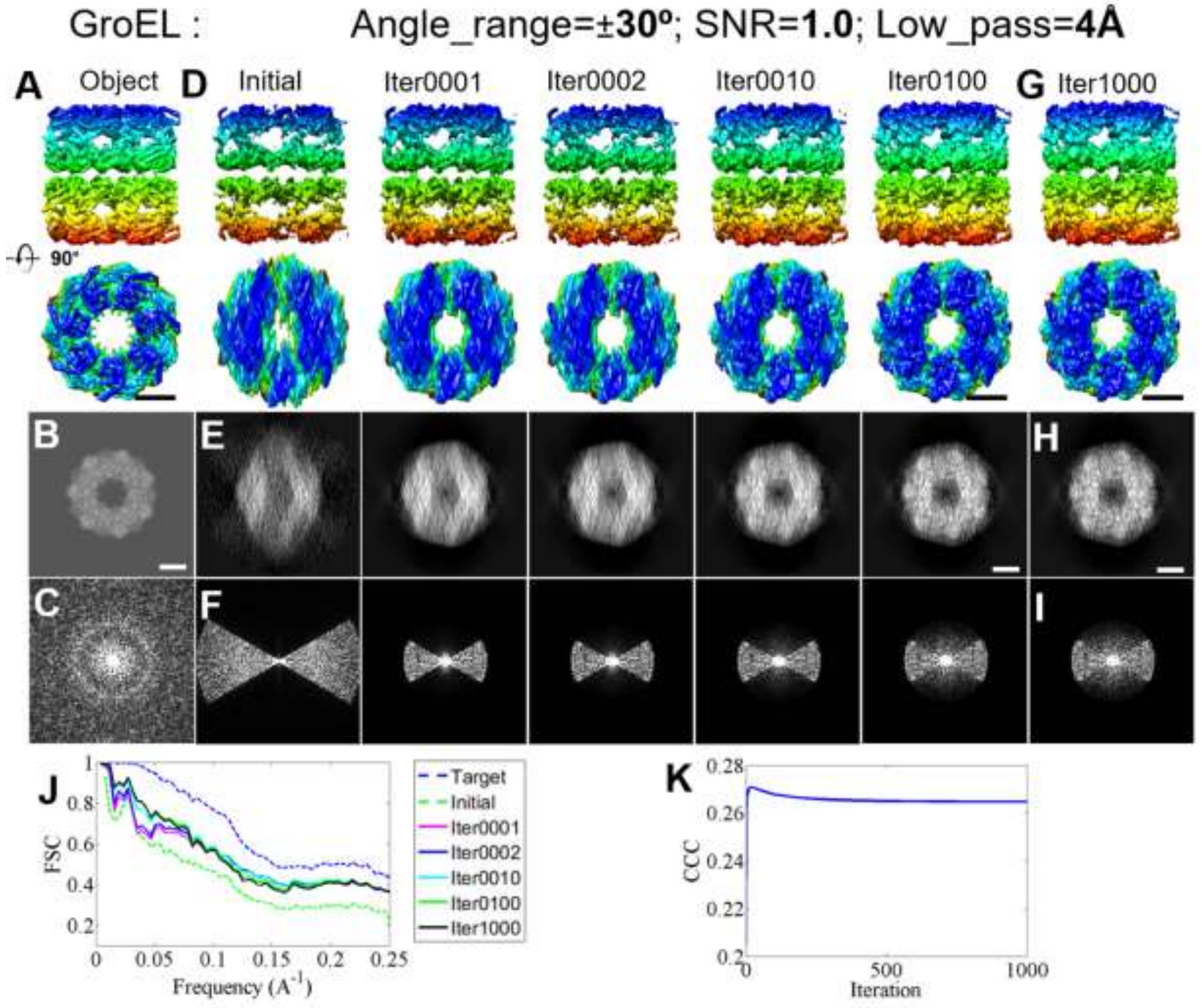

Supplementary Fig. 39 | The missing-wedge correction on a simulated 3D map of GroEL reconstructed from the low-pass filtered ±30° tilt series that with SNR=1.0 (A) A 3D object shown from two perpendicular views. The object was a generated from the crystal structure of GroEL, and the idea 3D was constructed from the tilt series of the noisy 2D projections (SNR=1.0) of the object from tilt angles in a range of ±90° in steps of 1.5°, while the initial 3D was reconstructed from angles of ±30° tilt series after low-pass filtered to 4 Å. (**B**) The projection of the object on the X-Z plane, and (**C**) the corresponding Fourier transform. (**D**) The initial 3D and iterative 3D maps, shown from perpendicular views. (**E**) Their

corresponding projections on the X-Z plane and the (**F**) Fourier transforms. The mask corresponding to ~3 times the molecular weight of GroEL was generated from the low-passed filtered object (~40 Å). (**G**) The final corrected 3D after 1,000 cycles of iteration (round 1, Rd_1), shown from two perpendicular views, and (**H**) the projection along the X-Z plane and the corresponding (**I**) Fourier transform. (**J**) FSC curves of the iterative 3Ds against the object. The blue dashed line is the ideal FSC curve, calculated between the ideal 3D and the object. The dashed green line is the initial FSC, calculated between the initial 3D and the object. The rest solid lines are the iterative FSC calculated between the object and 3Ds after 1 (in purple), 2 (in blue), 10 (in cyan), 100 (in green), and 1,000 (in black) cycles of iteration. (**K**) The plot of the CCC (between the iterative 3D and the object) against the cycles of the iteration. All 3Ds were low-pass filtered to 8 Å. Bars: 50 nm.

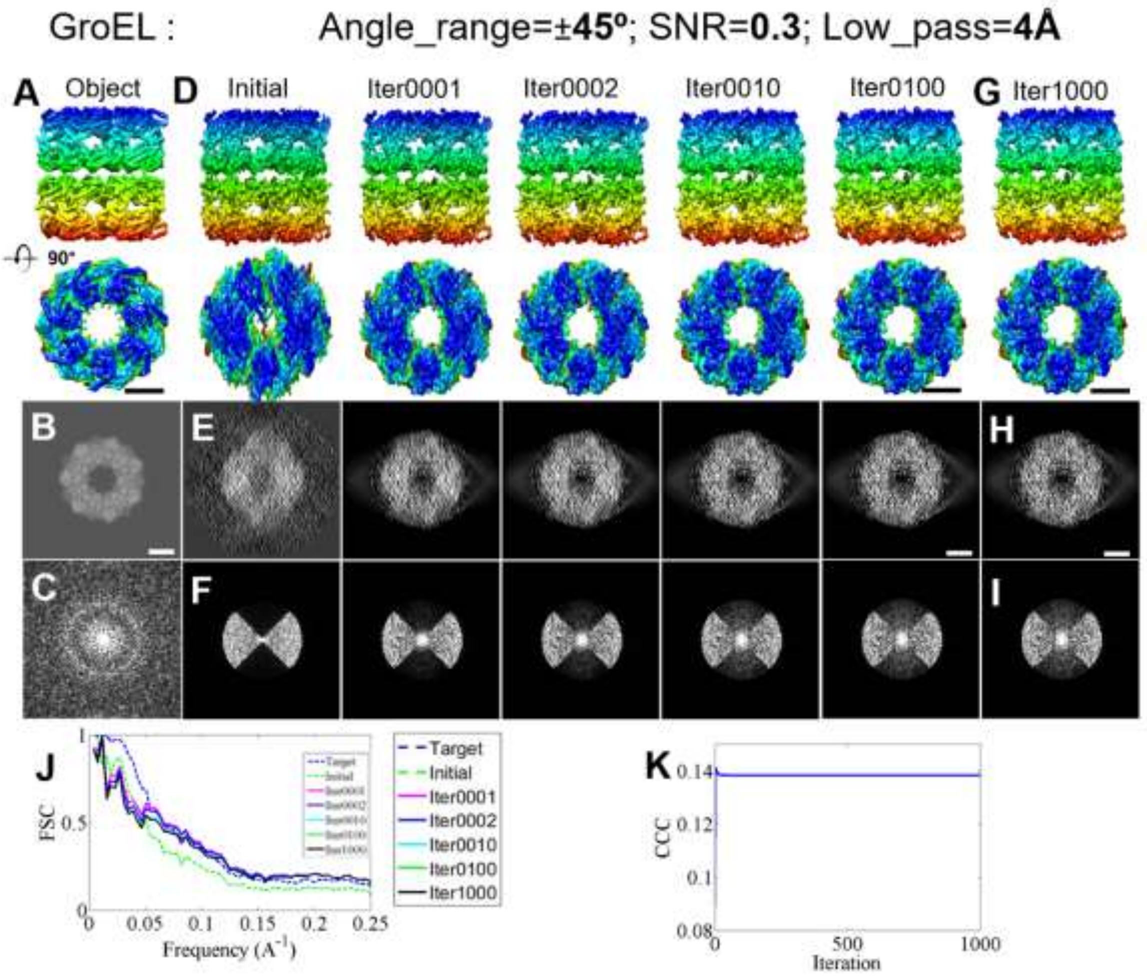

Supplementary Fig. 40 | The missing-wedge correction on a simulated 3D map of GroEL reconstructed from the low-pass filtered ±45° tilt series that with SNR=0.3 (A) A 3D object shown from two perpendicular views. The object was a generated from the crystal structure of GroEL, and the idea 3D was constructed from the tilt series of the noisy 2D projections (SNR=0.3) of the object from tilt angles in a range of ±90° in steps of 1.5°, while the initial 3D was reconstructed from angles of ±45° tilt series after low-pass filtered to 4 Å. (**B**) The projection of the object on the X-Z plane, and (**C**) the corresponding

Fourier transform. (**D**) The initial 3D and iterative 3D maps, shown from perpendicular views. (**E**) Their corresponding projections on the X-Z plane and the (**F**) Fourier transforms. The mask corresponding to ~3 times the molecular weight of GroEL was generated from the low-passed filtered object (~40 Å). (**G**) The final corrected 3D after 1,000 cycles of iteration (round 1, Rd_1), shown from two perpendicular views, and (**H**) the projection along the X-Z plane and the corresponding (**I**) Fourier transform. (**J**) FSC curves of the iterative 3Ds against the object. The blue dashed line is the ideal FSC curve, calculated between the ideal 3D and the object. The dashed green line is the initial FSC, calculated between the initial 3D and the object. The rest solid lines are the iterative FSC calculated between the object and 3Ds after 1 (in purple), 2 (in blue), 10 (in cyan), 100 (in green), and 1,000 (in black) cycles of iteration. (**K**) The plot of the CCC (between the iterative 3D and the object) against the cycles of the iteration. All 3Ds were low-pass filtered to 8 Å. Bars: 50 nm.

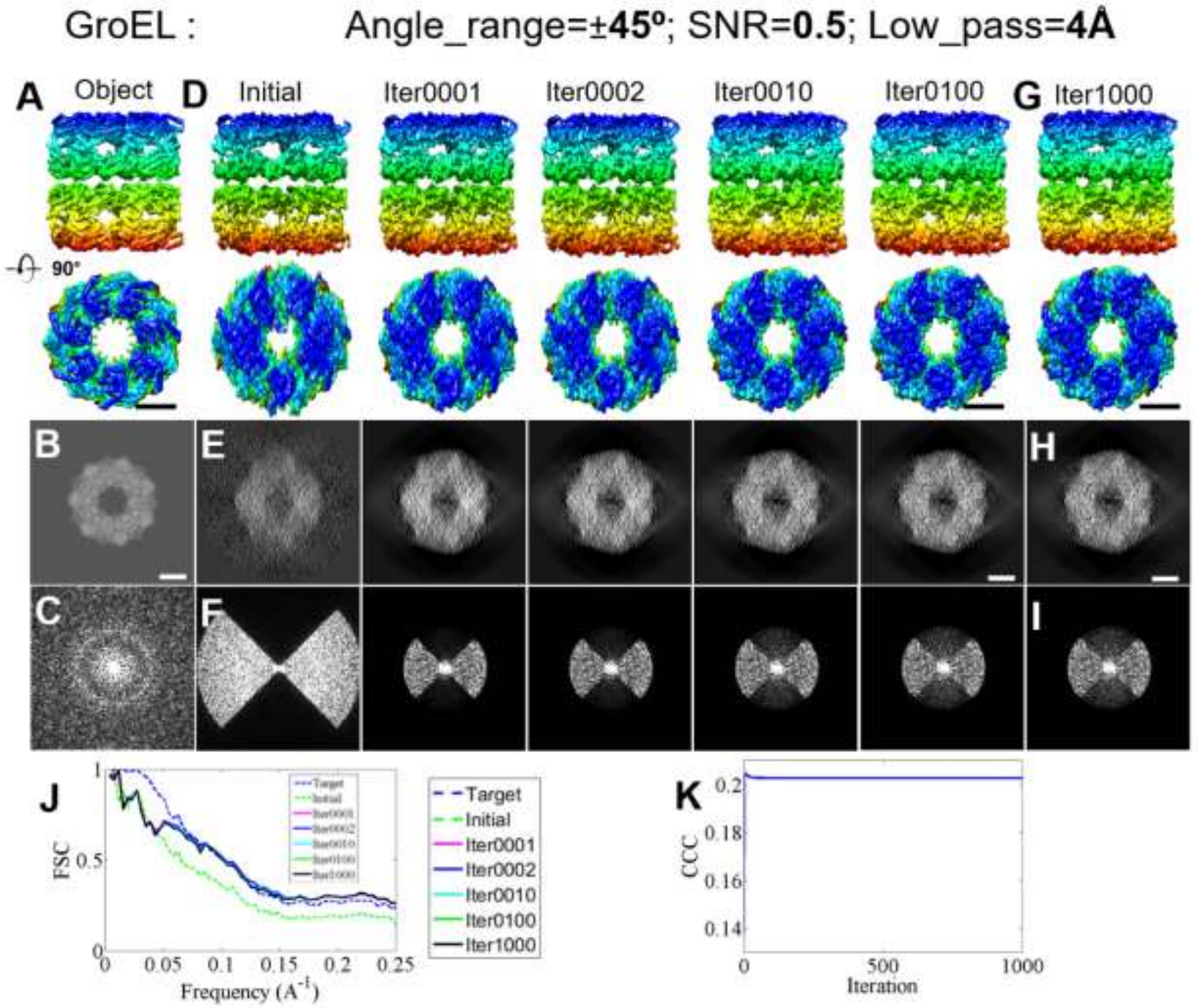

Supplementary Fig. 41 | The missing-wedge correction on a simulated 3D map of GroEL reconstructed from the low-pass filtered ±45° tilt series that with SNR=0.5 (A) A 3D object shown from two perpendicular views. The object was a generated from the crystal structure of GroEL, and the idea 3D was constructed from the tilt series of the noisy 2D projections (SNR=0.5) of the object from tilt angles in a range of ±90° in steps of 1.5°, while the initial 3D was reconstructed from angles of ±45° tilt series

after low-pass filtered to 4 Å. (**B**) The projection of the object on the X-Z plane, and (**C**) the corresponding Fourier transform. (**D**) The initial 3D and iterative 3D maps, shown from perpendicular views. (**E**) Their corresponding projections on the X-Z plane and the (**F**) Fourier transforms. The mask corresponding to ~3 times the molecular weight of GroEL was generated from the low-passed filtered object (~40 Å). (**G**) The final corrected 3D after 1,000 cycles of iteration (round 1, Rd_1), shown from two perpendicular views, and (**H**) the projection along the X-Z plane and the corresponding (**I**) Fourier transform. (**J**) FSC curves of the iterative 3Ds against the object. The blue dashed line is the ideal FSC curve, calculated between the ideal 3D and the object. The dashed green line is the initial FSC, calculated between the initial 3D and the object. The rest solid lines are the iterative FSC calculated between the object and 3Ds after 1 (in purple), 2 (in blue), 10 (in cyan), 100 (in green), and 1,000 (in black) cycles of iteration. (**K**) The plot of the CCC (between the iterative 3D and the object) against the cycles of the iteration. All 3Ds were low-pass filtered to 8 Å. Bars: 50 nm.

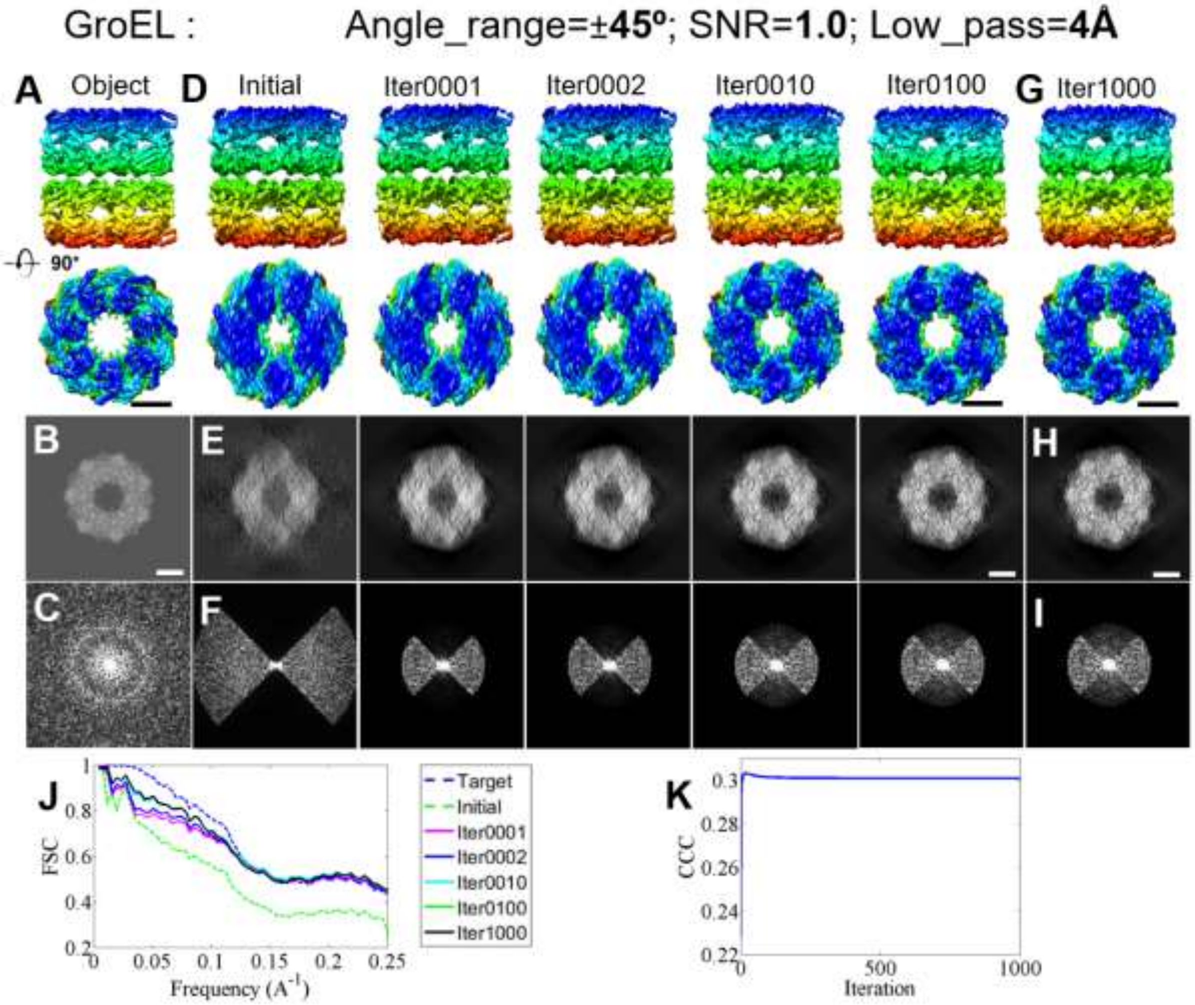

Supplementary Fig. 42 | The missing-wedge correction on a simulated 3D map of GroEL reconstructed from the low-pass filtered ±45° tilt series that with SNR=1.0 (A) A 3D object shown from two perpendicular views. The object was a generated from the crystal structure of GroEL, and the idea 3D was constructed from the tilt series of the noisy 2D projections (SNR=1.0) of the object from tilt angles

in a range of ±90° in steps of 1.5°, while the initial 3D was reconstructed from angles of ±45° tilt series after low-pass filtered to 4 Å. (**B**) The projection of the object on the X-Z plane, and (**C**) the corresponding Fourier transform. (**D**) The initial 3D and iterative 3D maps, shown from perpendicular views. (**E**) Their corresponding projections on the X-Z plane and the (**F**) Fourier transforms. The mask corresponding to ~3 times the molecular weight of GroEL was generated from the low-passed filtered object (~40 Å). (**G**) The final corrected 3D after 1,000 cycles of iteration (round 1, Rd_1), shown from two perpendicular views, and (**H**) the projection along the X-Z plane and the corresponding (**I**) Fourier transform. (**J**) FSC curves of the iterative 3Ds against the object. The blue dashed line is the ideal FSC curve, calculated between the ideal 3D and the object. The dashed green line is the initial FSC, calculated between the initial 3D and the object. The rest solid lines are the iterative FSC calculated between the object and 3Ds after 1 (in purple), 2 (in blue), 10 (in cyan), 100 (in green), and 1,000 (in black) cycles of iteration. (**K**) The plot of the CCC (between the iterative 3D and the object) against the cycles of the iteration. All 3Ds were low-pass filtered to 8 Å. Bars: 50 nm.

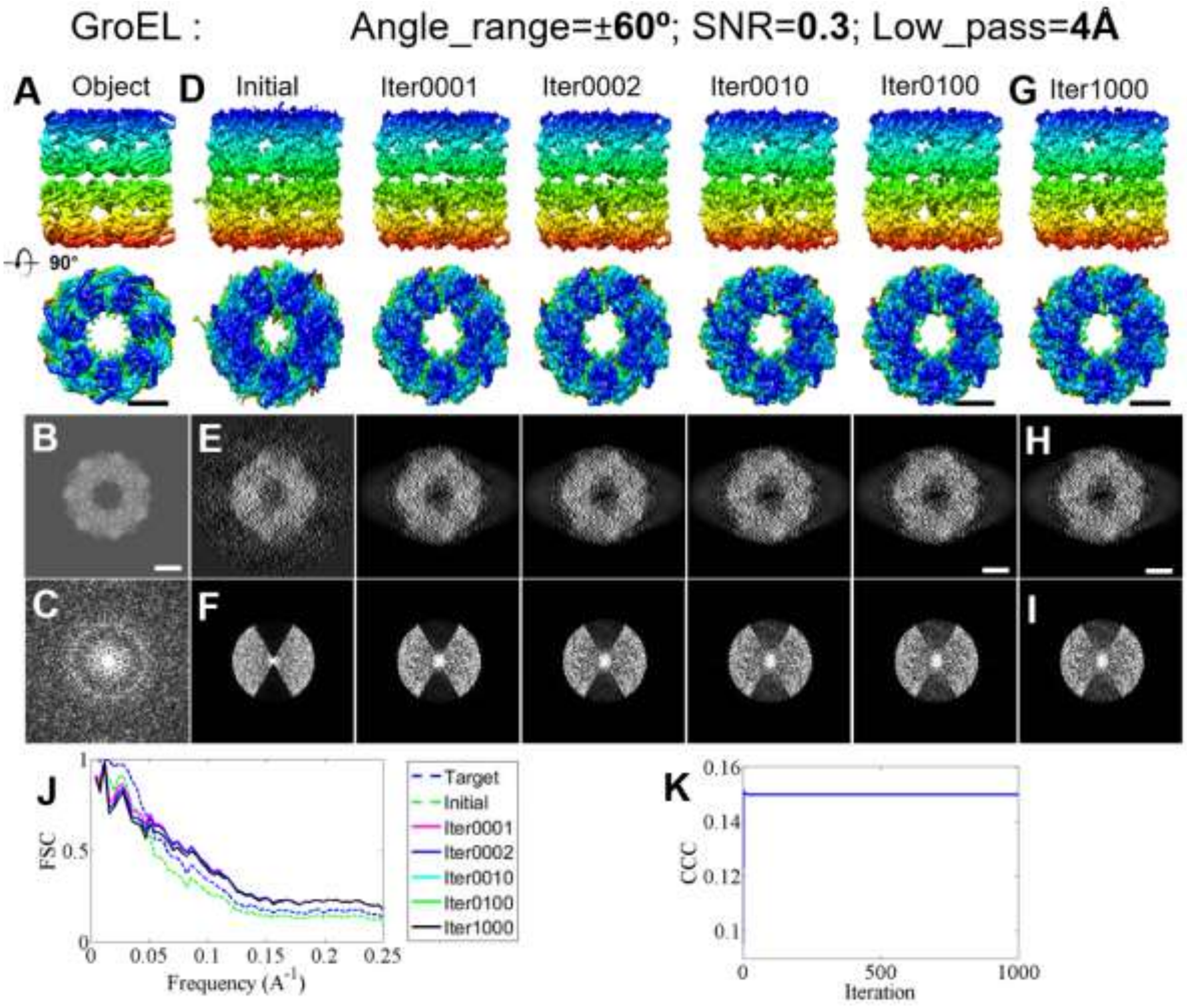

Supplementary Fig. 43 | The missing-wedge correction on a simulated 3D map of GroEL reconstructed from the low-pass filtered ±60° tilt series that with SNR=0.3 (A) A 3D object shown from two perpendicular views. The object was a generated from the crystal structure of GroEL, and the idea

3D was constructed from the tilt series of the noisy 2D projections (SNR=0.3) of the object from tilt angles in a range of ±90° in steps of 1.5°, while the initial 3D was reconstructed from angles of ±60° tilt series after low-pass filtered to 4 Å. (**B**) The projection of the object on the X-Z plane, and (**C**) the corresponding Fourier transform. (**D**) The initial 3D and iterative 3D maps, shown from perpendicular views. (**E**) Their corresponding projections on the X-Z plane and the (**F**) Fourier transforms. The mask corresponding to ~3 times the molecular weight of GroEL was generated from the low-passed filtered object (~40 Å). (**G**) The final corrected 3D after 1,000 cycles of iteration (round 1, Rd_1), shown from two perpendicular views, and (**H**) the projection along the X-Z plane and the corresponding (**I**) Fourier transform. (**J**) FSC curves of the iterative 3Ds against the object. The blue dashed line is the ideal FSC curve, calculated between the ideal 3D and the object. The dashed green line is the initial FSC, calculated between the initial 3D and the object. The rest solid lines are the iterative FSC calculated between the object and 3Ds after 1 (in purple), 2 (in blue), 10 (in cyan), 100 (in green), and 1,000 (in black) cycles of iteration. (**K**) The plot of the CCC (between the iterative 3D and the object) against the cycles of the iteration. All 3Ds were low-pass filtered to 8 Å. Bars: 50 nm.

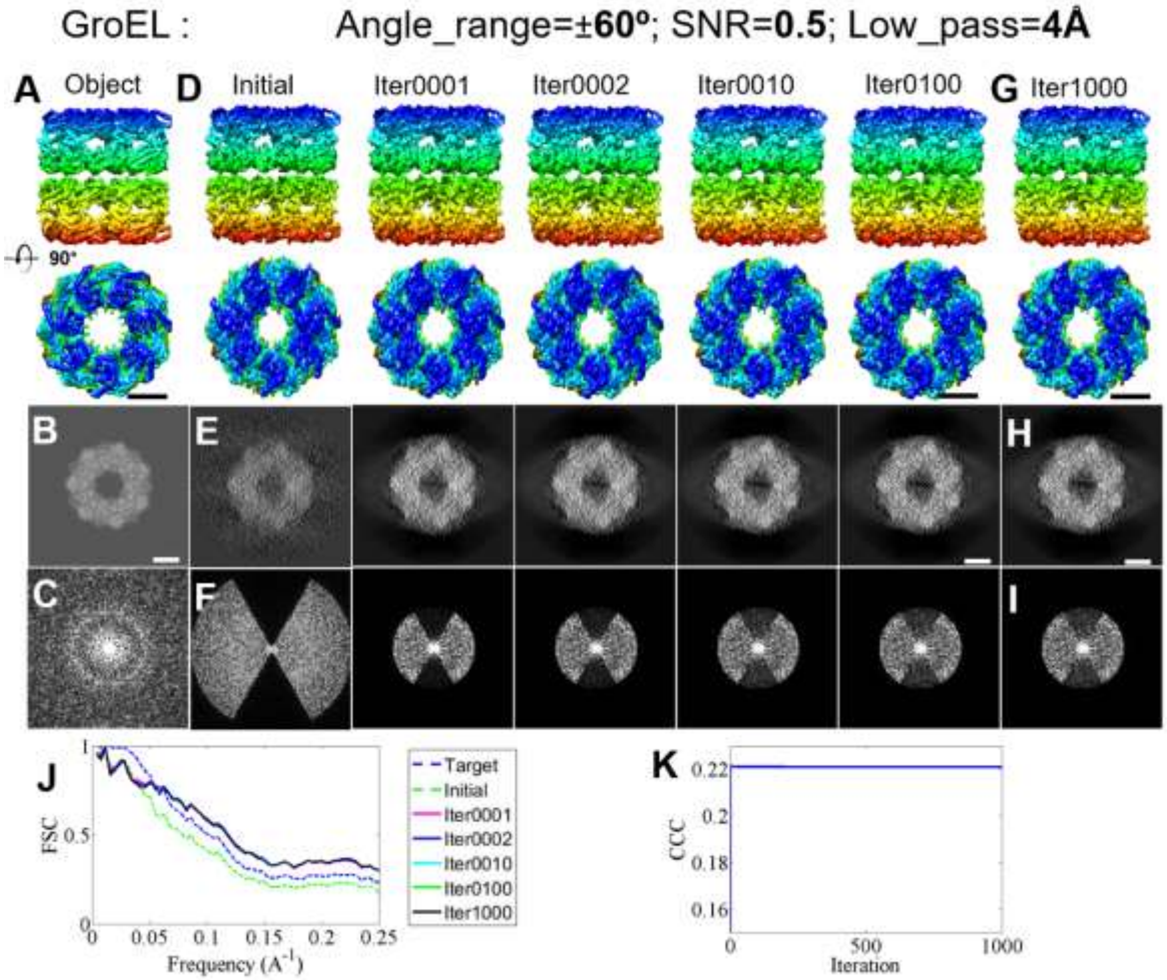

Supplementary Fig. 44 | The missing-wedge correction on a simulated 3D map of GroEL reconstructed from the low-pass filtered ±60° tilt series that with SNR=0.5 (A) A 3D object shown from two perpendicular views. The object was a generated from the crystal structure of GroEL, and the idea

3D was constructed from the tilt series of the noisy 2D projections (SNR=0.5) of the object from tilt angles in a range of ±90° in steps of 1.5°, while the initial 3D was reconstructed from angles of ±60° tilt series after low-pass filtered to 4 Å. (**B**) The projection of the object on the X-Z plane, and (**C**) the corresponding Fourier transform. (**D**) The initial 3D and iterative 3D maps, shown from perpendicular views. (**E**) Their corresponding projections on the X-Z plane and the (**F**) Fourier transforms. The mask corresponding to ~3 times the molecular weight of GroEL was generated from the low-passed filtered object (~40 Å). (**G**) The final corrected 3D after 1,000 cycles of iteration (round 1, Rd_1), shown from two perpendicular views, and (**H**) the projection along the X-Z plane and the corresponding (**I**) Fourier transform. (**J**) FSC curves of the iterative 3Ds against the object. The blue dashed line is the ideal FSC curve, calculated between the ideal 3D and the object. The dashed green line is the initial FSC, calculated between the initial 3D and the object. The rest solid lines are the iterative FSC calculated between the object and 3Ds after 1 (in purple), 2 (in blue), 10 (in cyan), 100 (in green), and 1,000 (in black) cycles of iteration. (**K**) The plot of the CCC (between the iterative 3D and the object) against the cycles of the iteration. All 3Ds were low-pass filtered to 8 Å. Bars: 50 nm.

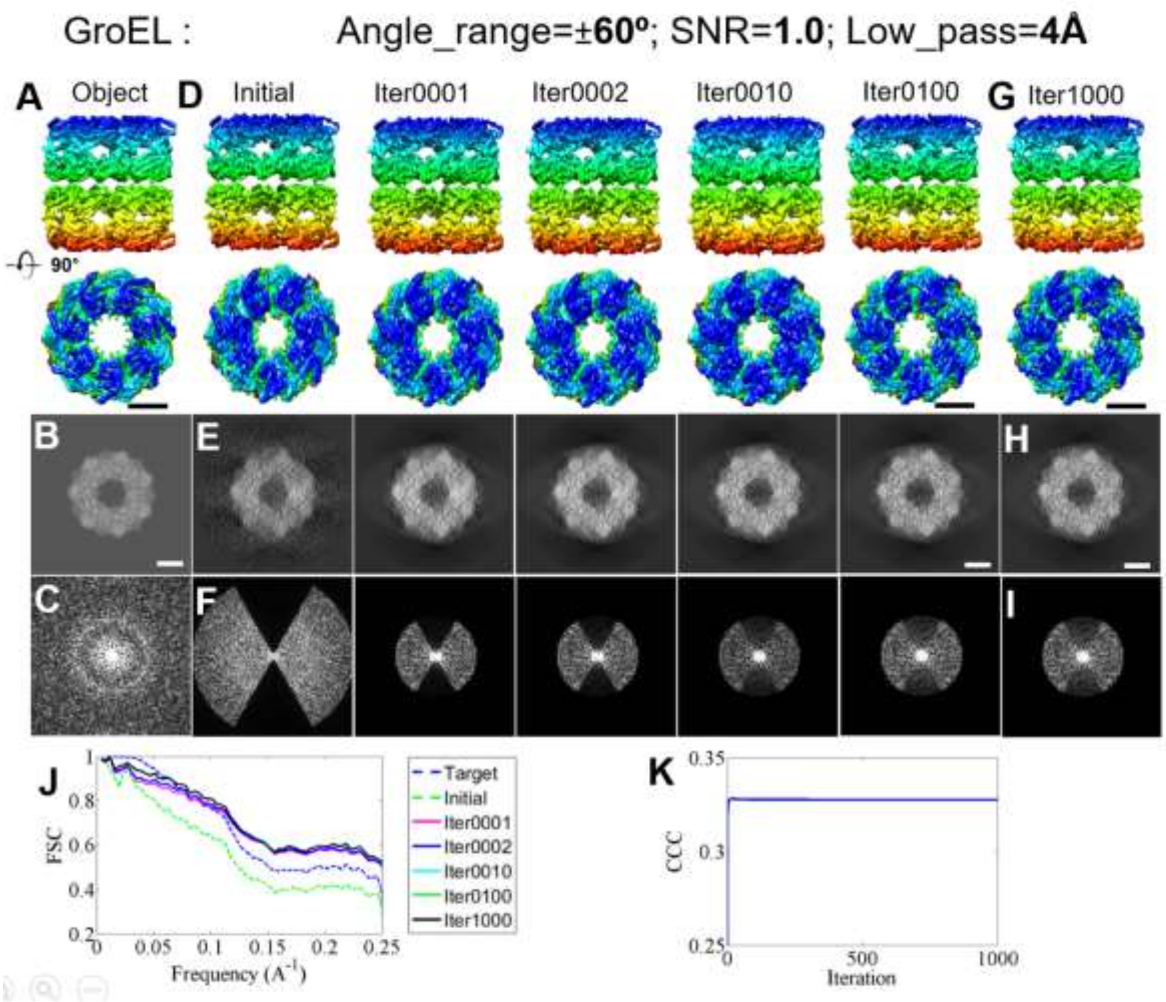

Supplementary Fig. 45 | The missing-wedge correction on a simulated 3D map of GroEL reconstructed from the low-pass filtered ±60° tilt series that with SNR=1.0 (A) A 3D object shown from two perpendicular views. The object was a generated from the crystal structure of GroEL, and the idea 3D was constructed from the tilt series of the noisy 2D projections (SNR=1.0) of the object from tilt angles in a range of ±90° in steps of 1.5°, while the initial 3D was reconstructed from angles of ±60° tilt series after low-pass filtered to 4 Å. (**B**) The projection of the object on the X-Z plane, and (**C**) the corresponding Fourier transform. (**D**) The initial 3D and iterative 3D maps, shown from perpendicular views. (**E**) Their corresponding projections on the X-Z plane and the (**F**) Fourier transforms. The mask corresponding to ~3 times the molecular weight of GroEL was generated from the low-passed filtered object (~40 Å). (**G**) The final corrected 3D after 1,000 cycles of iteration (round 1, Rd_1), shown from two perpendicular views, and (**H**) the projection along the X-Z plane and the corresponding (**I**) Fourier transform. (**J**) FSC curves of the iterative 3Ds against the object. The blue dashed line is the ideal FSC curve, calculated between the ideal 3D and the object. The dashed green line is the initial FSC, calculated between the initial 3D and the object. The rest solid lines are the iterative FSC calculated between the object and 3Ds after 1 (in purple), 2 (in blue), 10 (in cyan), 100 (in green), and 1,000 (in black) cycles of iteration. (**K**) The plot of the CCC (between the iterative 3D and the object) against the cycles of the iteration. All 3Ds were low-pass filtered to 8 Å. Bars: 50 nm.

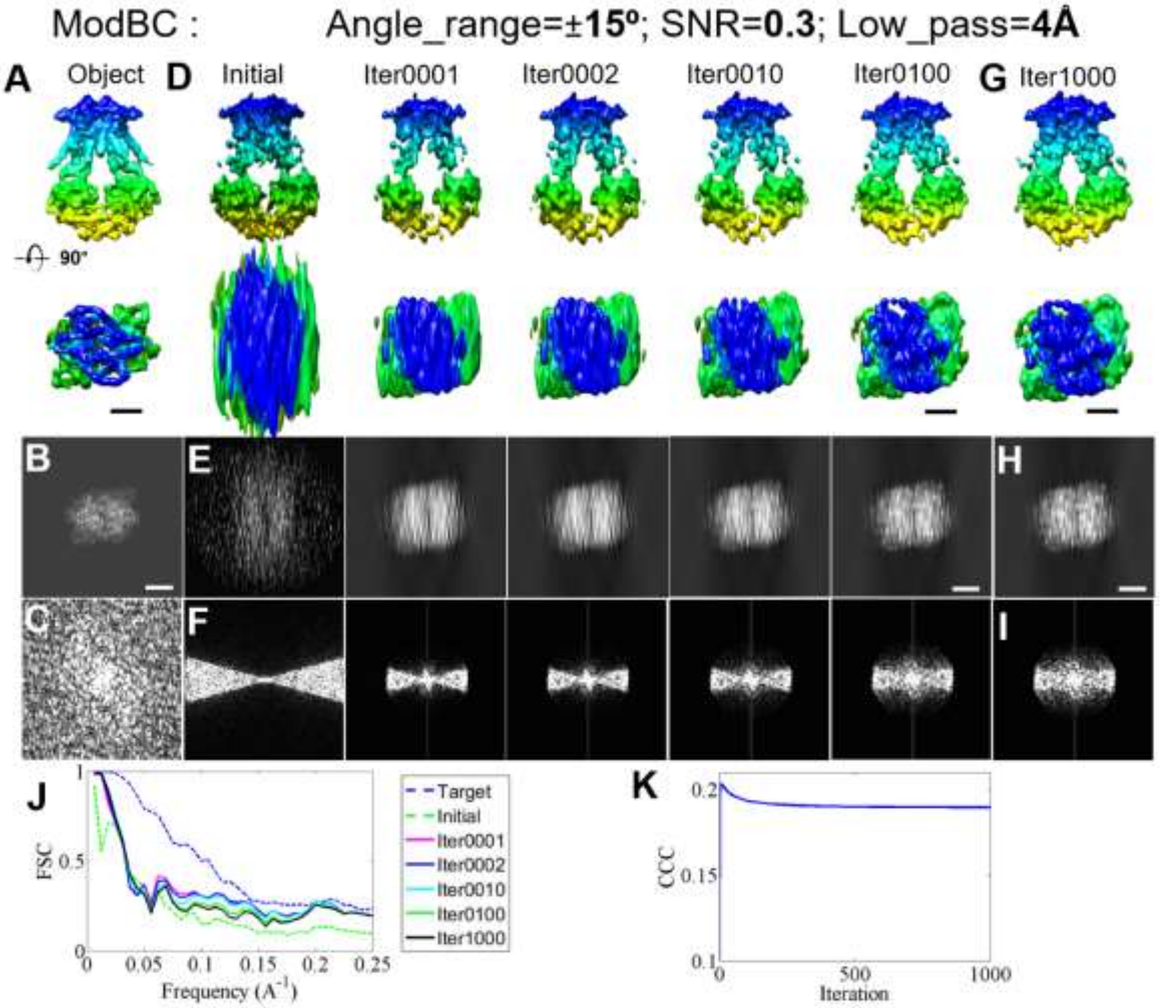

Supplementary Fig. 46 | The missing-wedge correction on a simulated 3D map of $ModB_2C_2$ reconstructed from the low-pass filtered ±15° tilt series that with SNR=0.3 (A) A 3D object shown from two perpendicular views. The object was a generated from the crystal structure of $ModB_2C_2$, and the idea 3D was constructed from the tilt series of the noisy 2D projections (SNR=0.3) of the object from tilt angles in a range of ±90° in steps of 1.5°, while the initial 3D was reconstructed from that within the angle range of ±15° tilt series after low-pass filtered to 4 Å. (**B**) The projection of the object on the X-Z plane, and (**C**) the corresponding Fourier transform. (**D**) The initial 3D and iterative 3D maps, shown from perpendicular views. (**E**) Their corresponding projections on the X-Z plane and the (**F**) Fourier transforms. The mask corresponding to ~3 times the molecular weight of $ModB_2C_2$ was generated from the low-passed filtered object (~40 Å). (**G**) The final corrected 3D after 1,000 cycles of iteration (round 1, Rd_1), shown from two perpendicular views, and (**H**) the projection along the X-Z plane and the corresponding (**I**) Fourier transform. (**J**) FSC curves of the iterative 3Ds against the object. The blue dashed line is the ideal FSC curve, calculated between the ideal 3D and the object. The dashed green line is the initial FSC, calculated between the initial 3D and the object. The rest solid lines are the iterative FSC calculated between the object and 3Ds after 1 (in purple), 2 (in blue), 10 (in cyan), 100 (in green), and 1,000 (in black) cycles of iteration.

(**K**) The plot of the CCC (between the iterative 3D and the object) against the cycles of the iteration. All 3Ds were low-pass filtered to 8 Å. Bars: 20 nm.

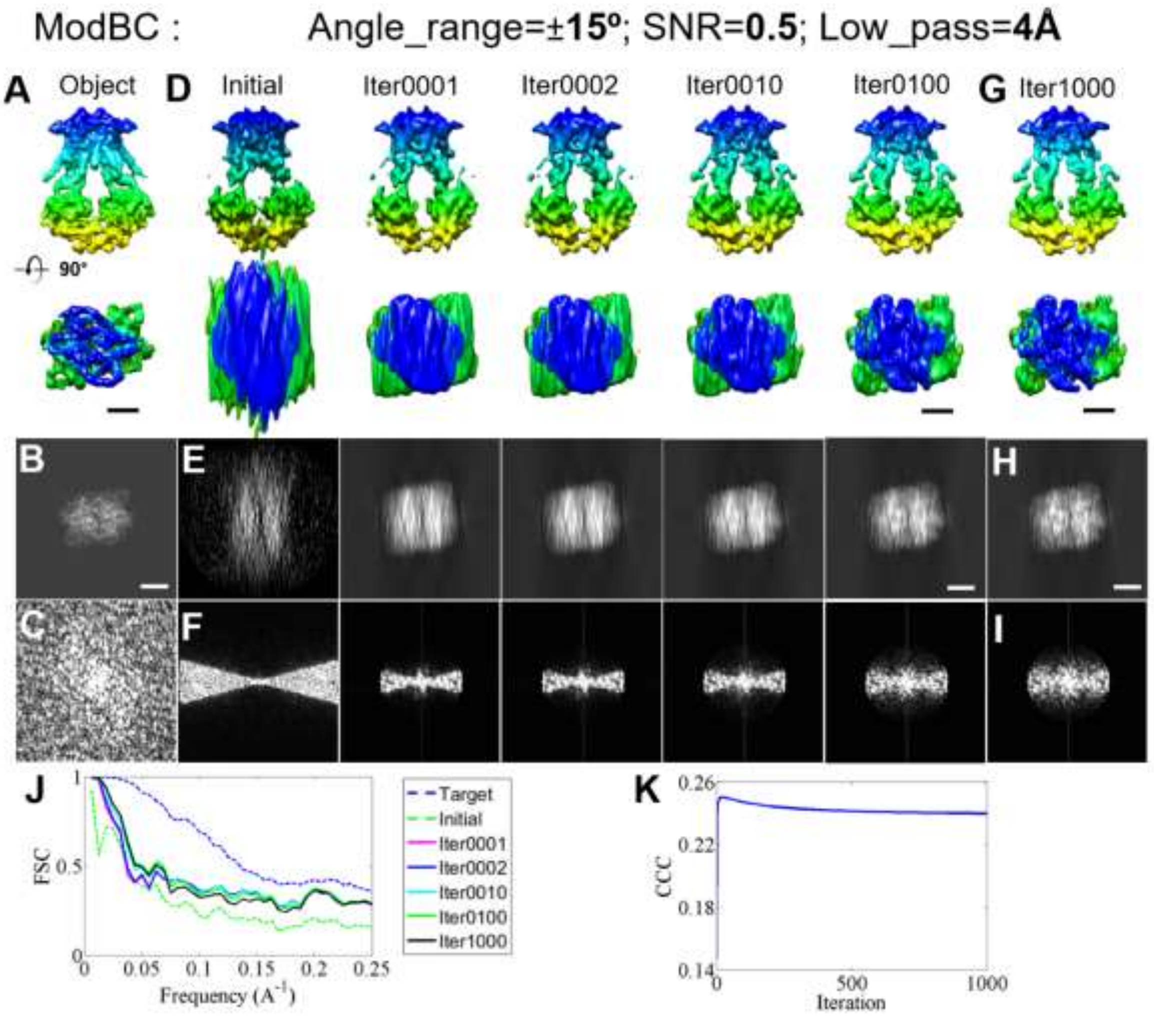

Supplementary Fig. 47 | The missing-wedge correction on a simulated 3D map of ModB$_2$C$_2$ reconstructed from the low-pass filtered ±15° tilt series that with SNR=0.5 (A) A 3D object shown from two perpendicular views. The object was a generated from the crystal structure of ModB$_2$C$_2$, and the idea 3D was constructed from the tilt series of the noisy 2D projections (SNR=0.5) of the object from tilt angles in a range of ±90° in steps of 1.5°, while the initial 3D was reconstructed from that within the angle range of ±15° tilt series after low-pass filtered to 4 Å. (**B**) The projection of the object on the X-Z plane, and (**C**) the corresponding Fourier transform. (**D**) The initial 3D and iterative 3D maps, shown from perpendicular views. (**E**) Their corresponding projections on the X-Z plane and the (**F**) Fourier transforms. The mask corresponding to ~3 times the molecular weight of ModB$_2$C$_2$ was generated from the low-passed filtered object (~40 Å). (**G**) The final corrected 3D after 1,000 cycles of iteration (round 1, Rd_1), shown from two perpendicular views, and (**H**) the projection along the X-Z plane and the corresponding (**I**) Fourier transform. (**J**) FSC curves of the iterative 3Ds against the object. The blue dashed line is the ideal FSC curve, calculated between the ideal 3D and the object. The dashed green line is the initial FSC, calculated between the initial 3D and the object. The rest solid lines are the iterative FSC calculated between the object

and 3Ds after 1 (in purple), 2 (in blue), 10 (in cyan), 100 (in green), and 1,000 (in black) cycles of iteration. (**K**) The plot of the CCC (between the iterative 3D and the object) against the cycles of the iteration. All 3Ds were low-pass filtered to 8 Å. Bars: 20 nm.

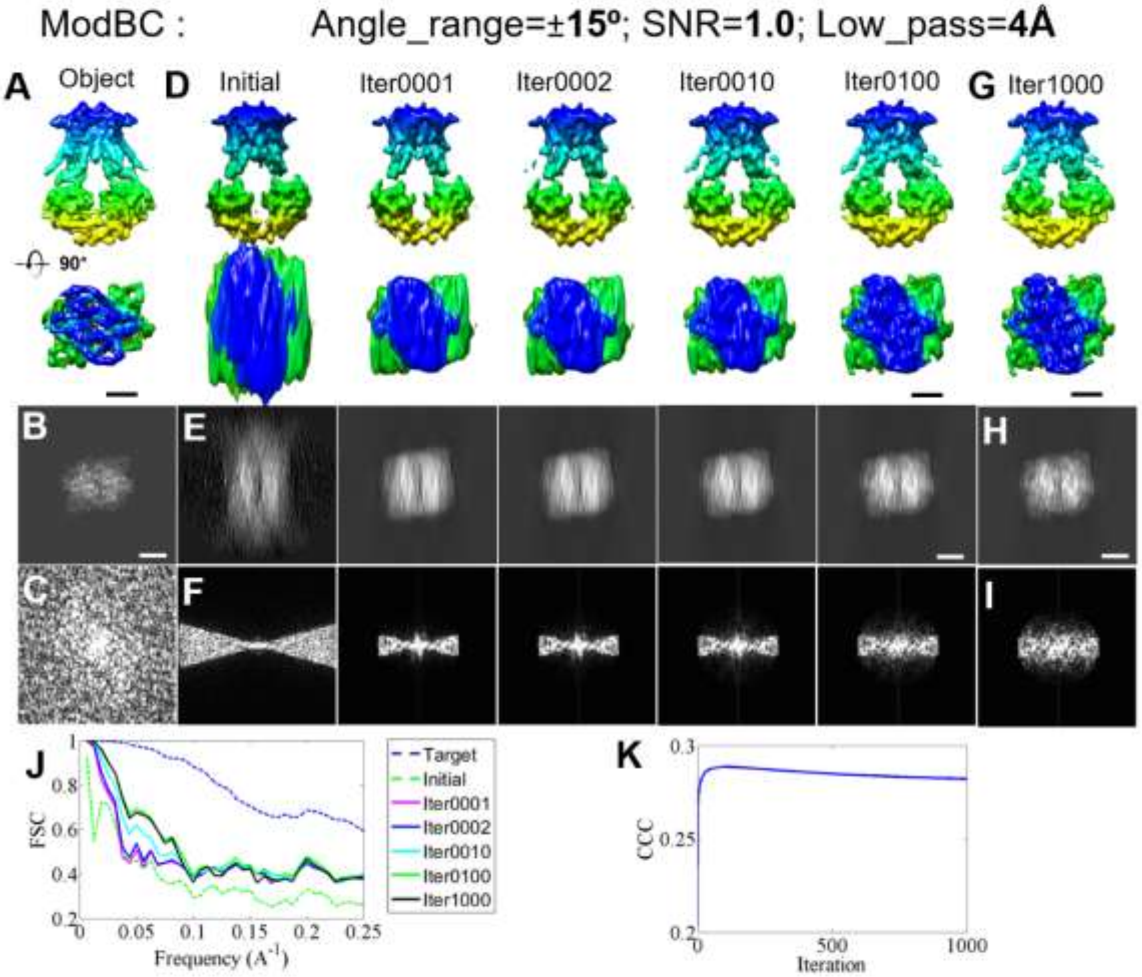

Supplementary Fig. 48 | The missing-wedge correction on a simulated 3D map of $ModB_2C_2$ reconstructed from the low-pass filtered ±15° tilt series that with SNR=1.0 (A) A 3D object shown from two perpendicular views. The object was a generated from the crystal structure of $ModB_2C_2$, and the idea 3D was constructed from the tilt series of the noisy 2D projections (SNR=1.0) of the object from tilt angles in a range of ±90° in steps of 1.5°, while the initial 3D was reconstructed from that within the angle range of ±15° tilt series after low-pass filtered to 4 Å. (**B**) The projection of the object on the X-Z plane, and (**C**) the corresponding Fourier transform. (**D**) The initial 3D and iterative 3D maps, shown from perpendicular views. (**E**) Their corresponding projections on the X-Z plane and the (**F**) Fourier transforms. The mask corresponding to ~3 times the molecular weight of $ModB_2C_2$ was generated from the low-passed filtered object (~40 Å). (**G**) The final corrected 3D after 1,000 cycles of iteration (round 1, Rd_1), shown from two perpendicular views, and (**H**) the projection along the X-Z plane and the corresponding (**I**) Fourier transform. (**J**) FSC curves of the iterative 3Ds against the object. The blue dashed line is the ideal FSC curve, calculated between the ideal 3D and the object. The dashed green line is the initial FSC, calculated between the initial 3D and the object. The rest solid lines are the iterative FSC calculated between the object

and 3Ds after 1 (in purple), 2 (in blue), 10 (in cyan), 100 (in green), and 1,000 (in black) cycles of iteration. (**K**) The plot of the CCC (between the iterative 3D and the object) against the cycles of the iteration. All 3Ds were low-pass filtered to 8 Å. Bars: 20 nm.

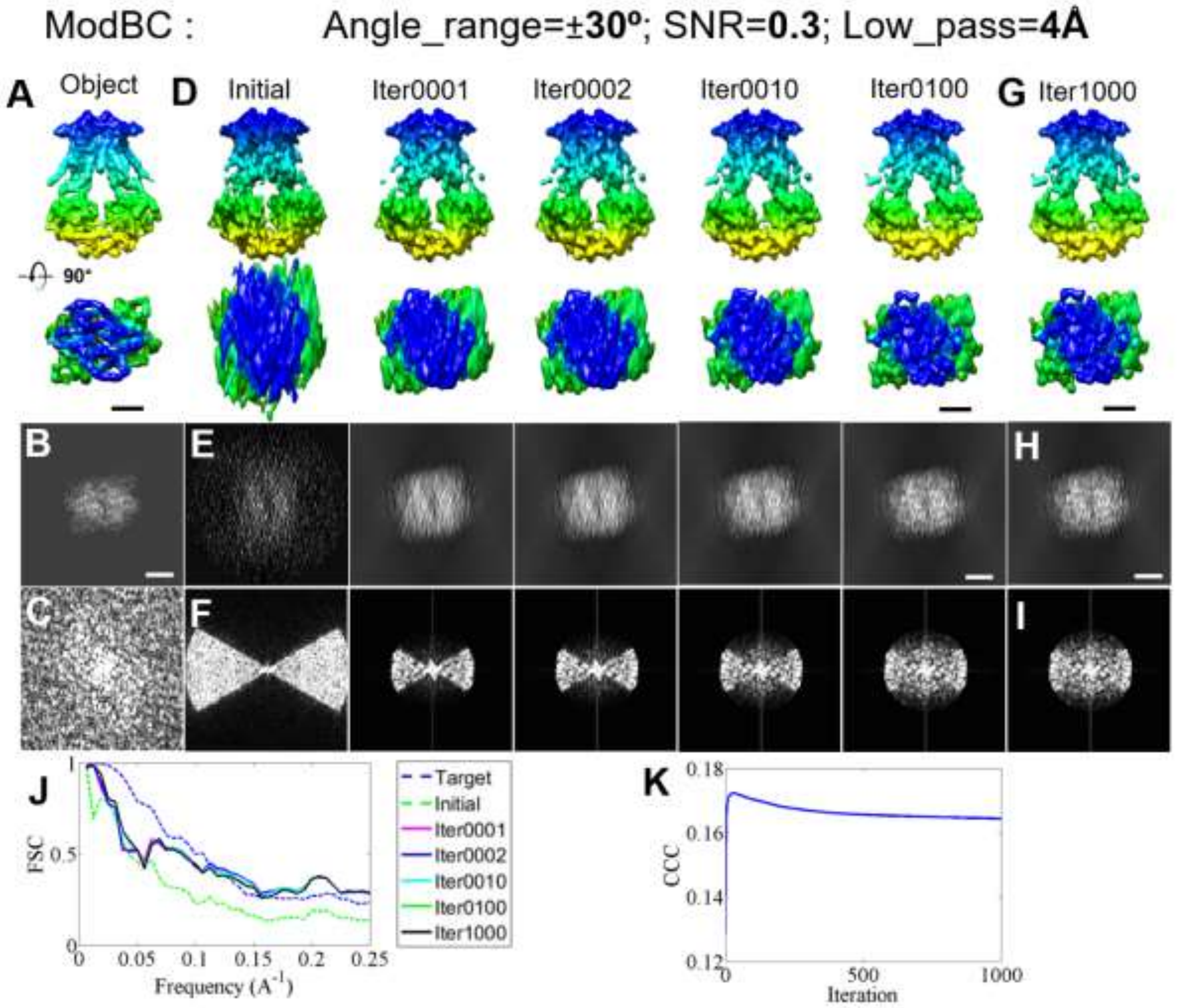

Supplementary Fig. 49 | The missing-wedge correction on a simulated 3D map of ModB$_2$C$_2$ reconstructed from the low-pass filtered ±30° tilt series that with SNR=0.3 (A) A 3D object shown from two perpendicular views. The object was a generated from the crystal structure of ModB$_2$C$_2$, and the idea 3D was constructed from the tilt series of the noisy 2D projections (SNR=0.3) of the object from tilt angles in a range of ±90° in steps of 1.5°, while the initial 3D was reconstructed from angles of ±30° tilt series after low-pass filtered to 4 Å. (**B**) The projection of the object on the X-Z plane, and (**C**) the corresponding Fourier transform. (**D**) The initial 3D and iterative 3D maps, shown from perpendicular views. (**E**) Their corresponding projections on the X-Z plane and the (**F**) Fourier transforms. The mask corresponding to ~3 times the molecular weight of ModB$_2$C$_2$ was generated from the low-passed filtered object (~40 Å). (**G**) The final corrected 3D after 1,000 cycles of iteration (round 1, Rd_1), shown from two perpendicular views, and (**H**) the projection along the X-Z plane and the corresponding (**I**) Fourier transform. (**J**) FSC curves of the iterative 3Ds against the object. The blue dashed line is the ideal FSC

curve, calculated between the ideal 3D and the object. The dashed green line is the initial FSC, calculated between the initial 3D and the object. The rest solid lines are the iterative FSC calculated between the object and 3Ds after 1 (in purple), 2 (in blue), 10 (in cyan), 100 (in green), and 1,000 (in black) cycles of iteration. (**K**) The plot of the CCC (between the iterative 3D and the object) against the cycles of the iteration. All 3Ds were low-pass filtered to 8 Å. Bars: 20 nm.

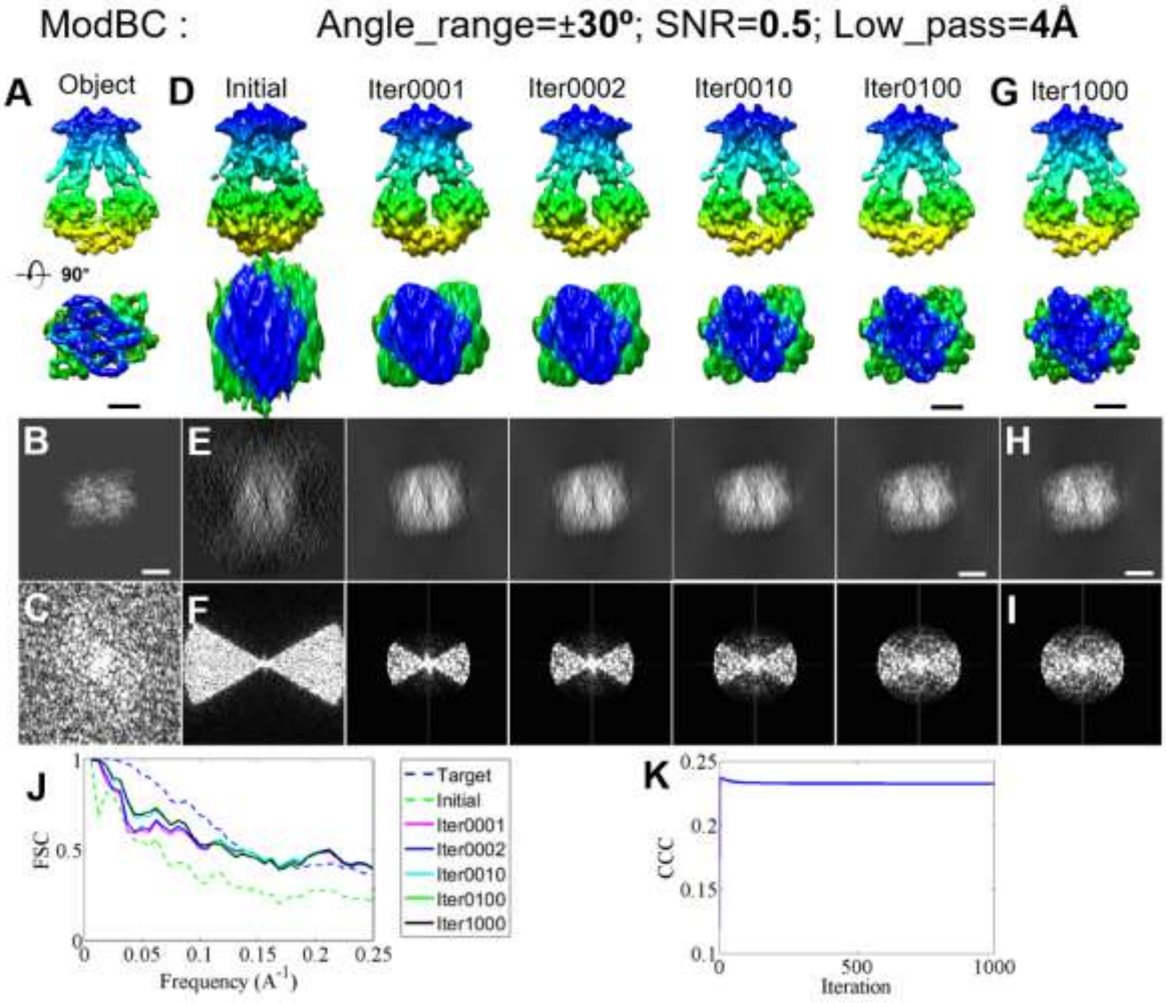

Supplementary Fig. 50 | The missing-wedge correction on a simulated 3D map of $ModB_2C_2$ reconstructed from the low-pass filtered ±30° tilt series that with SNR=0.5 (A) A 3D object shown from two perpendicular views. The object was a generated from the crystal structure of $ModB_2C_2$, and the idea 3D was constructed from the tilt series of the noisy 2D projections (SNR=0.5) of the object from tilt angles in a range of ±90° in steps of 1.5°, while the initial 3D was reconstructed from angles of ±30° tilt series after low-pass filtered to 4 Å. (**B**) The projection of the object on the X-Z plane, and (**C**) the corresponding Fourier transform. (**D**) The initial 3D and iterative 3D maps, shown from perpendicular views. (**E**) Their corresponding projections on the X-Z plane and the (**F**) Fourier transforms. The mask corresponding to ~3 times the molecular weight of $ModB_2C_2$ was generated from the low-passed filtered object (~40 Å). (**G**) The final corrected 3D after 1,000 cycles of iteration (round 1, Rd_1), shown from two perpendicular views, and (**H**) the projection along the X-Z plane and the corresponding (**I**) Fourier

transform. (**J**) FSC curves of the iterative 3Ds against the object. The blue dashed line is the ideal FSC curve, calculated between the ideal 3D and the object. The dashed green line is the initial FSC, calculated between the initial 3D and the object. The rest solid lines are the iterative FSC calculated between the object and 3Ds after 1 (in purple), 2 (in blue), 10 (in cyan), 100 (in green), and 1,000 (in black) cycles of iteration. (**K**) The plot of the CCC (between the iterative 3D and the object) against the cycles of the iteration. All 3Ds were low-pass filtered to 8 Å. Bars: 20 nm.

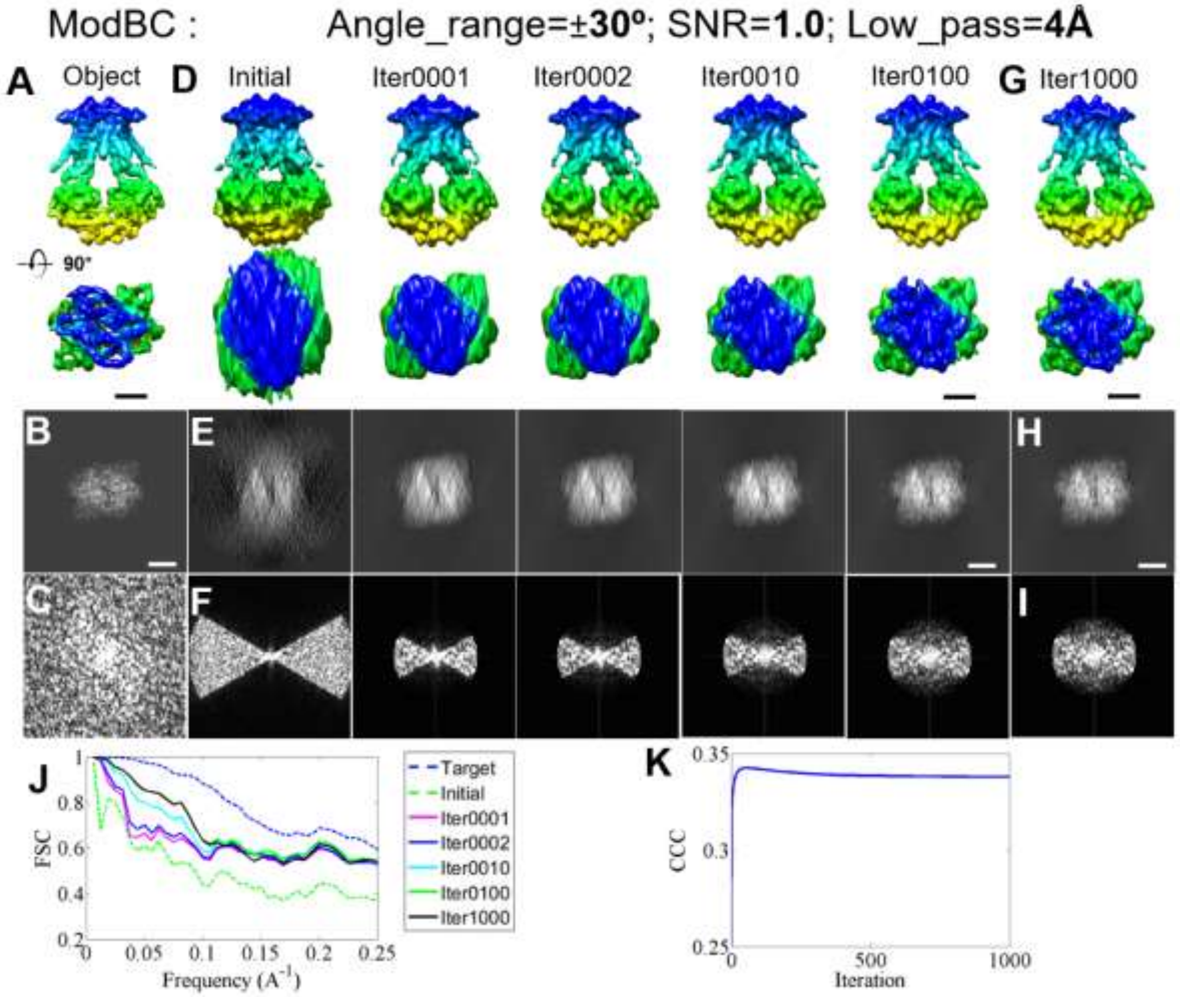

Supplementary Fig. 51 | The missing-wedge correction on a simulated 3D map of $ModB_2C_2$ reconstructed from the low-pass filtered ±30° tilt series that with SNR=1.0 (A) A 3D object shown from two perpendicular views. The object was a generated from the crystal structure of $ModB_2C_2$, and the idea 3D was constructed from the tilt series of the noisy 2D projections (SNR=1.0) of the object from tilt angles in a range of ±90° in steps of 1.5°, while the initial 3D was reconstructed from angles of ±30° tilt series after low-pass filtered to 4 Å. (**B**) The projection of the object on the X-Z plane, and (**C**) the corresponding Fourier transform. (**D**) The initial 3D and iterative 3D maps, shown from perpendicular views. (**E**) Their corresponding projections on the X-Z plane and the (**F**) Fourier transforms. The mask corresponding to ~3 times the molecular weight of $ModB_2C_2$ was generated from the low-passed filtered object (~40 Å). (**G**) The final corrected 3D after 1,000 cycles of iteration (round 1, Rd_1), shown from two

perpendicular views, and (**H**) the projection along the X-Z plane and the corresponding (**I**) Fourier transform. (**J**) FSC curves of the iterative 3Ds against the object. The blue dashed line is the ideal FSC curve, calculated between the ideal 3D and the object. The dashed green line is the initial FSC, calculated between the initial 3D and the object. The rest solid lines are the iterative FSC calculated between the object and 3Ds after 1 (in purple), 2 (in blue), 10 (in cyan), 100 (in green), and 1,000 (in black) cycles of iteration. (**K**) The plot of the CCC (between the iterative 3D and the object) against the cycles of the iteration. All 3Ds were low-pass filtered to 8 Å. Bars: 20 nm.

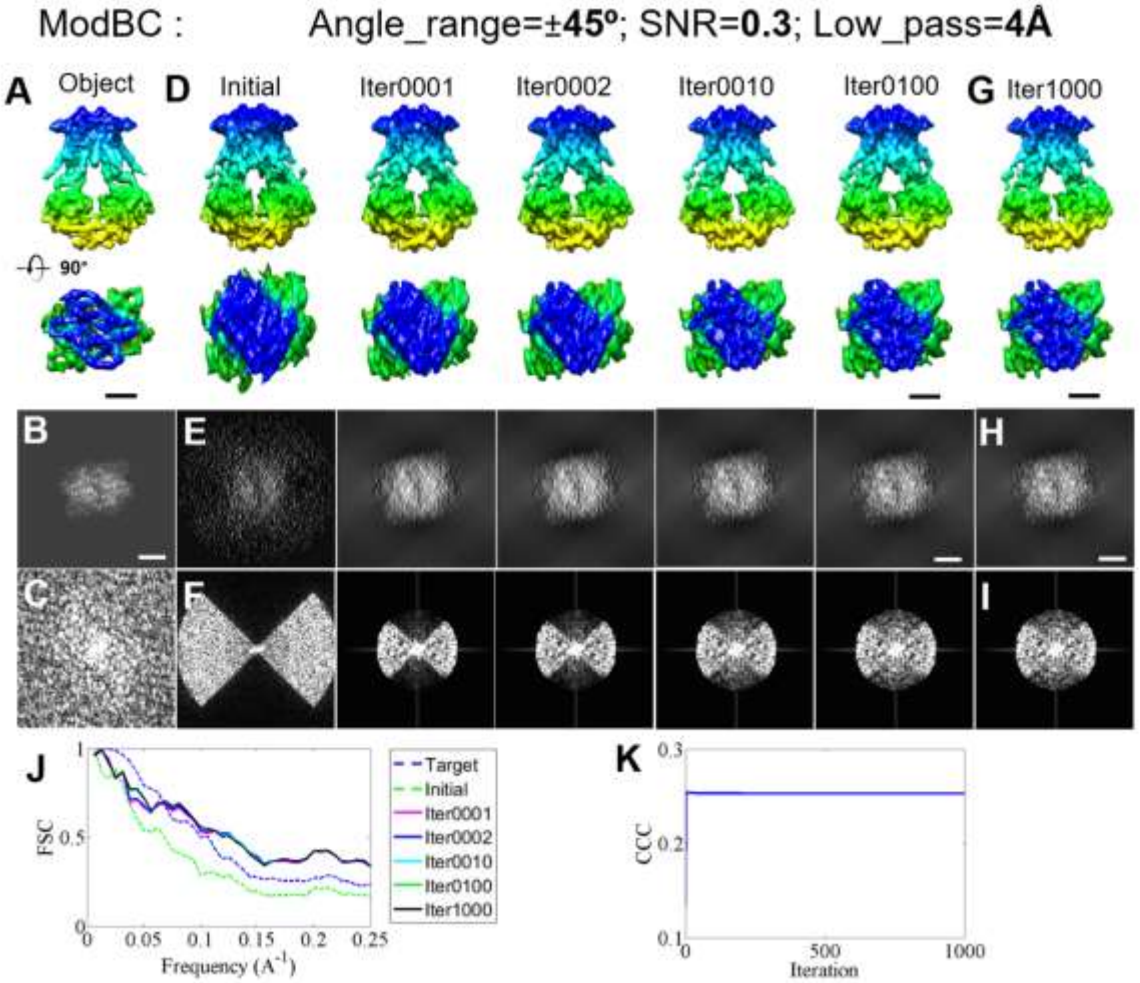

Supplementary Fig. 52 | The missing-wedge correction on a simulated 3D map of $ModB_2C_2$ reconstructed from the low-pass filtered ±45° tilt series that with SNR=0.3 (A) A 3D object shown from two perpendicular views. The object was a generated from the crystal structure of $ModB_2C_2$, and the idea 3D was constructed from the tilt series of the noisy 2D projections (SNR=0.3) of the object from tilt angles in a range of ±90° in steps of 1.5°, while the initial 3D was reconstructed from angles of ±45° tilt series after low-pass filtered to 4 Å. (**B**) The projection of the object on the X-Z plane, and (**C**) the corresponding Fourier transform. (**D**) The initial 3D and iterative 3D maps, shown from perpendicular views. (**E**) Their corresponding projections on the X-Z plane and the (**F**) Fourier transforms. The mask corresponding to ~3 times the molecular weight of $ModB_2C_2$ was generated from the low-passed filtered object (~40 Å). (**G**) The final corrected 3D after 1,000 cycles of iteration (round 1, Rd_1), shown from two

perpendicular views, and (**H**) the projection along the X-Z plane and the corresponding (**I**) Fourier transform. (**J**) FSC curves of the iterative 3Ds against the object. The blue dashed line is the ideal FSC curve, calculated between the ideal 3D and the object. The dashed green line is the initial FSC, calculated between the initial 3D and the object. The rest solid lines are the iterative FSC calculated between the object and 3Ds after 1 (in purple), 2 (in blue), 10 (in cyan), 100 (in green), and 1,000 (in black) cycles of iteration. (**K**) The plot of the CCC (between the iterative 3D and the object) against the cycles of the iteration. All 3Ds were low-pass filtered to 8 Å. Bars: 20 nm.

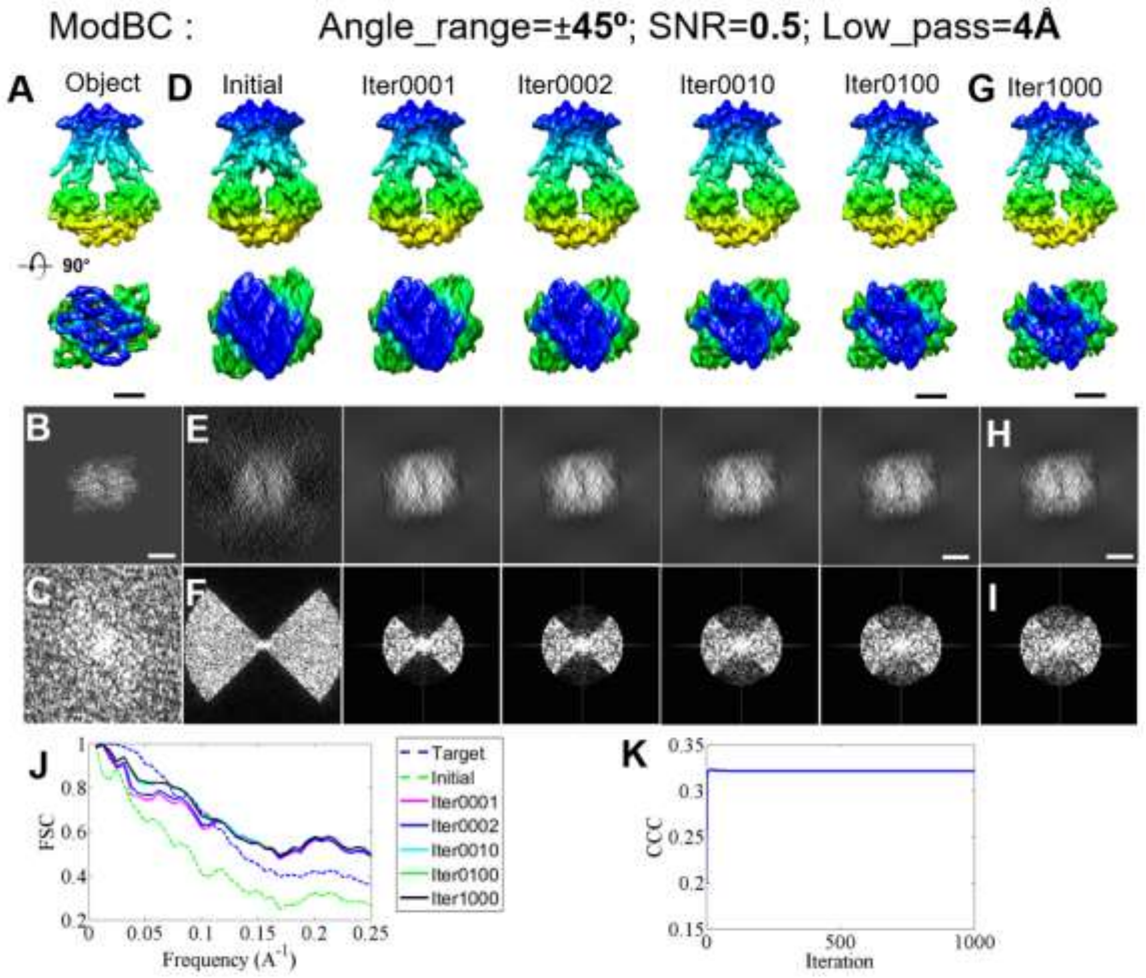

Supplementary Fig. 53 | The missing-wedge correction on a simulated 3D map of $ModB_2C_2$ reconstructed from the low-pass filtered ±45° tilt series that with SNR=0.5 (A) A 3D object shown from two perpendicular views. The object was a generated from the crystal structure of $ModB_2C_2$, and the idea 3D was constructed from the tilt series of the noisy 2D projections (SNR=0.5) of the object from tilt angles in a range of ±90° in steps of 1.5°, while the initial 3D was reconstructed from angles of ±45° tilt series after low-pass filtered to 4 Å. (**B**) The projection of the object on the X-Z plane, and (**C**) the corresponding Fourier transform. (**D**) The initial 3D and iterative 3D maps, shown from perpendicular views. (**E**) Their corresponding projections on the X-Z plane and the (**F**) Fourier transforms. The mask corresponding to ~3 times the molecular weight of $ModB_2C_2$ was generated from the low-passed filtered

object (~40 Å). (**G**) The final corrected 3D after 1,000 cycles of iteration (round 1, Rd_1), shown from two perpendicular views, and (**H**) the projection along the X-Z plane and the corresponding (**I**) Fourier transform. (**J**) FSC curves of the iterative 3Ds against the object. The blue dashed line is the ideal FSC curve, calculated between the ideal 3D and the object. The dashed green line is the initial FSC, calculated between the initial 3D and the object. The rest solid lines are the iterative FSC calculated between the object and 3Ds after 1 (in purple), 2 (in blue), 10 (in cyan), 100 (in green), and 1,000 (in black) cycles of iteration. (**K**) The plot of the CCC (between the iterative 3D and the object) against the cycles of the iteration. All 3Ds were low-pass filtered to 8 Å. Bars: 20 nm.

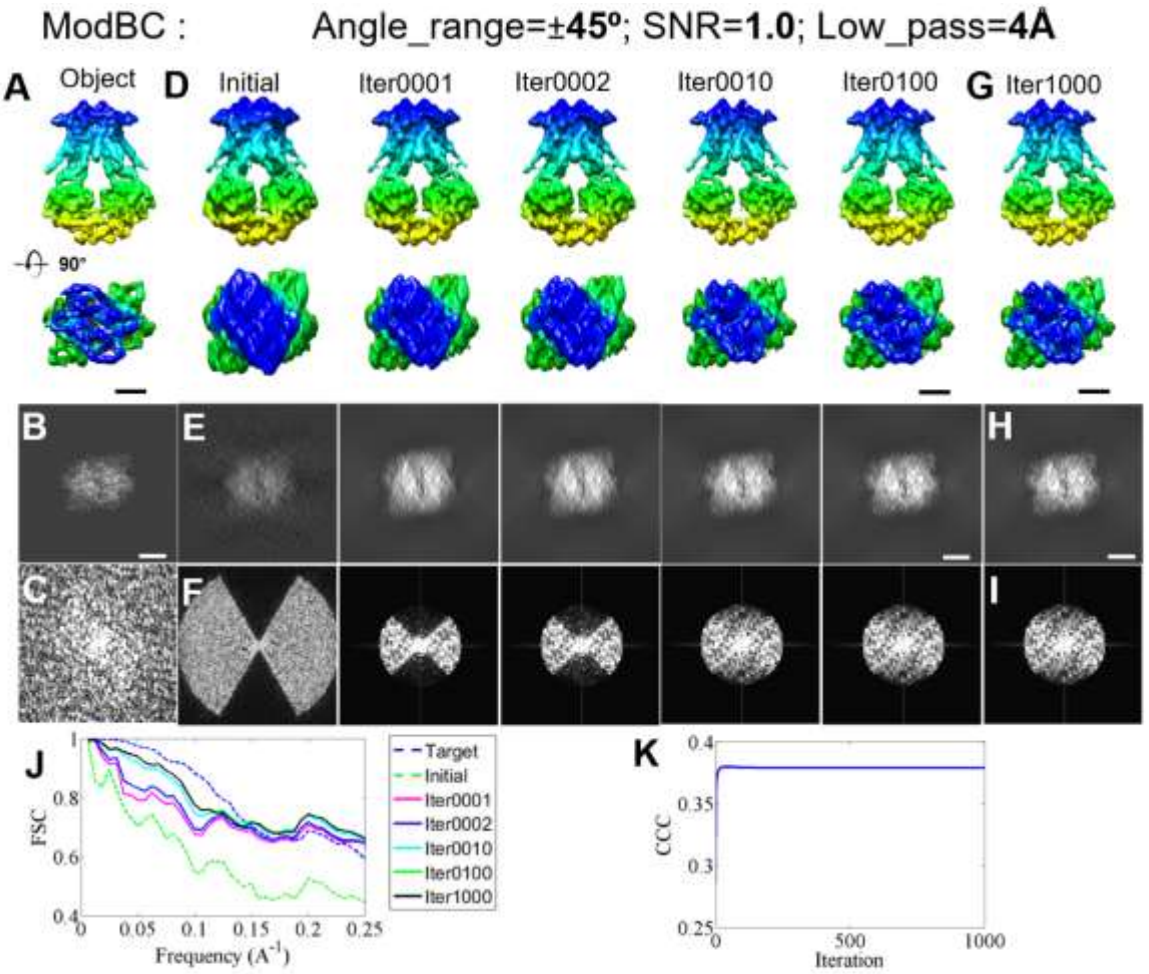

Supplementary Fig. 54 | The missing-wedge correction on a simulated 3D map of $ModB_2C_2$ reconstructed from the low-pass filtered ±45° tilt series that with SNR=1.0 (A) A 3D object shown from two perpendicular views. The object was a generated from the crystal structure of $ModB_2C_2$, and the idea 3D was constructed from the tilt series of the noisy 2D projections (SNR=1.0) of the object from tilt angles in a range of ±90° in steps of 1.5°, while the initial 3D was reconstructed from angles of ±45° tilt series after low-pass filtered to 4 Å. (**B**) The projection of the object on the X-Z plane, and (**C**) the corresponding Fourier transform. (**D**) The initial 3D and iterative 3D maps, shown from perpendicular views. (**E**) Their corresponding projections on the X-Z plane and the (**F**) Fourier transforms. The mask

corresponding to ~3 times the molecular weight of $ModB_2C_2$ was generated from the low-passed filtered object (~40 Å). (**G**) The final corrected 3D after 1,000 cycles of iteration (round 1, Rd_1), shown from two perpendicular views, and (**H**) the projection along the X-Z plane and the corresponding (**I**) Fourier transform. (**J**) FSC curves of the iterative 3Ds against the object. The blue dashed line is the ideal FSC curve, calculated between the ideal 3D and the object. The dashed green line is the initial FSC, calculated between the initial 3D and the object. The rest solid lines are the iterative FSC calculated between the object and 3Ds after 1 (in purple), 2 (in blue), 10 (in cyan), 100 (in green), and 1,000 (in black) cycles of iteration. (**K**) The plot of the CCC (between the iterative 3D and the object) against the cycles of the iteration. All 3Ds were low-pass filtered to 8 Å. Bars: 20 nm.

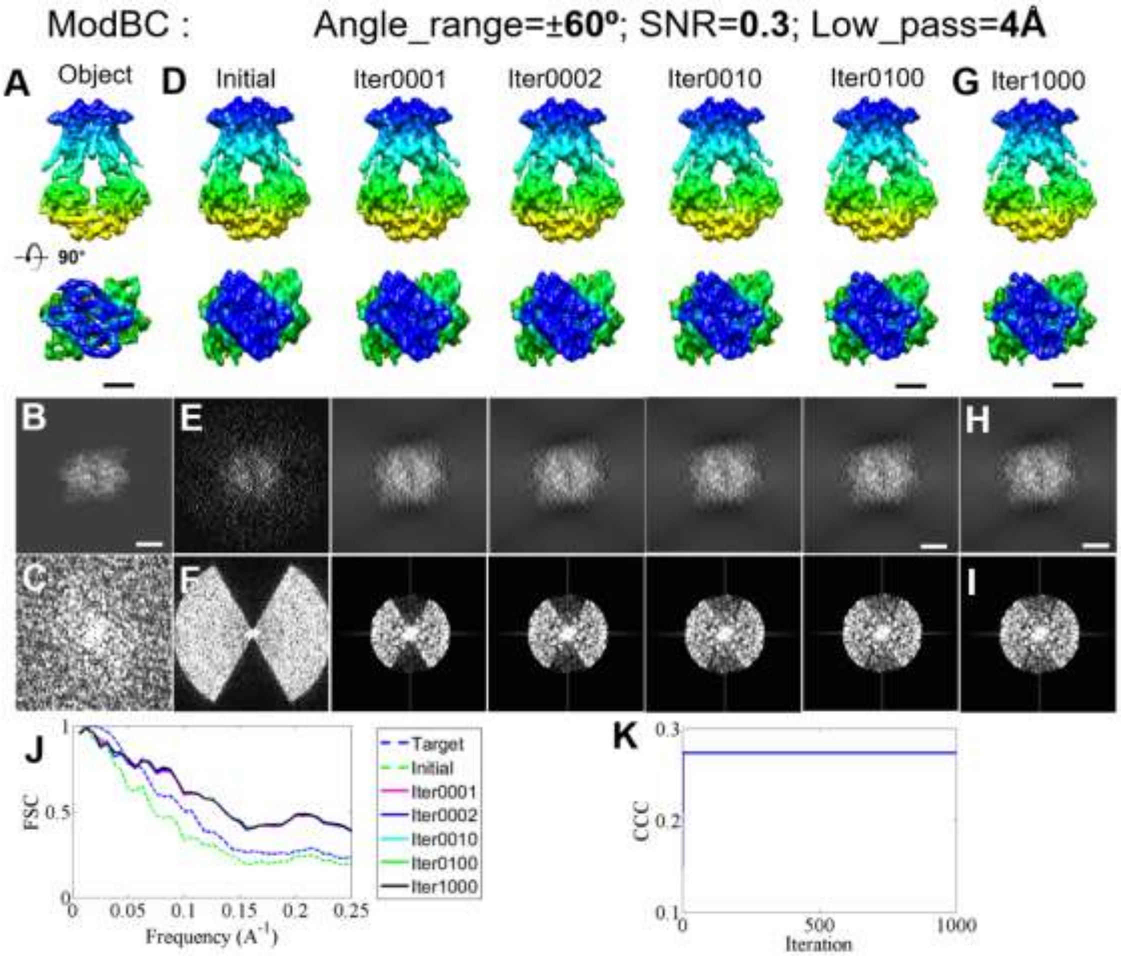

Supplementary Fig. 55 | The missing-wedge correction on a simulated 3D map of $ModB_2C_2$ reconstructed from the low-pass filtered ±60° tilt series that with SNR=0.3 (A) A 3D object shown from two perpendicular views. The object was a generated from the crystal structure of $ModB_2C_2$, and the idea 3D was constructed from the tilt series of the noisy 2D projections (SNR=0.3) of the object from tilt angles in a range of ±90° in steps of 1.5°, while the initial 3D was reconstructed from angles of ±60° tilt series after low-pass filtered to 4 Å. (**B**) The projection of the object on the X-Z plane, and (**C**) the corresponding Fourier transform. (**D**) The initial 3D and iterative 3D maps, shown from perpendicular

views. (**E**) Their corresponding projections on the X-Z plane and the (**F**) Fourier transforms. The mask corresponding to ~3 times the molecular weight of $ModB_2C_2$ was generated from the low-passed filtered object (~40 Å). (**G**) The final corrected 3D after 1,000 cycles of iteration (round 1, Rd_1), shown from two perpendicular views, and (**H**) the projection along the X-Z plane and the corresponding (**I**) Fourier transform. (**J**) FSC curves of the iterative 3Ds against the object. The blue dashed line is the ideal FSC curve, calculated between the ideal 3D and the object. The dashed green line is the initial FSC, calculated between the initial 3D and the object. The rest solid lines are the iterative FSC calculated between the object and 3Ds after 1 (in purple), 2 (in blue), 10 (in cyan), 100 (in green), and 1,000 (in black) cycles of iteration. (**K**) The plot of the CCC (between the iterative 3D and the object) against the cycles of the iteration. All 3Ds were low-pass filtered to 8 Å. Bars: 20 nm.

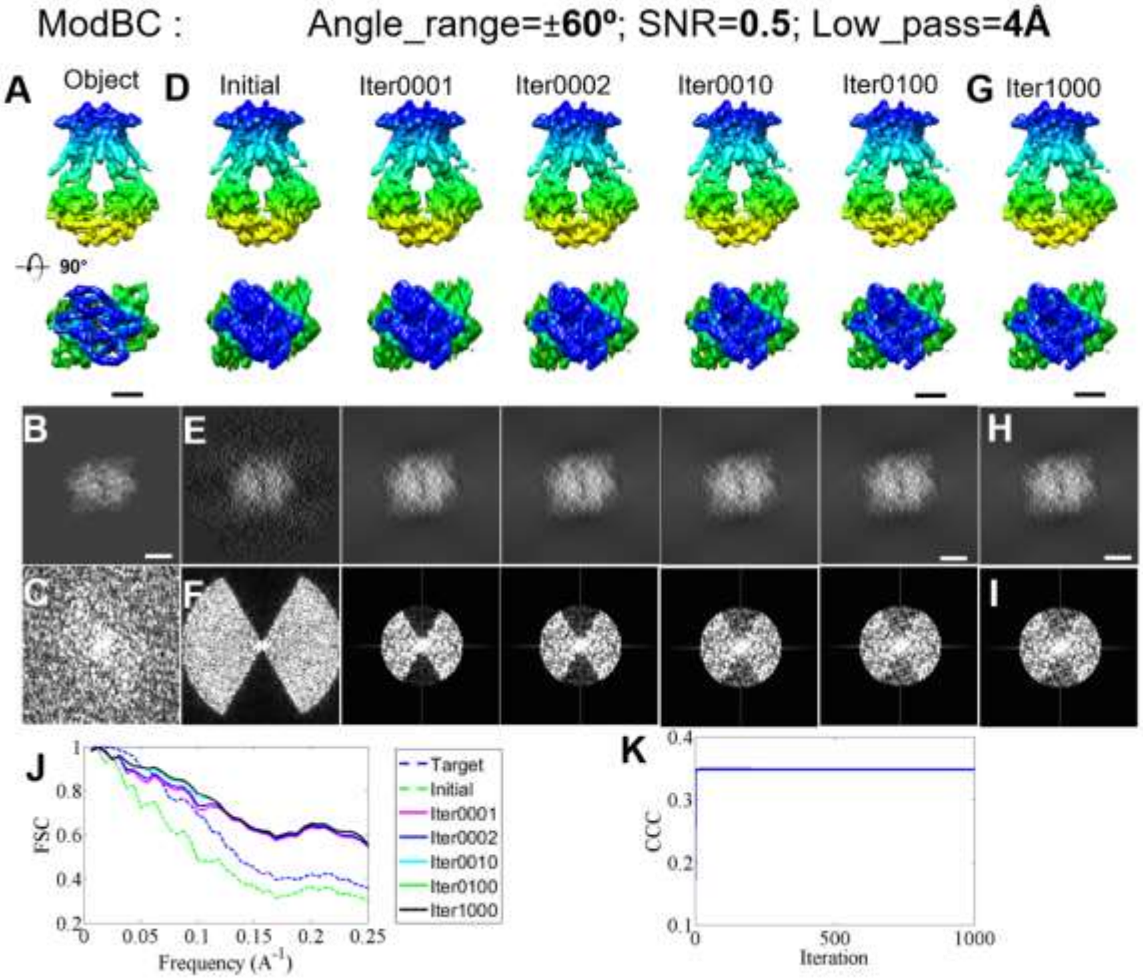

Supplementary Fig. 56 | The missing-wedge correction on a simulated 3D map of $ModB_2C_2$ reconstructed from the low-pass filtered ±60° tilt series that with SNR=0.5 (A) A 3D object shown from two perpendicular views. The object was a generated from the crystal structure of $ModB_2C_2$, and the idea 3D was constructed from the tilt series of the noisy 2D projections (SNR=0.5) of the object from tilt angles in a range of ±90° in steps of 1.5°, while the initial 3D was reconstructed from angles of ±60° tilt series after low-pass filtered to 4 Å. (**B**) The projection of the object on the X-Z plane, and (**C**) the

corresponding Fourier transform. (**D**) The initial 3D and iterative 3D maps, shown from perpendicular views. (**E**) Their corresponding projections on the X-Z plane and the (**F**) Fourier transforms. The mask corresponding to ~3 times the molecular weight of $ModB_2C_2$ was generated from the low-passed filtered object (~40 Å). (**G**) The final corrected 3D after 1,000 cycles of iteration (round 1, Rd_1), shown from two perpendicular views, and (**H**) the projection along the X-Z plane and the corresponding (**I**) Fourier transform. (**J**) FSC curves of the iterative 3Ds against the object. The blue dashed line is the ideal FSC curve, calculated between the ideal 3D and the object. The dashed green line is the initial FSC, calculated between the initial 3D and the object. The rest solid lines are the iterative FSC calculated between the object and 3Ds after 1 (in purple), 2 (in blue), 10 (in cyan), 100 (in green), and 1,000 (in black) cycles of iteration. (**K**) The plot of the CCC (between the iterative 3D and the object) against the cycles of the iteration. All 3Ds were low-pass filtered to 8 Å. Bars: 20 nm.

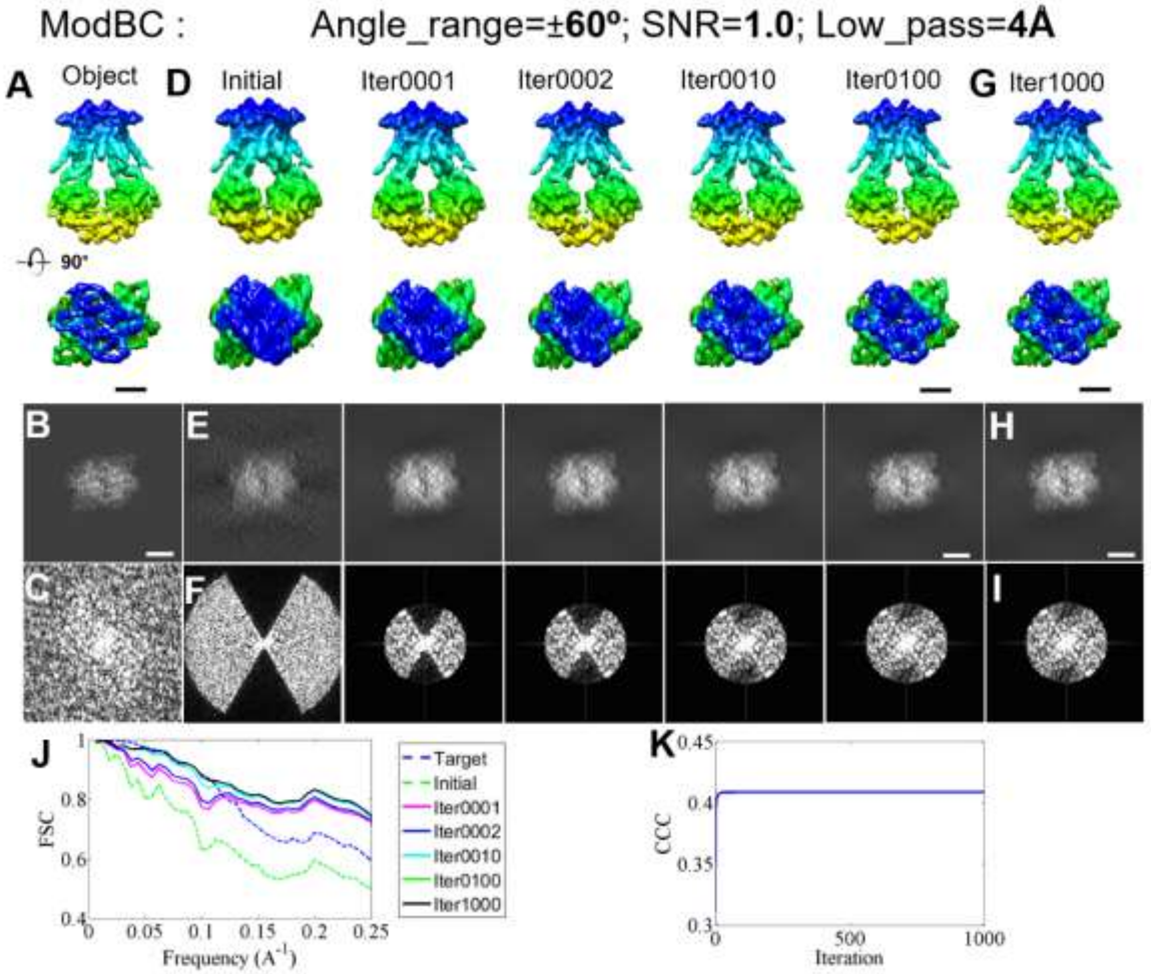

Supplementary Fig. 57 | The missing-wedge correction on a simulated 3D map of $ModB_2C_2$ reconstructed from the low-pass filtered ±60° tilt series that with SNR=1.0 (A) A 3D object shown from two perpendicular views. The object was a generated from the crystal structure of $ModB_2C_2$, and the idea 3D was constructed from the tilt series of the noisy 2D projections (SNR=1.0) of the object from tilt angles in a range of ±90° in steps of 1.5°, while the initial 3D was reconstructed from angles of ±60° tilt

series after low-pass filtered to 4 Å. (**B**) The projection of the object on the X-Z plane, and (**C**) the corresponding Fourier transform. (**D**) The initial 3D and iterative 3D maps, shown from perpendicular views. (**E**) Their corresponding projections on the X-Z plane and the (**F**) Fourier transforms. The mask corresponding to ~3 times the molecular weight of $ModB_2C_2$ was generated from the low-passed filtered object (~40 Å). (**G**) The final corrected 3D after 1,000 cycles of iteration (round 1, Rd_1), shown from two perpendicular views, and (**H**) the projection along the X-Z plane and the corresponding (**I**) Fourier transform. (**J**) FSC curves of the iterative 3Ds against the object. The blue dashed line is the ideal FSC curve, calculated between the ideal 3D and the object. The dashed green line is the initial FSC, calculated between the initial 3D and the object. The rest solid lines are the iterative FSC calculated between the object and 3Ds after 1 (in purple), 2 (in blue), 10 (in cyan), 100 (in green), and 1,000 (in black) cycles of iteration. (**K**) The plot of the CCC (between the iterative 3D and the object) against the cycles of the iteration. All 3Ds were low-pass filtered to 8 Å. Bars: 20 nm.

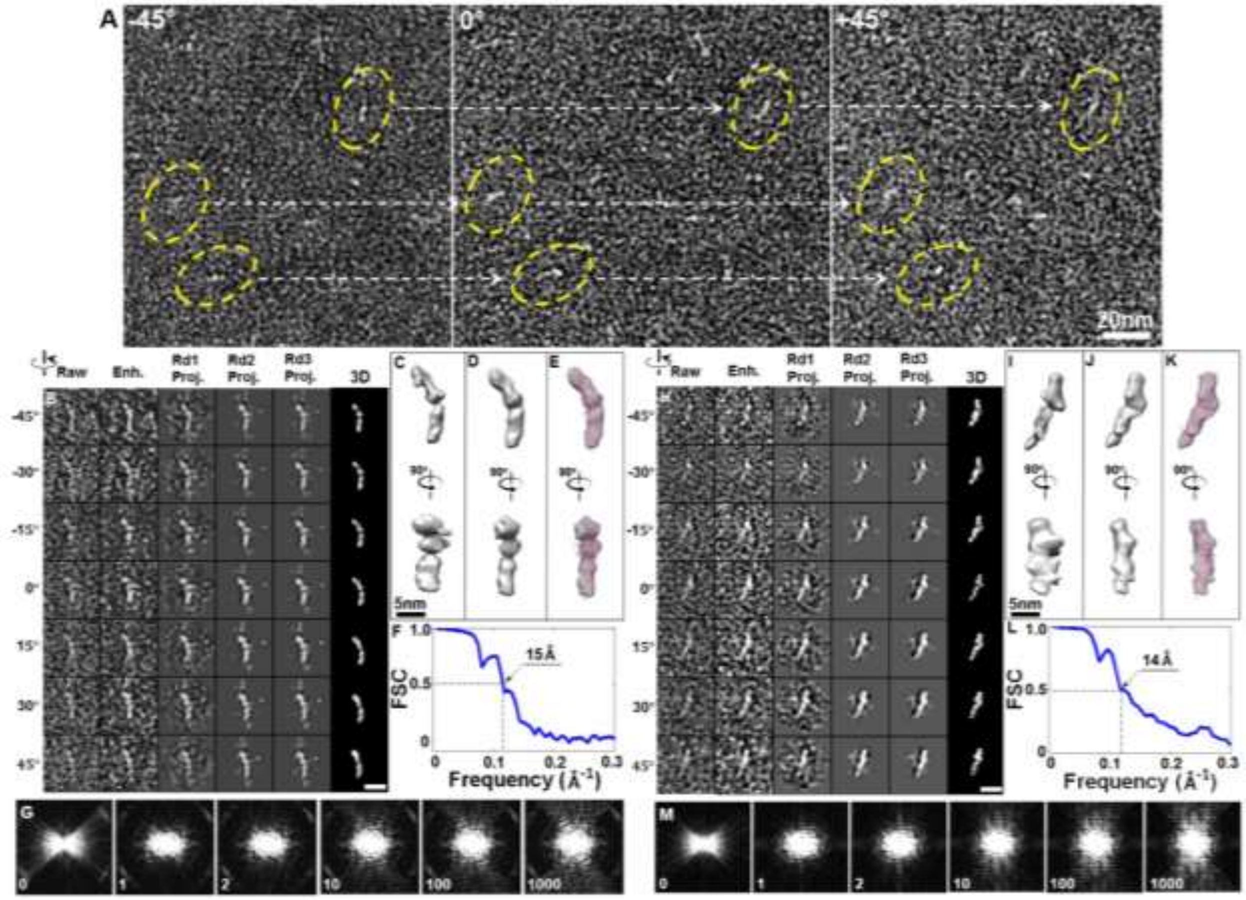

Supplementary Fig. 58 | Missing wedge correction on NS ET 3D reconstruction of individual CETP particles. (**A**) The tilt series of images of CETP particles are acquired by ET within a tilt angle range from -70° to +70° in steps of 1.5°.(66) The sample is prepared by OpNS.(62) Three representative tilt images are shown. (**B**) After CTF correction and contrast enhancement (via a low-pass filtering at 10 Å and a contrast enchantment program (67)), the tilt series of the images of an individual CETP particle within ±45° were

used for alignment and 3D reconstruction by IPET.(15) Representative tilt images before and after the contrast enhancement, projections of intermediate 3D reconstructions, and the final 3D are displayed sequentially from left to right. (**C**) Two orthogonal views of an IPET 3D reconstruction (initial 3D) and (**D**) its corrected 3D after 1000 rounds of iteration of missing-wedge correction (low-pass filtered at 8 Å). (**E**) The missing-wedge corrected 3D is rigid-body docked with CETP crystal structure (PDB entry 2OBD (71)). (**F**) The resolution of the IPET 3D at ~15 Å showed by FSC analyses (based on two density maps reconstructed from odd- and even-numbered tilt images). (**G**) The corresponding Fourier transforms of the X-Z plane projects of the initial, 3Ds after 0, 1, 2, 10, 100, and 1,000 iterations of missing-wedge correction (from left to right). (**H-M**) IPET 3D reconstruction and missing-wedge correction of a second individual CETP particle. Scale bars: 20 nm in A, 10 nm in B and H, and 5 nm in C and I.

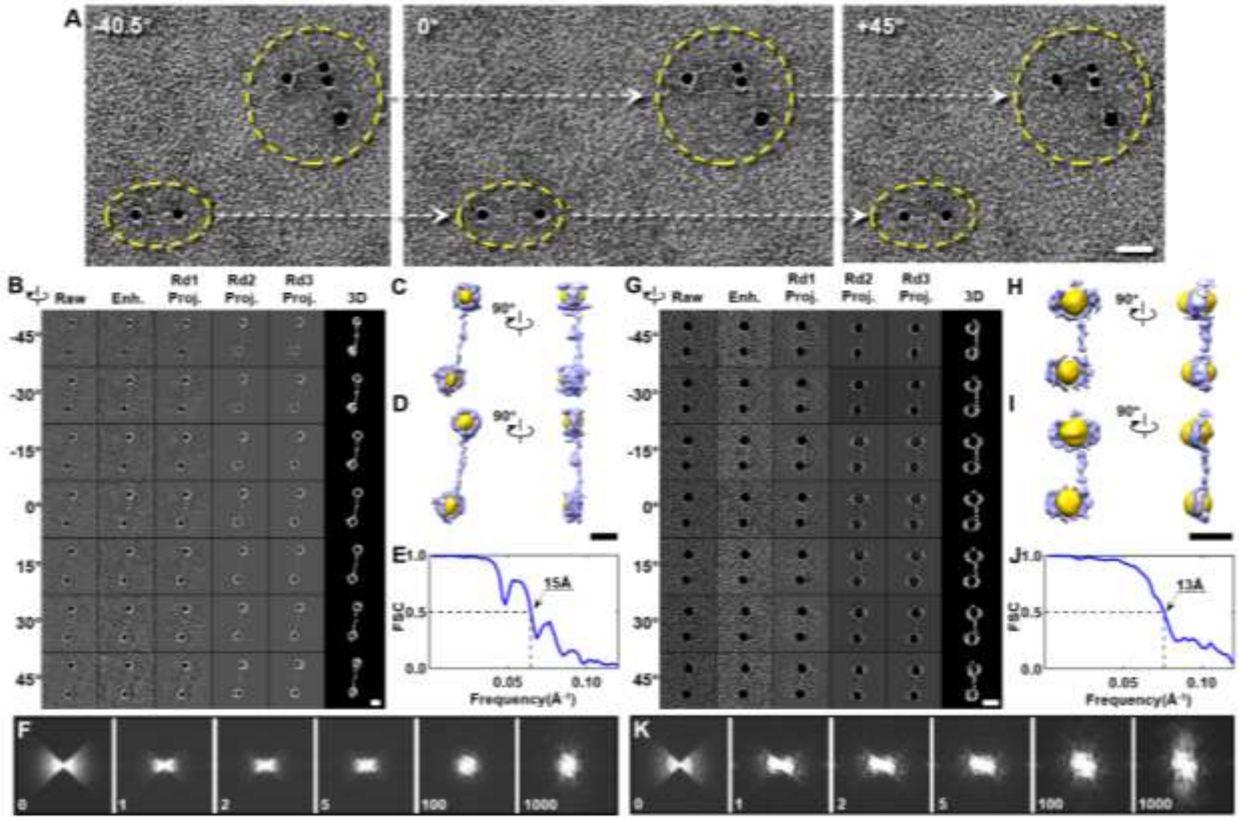

Supplementary Fig. 59 | Missing wedge correction on NS ET 3D reconstruction of individual DNA-nanogold conjugates. (**A**) The tilt series of images of DNA-nanogold conjugates (5) are acquired by ET within a tilt angle range from -60° to +60° in steps of 1.5°.(66) The sample is prepared by OpNS.(62) Three representative tilt images are shown (after a band-pass filtering from 15 to 2000 Å). (**B**) After CTF correction and contrast enhancement (via a low-pass filtering at 10 Å and a contrast enchantment program (67)), the tilt series of the images of an individual DNA-nanogold conjugate within ±45° were used for alignment and 3D reconstruction by IPET.(15) Representative tilt images before and after the contrast enhancement, projections of intermediate 3D reconstructions, and the final 3D are displayed sequentially from left to right. (**C**) Two orthogonal views of an IPET 3D reconstruction (initial 3D) and (**D**) its corrected 3D after 1000 rounds of iteration of missing-wedge correction (low-pass filtered at 15 Å). The contrast-

revised reconstruction was also shown (color gold, low-pass filtered at 30 Å). (**E**) The resolution of the IPET 3D at ~15 Å showed by FSC analyses (based on two density maps reconstructed from odd- and even-numbered tilt images). (**F**) The corresponding Fourier transforms of the X-Z plane projects of the initial, 3Ds after 0, 1, 2, 10, 100, and 1,000 iterations of missing-wedge correction (from left to right). (**G-K**) IPET 3D reconstruction and missing-wedge correction of a second individual DNA-nanogold conjugate. Scale bars: 20 nm in A, 10 nm in B through K.

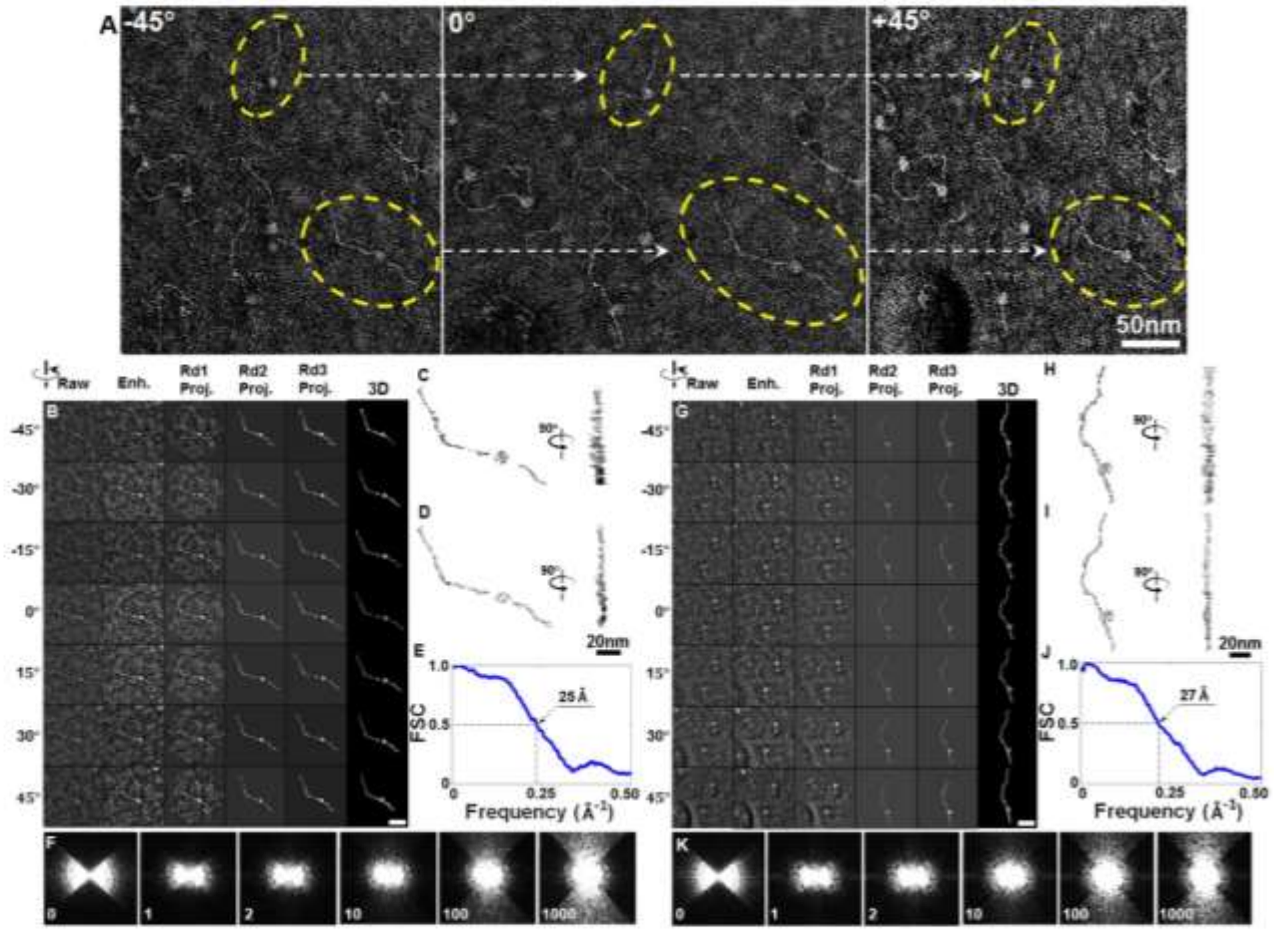

Supplementary Fig. 60 | Missing wedge correction on NS ET 3D reconstruction of individual nucleosome particles. (**A**) The tilt series of images of nucleosome particles are acquired by ET within a tilt angle range from -61.5° to +61.5° in steps of 1.5°.(66) The sample is prepared by OpNS.(62) Three representative tilt images are shown. (**B**) After CTF correction and contrast enhancement (via a low-pass filtering at 10 Å and a contrast enchantment program (67)), the tilt series of the images of an individual nucleosome particle within ±45° were used for alignment and 3D reconstruction by IPET.(15) Representative tilt images before and after the contrast enhancement, projections of intermediate 3D reconstructions, and the final 3D are displayed sequentially from left to right. (**C**) Two orthogonal views of an IPET 3D reconstruction (initial 3D) and (**D**) its corrected 3D after 1000 rounds of iteration of missing-wedge correction (low-pass filtered at 12 Å). (**E**) The resolution of the IPET 3D at ~25 Å showed by FSC analyses (based on two density maps reconstructed from odd- and even-numbered tilt images). (**F**) The

corresponding Fourier transforms of the X-Z plane projects of the initial, 3Ds after 0, 1, 2, 10, 100, and 1,000 iterations of missing-wedge correction (from left to right). (**G-K**) IPET 3D reconstruction and missing-wedge correction of a second individual nucleosome particle. Scale bars: 50 nm in A, B, and G, and 20 nm in D and I.

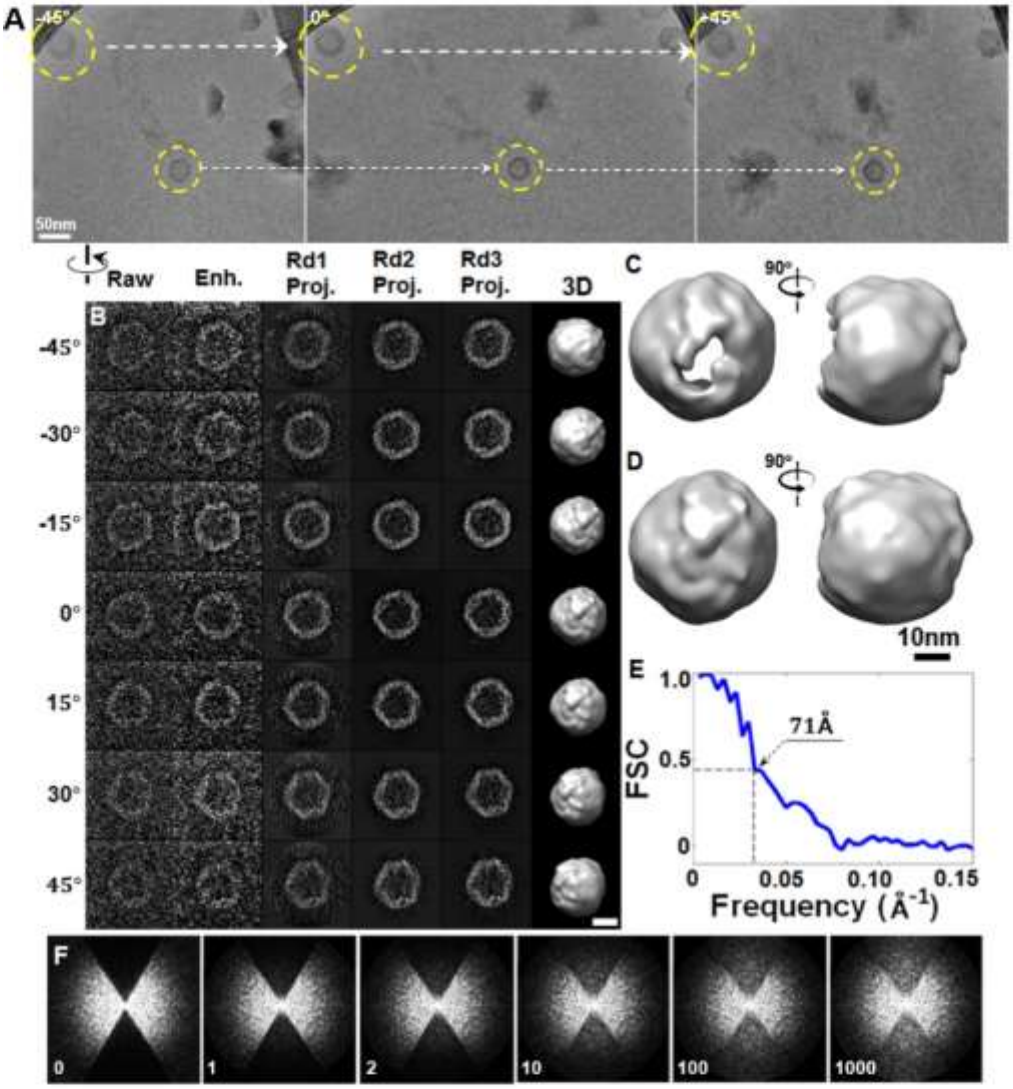

Supplementary Fig. 61 | Missing wedge correction on cryo-ET 3D reconstruction of individual liposome particles. (**A**) The tilt series of images of liposome particles are acquired by cryo-ET within a tilt angle range from -57° to +60°in steps of 1.5°.(66) The sample is prepared by cryo-EM.(27) Three representative tilt images are shown. (**B**) After CTF correction and contrast enhancement (via a low-pass filtering at 10 Å and a contrast enchantment program (67)), the tilt series of the images of an individual liposome particle within ±45° were used for alignment and 3D reconstruction by IPET.(15) Representative tilt images before and after the contrast enhancement, projections of intermediate 3D reconstructions, and the final 3D are displayed sequentially from left to right. (**C**) Two orthogonal views of an IPET 3D reconstruction (initial 3D) and (**D**) its corrected 3D after 1,000 rounds of iteration of missing-wedge

correction (low-pass filtered at 70 Å). (**E**) The resolution of the IPET 3D at ~71 Å showed by FSC analyses (based on two density maps reconstructed from odd- and even-numbered tilt images). (**F**) The corresponding Fourier transforms of the X-Z plane projects of the initial, 3Ds after 0, 1, 2, 10, 100, and 1,000 iterations of missing-wedge correction (from left to right). (**G-K**) IPET 3D reconstruction and missing-wedge correction of a second individual nucleosome particle. Scale bars: 50 nm in A, 20 nm in B, and 10 nm in D.

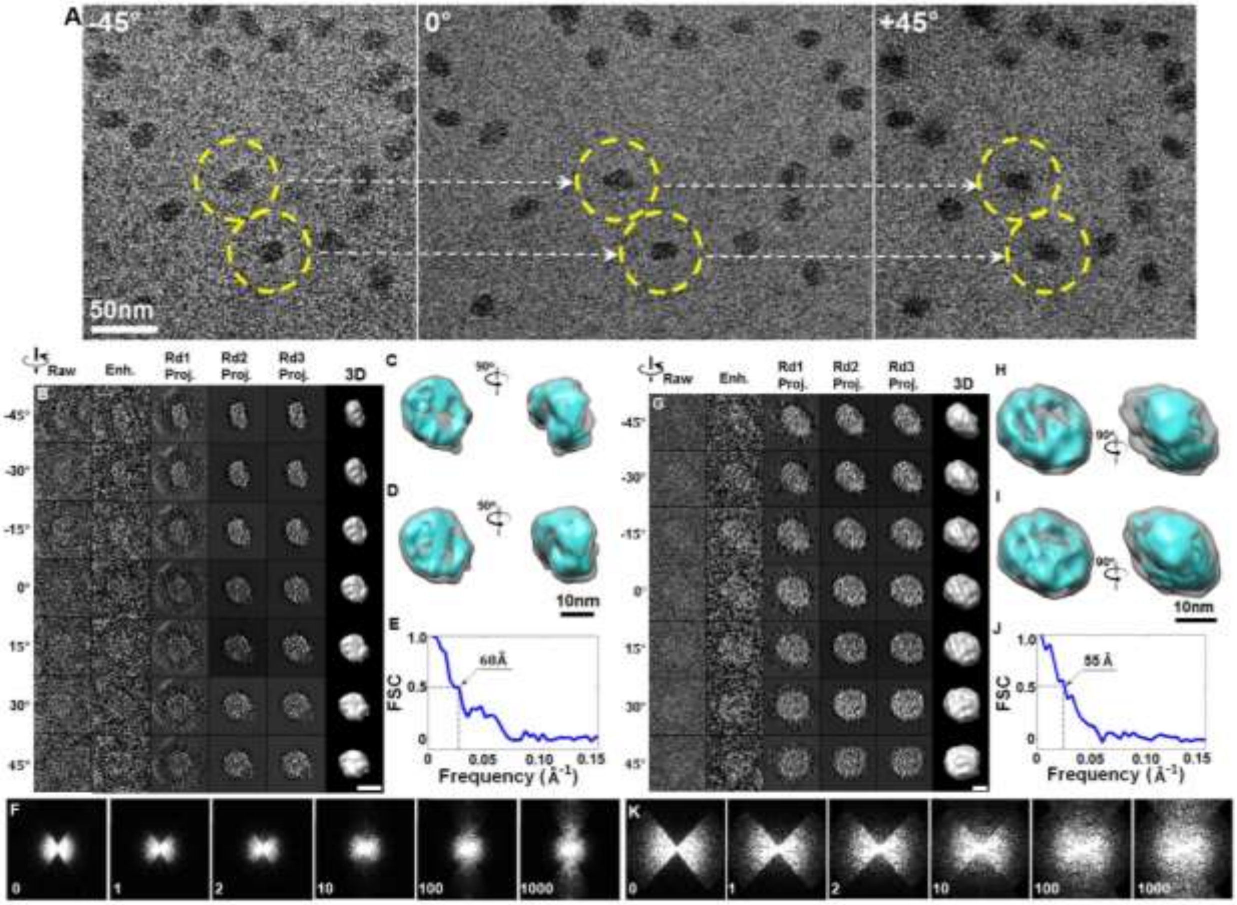

Supplementary Fig. 62 | Missing wedge correction on cryo-ET 3D reconstruction of individual LDL particles. (**A**) The tilt series of images of LDL particles (74) are acquired by cryo-ET within a tilt angle range from -57° to +52.5° in steps of 1.5°.(66) The sample is prepared by cryo-EM.(27) Three representative tilt images are shown. (**B**) After CTF correction and contrast enhancement (via a low-pass filtering at 10 Å and a contrast enchantment program (67)), the tilt series of the images of an individual liposome particle within ±45° were used for alignment and 3D reconstruction by IPET.(15) Representative tilt images before and after the contrast enhancement, projections of intermediate 3D reconstructions, and the final 3D are displayed sequentially from left to right. (**C**) Two orthogonal views of an IPET 3D reconstruction (initial 3D) and (**D**) its corrected 3D after 1,000 rounds of iteration of missing-wedge correction (low-pass filtered at 50 Å). (**E**) The resolution of the IPET 3D at ~68 Å showed by FSC analyses (based on two density maps reconstructed from odd- and even-numbered tilt images). (**F**) The

corresponding Fourier transforms of the X-Z plane projects of the initial, 3Ds after 0, 1, 2, 10, 100, and 1,000 iterations of missing-wedge correction (from left to right). (**G-K**) IPET 3D reconstruction and missing-wedge correction of a second individual LDL particle. Scale bars: 50 nm in A, 20 nm in B and G, and 10 nm in D and I.

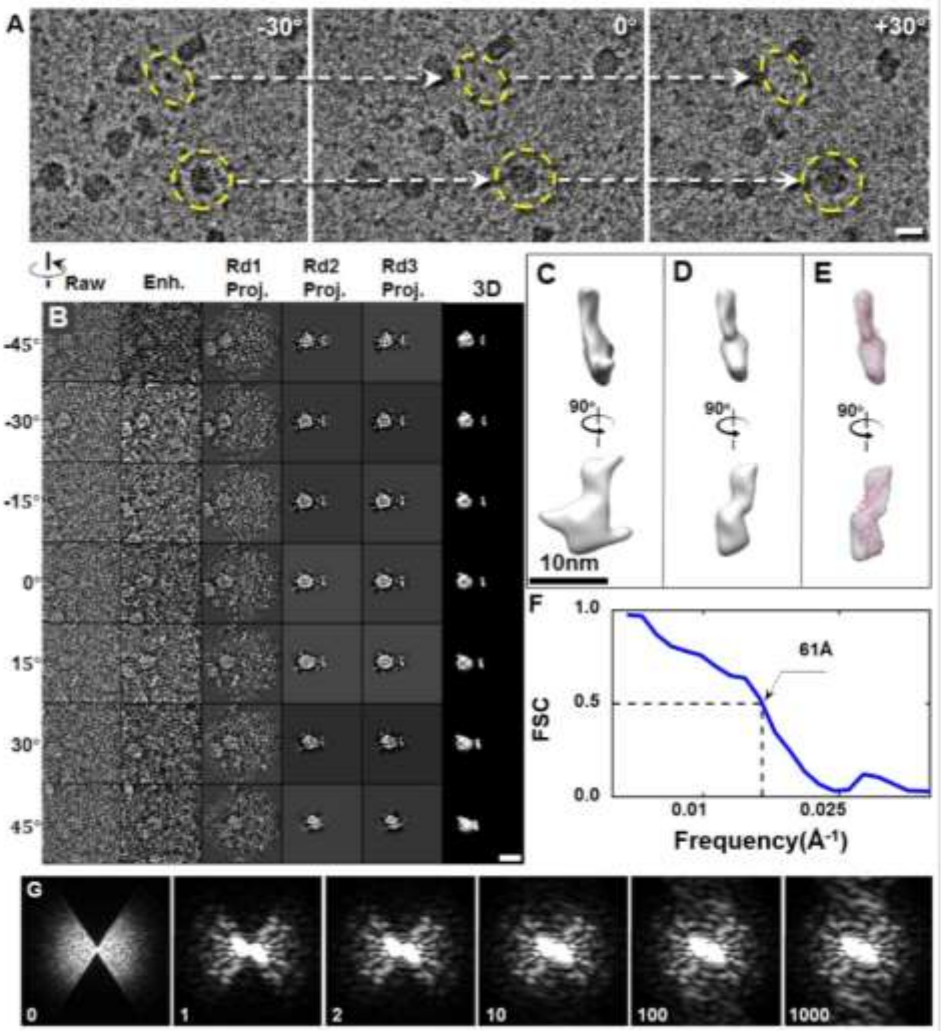

Supplementary Fig. 63 | Missing wedge correction on cryo-ET 3D reconstruction of individual CETP particles. (**A**) The tilt series of images of CETP particles (72) are acquired by cryo-ET within a tilt angle range from -49° to +65° in steps of 1.5°.(66) The sample of CETP mixed with LDL is prepared by cryo-EM.(27) Three representative tilt images are shown. (**B**) After CTF correction and contrast enhancement (with low-pass filtering at 10 Å and a contrast enchantment program (67)), the tilt series of the images of an individual liposome particle within ±45° were used for alignment and 3D reconstruction by IPET.(15) Representative tilt images before and after the contrast enhancement, projections of intermediate 3D reconstructions, and the final 3D are displayed sequentially from left to right. (**C**) Two orthogonal views of an IPET 3D reconstruction (initial 3D) and (**D**) its corrected 3D after 1,000 rounds of iteration of missing-

wedge correction (low-pass filtered at 30 Å). (**E**) The missing-wedge corrected 3D is rigid-body docked with CETP crystal structure (PDB entry 2OBD (71)). (**F**) The resolution of the IPET 3D at ~65 Å showed by FSC analyses (based on two density maps reconstructed from odd- and even-numbered tilt images). (**G**) The corresponding Fourier transforms of the X-Z plane projects of the initial, 3Ds after 0, 1, 2, 10, 100, and 1,000 iterations of missing-wedge correction (from left to right). Scale bars: 20nm in A, B and H, and 10 nm in E and I.
